# Supplementary material for: Socioeconomic, demographic and obstetric determinants of maternal near miss in Africa: A systematic review
Source: PLoS One. 2025 Feb 12;20(2):e0313897. doi: 10.1371/journal.pone.0313897 (PMC11819575; doi:10.1371/journal.pone.0313897)
Supplement: S1 File — (DOCX) [file pone.0313897.s001.docx]

Appendices

S 1: search strategies

1. (life threatening maternal morbidity[tw] OR severe obstetric[tw] OR severe maternal morbidity[tw] OR near miss[tw] OR severe acute maternal morbidity[tw]) AND ("socioeconomic status "[MeSH Terms] OR risk factors [tw]) AND ("Africa [tw])
2. ("maternal near miss" OR "severe maternal morbidity" OR "severe acute maternal morbidity" OR "severe obstetric" AND "Africa")
3. «severe maternal morbidity AND socioeconomic status AND Africa»
4. «severe maternal morbidity» OR «life threatening maternal morbidity » AND «socioeconomic status» OR «demographic» AND «determinants» OR «risk factors» AND «Africa»
5. « severe maternal morbidity» AND «Africa»
6. «maternal near miss» OR «severe maternal morbidity» OR «severe acute maternal morbidity» OR «life threatening maternal» AND «Africa»
7. ("maternal near miss" OR "severe maternal morbidity" OR "severe acute maternal morbidity" OR "severe obstetric" OR "life threatening maternal" OR "risk factors" and "Africa")
8. ((('life threatening' OR severe) NEAR/5 (obstetric OR 'maternal morbidity')):ab,ti OR 'near miss':ab,ti OR 'severe acute maternal morbidity':ab,ti) AND ('socioeconomic status OR 'risk factors':ab,ti) and ('Africa':ab,ti)
9. « maternal near miss» AND «Africa»

Note: First delay: delayed > 4 h at home; Second delay: take > 1 h to reach health facility on foot; Third Delay: treatment delayed > 2 h after admission
