# Supplementary material for: Socioeconomic, demographic and obstetric determinants of maternal near miss in Africa: A systematic review
Source: PLoS One. 2025 Feb 12;20(2):e0313897. doi: 10.1371/journal.pone.0313897 (PMC11819575; doi:10.1371/journal.pone.0313897)
Supplement: S1 Fig — (DOCX) [file pone.0313897.s002.docx]

**Identification of studies via databases and registers**

Records removed *before screening*:

Duplicate records removed (n =932)

Records identified from*:

Databases (n = 3001)

PubMed (n=649)

SCOPUS (n=122)

Science Direct (n=2230)

**Identification**

Records screened

(n = 2069)

Records excluded**

(n = 1814)

Reports sought for retrieval

(n =255)

**Screening**

Reports assessed for eligibility

(n = 255)

Reports excluded:

(n=230)

-Irrelevant results (n =148)

-Not specific to MMS (n=3)

- Excluded for out of context (n=78)

-No full text available (n=1)

Studies included in review

(n =25)

**Included**
