# Supplementary material for: Socioeconomic, demographic and obstetric determinants of maternal near miss in Africa: A systematic review
Source: PLoS One. 2025 Feb 12;20(2):e0313897. doi: 10.1371/journal.pone.0313897 (PMC11819575; doi:10.1371/journal.pone.0313897)
Supplement: S2 Table — (DOCX) [file pone.0313897.s005.docx]

**Table S3:** NIH Quality Assessment Tool for observational and case control studies**.**

| **Autor/ reference** | **Was the research question or objective in this paper clearly stated and appropriate?** | **Was the study population clearly specified and defined?** | **Did the authors include a sample size justification?** | **Were controls selected or recruited from the same or similar population that gave rise to the cases (including the same timeframe)?** | **Were the definitions, inclusion and exclusion criteria, algorithms or processes used to identify or select cases and controls valid, reliable, and implemented consistently across all study participants?** | **Were the cases clearly defined and differentiated from controls?** | **If less than 100 percent of eligible cases and/or controls were selected for the study, were the cases and/or controls randomly selected from those eligible?** | **Was there use of concurrent controls?** | **Were the investigators able to confirm that the exposure/risk occurred prior to the development of the condition or event that defined a participant as a case?** | **Were the measures of exposure/risk clearly defined, valid, reliable, and implemented consistently (including the same time period) across all study participants?** | **Were the assessors of exposure/risk blinded to the case or control status of participants?** | **Were key potential confounding variables measured and adjusted statistically in the analyses? If matching was used, did the investigators account for matching during study analysis?** | **Quality Rating (Good, Fair, or Poor)** |
| --- | --- | --- | --- | --- | --- | --- | --- | --- | --- | --- | --- | --- | --- |
| Kasahun AW and al 2017 | Yes | Yes | Yes | Yes | Yes | Yes | NA | Yes | Yes | Yes | NA | Yes | Good |
| Liyew EF and al 2018 | Yes | Yes | Yes | Yes | Yes | Yes | NA | NR | Yes | Yes | NA | Yes | Good |
| Bouchra A and al 2015 | Yes | Yes | Yes | Yes | Yes | Yes | NA | NR | Yes | Yes | NA | Yes | Good |
| Teshome HN and al 2022 | Yes | Yes | Yes | Yes | Yes | Yes | NA | NR | Yes | Yes | NA | Yes |  |
| Lemi K and al 2020 | Yes | Yes | Yes | Yes | CD | Yes | NA | NR | Yes | Yes | NA | Yes | Fair |
| Dessalegn FN and al 2020 | Yes | Yes | Yes | Yes | Yes | Yes | NA | NR | Yes | Yes | NA | Yes | Good |
| Adeoye IA and al 2013 | Yes | Yes | Yes | Yes | Yes | Yes | NA | Yes | Yes | Yes | NA | Yes | Good |
| Habtei A and al 2022 | Yes | Yes | Yes | Yes | Yes | Yes | NA | NR | Yes | Yes | NA | Yes | Good |
| Geze Tenaw S and al 2021 | Yes | Yes | Yes | Yes | Yes | Yes | NA | NR | Yes | Yes | NA | Yes | Good |
| Oppong SA and al 2019 | Yes | CD | CD | Yes | CD | Yes | NA | NR | Yes | Yes | NA | Yes | Fair |

*CD, cannot determine; NA, not applicable; NR, not reported
