# Supplementary material for: Socioeconomic, demographic and obstetric determinants of maternal near miss in Africa: A systematic review
Source: PLoS One. 2025 Feb 12;20(2):e0313897. doi: 10.1371/journal.pone.0313897 (PMC11819575; doi:10.1371/journal.pone.0313897)
Supplement: S3 Table — (DOCX) [file pone.0313897.s006.docx]

**Table S4:** Critical Appraisal Skills Program (CASP) quality-assessment tool for qualitative studies.

| Autor/reference | Was there a clearstatement of the aims ofthe research? | Is a qualitative methodology appropriate? | Was the researchdesign appropriate toaddress the aims of theresearch? | Was the recruitment strategy appropriate to the aims of the research? | Was the data collected in a way that addressed the research issue? | Has the relationship between researcher and participants been adequately considered? | Have ethical issues been taken into consideration? | Was the data analysis sufficiently rigorous? | Is there a clear statement of findings? | How valuable is the research? | Quality Rating (High, Medium or Low) |
| --- | --- | --- | --- | --- | --- | --- | --- | --- | --- | --- | --- |
| Lori JR and al 2012 | Yes | Yes | Yes | Yes | Yes | Can’t Tell | Yes | Yes | Yes | Yes | High |
| Storeng TK and al 2010 | Yes | Yes | Yes | Yes | Yes | Can’t Tell | Yes | Yes | Yes | Yes | High |
