# Supplementary material for: Socioeconomic, demographic and obstetric determinants of maternal near miss in Africa: A systematic review
Source: PLoS One. 2025 Feb 12;20(2):e0313897. doi: 10.1371/journal.pone.0313897 (PMC11819575; doi:10.1371/journal.pone.0313897)
Supplement: S4 Table — (DOCX) [file pone.0313897.s007.docx]

**Table S5:** NIH Quality Assessment Tool for Systematic review**.**

| Autor/reference | Is the review based on a focused question that is adequately formulated and described? | Were eligibility criteria for included and excluded studies predefined and specified? | Did the literature search strategy use a comprehensive, systematic approach? | Were titles, abstracts, and full-text articles dually and independently reviewed for inclusion and exclusion to minimize bias? | Was the quality of each included study rated independently by two or more reviewers using a standard method to appraise its internal validity? | Were the included studies listed along with important characteristics and results of each study? | Was publication bias assessed? | Was heterogeneity assessed? (This question applies only to meta-analyses.) | Quality Rating (Good, Fair, or Poor) |
| --- | --- | --- | --- | --- | --- | --- | --- | --- | --- |
| Mengist B and al 2021 | Yes | Yes | Yes | Yes | Yes | Yes | Yes | Yes | Good |
| Turi E and al 2020 | Yes | Yes | Yes | Yes | Yes | Yes | CD | Yes | Good |

*CD, cannot determine; NA, not applicable; NR, not reported
