# Supplementary material for: Socioeconomic, demographic and obstetric determinants of maternal near miss in Africa: A systematic review
Source: PLoS One. 2025 Feb 12;20(2):e0313897. doi: 10.1371/journal.pone.0313897 (PMC11819575; doi:10.1371/journal.pone.0313897)
Supplement: S5 Table — (DOCX) [file pone.0313897.s008.docx]

**Table S6 :** Complete list of studies identified in the literature search

| N° | **Publication Year** | **Author** | **Title** | **Raisons** |
| --- | --- | --- | --- | --- |
| 1 | 2018 | Tarekegne, Fitsum Eyayu; Padyab, Mojgan; Schröders, Julia; Stewart Williams, Jennifer | Sociodemographic and behavioral characteristics associated with self-reported diagnosed diabetes mellitus in adults aged 50+ years in Ghana and South Africa: results from the WHO-SAGE wave 1 | Irrelevant results |
| 2 | 2019 | Wright, Caradee Yael; Dominick, Friederike; Kapwata, Thandi; Bidassey-Manilal, Shalin; Engelbrecht, Jacobus Christoffel; Stich, Heribert; Mathee, Angela; Matooane, Mamopeli | Socio-economic, infrastructural and health-related risk factors associated with adverse heat-health effects reportedly experienced during hot weather in South Africa | Irrelevant results |
| 3 | 2015 | Benova, Lenka; Campbell, Oona M. R.; Ploubidis, George B. | Socio-economic inequalities in curative health-seeking for children in Egypt: analysis of the 2008 Demographic and Health Survey | Irrelevant results |
| 4 | 2020 | Baye, Kaleab; Laillou, Arnaud; Chitweke, Stanley | Socio-Economic Inequalities in Child Stunting Reduction in Sub-Saharan Africa | Irrelevant results |
| 5 | 2018 | Mekonnen, Fantahun Ayenew; Ambaw, Yohannes Abere; Neri, Genanew Timerga | Socio-economic determinants of anemia in pregnancy in North Shoa Zone, Ethiopia | Irrelevant results |
| 6 | 2020 | Mwangi, Kibachio Joseph; Mwenda, Valerian; Gathecha, Gladwell; Beran, David; Guessous, Idris; Ombiro, Oren; Ndegwa, Zachary; Masibo, Peninnah | Socio-economic and demographic determinants of non-communicable diseases in Kenya: a secondary analysis of the Kenya stepwise survey | Irrelevant results |
| 7 | 2016 | Muwonge, Richard; Ngo Mbus, Louise; Ngoma, Twalib; Gombe Mbalawa, Charles; Dolo, Amadou; da Ganda Manuel, Miraldina; and al | Socio-demographic and reproductive determinants of cervical neoplasia in seven sub-Sahara African countries | Irrelevant results |
| 8 | 2019 | Sogbanmu, Olufunso O.; Goon, Daniel T.; Obi, Larry C.; Iweriebor, Ben C.; Nwodo, Uchechukwu N.; Ajayi, Anthony I.; Okoh, Anthony I. | Socio-demographic and clinical determinants of late presentation among patients newly diagnosed with HIV in the Eastern Cape, South Africa | Irrelevant results |
| 9 | 2015 | Clouston, Sean A. P.; Yukich, Josh; Anglewicz, Phil | Social inequalities in malaria knowledge, prevention and prevalence among children under 5 years old and women aged 15-49 in Madagascar | Irrelevant results |
| 10 | 2003 | Haelterman, Edwige; Qvist, Rikke; Barlow, Patricia; Alexander, Sophie | Social deprivation and poor access to care as risk factors for severe pre-eclampsia | Irrelevant results |
| 11 | 2020 | Aheto, Justice Moses K. | Simultaneous quantile regression and determinants of under-five severe chronic malnutrition in Ghana | Irrelevant results |
| 12 | 2001 | Paruk, F.; Moodley, J. | Severe obstetric morbidity | Irrelevant results |
| 13 | 2018 | Tura, A.K.; Zwart, J.; Van Roosmalen, J.; Stekelenburg, J.; Van Den Akker, T.; Scherjon, S. | Severe maternal outcomes in eastern Ethiopia: Application of the adapted maternal near miss tool | Irrelevant results |
| 14 | 2021 | De Silva, Manarangi; Panisi, Leeanne; Lindquist, Anthea; Cluver, Catherine; Middleton, Anna; Koete, Benjamin; Vogel, Joshua P.; Walker, Susan; Tong, Stephen; Hastie, Roxanne | Severe maternal morbidity in the Asia Pacific: a systematic review and meta-analysis | Excluded for out of context |
| 15 | 2012 | van Mello, Norah M.; Zietse, Carlijn S.; Mol, Femke; Zwart, Joost J.; van Roosmalen, Jos; Bloemenkamp, Kitty W. and al | Severe maternal morbidity in ectopic pregnancy is not associated with maternal factors but may be associated with quality of care | Excluded for out of context |
| 16 | 2016 | Igbaruma, S.; Olagbuji, B.; Aderoba, A.; Kubeyinje, W.; Ande, B.; Imarengiaye, C. | Severe maternal morbidity in a general intensive care unit in Nigeria: clinical profiles and outcomes | Irrelevant results |
| 17 | 2000 | Prual, A.; Bouvier-Colle, M.-H.; De Bernis, L.; Bréart, G. | Severe maternal morbidity from direct obstetric causes in West Africa: Incidence and case fatality rates | Irrelevant results |
| 18 | 2009 | Murphy, Cliona M.; Murad, Khulood; Deane, Richard; Byrne, Bridgette; Geary, Michael P.; McAuliffe, Fionnuala M. | Severe maternal morbidity for 2004–2005 in the three Dublin maternity hospitals | Excluded for out of context |
| 19 | 2017 | Urquia, M.L.; Wanigaratne, S.; Ray, J.G.; Joseph, K.S. | Severe Maternal Morbidity Associated With Maternal Birthplace: A Population-Based Register Study | Excluded for out of context |
| 20 | 2015 | Urquia, M.L.; Glazier, R.H.; Mortensen, L.; Nybo-Andersen, A.-M.; Small, R.; Davey, M.-A.; Rööst, M.; Essén, B. | Severe maternal morbidity associated with maternal birthplace in three high-immigration settings | Excluded for out of context |
| 21 | 2022 | Eslier, Maxime; Deneux-Tharaux, Catherine; Sauvegrain, Priscille; Schmitz, Thomas; Luton, Dominique; Mandelbrot, Laurent; Estellat, Candice; Azria, Elie | Severe maternal morbidity among undocumented migrant women in the PreCARE prospective cohort study | Irrelevant results |
| 22 | 2000 | Hautvast, J. L.; Tolboom, J. J.; Kafwembe, E. M.; Musonda, R. M.; Mwanakasale, V.; van Staveren, W. A.; van 't Hof, M. A.; Sauerwein, R. W.; Willems, J. L.; Monnens, L. A. | Severe linear growth retardation in rural Zambian children: the influence of biological variables | Irrelevant results |
| 23 | 2020 | Tura, Abera Kenay; Scherjon, Sicco; Stekelenburg, Jelle; van Roosmalen, Jos; van den Akker, Thomas; Zwart, Joost | Severe Hypertensive Disorders of Pregnancy in Eastern Ethiopia: Comparing the Original WHO and Adapted sub-Saharan African Maternal Near-Miss Criteria | Included |
| 24 | 2019 | Ackerman, Christina M.; Platner, Marissa H.; Spatz, Erica S.; Illuzzi, Jessica L.; Xu, Xiao; Campbell, Katherine H.; Smith, Graeme N.; Paidas, Michael J.; Lipkind, Heather S. | Severe cardiovascular morbidity in women with hypertensive diseases during delivery hospitalization | Irrelevant results |
| 25 | 2020 | Proussaloglou, Ellie; Mueller, Ariel; Minhas, Ruby; Rana, Sarosh | Severe antepartum hypertension and associated peripartum morbidity among pregnant women in an urban tertiary care medical center | Excluded for out of context |
| 26 | 2021 | Johnson-Agbakwu, Crista E.; Eakin, Cortney M.; Bailey, Celeste V.; Sood, Shelly; Ali, Nyima; Doehrman, Pooja; Bhattarai, Bikash; Chambliss, Linda; Coonrod, Dean V. | Severe acute respiratory syndrome coronavirus 2: a canary in the coal mine for public safety net hospitals | Irrelevant results |
| 27 | 2004 | Gandhi, M.N.; Welz, T.; Ronsmans, C. | Severe acute maternal morbidity in rural South Africa | Included |
| 28 | 2002 | Vandecruys, H.I.B.; Pattinson, R.C.; Macdonald, A.P.; Mantel, G.D. | Severe acute maternal morbidity and mortality in the Pretoria Academic Complex: Changing patterns over 4 years | Irrelevant results |
| 29 | 2012 | Tamura, M.; Hinderaker, S. G.; Manzi, M.; Van Den Bergh, R.; Zachariah, R. | Severe acute maternal morbidity and associated deaths in conflict and post-conflict settings in Africa | Irrelevant results |
| 30 | 2016 | Adeyemi, Rasheed A.; Zewotir, Temesgen; Ramroop, Shaun | Semiparametric Multinomial Ordinal Model to Analyze Spatial Patterns of Child Birth Weight in Nigeria | Irrelevant results |
| 31 | 2019 | Pengpid, Supa; Peltzer, Karl | Self-rated oral health status and social and health determinants among community dwelling adults in Kenya | Irrelevant results |
| 32 | 2013 | Audureau, Etienne; Kahn, James G.; Besson, Marie-Hélène; Saba, Joseph; Ladner, Joël | Scaling up prevention of mother-to-child HIV transmission programs in sub-Saharan African countries: a multilevel assessment of site-, program- and country-level determinants of performance | Irrelevant results |
| 33 | 2016 | Maheu-Giroux, M.; Filippi, V.; Maulet, N.; Samadoulougou, S.; Castro, M.C.; Meda, N.; Pouliot, M.; Kirakoya-Samadoulougou, F. | Risk factors for vaginal fistula symptoms in Sub-Saharan Africa: A pooled analysis of national household survey data | Irrelevant results |
| 34 | 2005 | Wandabwa, Julius; Doyle, Pat; Paul, Kiondo; Wandabwa, Margaret A.; Aziga, Florence | Risk factors for severe abruptio placenta in Mulago Hospital, Kampala, Uganda | Irrelevant results |
| 35 | 2020 | Korb, Diane; Schmitz, Thomas; and al | Risk factors and high-risk subgroups of severe acute maternal morbidity in twin pregnancy: A population-based study | Excluded for out of context |
| 36 | 2004 | Gohou, V.; Ronsmans, C.; Kacou, L.; Yao, K.; Bohoussou, K.M.; Houphouet, B.; Bosso, P.; Diarra-Nama, A.J.; Bacci, A.; Filippi, V. | Responsiveness to life-threatening obstetric emergencies in two hospitals in Abidjan, Côte d'Ivoire | Irrelevant results |
| 37 | 2018 | Ramsay, Michèle; Crowther, Nigel J.; Agongo, Godfred; Ali, Stuart A.; Asiki, Gershim; Boua, Romuald P. and al | Regional and sex-specific variation in BMI distribution in four sub-Saharan African countries: The H3Africa AWI-Gen study | Irrelevant results |
| 38 | 2013 | Nattey, Cornelius; Masanja, Honorati; Klipstein-Grobusch, Kerstin | Relationship between household socio-economic status and under-five mortality in Rufiji DSS, Tanzania | Irrelevant results |
| 39 | 2020 | Nakimuli, Annettee; Starling, Jennifer E.; Nakubulwa, Sarah; Namagembe, Imelda; Sekikubo, Musa; Nakabembe, Eve; and al | Relative impact of pre-eclampsia on birth weight in a low resource setting: A prospective cohort study | Irrelevant results |
| 40 | 2019 | Bishop, D and al | Maternal and neonatal outcomes after caesarean delivery in the African Surgical Outcomes Study: a 7-day prospective observational cohort study | Irrelevant results |
| 41 | 2012 | Briand, Valérie; Dumont, Alexandre; Abrahamowicz, Michal; Sow, Amadou; Traore, Mamadou; Rozenberg, Patrick; Watier, Laurence; Fournier, Pierre | Maternal and perinatal outcomes by mode of delivery in senegal and mali: a cross-sectional epidemiological survey | Included |
| 42 | 2017 | Kalisa, R.; Rulisa, S.; van Roosmalen, J.; van den Akker, T. | Maternal and perinatal outcome after previous caesarean section in rural Rwanda | Included |
| 43 | 2020 | Minehart, Rebecca D.; Jackson, Jaleesa; Daly, Jaime | Racial Differences in Pregnancy-Related Morbidity and Mortality | Irrelevant results |
| 44 | 2019 | Leonard, Stephanie A.; Main, Elliott K.; Scott, Karen A.; Profit, Jochen; Carmichael, Suzan L. | Racial and ethnic disparities in severe maternal morbidity prevalence and trends | Excluded for out of context |
| 45 | 2019 | Tannor, Elliot K.; Norman, Betty R.; Adusei, Kwame K.; Sarfo, Fred S.; Davids, Mogamat R.; Bedu-Addo, George | Quality of life among patients with moderate to advanced chronic kidney disease in Ghana - a single centre study | Irrelevant results |
| 46 | 2018 | Soma-Pillay, Priya; Makin, Jennifer D.; Pattinson, Robert C. | Quality of life 1 year after a maternal near-miss event | Irrelevant results |
| 47 | 2017 | Howell, Elizabeth A.; Zeitlin, Jennifer | Quality of Care and Disparities in Obstetrics | Irrelevant results |
| 48 | 2022 | Ndicunguye, Victor Mivumbi; El Ayadi, Alison M. | Quality Improvement Models and Methods for Maternal Health in Lower-Resource Settings | Irrelevant results |
| 49 | 2020 | Folayan, Morenike Oluwatoyin; El Tantawi, Maha; Oginni, Ayodeji; Adeniyi, Abiola; Alade, Michael; Finlayson, Tracy L. | Psychosocial, education, economic factors, decision-making ability, and caries status of mothers of children younger than 6 years in suburban Nigeria | Irrelevant results |
| 50 | 2019 | Benova, Lenka; Owolabi, Onikepe; Radovich, Emma; Wong, Kerry L. M.; Macleod, David; Langlois, Etienne V.; Campbell, Oona M. R. | Provision of postpartum care to women giving birth in health facilities in sub-Saharan Africa: A cross-sectional study using Demographic and Health Survey data from 33 countries | Irrelevant results |
| 51 | 2019 | Senekal, Marjanne; Nel, Johanna H.; Malczyk, Sonia; Drummond, Linda; Harbron, Janetta; Steyn, Nelia P. | Provincial Dietary Intake Study (PDIS): Prevalence and Sociodemographic Determinants of the Double Burden of Malnutrition in A Representative Sample of 1 to Under 10-Year-Old Children from Two Urbanized and Economically Active Provinces in South Africa | Irrelevant results |
| 52 | 2014 | Akinyemi, Rufus O.; Allan, Louise; Owolabi, Mayowa O.; Akinyemi, Joshua O.; Ogbole, Godwin; Ajani, Akinlolu; Firbank, Michael; Ogunniyi, Adesola; Kalaria, Raj N. | Profile and determinants of vascular cognitive impairment in African stroke survivors: the CogFAST Nigeria Study | Irrelevant results |
| 53 | 2018 | Mohamed, Shukri F.; Mutua, Martin K.; Wamai, Richard; Wekesah, Frederick; Haregu, Tilahun; Juma, Pamela; Nyanjau, Loise; Kyobutungi, Catherine; Ogola, Elijah | Prevalence, awareness, treatment and control of hypertension and their determinants: results from a national survey in Kenya | Irrelevant results |
| 54 | 2018 | Monakali, Sizeka; Ter Goon, Daniel; Seekoe, Eunice; Owolabi, Eyitayo O. | Prevalence, awareness, control and determinants of hypertension among primary health care professional nurses in Eastern Cape, South Africa | Irrelevant results |
| 55 | 2017 | Bosu, William K.; Aheto, Justice M. K.; Zucchelli, Eugenio; Reilly, Siobhan | Prevalence, awareness, and associated risk factors of hypertension in older adults in Africa: a systematic review and meta-analysis protocol | Irrelevant results |
| 56 | 2015 | Folayan, Morenike O.; Kolawole, Kikelomo A.; Oziegbe, Elizabeth O.; Oyedele, Titus; Oshomoji, Olusegun V.; Chukwumah, Nneka M.; Onyejaka, Nneka | Prevalence, and early childhood caries risk indicators in preschool children in suburban Nigeria | Irrelevant results |
| 57 | 2017 | Hamed, Ahmed; Yousf, Fouad; Hussein, Mohamed M. | Prevalence of nocturnal enuresis and related risk factors in school-age children in Egypt: an epidemiological study | Irrelevant results |
| 58 | 2016 | Nansubuga, Elizabeth; Ayiga, Natal; Moyer, Cheryl A. | Prevalence of maternal near miss and community-based risk factors in Central Uganda | Included |
| 59 | 2015 | Jenkins, Rachel; Omollo, Raymond; Ongecha, Michael; Sifuna, Peter; Othieno, Caleb; Ongeri, Linnet; Kingora, James; Ogutu, Bernhards | Prevalence of malaria parasites in adults and its determinants in malaria endemic area of Kisumu County, Kenya | Irrelevant results |
| 60 | 2017 | Brennan-Olsen, Sharon L.; Cook, S.; Leech, M. T.; Bowe, S. J.; Kowal, P.; Naidoo, N.; Ackerman, I. N.; Page, R. S.; Hosking, S. M.; Pasco, J. A.; Mohebbi, M. | Prevalence of arthritis according to age, sex and socioeconomic status in six low and middle income countries: analysis of data from the World Health Organization study on global AGEing and adult health (SAGE) Wave 1 | Irrelevant results |
| 61 | 2019 | Gebreweld, Angesom; Ali, Neima; Ali, Radiya; Fisha, Temesgen | Prevalence of anemia and its associated factors among children under five years of age attending at Guguftu health center, South Wollo, Northeast Ethiopia | Irrelevant results |
| 62 | 2019 | Enawgaw, Bamlaku; Birhanie, Meseret; Terefe, Betelihem; Asrie, Fikir | Prevalence of Anemia and Iron Deficiency Among Pregnant Women Attending Antenatal Care Service at University of Gondar Hospital, Northwest Ethiopia | Irrelevant results |
| 63 | 2017 | Getaneh, Zegeye; Enawgaw, Bamlaku; Engidaye, Getabalew; Seyoum, Masresha; Berhane, Muruts; Abebe, Zegeye; Asrie, Fikir; Melku, Mulugeta | Prevalence of anemia and associated factors among school children in Gondar town public primary schools, northwest Ethiopia: A school-based cross-sectional study | Irrelevant results |
| 64 | 2011 | Senbanjo, Idowu O.; Oshikoya, Kazeem A.; Odusanya, Olumuyiwa O.; Njokanma, Olisamedua F. | Prevalence of and risk factors for stunting among school children and adolescents in Abeokuta, southwest Nigeria | Irrelevant results |
| 65 | 2021 | Turyare, Mahad Dahir; Mativo, Japheth Nzioki; Kerich, Mary; Ndiritu, Alex Karuiru | Prevalence and socio-demographic determinants of diarrhea among children below 5 years in Bondhere district Somalia | Irrelevant results |
| 66 | 2013 | Kinyanda, Eugene; Kizza, Ruth; Abbo, Catherine; Ndyanabangi, Sheila; Levin, Jonathan | Prevalence and risk factors of depression in childhood and adolescence as seen in four districts of North-Eastern Uganda | Irrelevant results |
| 67 | 2019 | Nambiema, Aboubakari; Robert, Alexie; Yaya, Issifou | Prevalence and risk factors of anemia in children aged from 6 to 59 months in Togo: analysis from Togo demographic and health survey data, 2013-2014 | Irrelevant results |
| 68 | 2019 | Yarfi, Cosmos; Elekusi, Cephas; Banson, Adjoa Nkrumah; Angmorterh, Seth Kwadjo; Kortei, Nii Korley; Ofori, Eric Kwasi | Prevalence and predisposing factors of brachial plexus birth palsy in a regional hospital in Ghana: a five year retrospective study | Irrelevant results |
| 69 | 2021 | Sarfo, Fred Stephen; Ovbiagele, Bruce | Prevalence and Predictors of Multivitamin Supplement Use After Stroke in Ghana | Irrelevant results |
| 70 | 2022 | Nwabueze, Ngozi; Docheva, Nikolina; Arenas, Gabriel; Mueller, Ariel; Lopes Perdigao, Joana; Rana, Sarosh | Prevalence and management of severe intrapartum hypertension in patients with preeclampsia at an urban tertiary care medical center | Excluded for out of context |
| 71 | 2020 | Jabbi, Alhaji; Ndow, Bakary; Senghore, Thomas; Sanyang, Edrisa; Kargbo, Jainaba Catherina; Bass, Paul | Prevalence and factors associated with intimate partner violence against women in The Gambia: a population-based analysis | Irrelevant results |
| 72 | 2019 | Chilunga, Felix P.; Henneman, Peter; Meeks, Karlijn Ac; Beune, Erik; Requena-Méndez, Ana; Smeeth, Liam; and al | Prevalence and determinants of type 2 diabetes among lean African migrants and non-migrants: the RODAM study | Irrelevant results |
| 73 | 2017 | Jallow, Isatou K.; Britton, John; Langley, Tessa | Prevalence and determinants of tobacco use among young people in The Gambia | Irrelevant results |
| 74 | 2013 | Belfki, Hanen; Ben Ali, Samir; Aounallah-Skhiri, Hajer; Traissac, Pierre; Bougatef, Souha; Maire, Bernard; Delpeuch, Francis; Achour, Noureddine; Ben Romdhane, Habiba | Prevalence and determinants of the metabolic syndrome among Tunisian adults: results of the Transition and Health Impact in North Africa (TAHINA) project | Irrelevant results |
| 75 | 2020 | Ekholuenetale, Michael; Barrow, Amadou | Prevalence and determinants of self-reported high blood pressure among women of reproductive age in Benin: a population-based study | Irrelevant results |
| 76 | 2013 | Pessinaba, Soulemane; Mbaye, Alassane; Yabeta, Grace-A.-Dieu; Kane, Adama; Ndao, Cheikh Tidiane; Ndiaye, Mouhamadou Bambaand al | Prevalence and determinants of hypertension and associated cardiovascular risk factors: data from a population-based, cross-sectional survey in Saint Louis, Senegal | Irrelevant results |
| 77 | 2021 | Jeyakumar, Angeline; Jungari, Suresh; Nair, Raji; Menon, Pramila; Babar, Prasad; Bhushan, Barai; Yogita, Hulsurkar; Ali, Janan; Saddichha, Marathe; Bhagyashree, Mitragotri; Monika, Phadake; Sakshi, Sneha | Prevalence and Determinants of Early Initiation (EI), Exclusive Breastfeeding (EBF), and Prelacteal Feeding among Children Aged 0-24 Months in Slums of Pune City, in Maharashtra | Irrelevant results |
| 78 | 2015 | Oppong Asante, Kwaku; Andoh-Arthur, Johnny | Prevalence and determinants of depressive symptoms among university students in Ghana | Irrelevant results |
| 79 | 2019 | Ademola, A. D.; Boima, V.; Odusola, A. O.; Agyekum, F.; Nwafor, C. E.; Salako, B. L. | Prevalence and determinants of depression among patients with hypertension: A cross-sectional comparison study in Ghana and Nigeria | Irrelevant results |
| 80 | 2018 | Ngu, Roland Cheofor; Feteh, Vitalis Fambombi; Kika, Belmond Tse; F, Emade Ketchemen Nerice; Ayeah, Chia Mark; Chifor, Theresia; and al | Prevalence and Determinants of Antibiotic Self-Medication among Adult Patients with Respiratory Tract Infections in the Mboppi Baptist Hospital, Douala, Cameroon: A Cross-Sectional Study | Irrelevant results |
| 81 | 2014 | Weobong, Benedict; Soremekun, Seyi; Ten Asbroek, Augustinus Ha; Amenga-Etego, Seeba; Danso, Samuel; Owusu-Agyei, Seth and al | Prevalence and determinants of antenatal depression among pregnant women in a predominantly rural population in Ghana: the DON population-based study | Irrelevant results |
| 82 | 2021 | Liyew, Alemneh Mekuriaw; Tesema, Getayeneh Antehunegn; Alamneh, Tesfa Sewunet; Worku, Misganaw Gebrie; Teshale, Achamyeleh Birhanu; Alem, Adugnaw Zeleke; and al | Prevalence and determinants of anemia among pregnant women in East Africa; A multi-level analysis of recent Demographic and Health Surveys | Irrelevant results |
| 83 | 2020 | Elmardi, Khalid Abdelmutalab; Adam, Ishag; Malik, Elfatih Mohammed; Abdelrahim, Tarig Abdalla; Elhag, Mousab Siddig; Ibrahim, Abdalla Ahmed and al | Prevalence and determinants of anaemia in women of reproductive age in Sudan: analysis of a cross-sectional household survey | Irrelevant results |
| 84 | 2019 | Adom, Theodosia; De Villiers, Anniza; Puoane, Thandi; Kengne, André Pascal | Prevalence and correlates of overweight and obesity among school children in an urban district in Ghana | Irrelevant results |
| 85 | 2019 | Alemayehu, Mihiretu; Meskele, Mengistu; Alemayehu, Bereket; Yakob, Bereket | Prevalence and correlates of anemia among children aged 6-23 months in Wolaita Zone, Southern Ethiopia | Irrelevant results |
| 86 | 2018 | Nigatu, Getnet; Assefa Woreta, Solomon; Akalu, Temesgen Yihunie; Yenit, Melaku Kindie | Prevalence and associated factors of underweight among children 6-59 months of age in Takusa district, Northwest Ethiopia | Irrelevant results |
| 87 | 2018 | Kasahun, A.W.; Wako, W.G. | Predictors of maternal near miss among women admitted in Gurage zone hospitals, South Ethiopia, 2017: A case control study | Included |
| 88 | 2015 | Adeniran, A. S.; Bolaji, B. O.; Fawole, A. A.; Oyedepo, O. O. | Predictors of maternal mortality among critically ill obstetric patients | Irrelevant results |
| 89 | 2011 | Ogunlesi, Tinuade A.; Ogunfowora, Olusoga B. | Predictors of acute bilirubin encephalopathy among Nigerian term babies with moderate-to-severe hyperbilirubinaemia | Irrelevant results |
| 90 | 2014 | Abalos, E.; Cuesta, C.; Carroli, G.; Qureshi, Z.; Widmer, M.; Vogel, J.P.; Souza, J.P.; WHO Multicountry Survey on Maternal and Newborn Health Research Network | Pre-eclampsia, eclampsia and adverse maternal and perinatal outcomes: a secondary analysis of the World Health Organization Multicountry Survey on Maternal and Newborn Health. | Irrelevant results |
| 91 | 2013 | Spector, J. | Practical criteria for maternal near miss needed for low-income settings | Irrelevant results |
| 92 | 2010 | Yusuf, Oyindamola B.; Adeoye, Babatunde W.; Oladepo, Oladimeji O.; Peters, David H.; Bishai, David | Poverty and fever vulnerability in Nigeria: a multilevel analysis | Irrelevant results |
| 93 | 2015 | Anbazhagan, Akila; Harper, Ann | Postpartum pyrexia | Irrelevant results |
| 94 | 2021 | Gresh, Ashley; Cohen, Megan; Anderson, Jean; Glass, Nancy | Postpartum care content and delivery throughout the African continent: An integrative review | Irrelevant results |
| 95 | 2020 | Small, Maria J.; Gondwe, Kaboni W.; Brown, Haywood L. | Post-Traumatic Stress Disorder and Severe Maternal Morbidity | Irrelevant results |
| 96 | 2017 | Abebe, Zegeye; Gebeye, Ejigu; Tariku, Amare | Poor dietary diversity, wealth status and use of un-iodized salt are associated with goiter among school children: a cross-sectional study in Ethiopia | Irrelevant results |
| 97 | 2018 | Marcellin, Louis; Delorme, Pierre; Bonnet, Marie Pierre; Grange, Gilles; Kayem, Gilles; Tsatsaris, Vassilis; Goffinet, François | Placenta percreta is associated with more frequent severe maternal morbidity than placenta accreta | Excluded for out of context |
| 98 | 2019 | Zanconato, G.; Cavaliere, E.; Mariotto, O.; Zatti, N. | Perinatal outcome of severe obstetric complications:findings of a 10-year hospital-based surveillance study in Italy | Irrelevant results |
| 99 | 2011 | Iyoke, C. A.; Ifeadike, C. O.; Nnebue, C. C.; Onah, H. E.; Ezugwu, F. O. | Perception and care-seekling behaviour for post partum morbidity among mothers in Enugu south east, Nigeria | Irrelevant results |
| 100 | 2016 | Durst, Jennifer K.; Tuuli, Methodius G.; Stout, Molly J.; Macones, George A.; Cahill, Alison G. | Degree of obesity at delivery and risk of preeclampsia with severe features | Irrelevant results |
| 101 | 2021 | Sabol, Bethany A.; Porcelli, Bree; Diveley, Emily; Meyenburg, Kaytelyn; Woolfolk, Candice; Rosenbloom, Joshua I. and al | Defining the risk profile of women with stage 1 hypertension: a time to event analysis | Irrelevant results |
| 102 | 2021 | Phillips, Jaclyn M.; Hacker, Francis; Lemon, Lara; Simhan, Hyagriv N. | Correlation between hemorrhage risk prediction score and severe maternal morbidity | Irrelevant results |
| 103 | 2018 | Hitti, Jane; Sienas, Laura; Walker, Suzan; Benedetti, Thomas J.; Easterling, Thomas | Contribution of hypertension to severe maternal morbidity | Excluded for out of context |
| 104 | 2012 | Salim, Raed; Mfra, Ali; Garmi, Gali; Shalev, Eliezer | Comparison of intrapartum outcome among immigrant women from Ethiopia and the general obstetric population in Israel | Irrelevant results |
| 105 | 2009 | Lombaard, Hennie; Pattinson, Robert C. | Common errors and remedies in managing postpartum haemorrhage | Irrelevant results |
| 106 | 2007 | Stanton, C.; Holtz, S. A.; Ahmed, S. | Challenges in measuring obstetric fistula | Irrelevant results |
| 107 | 2018 | Hehir, Mark P.; Ananth, Cande V.; Siddiq, Zainab; Flood, Karen; Friedman, Alexander M.; D’Alton, Mary E. | Cesarean delivery in the United States 2005 through 2014: a population-based analysis using the Robson 10-Group Classification System | Irrelevant results |
| 108 | 2017 | Soma-Pillay, P.; Suleman, F. E.; Makin, J. D.; Pattinson, R. C. | Cerebral white matter lesions after pre-eclampsia | Irrelevant results |
| 109 | 2016 | Razali, Nuguelis; Md Latar, Ida Lilywaty; Chan, Yoo Kuen; Omar, Siti Zawiah; Tan, Peng Chiong | Carbetocin compared to oxytocin in emergency cesarean section: a randomized trial | Irrelevant results |
| 110 | 2019 | Reid, M J A; Arinaminpathy, N; Bloom, A; Bloom, B R; Boehme, C; Chaisson, R and al | Building a tuberculosis-free world: The Lancet Commission on tuberculosis | Irrelevant results |
| 111 | 2016 | Howell, Elizabeth A.; Egorova, Natalia; Balbierz, Amy; Zeitlin, Jennifer; Hebert, Paul L. | Black-white differences in severe maternal morbidity and site of care | Irrelevant results |
| 112 | 2015 | Park, Christina K.; Krebs, Lynette; Lutsiv, Olha; van Blyderveen, Sherry; Schmidt, Louis A.; Beyene, Joseph; McDonald, Sarah D. | Binge Eating Predicts Excess Gestational Weight Gain: A Pilot Prospective Cohort Study | Irrelevant results |
| 113 | 2004 | Warren, Jennifer B; Silver, Robert M | Autoimmune disease in pregnancy: systemic lupus erythematosus and antiphospholipid syndrome | Irrelevant results |
| 114 | 2021 | Arditi, Brittany; Wen, Timothy; Riley, Laura E.; D'Alton, Mary; Sobhani, Nasim C.; Friedman, Alexander M.; Venkatesh, Kartik K. | Associations of influenza, chronic comorbid conditions, and severe maternal morbidity among pregnant women in the United States with influenza at delivery hospitalization, 2000–2015 | Excluded for out of context |
| 115 | 2018 | Fahmy, Walid Makin; Crispim, Cibele Aparecida; Cliffe, Susan | Association between maternal death and cesarean section in Latin America: A systematic literature review | Irrelevant results |
| 116 | 2021 | Clapp, Mark A.; James, Kaitlyn E.; Little, Sarah E.; Robinson, Julian N.; Kaimal, Anjali J. | Association between hospital-level cesarean delivery rates and severe maternal morbidity and unexpected newborn complications | Excluded for out of context |
| 117 | 2019 | Blanc, Julie; Resseguier, Noémie; Goffinet, François; Lorthe, Elsa; Kayem, Gilles; Delorme, Pierre; Vayssière, Christophe; Auquier, Pascal; D’Ercole, Claude | Association between gestational age and severe maternal morbidity and mortality of preterm cesarean delivery: a population-based cohort study | Irrelevant results |
| 118 | 2021 | Hastie, Roxanne; Tong, Stephen; Wikström, Anna-Karin; Sandström, Anna; Hesselman, Susanne; Bergman, Lina | Aspirin use during pregnancy and the risk of bleeding complications: a Swedish population-based cohort study | Irrelevant results |
| 119 | 2021 | Butwick, A. J.; McDonnell, N. | Antepartum and postpartum anemia: a narrative review | Irrelevant results |
| 120 | 2018 | Rana, Sarosh; Salahuddin, Saira; Mueller, Ariel; Berg, Anders H.; Thadhani, Ravi I.; Karumanchi, S. Ananth | Angiogenic biomarkers in triage and risk for preeclampsia with severe features | Irrelevant results |
| 121 | 2020 | Villalain, Cecilia; Herraiz, Ignacio; Cantero, Borja; Quezada, Soledad; Lopez, Ana; Simón, Elisa; Galindo, Alberto | Angiogenesis biomarkers for the prediction of severe adverse outcomes in late-preterm preeclampsia | Irrelevant results |
| 122 | 2018 | Luitjes, Susanne H. E.; Hermens, Rosella P. M. G.; de Wit, Linda; Heymans, Martijn W.; van Tulder, Maurits W.; Wouters, Maurice G. A. J. | An innovative implementation strategy to improve the use of Dutch guidelines on hypertensive disorders in pregnancy: A randomized controlled trial | Irrelevant results |
| 123 | 2017 | Luke, Barbara; Gopal, Daksha; Cabral, Howard; Stern, Judy E.; Diop, Hafsatou | Adverse pregnancy, birth, and infant outcomes in twins: effects of maternal fertility status and infant gender combinations; the Massachusetts Outcomes Study of Assisted Reproductive Technology | Irrelevant results |
| 124 | 2016 | Rehkopf, David H.; Headen, Irene; Hubbard, Alan; Deardorff, Julianna; Kesavan, Yamini; Cohen, Alison K.; Patil, Divya; Ritchie, Lorrene D.; Abrams, Barbara | Adverse childhood experiences and later life adult obesity and smoking in the United States | Irrelevant results |
| 125 | 2012 | Sagot, P.; Mourtialon, P.; Benzenine, E.; Bardou, M.; Ferdynus, C.; Morel, P.; Quantin, C. | Accuracy of blood transfusion in postpartum hemorrhage to assess maternal morbidity | Irrelevant results |
| 127 | 2020 | Deleu, F.; Deneux-Tharaux, C.; Chiesa-Dubruille, C.; Seco, A.; Bonnet, M. P. | A population-based analysis of French transfusion practices for women experiencing severe postpartum hemorrhage | Irrelevant results |
| 128 | 2020 | Porcelli, Bree A.; Diveley, Emily; Meyenburg, Kaytelyn; Woolfolk, Candice; Rosenbloom, Joshua I.; Raghuraman, Nandini; Stout, Molly J.; Sabol, Bethany A. | A new definition of gestational hypertension? New-onset blood pressures of 130 to 139/80 to 89 mm Hg after 20 weeks of gestation | Irrelevant results |
| 129 | 2010 | D’Ambruoso, Lucia; Byass, Peter; Qomariyah, Siti Nurul; Ouédraogo, Moctar | A lost cause? Extending verbal autopsy to investigate biomedical and socio-cultural causes of maternal death in Burkina Faso and Indonesia | Irrelevant results |
| 130 | 2011 | Fawole, Adeniran O.; Sotiloye, Oladapo S.; Hunyinbo, Kehinde I.; Umezulike, Augustine C.; Okunlola, Michael A.; Adekanle, Daniel A.; and al | A double-blind, randomized, placebo-controlled trial of misoprostol and routine uterotonics for the prevention of postpartum hemorrhage | Irrelevant results |
| 131 | 2022 | Phibbs, Claire M.; Kozhimannil, Katy B.; Leonard, Stephanie A.; Lorch, Scott A.; Main, Elliott K.; Schmitt, Susan K.; Phibbs, Ciaran S. | A Comprehensive Analysis of the Costs of Severe Maternal Morbidity | Irrelevant results |
| 132 | 2019 | Easter, Sarah Rae; Bateman, Brian T.; Sweeney, Valerie Horton; Manganaro, Karen; Lassey, Sarah C.; Gagne, Joshua J.; Robinson, Julian N. | A comorbidity-based screening tool to predict severe maternal morbidity at the time of delivery | Irrelevant results |
| 133 | 2016 | Silveira, Carla; Parpinelli, Mary A.; Pacagnella, Rodolfo C.; Andreucci, Carla B.; Ferreira, Elton C.; Angelini, Carina R.; and al | A cohort study of functioning and disability among women after severe maternal morbidity | Excluded for out of context |
| 134 | 2016 | Olsen, Michael H; Angell, Sonia Y; Asma, Samira; Boutouyrie, Pierre; Burger, Dylan; Chirinos, Julio A; and al | A call to action and a lifecourse strategy to address the global burden of raised blood pressure on current and future generations: the Lancet Commission on hypertension | Irrelevant results |
| 135 | 2014 | Cleary, Brian J; Rice, Úna; Eogan, Maeve; Metwally, Nehad; McAuliffe, Fionnuala | 2009 A/H1N1 influenza vaccination in pregnancy: uptake and pregnancy outcomes – a historical cohort study | Irrelevant results |
| 136 | 2020 | Wang, Eileen; Glazer, Kimberly B.; Howell, Elizabeth A.; Janevic, Teresa M. | Social Determinants of Pregnancy-Related Mortality and Morbidity in the United States: A Systematic Review | Irrelevant results |
| 137 | 2018 | Howell, Elizabeth A. | Reducing Disparities in Severe Maternal Morbidity and Mortality | Irrelevant results |
| 138 | 2012 | Tunçalp, Ozge; Hindin, Michelle J.; Adu-Bonsaffoh, Kwame; Adanu, Richard | Listening to women's voices: the quality of care of women experiencing severe maternal morbidity, in Accra, Ghana | Irrelevant results |
| 139 | 2016 | Alkema, Leontine; Chou, Doris; Hogan, Daniel; Zhang, Sanqian; Moller, Ann-Beth; Gemmill, Alison; Fat, Doris Ma; Boerma, Ties; Temmerman, Marleen; Mathers, Colin; Say, Lale | Global, regional, and national levels and trends in maternal mortality between 1990 and 2015, with scenario-based projections to 2030: a systematic analysis by the UN Maternal Mortality Estimation Inter-Agency Group | Irrelevant results |
| 140 | 1998 | Mantel, G. D.; Buchmann, E.; Rees, H.; Pattinson, R. C. | Severe acute maternal morbidity: a pilot study of a definition for a near-miss | Irrelevant results |
| 141 | 2017 | Völker, Fabian; Cooper, Paul; Bader, Oliver; Uy, Angela; Zimmermann, Ortrud; Lugert, Raimond; Groß, Uwe | Prevalence of pregnancy-relevant infections in a rural setting of Ghana | Irrelevant results |
| 142 | 2018 | Liyew, Ewnetu Firdawek; Yalew, Alemayehu Worku; Afework, Mesganaw Fantahun; Essén, Birgitta | Maternal near-miss and the risk of adverse perinatal outcomes: a prospective cohort study in selected public hospitals of Addis Ababa, Ethiopia | Irrelevant results |
| 143 | 2020 | Geleto, Ayele; Chojenta, Catherine; Taddele, Tefera; Loxton, Deborah | Incidence of maternal near miss among women in labour admitted to hospitals in Ethiopia | Irrelevant results |
| 144 | 2004 | Bates, Imelda; Fenton, Caroline; Gruber, Janet; Lalloo, David; Lara, Antonieta Medina; Squire, S Bertel; Theobald, Sally; Thomson, Rachael; Tolhurst, Rachel | Vulnerability to malaria, tuberculosis, and HIV/AIDS infection and disease. Part 1: determinants operating at individual and household level | Irrelevant results |
| 145 | 2019 | Houwing, M. E.; de Pagter, P. J.; van Beers, E. J.; Biemond, B. J.; Rettenbacher, E.; Rijneveld, A. W.; Schols, E. M.; Philipsen, J. N. J.; Tamminga, R. Y. J.; van Draat, K. Fijn; Nur, E.; Cnossen, M. H. | Sickle cell disease: Clinical presentation and management of a global health challenge | Irrelevant results |
| 146 | 2020 | Kidane, Daniel; Woldemichael, Andinet | Does inflation kill? Exposure to food inflation and child mortality | Irrelevant results |
| 147 | 2010 | Xu, Hairong; Perez-Cuevas, Ricardo; Xiong, Xu; Reyes, Hortensia; Roy, Chantal; Julien, Pierre; Smith, Graeme and al | An international trial of antioxidants in the prevention of preeclampsia (INTAPP) | Irrelevant results |
| 148 | 2007 | Tielsch, James M; Khatry, Subarna K; Stoltzfus, Rebecca J; Katz, Joanne; LeClerq, Steven C; Adhikari, Ramesh; Mullany, Luke C; Black, Robert; Shresta, Shardaram | Effect of daily zinc supplementation on child mortality in southern Nepal: a community-based, cluster randomised, placebo-controlled trial | Irrelevant results |
| 149 | 2000 |  | Effect of breastfeeding on infant and child mortality due to infectious diseases in less developed countries: a pooled analysis | Irrelevant results |
| 150 | 2018 | Nyakang’o, Sarange B.; Booth, Andrew | Women's perceived barriers to giving birth in health facilities in rural Kenya: A qualitative evidence synthesis | Irrelevant results |
| 151 | 2018 | Cruz, Meredith O.; Briller, Joan; Hibbard, Judith U. | New Insights in Peripartum Cardiomyopathy | Irrelevant results |
| 152 | 2020 | Panda, Basant Kumar; Kumar, Gulshan; Mishra, Suyash | Understanding the full-immunization gap in districts of India: A geospatial approach | Irrelevant results |
| 153 | 2013 | Wise, Lauren A.; Palmer, Julie R.; Rosenberg, Lynn | Lifetime abuse victimization and risk of uterine leiomyomata in black women | Irrelevant results |
| 154 | 2000 | von Mutius, Erika | The environmental predictors of allergic disease | Irrelevant results |
| 155 | 2007 | Omondi, Dickens; Ogol, Calistus; Otieno, Syprine; Macharia, Isaac | Parental awareness of hearing impairment in their school-going children and healthcare seeking behaviour in Kisumu district, Kenya | Irrelevant results |
| 156 | 2021 | Mahalingaiah, Shruthi; Fruh, Victoria; Rodriguez, Erika; Konanki, Sai Charan; Jukka-Pekka, Onnela; De Figueiredo Veiga, Alexis and al | Design and Methods of the Apple Women’s Health Study: A Digital Longitudinal Cohort Study | Irrelevant results |
| 157 | 2020 | Kumar, Kaushalendra; Singh, Abhishek; James, K. S.; McDougal, Lotus; Raj, Anita | Gender bias in hospitalization financing from borrowings, selling of assets, contribution from relatives or friends in India | Irrelevant results |
| 158 | 2006 | Kulkarni, Anjali | Very Low Birth Weight Baby: Review of Outcome and Personal Experience | Irrelevant results |
| 159 | 2019 | Ahmed, Rukhsana; Poespoprodjo, Jeanne R; Syafruddin, Din; Khairallah, Carole; Pace, Cheryl; Lukito, Theda; and al | Efficacy and safety of intermittent preventive treatment and intermittent screening and treatment versus single screening and treatment with dihydroartemisinin–piperaquine for the control of malaria in pregnancy in Indonesia: a cluster-randomised, open-label, superiority trial | Irrelevant results |
| 160 | 2020 | Sarkar, Amitabha; Liu, Guangqi; Jin, Yinzi; Xie, Zheng; Zheng, Zhi-Jie | Public health preparedness and responses to the coronavirus disease 2019 (COVID-19) pandemic in South Asia: a situation and policy analysis | Irrelevant results |
| 161 | 2003 | Jarvis, Nicole T | Rheumatic fever | Irrelevant results |
| 162 | 2013 | Pasricha, Sant-Rayn; Drakesmith, Hal; Black, James; Hipgrave, David; Biggs, Beverley-Ann | Control of iron deficiency anemia in low- and middle-income countries | Irrelevant results |
| 163 | 2019 | Blanco, Carlos; Volkow, Nora D | Management of opioid use disorder in the USA: present status and future directions | Irrelevant results |
| 165 | 2012 | Carter-Pokras, Olivia D.; Offutt-Powell, Tabatha N.; Kaufman, Jay S.; Giles, Wayne H.; Mays, Vickie M. | Epidemiology, Policy, and Racial/Ethnic Minority Health Disparities | Irrelevant results |
| 166 | 2002 | Goldenberg, Robert L | The management of preterm labor | Irrelevant results |
| 167 | 2010 | Patel, Uptal D. | Fetal Origins of Renal Disparities | Irrelevant results |
| 168 | 2016 | Bourtzis, Kostas; Lees, Rosemary Susan; Hendrichs, Jorge; Vreysen, Marc J. B. | More than one rabbit out of the hat: Radiation, transgenic and symbiont-based approaches for sustainable management of mosquito and tsetse fly populations | Irrelevant results |
| 169 | 2012 | Pasricha, Sant-Rayn | Should we screen for iron deficiency anaemia? A review of the evidence and recent recommendations | Irrelevant results |
| 170 | 2019 | Kassa, Desta; Gebremichael, Gebremedhin; Tilahun, Tesfaye; Ayalkebet, Abenezer; Abrha, Yemane; Mesfin, Getnet; and al | Prevalence of sexually transmitted infections (HIV, hepatitis B virus, herpes simplex virus type 2, and syphilis) in pregnant women in Ethiopia: Trends over 10 years (2005–2014) | Irrelevant results |
| 171 | 2003 | Anachebe, Ngozi F.; Sutton, Madeline Y. | Racial disparities in reproductive health outcomes | Irrelevant results |
| 172 | 2020 | Quiroz, Hallie J.; Rao, Krishnamurti; Brady, Ann-Christina; Hogan, Anthony R.; Thorson, Chad M.; Perez, Eduardo A.; Neville, Holly L.; Sola, Juan E. | Protocol-Driven Surgical Care of Necrotizing Enterocolitis and Spontaneous Intestinal Perforation | Irrelevant results |
| 173 | 2019 | Chandrashekar, Valleesha N.; Punnath, Kishore; Dayanand, Kiran K.; Achur, Rajeshwara N.; Kakkilaya, Srinivas B.; Jayadev, Poornima; Kumari, Suchetha N.; Gowda, D. Channe | Malarial anemia among pregnant women in the south-western coastal city of Mangaluru in India | Irrelevant results |
| 174 | 2021 | Gulcebi, Medine I.; Bartolini, Emanuele; Lee, Omay; Lisgaras, Christos Panagiotis; Onat, Filiz; Mifsud, Janet; Striano, Pasquale and al | Climate change and epilepsy: Insights from clinical and basic science studies | Irrelevant results |
| 175 | 2004 | Adler, Stuart P.; Finney, Jack W.; Manganello, Anne Marie; Best, Al M. | Prevention of child-to-mother transmission of cytomegalovirus among pregnant women | Irrelevant results |
| 176 | 2016 | Sharma, Jai B.; Bumma, Sirisha D.; Saxena, Renu; Kumar, Sunesh; Roy, Kallol K.; Singh, Neeta; Vanamail, P. | Cross sectional, comparative study of serum erythropoietin, transferrin receptor, ferritin levels and other hematological indices in normal pregnancies and iron deficiency anemia during pregnancy | Irrelevant results |
| 177 | 2008 | Wiseman, Virginia; Scott, Anthony; Conteh, Lesong; McElroy, Brendan; Stevens, Warren | Determinants of provider choice for malaria treatment: Experiences from The Gambia | Irrelevant results |
| 178 | 2016 | Boone, Peter; Elbourne, Diana; Fazzio, Ila; Fernandes, Samory; Frost, Chris; Jayanty, Chitra; and al | Effects of community health interventions on under-5 mortality in rural Guinea-Bissau (EPICS): a cluster-randomised controlled trial | Irrelevant results |
| 179 | 2018 | Grayson, Mitchell H.; Feldman, Scott; Prince, Benjamin T.; Patel, Priya J.; Matsui, Elizabeth C.; Apter, Andrea J. | Advances in asthma in 2017: Mechanisms, biologics, and genetics | Irrelevant results |
| 180 | 2009 | Craig, Sienna R.; Adams, Lisa V.; Spielberg, Stephen P.; Campbell, Benjamin | Pediatric therapeutics and medicine administration in resource-poor settings: A review of barriers and an agenda for interdisciplinary approaches to improving outcomes | Irrelevant results |
| 181 | 2018 | Zachara, Bronislaw A. | Chapter Five - Selenium in Complicated Pregnancy. A Review | Irrelevant results |
| 182 | 2020 | Lapolla, Annunziata; Amaro, Flavia; Bruttomesso, Daniela; Di Bartolo, Paolo; Grassi, Giorgio; Maffeis, Claudio; Purrello, Francesco; Tumini, Stefano | Diabetic ketoacidosis: A consensus statement of the Italian Association of Medical Diabetologists (AMD), Italian Society of Diabetology (SID), Italian Society of Endocrinology and Pediatric Diabetoloy (SIEDP) | Irrelevant results |
| 183 | 2006 | Grimes, David A; Benson, Janie; Singh, Susheela; Romero, Mariana; Ganatra, Bela; Okonofua, Friday E; Shah, Iqbal H | Unsafe abortion: the preventable pandemic | Irrelevant results |
| 184 | 2005 | Max, Jeffrey E; Levin, Harvey S; Landis, Julie; Schachar, Russell; Saunders, Ann; Ewing-Cobbs, Linda; Chapman, Sandra B; Dennis, Maureen | Predictors of Personality Change Due to Traumatic Brain Injury in Children and Adolescents in the First Six Months After Injury | Irrelevant results |
| 185 | 2014 | Atari, Dominic Odwa; Mkandawire, Paul | Spatial variation of management of childhood diarrhea in Malawi | Irrelevant results |
| 186 | 2009 | Schempf, Ashley; Strobino, Donna; O'Campo, Patricia | Neighborhood effects on birthweight: An exploration of psychosocial and behavioral pathways in Baltimore, 1995–1996 | Irrelevant results |
| 187 | 2017 | Oppenheimer, John J.; Marshall, Gailen D. | Increasing our knowledge base of asthma | Irrelevant results |
| 188 | 2019 | Deribe, Kebede; Mbituyumuremyi, Aimable; Cano, Jorge; Jean Bosco, Mbonigaba; Giorgi, Emanuele; Ruberanziza, Eugene; and al | Geographical distribution and prevalence of podoconiosis in Rwanda: a cross-sectional country-wide survey | Irrelevant results |
| 189 | 2018 | Bellin, Melissa H.; Newsome, Angelica; Lewis-Land, Cassie; Kub, Joan; Mudd, Shawna S.; Margolis, Rachel; Butz, Arlene M. | Improving Care of Inner-City Children with Poorly Controlled Asthma: What Mothers Want You to Know | Irrelevant results |
| 190 | 2015 | Jones, Kelly M.; de Brauw, Alan | Using Agriculture to Improve Child Health: Promoting Orange Sweet Potatoes Reduces Diarrhea | Irrelevant results |
| 191 | 2004 | Fowles, Eileen R. | Prenatal Nutrition and Birth Outcomes | Irrelevant results |
| 192 | 2018 | Lehna, Carlee; Furmanek, Stephen; Fahey, Erin; Hanchette, Carol | Geographic modeling for children at risk for home fires and burns | Irrelevant results |
| 193 | 2011 | Johnson, Lois; Bhutani, Vinod K. | The Clinical Syndrome of Bilirubin-Induced Neurologic Dysfunction | Irrelevant results |
| 194 | 2011 | Hall, Nicole Ruddock | What Agent Should be Used to Prevent Recurrent Preterm Birth: 17-P or Natural Progesterone? | Irrelevant results |
| 195 | 2010 | Cruz, Meredith O.; Briller, Joan; Hibbard, Judith U. | Update on Peripartum Cardiomyopathy | Irrelevant results |
| 196 | 2017 | Moss, William J | Measles | Irrelevant results |
| 197 | 2007 | Gunter, Jennifer | Intimate Partner Violence | Irrelevant results |
| 198 | 2014 | Burlando, Alfredo | Transitory shocks and birth weights: Evidence from a blackout in Zanzibar | Irrelevant results |
| 199 | 2021 | Alcindor, Magalie L.; Alcindor, FitzGerald; Richard, Kristy E.; Ajay, Geetha; Denis, Anne Marie; Dickson, Darlene M.; Lawal, Ekaete; Alcindor, Magaline A.; Allen, Deborah | COVID-19 Management in Pediatrics | Irrelevant results |
| 200 | 2012 | Moroni, Luca; Bianchi, Ilaria; Lleo, Ana | Geoepidemiology, gender and autoimmune disease | Irrelevant results |
| 201 | 2016 | Ariff, Shabina; Lee, ANNE CC; Lawn, Joy; Bhutta, Zulfiqar A. | Global Burden, Epidemiologic Trends, and Prevention of Intrapartum-Related Deaths in Low-Resource Settings | Irrelevant results |
| 202 | 2021 | Cantor, David James; Swartz, Jina; Bayard, Roberts; Abbara, Aula; Ager, Alastair; Bhutta, Zulfiqar A. and al | Understanding the health needs of internally displaced persons: a scoping review | Irrelevant results |
| 203 | 2021 | Gonzalez-Rivas, Juan P.; Mechanick, Jeffrey I.; Infante-Garcia, Maria M.; Medina-Inojosa, Jose R.; Pavlovska, Iuliia; Hlinomaz, Ota; Zak, Petr; Kunzova, Sarka; and al | The Prevalence of Dysglycemia-Based Chronic Disease in a European Population – a New Paradigm to Address Diabetes Burden: A Kardiovize Study | Irrelevant results |
| 204 | 2015 | Records, Barbara L. Wilson; Dyer, Jane M.; Latendresse, Gwen; Wong, Bob; Baksh, Laurie | Exploring the Psychosocial Predictors of Gestational Diabetes and Birth Weight | Irrelevant results |
| 205 | 2017 | Dugani, Sagar; Kissoon, Niranjan | Global advocacy needed for sepsis in children | Irrelevant results |
| 206 | 2017 | Pal, Amitava; Pari, Amal Kumar; Sinha, Arunangshu; Dhara, Prakash C. | Prevalence of undernutrition and associated factors: A cross-sectional study among rural adolescents in West Bengal, India | Irrelevant results |
| 207 | 2019 | Avorgbedor, Forgive; Silva, Susan; Merwin, Elizabeth; Blumenthal, James A.; Holditch-Davis, Diane | Health, Physical Growth, and Neurodevelopmental Outcomes in Preterm Infants of Women With Hypertensive Disorders of Pregnancy | Irrelevant results |
| 208 | 2014 | Hu, Hui; Ha, Sandie; Roth, Jeffrey; Kearney, Greg; Talbott, Evelyn O.; Xu, Xiaohui | Ambient air pollution and hypertensive disorders of pregnancy: A systematic review and meta-analysis | Irrelevant results |
| 209 | 2009 | Gadalla, Tahany M. | Determinants, correlates and mediators of psychological distress: A longitudinal study | Irrelevant results |
| 210 | 2016 | Nimi, Tazi; Fraga, Sílvia; Costa, Diogo; Campos, Paulo; Barros, Henrique | Prenatal care and pregnancy outcomes: A cross-sectional study in Luanda, Angola | Irrelevant results |
| 211 | 2005 | Wadhwa, Pathik D. | Psychoneuroendocrine processes in human pregnancy influence fetal development and health | Irrelevant results |
| 212 | 2021 | Kim, Faith; Bernbaum, Judy; Connelly, James; Gerdes, Marsha; Hedrick, Holly L.; Hoffman, Casey; Rintoul, Natalie E.; Ziolkowski, Kristina; DeMauro, Sara B. | Survival and Developmental Outcomes of Neonates Treated with Extracorporeal Membrane Oxygenation: A 10-Year Single-Center Experience | Irrelevant results |
| 213 | 2006 | Ornoy, Asher; Diav-Citrin, Orna | Fetal effects of primary and secondary cytomegalovirus infection in pregnancy | Irrelevant results |
| 214 | 2015 | Fagerberg, Marie C.; Maršál, Karel; Källén, Karin | Predicting the chance of vaginal delivery after one cesarean section: validation and elaboration of a published prediction model | Irrelevant results |
| 215 | 2010 | Heath, Paul T.; Okike, Ifeanyichukwu O. | Neonatal bacterial meningitis: an update | Irrelevant results |
| 216 | 2018 | Turner, Paul J.; Campbell, Dianne E.; Boyle, Robert J.; Levin, Michael E. | Primary Prevention of Food Allergy: Translating Evidence from Clinical Trials to Population-Based Recommendations | Irrelevant results |
| 217 | 2021 | Ariyo, Oluwaseun; Aderibigbe, Olaide R; Ojo, Tomilola J.; Sturm, Barbara; Hensel, Oliver | Determinants of appropriate complementary feeding practices among women with children aged 6-23 months in Iseyin, Nigeria | Irrelevant results |
| 218 | 2018 | Ahsan, Md Nazmul; Maharaj, Riddhi | Parental human capital and child health at birth in India | Irrelevant results |
| 219 | 2003 | Kestenbaum, Bryan; Seliger, Stephen L.; Easterling, Thomas R.; Gillen, Daniel L.; Critchlow, Cathy W.; Stehman-Breen, Catherine O.; Schwartz, Stephen M. | Cardiovascular and thromboembolic events following hypertensive pregnancy | Irrelevant results |
| 220 | 2016 | Muzik, Maria; Brier, Zoe; Menke, Rena A.; Davis, Margaret T.; Sexton, Minden B. | Longitudinal suicidal ideation across 18-months postpartum in mothers with childhood maltreatment histories | Irrelevant results |
| 221 | 2013 | Tedeschi, Sara K.; Bermas, Bonnie; Costenbader, Karen H. | Sexual disparities in the incidence and course of SLE and RA | Irrelevant results |
| 222 | 2017 | Denno, Donna M.; Paul, Shadae L. | Child Health and Survival in a Changing World | Irrelevant results |
| 223 | 2014 | Karkee, Rajendra; Lee, Andy H.; Khanal, Vishnu; Pokharel, Paras K.; Binns, Colin W. | Obstetric complications and cesarean delivery in Nepal | Irrelevant results |
| 224 | 2012 | Horton, Amanda L.; Boggess, Kim A. | Periodontal Disease and Preterm Birth | Irrelevant results |
| 225 | 2000 | Zelenko, Marina; Lock, James; Kraemer, Helena C.; Steiner, Hans | Perinatal complications and child abuse in a poverty sample | Irrelevant results |
| 226 | 2017 | Joshi, Rohina; John, Oommen; Jha, Vivekanand | The Potential Impact of Public Health Interventions in Preventing Kidney Disease | Irrelevant results |
| 227 | 2020 | Reddy, Ché L.; Patterson, Rolvix H.; Wasserman, Isaac; Meara, John G.; Afshar, Salim | Oral and Maxillofacial Surgery: An Opportunity to Improve Surgical Care and Advance Sustainable Development Globally | Irrelevant results |
| 228 | 2005 | Halbreich, Uriel | The association between pregnancy processes, preterm delivery, low birth weight, and postpartum depressions—The need for interdisciplinary integration | Irrelevant results |
| 229 | 2019 | Chaemsaithong, Piya; Pooh, Ritsuko K.; Zheng, Mingming; Ma, Runmei; Chaiyasit, Noppadol; Tokunaka, Mayumi; and al | Prospective evaluation of screening performance of first-trimester prediction models for preterm preeclampsia in an Asian population | Irrelevant results |
| 230 | 2021 | Reilly, Nicole; Hadzi-Pavlovic, Dusan; Loxton, Deborah; Black, Emma; Mule, Victoria; Austin, Marie-Paule | Supporting routine psychosocial assessment in the perinatal period: The concurrent and predictive validity of the Antenatal Risk Questionnaire-Revised | Irrelevant results |
| 231 | 2000 | Richards, Lynn A.; Klemm, Paula | An Inpatient Cervical Cancer Screening Program to Reach Underserved Women | Irrelevant results |
| 232 | 2016 | Allen, Alina M.; Kim, W. Ray; Larson, Joseph J.; Rosedahl, Jordan K.; Yawn, Barbara P.; McKeon, Kimberly; Hay, J. Eileen | The Epidemiology of Liver Diseases Unique to Pregnancy in a US Community: A Population-Based Study | Irrelevant results |
| 233 | 2017 | Murphy, M. O.; Cohn, D. M.; Loria, A. S. | Developmental origins of cardiovascular disease: Impact of early life stress in humans and rodents | Irrelevant results |
| 234 | 2011 | Piteo, A. M.; Lushington, K.; Roberts, R. M.; van den Heuvel, C. J.; Nettelbeck, T.; Kohler, M. J.; Martin, A. J.; Kennedy, J. D. | Prevalence of snoring and associated factors in infancy | Irrelevant results |
| 235 | 2015 | Wise, Lauren A.; Li, Se; Palmer, Julie R.; Rosenberg, Lynn | Depressive symptoms and risk of uterine leiomyomata | Irrelevant results |
| 236 | 2018 | Yunus, Fakir Md.; Khan, Safayet; Mitra, Dipak K.; Mistry, Sabuj Kanti; Afsana, Kaosar; Rahman, Mahfuzar | Relationship of sleep pattern and snoring with chronic disease: findings from a nationwide population-based survey | Irrelevant results |
| 237 | 2015 | Liberato, Selma C.; Singh, Gurmeet; Mulholland, Kim | Zinc supplementation in young children: A review of the literature focusing on diarrhoea prevention and treatment | Irrelevant results |
| 238 | 2012 | Zöller, Bengt; Li, Xinjun; Sundquist, Jan; Sundquist, Kristina | Risk of venous thromboembolism in first- and second-generation immigrants in Sweden | Irrelevant results |
| 239 | 2014 | Khilanani, Ajay; Mazwi, Mjaye; Paquette, Erin Talati | Pediatric Sepsis in the Global Setting | Irrelevant results |
| 240 | 2017 | Mondi, Christina F.; Reynolds, Arthur J.; Ou, Suh-Ruu | Predictors of depressive symptoms in emerging adulthood in a low-income urban cohort | Irrelevant results |
| 241 | 2009 | Holland, Marium G.; Refuerzo, Jerrie S.; Ramin, Susan M.; Saade, George R.; Blackwell, Sean C. | Late preterm birth: how often is it avoidable? | Irrelevant results |
| 242 | 2013 | Chavkin, Wendy; Leitman, Liddy; Polin, Kate | Conscientious objection and refusal to provide reproductive healthcare: A White Paper examining prevalence, health consequences, and policy responses | Irrelevant results |
| 243 | 2005 | Schnell-Anzola, Beatrice; Rowe, Meredith L.; LeVine, Robert A. | Literacy as a pathway between schooling and health-related communication skills: a study of Venezuelan mothers | Irrelevant results |
| 244 | 2006 | May, John P. | Chapter 24 - Preventive Health Issues for Individuals in Jails and Prisons | Irrelevant results |
| 245 | 2009 | Weiner, Carl P.; Buhimschi, Catalin | C | Irrelevant results |
| 246 | 2018 | Leibson, Tom; Lala, Prateek; Ito, Shinya | Chapter 24 - Drug and Chemical Contaminants in Breast Milk: Effects on Neurodevelopment of the Nursing Infant | Irrelevant results |
| 247 | 2008 | Liu, Andrew H.; Covar, Ronina A. | Chapter 58 - Clinical Features, Outcomes, and Prognosis | Irrelevant results |
| 248 | 2006 | Dohil, Ranjan; Hassall, Eric | Chapter 25 - Gastritis, gastropathy and ulcer disease | Irrelevant results |
| 249 | 2007 | Sergueef, Nicette | Chapter 7 - Clinical Conditions | Irrelevant results |
| 250 | 2009 |  | Chapter 5 - Other Infectious Diseases Related to Travel | Irrelevant results |
| 251 | 2014 | Tulchinsky, Theodore H.; Varavikova, Elena A. | Chapter 3 - Measuring, Monitoring, and Evaluating the Health of a Population | Irrelevant results |
| 252 | 2011 | McKenzie, Steven E. | CHAPTER 9 - Hematology | Irrelevant results |
| 253 | 2008 |  | CHAPTER 9 - Commonly Encountered Medical Problems in Pregnancy | Irrelevant results |
| 254 | 2005 | Luderer, Ulrike; Cullen, Mark R; Luderer, Ulrike; Cullen, Mark R; Mattison, Donald R | Chapter 27 - Endocrine and Reproductive Disorders | Irrelevant results |
| 255 | 2014 | Guilbert, Theresa W.; Lemanske, Robert F.; Jackson, Daniel J. | 52 - Diagnosis of Asthma in Infants and Children | Irrelevant results |
| 256 | 2021 | Sushma, Rajbanshi; Norhayati, Mohd Noor; Nik Hazlina, Nik Hussain | Prevalence of neonatal near miss and associated factors in Nepal: a cross-sectional study | Irrelevant results |
| 257 | 2006 | Okong, Pius; Byamugisha, Josaphat; Mirembe, Florence; Byaruhanga, Romano; Bergstrom, Staffan | Audit of severe maternal morbidity in Uganda – implications for quality of obstetric care | Irrelevant results |
| 258 | 2017 | Moll, Ulrika; Olsson, Håkan; Landin-Olsson, Mona | Impact of Pregestational Weight and Weight Gain during Pregnancy on Long-Term Risk for Diseases | Irrelevant results |
| 259 | 2017 | Sharma, Jigyasa; Leslie, Hannah H.; Kundu, Francis; Kruk, Margaret E. | Poor Quality for Poor Women? Inequities in the Quality of Antenatal and Delivery Care in Kenya | Irrelevant results |
| 260 | 2019 | Dingemann, Carmen; Sonne, Martin; Ure, Benno; Bohnhorst, Bettina; Kaisenberg, Constantin von; Pirr, Sabine | Impact of maternal education on the outcome of newborns requiring surgery for congenital malformations | Irrelevant results |
| 261 | 2015 | Delamou, Alexandre; Dubourg, Dominique; Beavogui, Abdoul Habib; Delvaux, Thérèse; Kolié, Jacques Seraphin; Barry, Thierno Hamidou; and al | How Has the Free Obstetric Care Policy Impacted Unmet Obstetric Need in a Rural Health District in Guinea? | Irrelevant results |
| 262 | 2017 | Bellad, Mrutynjaya B.; Vidler, Marianne; Honnungar, Narayan V.; Mallapur, Ashalata; Ramadurg, Umesh; Charanthimath, Umesh; and al | Maternal and Newborn Health in Karnataka State, India: The Community Level Interventions for Pre-Eclampsia (CLIP) Trial’s Baseline Study Results | Irrelevant results |
| 263 | 2012 | Koch, Elard; Thorp, John; Bravo, Miguel; Gatica, Sebastián; Romero, Camila X.; Aguilera, Hernán; Ahlers, Ivonne | Women's Education Level, Maternal Health Facilities, Abortion Legislation and Maternal Deaths: A Natural Experiment in Chile from 1957 to 2007 | Irrelevant results |
| 264 | 2016 | Phiri, Selia Ng’anjo; Fylkesnes, Knut; Moland, Karen Marie; Byskov, Jens; Kiserud, Torvid | Rural-Urban Inequity in Unmet Obstetric Needs and Functionality of Emergency Obstetric Care Services in a Zambian District | Irrelevant results |
| 265 | 2019 | Todd, Catherine S.; Chowdhury, Zakaria; Mahmud, Zeba; Islam, Nazia; Shabnam, Sadia; Parvin, Musarrat; Bernholc, Alissa; Martinez, Andres; Aktar, Bachera; Afsana, Kaosar; Sanghvi, Tina | Maternal nutrition intervention and maternal complications in 4 districts of Bangladesh: A nested cross-sectional study | Irrelevant results |
| 266 | 2020 | Okoroiwu, Henshaw Uchechi; Uchendu, Kingsley Ikenna; Essien, Rita A. | Causes of morbidity and mortality among patients admitted in a tertiary hospital in southern Nigeria: A 6 year evaluation | Irrelevant results |
| 267 | 2013 | Acosta, Colleen D.; Knight, Marian; Lee, Henry C.; Kurinczuk, Jennifer J.; Gould, Jeffrey B.; Lyndon, Audrey | The Continuum of Maternal Sepsis Severity: Incidence and Risk Factors in a Population-Based Cohort Study | Irrelevant results |
| 268 | 2021 | Tenaw, Shegaw Geze; Assefa, Nega; Mulatu, Teshale; Tura, Abera Kenay | Maternal near miss among women admitted in major private hospitals in eastern Ethiopia: a retrospective study | Irrelevant results |
| 269 | 2019 | Adanikin, A. I.; Umeora, O. U. J.; Nzeribe, E.; Agbata, A. T.; Ezeama, C.; Ezugwu, F. O.; Ugwu, G. O.; Ikechebelu, J. I.; Oladapo, O. T. | Maternal near-miss and death associated with abortive pregnancy outcome: a secondary analysis of the Nigeria Near-miss and Maternal Death Survey | Irrelevant results |
| 270 | 2019 | Chhabra, Pragti; Guleria, Kiran; Bhasin, Sanjiv Kumar; Kumari, Komal; Singh, Shalini; Lukhmana, Shveta | Severe maternal morbidity and maternal near miss in a tertiary hospital of Delhi | Irrelevant results |
| 271 | 2011 | Gavin, Amelia R.; Hill, Karl G.; Hawkins, J. David; Maas, Carl | The Role of Maternal Early-Life and Later-Life Risk Factors on Offspring Low Birth Weight: Findings From a Three-Generational Study | Irrelevant results |
| 272 | 2020 | Jairam, Jennifer A.; Vigod, Simone N.; O'Campo, Patricia; Park, Alison L.; Siddiqi, Arjumand; Ray, Joel G. | Neighbourhood Income and Risk of Having an Infant With Concomitant Preterm Birth and Severe Small for Gestational Age Birth Weight | Irrelevant results |
| 273 | 2020 | Khan, Md. Salauddin; Halder, Henry Ratul; Rashid, Mamunor; Afroja, Sohani; Islam, Masudul | Impact of socioeconomic and demographic factors for underweight and overweight children in Bangladesh: A polytomous logistic regression model | Irrelevant results |
| 274 | 2009 | Coovadia, Hoosen; Jewkes, Rachel; Barron, Peter; Sanders, David; McIntyre, Diane | The health and health system of South Africa: historical roots of current public health challenges | Irrelevant results |
| 275 | 2015 | Harper, Felicity W. K.; Eggly, Susan; Crider, Beverly; Kobayashi, Hitomi; Kathleen, R. N.; Meert, L.; Ball, Allison; Penner, Louis A.; Gray, Herman; Albrecht, Terrance L. | Patient- and Family-Centered Care as an approach to reducing disparities in asthma outcomes in urban African American children: A review of the literature | Irrelevant results |
| 276 | 2020 | Alemu, Birtukan; Gashu, Dawd | Association of maternal anthropometry, hemoglobin and serum zinc concentration during pregnancy with birth weight | Irrelevant results |
| 277 | 2014 | Bublitz, Margaret H.; Rodriguez, Daniel; Polly Gobin, Asi; Waldemore, Marissa; Magee, Susanna; Stroud, Laura R. | Maternal history of adoption or foster care placement in childhood: a risk factor for preterm birth | Irrelevant results |
| 278 | 2012 | Ramsay, Michèle | Africa: Continent of genome contrasts with implications for biomedical research and health | Irrelevant results |
| 279 | 2007 | Naqvi, Mariam; Choudhry, Shweta; Tsai, Hui-Ju; Thyne, Shannon; Navarro, Daniel; Nazario, Sylvette; and al | Association between IgE levels and asthma severity among African American, Mexican, and Puerto Rican patients with asthma | Irrelevant results |
| 280 | 2020 | Zhang, Ning; Tan, Jing; Yang, HaiFeng; Khalil, Raouf A. | Comparative risks and predictors of preeclamptic pregnancy in the Eastern, Western and developing world | Irrelevant results |
| 281 | 2021 | Keats, Emily C; Das, Jai K; Salam, Rehana A; Lassi, Zohra S; Imdad, Aamer; Black, Robert E; Bhutta, Zulfiqar A | Effective interventions to address maternal and child malnutrition: an update of the evidence | Irrelevant results |
| 282 | 2017 | Ligthart, Kelly A. M.; Buitendijk, Leanne; Koes, Bart W.; van Middelkoop, Marienke | The association between ethnicity, socioeconomic status and compliance to pediatric weight-management interventions – A systematic review | Irrelevant results |
| 283 | 2009 | Baum, Charles L.; Ruhm, Christopher J. | Age, socioeconomic status and obesity growth | Irrelevant results |
| 284 | 2016 | Premkumar, Ashish; Henry, Dana E.; Moghadassi, Michelle; Nakagawa, Sanae; Norton, Mary E. | The interaction between maternal race/ethnicity and chronic hypertension on preterm birth | Irrelevant results |
| 285 | 2006 | Cox, Anna M; McKevitt, Christopher; Rudd, Anthony G; Wolfe, Charles DA | Socioeconomic status and stroke | Irrelevant results |
| 286 | 2019 | McAllister, David A; Liu, Li; Shi, Ting; Chu, Yue; Reed, Craig; Burrows, John; Adeloye, Davies; Rudan, Igor; Black, Robert E; Campbell, Harry; Nair, Harish | Global, regional, and national estimates of pneumonia morbidity and mortality in children younger than 5 years between 2000 and 2015: a systematic analysis | Irrelevant results |
| 287 | 2008 | Lang, Christopher T.; King, Jeffrey C. | Maternal mortality in the United States | Irrelevant results |
| 288 | 2019 | Hyland, Marie; Russ, Jason | Water as destiny – The long-term impacts of drought in sub-Saharan Africa | Irrelevant results |
| 289 | 2005 | Parham, Groesbeck P.; Hicks, Michael L. | Racial Disparities Affecting the Reproductive Health of African-American Women | Irrelevant results |
| 290 | 2006 | Cohen, Robyn T.; Celedón, Juan C.; Hinckson, Vanessa J.; Ramsey, Clare D.; Wakefield, Dorothy B.; Weiss, Scott T.; Cloutier, Michelle M. | Health-Care Use Among Puerto Rican and African-American Children With Asthma | Irrelevant results |
| 291 | 2021 | Sole, Kristina Baker; Staff, Anne Cathrine; Laine, Katariina | Maternal diseases and risk of hypertensive disorders of pregnancy across gestational age groups | Irrelevant results |
| 292 | 2006 | Ford, Jean G.; McCaffrey, Lee | Understanding Disparities in Asthma Outcomes Among African Americans | Irrelevant results |
| 293 | 2002 | Ehrenberg, Hugh M.; Dierker, LeRoy; Milluzzi, Cynthia; Mercer, Brian M. | Prevalence of maternal obesity in an urban center | Irrelevant results |
| 294 | 2014 | Bustinduy, Amaya; King, Charles; Scott, Janet; Appleton, Sarah; Sousa-Figueiredo, José Carlos; Betson, Martha; Stothard, J Russell | HIV and schistosomiasis co-infection in African children | Irrelevant results |
| 295 | 2017 | Canfield, Martha; Radcliffe, Polly; Marlow, Sally; Boreham, Marggie; Gilchrist, Gail | Maternal substance use and child protection: a rapid evidence assessment of factors associated with loss of child care | Irrelevant results |
| 296 | 2020 | Millar, Jane R.; Bengu, Nomonde; Fillis, Rowena; Sprenger, Ken; Ntlantsana, Vuyokazi; Vieira, Vinicius A.; and al | HIGH-FREQUENCY failure of combination antiretroviral therapy in paediatric HIV infection is associated with unmet maternal needs causing maternal NON-ADHERENCE | Irrelevant results |
| 297 | 2017 | Dramowski, A.; Aucamp, M.; Bekker, A.; Mehtar, S. | Infectious disease exposures and outbreaks at a South African neonatal unit with review of neonatal outbreak epidemiology in Africa | Irrelevant results |
| 298 | 2013 | Lund, Troy C.; Hume, Heather; Allain, Jean P.; McCullough, Jeffrey; Dzik, Walter | The blood supply in Sub-Saharan Africa: Needs, challenges, and solutions | Irrelevant results |
| 299 | 2016 | Kibiribiri, Edith T.; Moodley, Dhayendre; Groves, Allison K.; Sebitloane, Motshedisi H. | Exploring disparities in prenatal care between refugees and local South African women | Irrelevant results |
| 300 | 2014 | Donald, Kirsten Ann; Hoare, Jackie; Eley, Brian; Wilmshurst, Jo M. | Neurologic Complications of Pediatric Human Immunodeficiency Virus: Implications for Clinical Practice and Management Challenges in the African Setting | Irrelevant results |
| 301 | 2014 | Mallewa, Macpherson; Wilmshurst, Jo M. | Overview of the Effect and Epidemiology of Parasitic Central Nervous System Infections in African Children | Irrelevant results |
| 302 | 2020 | Fatema, Kaniz; Lariscy, Joseph T. | Mass media exposure and maternal healthcare utilization in South Asia | Irrelevant results |
| 303 | 2018 | Li, Yang; Rosemberg, Marie-Anne Sanon; Seng, Julia S. | Allostatic load: A theoretical model for understanding the relationship between maternal posttraumatic stress disorder and adverse birth outcomes | Irrelevant results |
| 304 | 2017 | Slogrove, A. L.; Frigati, L.; Gray, D. M. | Maternal HIV and Paediatric Lung Health | Irrelevant results |
| 305 | 2018 | Adams, Katherine P.; Lybbert, Travis J.; Vosti, Stephen A.; Ayifah, Emmanuel; Arimond, Mary; Adu-Afarwuah, Seth; Dewey, Kathryn G. | Unintended effects of a targeted maternal and child nutrition intervention on household expenditures, labor income, and the nutritional status of non-targeted siblings in Ghana | Irrelevant results |
| 306 | 2013 | Griffiths, Paula L.; Johnson, William; Cameron, Noël; Pettifor, John M.; Norris, Shane A. | In urban South Africa, 16 year old adolescents experience greater health equality than children | Irrelevant results |
| 307 | 2013 | Wild, Katherine T.; Betancourt, Laura M.; Brodsky, Nancy L.; Hurt, Hallam | The effect of socioeconomic status on the language outcome of preterm infants at toddler age | Irrelevant results |
| 308 | 2004 | Burgard, Sarah | Race and pregnancy-related care in Brazil and South Africa | Irrelevant results |
| 309 | 2021 | Zhou, Yuan-yue; Zhang, Wen-wu; Chen, Fang; Hu, Sha-sha; Jiang, Hai-yin | Maternal infection exposure and the risk of psychosis in the offspring: A systematic review and meta-analysis | Irrelevant results |
| 310 | 2022 | Tan, Youran; Barr, Dana Boyd; Ryan, P. Barry; Fedirko, Veronika; Sarnat, Jeremy A.; Gaskins, Audrey J.; and al | High-resolution metabolomics of exposure to tobacco smoke during pregnancy and adverse birth outcomes in the Atlanta African American maternal-child cohort | Irrelevant results |
| 311 | 1999 | Prual, A. | [Pregnancy and delivery in western Africa. High risk motherhood] | Irrelevant results |
| 312 | 2000 | Ikamari, L. | Sibling mortality correlation in Kenya | Irrelevant results |
| 313 | 2014 | Mamabolo, Ramoteme L.; Alberts, Marianne | Prevalence of anaemia and its associated factors in African children at one and three years residing in the Capricorn District of Limpopo Province, South Africa | Irrelevant results |
| 314 | 2020 | Habyarimana, Faustin; Zewotir, Temesgen; Ramroop, Shaun | Prevalence and Risk Factors Associated with Anemia among Women of Childbearing Age in Rwanda | Irrelevant results |
| 315 | 2020 | Lisk, D. R.; Ngobeh, F.; Kumar, B.; Moses, F.; Russell, J. B. | Stroke in Sierra Leonean Africans:Perspectives from a Private Health Facility | Irrelevant results |
| 316 | 1989 | Harrison, K. A. | Tropical obstetrics and gynaecology. 2. Maternal mortality | Irrelevant results |
| 317 | 2010 | Wandabwa, J.; Doyle, P.; Kiondo, P.; Campbell, O.; Maconichie, N.; Welishe, G. | Risk factors for severe pre-eclampsia and eclampsia in Mulago Hospital, Kampala, Uganda | Irrelevant results |
| 318 | 2003 | Kaye, D.; Mirembe, F.; Aziga, F.; Namulema, B. | Maternal mortality and associated near-misses among emergency intrapartum obstetric referrals in Mulago Hospital, Kampala, Uganda | Irrelevant results |
| 319 | 2013 | Mekonnen, Alemayehu; Mahmoud, Emebet; Fantahun, Mesganaw; Hagos, Seifu; Assegid, Meselech | Maternal morbidity in Butajira and Wukro districts, North and South central Ethiopia | Irrelevant results |
| 320 | 2014 | Mohamed, S.; Hussein, M. D. | PREVALENCE OF ACUTE MALNUTRITION IN PRE-SCHOOL CHILDREN IN A RURAL AREA OF NORTHERN SUDAN | Irrelevant results |
| 321 | 2008 | Sudhinaraset, May | Reducing unsafe abortion in Nigeria | Irrelevant results |
| 322 | 2011 | Adesina, O.; Oladokun, A.; Akinyemi, O.; Akingbola, T.; Awolude, O.; Adewole, I. | Risk of anaemia in HIV positive pregnant women in Ibadan, south west Nigeria | Irrelevant results |
| 323 | 2009 | Olusanya, B. O.; Somefun, A. O. | Sensorineural hearing loss in infants with neonatal jaundice in Lagos: a community-based study | Irrelevant results |
| 324 | 2002 | Ndosi, Noah K.; Mtawali, M. L. W. | The nature of puerperal psychosis at Muhimbili National Hospital: its physical co-morbidity, associated main obstetric and social factors | Irrelevant results |
| 325 | 2006 | Satti, Alia; Elmusharaf, Susan; Bedri, Hibba; Idris, Tayseer; Hashim, M. Sir K.; Suliman, Gaafar I.; Almroth, Lars | Prevalence and determinants of the practice of genital mutilation of girls in Khartoum, Sudan | Irrelevant results |
| 326 | 2008 | Biddlecom, Ann | Unsafe abortion in Kenya | Irrelevant results |
| 327 | 2004 | Ayaya, S. O.; Esamai, F. O.; Rotich, J.; Olwambula, A. R. | Socio-economic factors predisposing under five-year-old children to severe protein energy malnutrition at the Moi Teaching and Referral Hospital, Eldoret, Kenya | Irrelevant results |
| 328 | 2013 | Habte, Dereje; Asrat, Kalid; Magafu, Mgaywa G. M. D.; Ali, Ibrahim M.; Benti, Tadele; Abtew, Wubeshet; Tegegne, Girma; Abera, Dereje; Shiferaw, Solomon | Maternal risk factors for childhood anaemia in Ethiopia | Irrelevant results |
| 329 | 2011 | Nwizu, E. N.; Iliyasu, Z.; Ibrahim, S. A.; Galadanci, H. S. | Socio-demographic and maternal factors in anaemia in pregnancy at booking in Kano, northern Nigeria | Irrelevant results |
| 330 | 2010 | Souza, João Paulo; Cecatti, Jose Guilherme; Faundes, Anibal; Morais, Sirlei Siani; Villar, Jose; Carroli, Guillermo; and al | Maternal near miss and maternal death in the World Health Organization's 2005 global survey on maternal and perinatal health | Irrelevant results |
| 331 | 2020 | Pham, Olivia; Usha, Ranji | Racial Disparities in Maternal and Infant Health: An Overview - Issue Brief | Irrelevant results |
| 332 | 2019 | Organisation Mondiale de la Santé | Mortalité maternelle | Irrelevant results |
| 333 | 2021 | Glazer, Kimberly B.; Zeitlin, Jennifer; Egorova, Natalia N.; Janevic, Teresa; Balbierz, Amy; Hebert, Paul L.; Howell, Elizabeth A. | Hospital Quality of Care and Racial and Ethnic Disparities in Unexpected Newborn Complications | Irrelevant results |
| 334 | 2021 | Olapeju, Bolanle; Hong, Xiumei; Wang, Guoying; Summers, Amber; Burd, Irina; Cheng, Tina L.; Wang, Xiaobin | Birth outcomes across the spectrum of maternal age: dissecting aging effect versus confounding by social and medical determinants | Irrelevant results |
| 335 | 2008 | Adisasmita, Asri; Deviany, Poppy E.; Nandiaty, Fitri; Stanton, Cynthia; Ronsmans, Carine | Obstetric near miss and deaths in public and private hospitals in Indonesia | Irrelevant results |
| 336 | 2019 | Lange, Isabelle L.; Gherissi, Atf; Chou, Doris; Say, Lale; Filippi, Veronique | What maternal morbidities are and what they mean for women: A thematic analysis of twenty years of qualitative research in low and lower-middle income countries | Irrelevant results |
| 337 | 1991 | Stones, W.; Lim, W.; Al-Azzawi, F.; Kelly, M. | An investigation of maternal morbidity with identification of life-threatening 'near miss' episodes | Irrelevant results |
| 338 | 2019 | Liese, Kylea L.; Mogos, Mulubrhan; Abboud, Sarah; Decocker, Karen; Koch, Abigail R.; Geller, Stacie E. | Racial and Ethnic Disparities in Severe Maternal Morbidity in the United States | Irrelevant results |
| 339 | 2020 | de Morais, Luiza Russo; Patz, Beatriz Costa; Campanharo, Felipe Favorette; Dualib, Patricia Médici; Sun, Sue Yazaki; Mattar, Rosiane | Maternal near miss and potentially life-threatening condition determinants in patients with type 1 diabetes mellitus at a university hospital in São Paulo, Brazil: a retrospective study | Irrelevant results |
| 340 | 2017 | Lisonkova, Sarka; Potts, Jayson; Muraca, Giulia M.; Razaz, Neda; Sabr, Yasser; Chan, Wee-Shian; Kramer, Michael S. | Maternal age and severe maternal morbidity: A population-based retrospective cohort study | Irrelevant results |
| 341 | 2017 | Moreira, Douglas dos Santos; Gubert, Muriel Bauermann | Healthcare and sociodemographic conditions related to severe maternal morbidity in a state representative population, Federal District, Brazil: A cross-sectional study | Irrelevant results |
| 342 | 2014 | Larru, Beatriz; Zaoutis, Theoklis E. | Chapter 13 - Infection and Immunity | Irrelevant results |
| 343 | 2005 | Lawrence, Ruth A.; Lawrence, Robert M. | Chapter 12 - Normal growth, failure to thrive, and obesity in the breastfed infant | Irrelevant results |
| 344 | 1997 | Sickles, Robin C.; Taubman, Paul | Chapter 11 Mortality and morbidity among adults and the elderly | Irrelevant results |
| 345 | 2001 | Fanaroff, Avroy A.; Kiwi, Robert; Shah, Dinesh M. | Chapter 1 - Antenatal and Intrapartum Care of the High-Risk Infant | Irrelevant results |
| 346 | 2018 | Rogawski, Elizabeth T; Liu, Jie; Platts-Mills, James A; Kabir, Furqan; Lertsethtakarn, Paphavee; Siguas, Mery; and al | Use of quantitative molecular diagnostic methods to investigate the effect of enteropathogen infections on linear growth in children in low-resource settings: longitudinal analysis of results from the MAL-ED cohort study | Irrelevant results |
| 347 | 2015 |  | Heterogeneity of postpartum depression: a latent class analysis | Irrelevant results |
| 348 | 2017 | Putnam, Karen T; Wilcox, Marsha; Robertson-Blackmore, Emma; Sharkey, Katherine; Bergink, Veerle; Munk-Olsen, Trine; and al | Clinical phenotypes of perinatal depression and time of symptom onset: analysis of data from an international consortium | Irrelevant results |
| 349 | 2003 | Klonoff-Cohen, Hillary; Lam-Kruglick, Phung; Gonzalez, Cristina | Effects of maternal and paternal alcohol consumption on the success rates of in vitro fertilization and gamete intrafallopian transfer | Irrelevant results |
| 350 | 2018 | Hanifi, Syed Manzoor Ahmed; Ravn, Henrik; Aaby, Peter; Bhuiya, Abbas | Where girls are less likely to be fully vaccinated than boys: Evidence from a rural area in Bangladesh | Irrelevant results |
| 351 | 2019 | Biradar, Rajeshwari; Patel, Kamalesh Kumar; Prasad, Jang Bahadur | Effect of birth interval and wealth on under-5 child mortality in Nigeria | Irrelevant results |
| 352 | 2015 | Garg, Nitin; Silverberg, Jonathan I. | Epidemiology of childhood atopic dermatitis | Irrelevant results |
| 353 | 2019 | Cruz, Paulina; Granados, Andrea | Type 2 Diabetes in Latino Youth: A Clinical Update and Current Challenges | Irrelevant results |
| 354 | 2010 | Fuller, Stephanie; Rajagopalan, Ramakrishnan; Jarvik, Gail P.; Gerdes, Marsha; Bernbaum, Judy; Wernovsky, Gil; and al | Deep Hypothermic circulatory arrest does not impair neurodevelopmental outcome in school-age children after infant cardiac surgery | Irrelevant results |
| 355 | 2009 | Nwagha, Uchenna Ifeanyi; Ugwu, Valentine Onyebuchi; Nwagha, Theresa Ukamaka; Anyaehie, Bond Ugochukwu | Asymptomatic Plasmodium parasitaemia in pregnant Nigerian women: almost a decade after Roll Back Malaria | Irrelevant results |
| 356 | 2016 | Sullivan, Ginger; O’Brien, Beverley; Mwini-Nyaledzigbor, Prudence | Sources of support for women experiencing obstetric fistula in northern Ghana: A focused ethnography | Irrelevant results |
| 357 | 2018 | Lennon, Robert P.; Claussen, Keith A.; Kuersteiner, Karl A. | State of the Heart: An Overview of the Disease Burden of Cardiovascular Disease from an Epidemiologic Perspective | Irrelevant results |
| 358 | 2014 | Dickson, Kim E; Simen-Kapeu, Aline; Kinney, Mary V; Huicho, Luis; Vesel, Linda; Lackritz, Eve; and al | Every Newborn: health-systems bottlenecks and strategies to accelerate scale-up in countries | Irrelevant results |
| 359 | 2016 | Booster, Genery D.; Oland, Alyssa A.; Bender, Bruce G. | Psychosocial Factors in Severe Pediatric Asthma | Irrelevant results |
| 360 | 2020 | Pavličev, Mihaela; Romero, Roberto; Mitteroecker, Philipp | Evolution of the human pelvis and obstructed labor: new explanations of an old obstetrical dilemma | Irrelevant results |
| 361 | 2010 | Bittles, A. H.; Black, M. L. | The impact of consanguinity on neonatal and infant health | Irrelevant results |
| 362 | 2005 | Boyer, Kenneth M.; Holfels, Ellen; Roizen, Nancy; Swisher, Charles; Mack, Douglas; Remington, Jack; and al | Risk factors for Toxoplasma gondii infection in mothers of infants with congenital toxoplasmosis: Implications for prenatal management and screening | Irrelevant results |
| 363 | 2021 | Wang, Xin; Li, You; Deloria-Knoll, Maria; Madhi, Shabir A; Cohen, Cheryl; Ali, Asad; Basnet, Sudha; | Global burden of acute lower respiratory infection associated with human metapneumovirus in children under 5 years in 2018: a systematic review and modelling study | Irrelevant results |
| 364 | 2019 | Maghsoudlou, Siavash; Yu, Zhijie Michael; Beyene, Joseph; McDonald, Sarah D. | Phenotypic Classification of Preterm Birth Among Nulliparous Women: A Population-Based Cohort Study | Irrelevant results |
| 365 | 2016 | Sargsyan, Sergey; Movsesyan, Yeva; Melkumova, Marina; Babloyan, Ara | Child and Adolescent Health in Armenia: Experiences and Learned Lessons | Irrelevant results |
| 366 | 2020 | Sharma, Deva; Ogbenna, Ann Abiola; Kassim, Adetola; Andrews, Jennifer | Transfusion support in patients with sickle cell disease | Irrelevant results |
| 367 | 2009 | Lawn, Joy E.; Lee, Anne CC; Kinney, Mary; Sibley, Lynn; Carlo, Wally A.; Paul, Vinod K.; and al | Two million intrapartum-related stillbirths and neonatal deaths: Where, why, and what can be done? | Irrelevant results |
| 368 | 2020 | Huschke, Susann; Murphy-Tighe, Sylvia; Barry, Maebh | Perinatal mental health in Ireland: A scoping review | Irrelevant results |
| 369 | 2021 | Baqui, Abdullah H.; Koffi, Alain K.; McCollum, Eric D.; Roy, Arunangshu D.; Chowdhury, Nabidul H.; Rafiqullah, Iftekhar; Ahmed, Zabed B.; and al | Impact of national introduction of ten-valent pneumococcal conjugate vaccine on invasive pneumococcal disease in Bangladesh: Case-control and time-trend studies | Irrelevant results |
| 370 | 2012 | Perkins, Ellen; Murphy, Susan K.; Murtha, Amy P.; Schildkraut, Joellen; Jirtle, Randy L.; Demark-Wahnefried, Wendy; and al | Insulin-Like Growth Factor 2/H19 Methylation at Birth and Risk of Overweight and Obesity in Children | Irrelevant results |
| 371 | 2014 | Niyibizi, Joseph; Rodier, Caroline; Wassef, Maggy; Trottier, Helen | Risk factors for the development and severity of juvenile-onset recurrent respiratory papillomatosis: A systematic review | Irrelevant results |
| 372 | 2013 | Coral-Vázquez, Ramón Mauricio; Romero Arauz, Juan Fernando; Canizales-Quinteros, Samuel; Coronel, Agustín; Valencia Villalvazo, Elith Yazmin; Hernández Rivera, Jaime; and al | Analysis of polymorphisms and haplotypes in genes associated with vascular tone, hypertension and oxidative stress in Mexican-Mestizo women with severe preeclampsia | Irrelevant results |
| 373 | 2020 | Mavhu, Webster; Willis, Nicola; Mufuka, Juliet; Bernays, Sarah; Tshuma, Maureen; Mangenah, Collin; and al | Effect of a differentiated service delivery model on virological failure in adolescents with HIV in Zimbabwe (Zvandiri): a cluster-randomised controlled trial | Irrelevant results |
| 374 | 2020 | Badakhsh, Mahin; Hastings-Tolsma, Marie; Firouzkohi, Mohammadreza; Amirshahi, Mehrbanoo; Hashemi, Zohreh Sadat | The lived experience of women with a high-risk pregnancy: A phenomenology investigation | Irrelevant results |
| 375 | 2003 | Yanda, K.; Smith, S. V.; Rosenfield, A. | Reproductive health and human rights | Irrelevant results |
| 376 | 2019 | Gupta, Renu; Brown, Hilary K.; Barker, Lucy C.; Dennis, Cindy-Lee; Vigod, Simone N. | Rapid repeat pregnancy in women with schizophrenia | Irrelevant results |
| 377 | 2019 | Fajtova, Vera | Impact of Family History of Diabetes on Diabetes Control and Complications | Irrelevant results |
| 378 | 2002 | Foxman, Betsy | Epidemiology of urinary tract infections: incidence, morbidity, and economic costs | Irrelevant results |
| 379 | 2004 | Richey, Lisa Ann | From the Policies to the Clinics: The Reproductive Health Paradox in Post-Adjustment Health Care | Irrelevant results |
| 380 | 2012 | Bell, Suzanne; Prata, Ndola; Lahiff, Maureen; Eskenazi, Brenda | Civil unrest and birthweight: An exploratory analysis of the 2007/2008 Kenyan Crisis | Irrelevant results |
| 381 | 2019 | Pullan, Rachel L; Halliday, Katherine E; Oswald, William E; Mcharo, Carlos; Beaumont, Emma; Kepha, Stella; and al | Effects, equity, and cost of school-based and community-wide treatment strategies for soil-transmitted helminths in Kenya: a cluster-randomised controlled trial | Irrelevant results |
| 382 | 2010 | Agee, Mark D. | Reducing child malnutrition in Nigeria: Combined effects of income growth and provision of information about mothers’ access to health care services | Irrelevant results |
| 383 | 2018 | Thorsness, Katie R.; Watson, Corey; LaRusso, Elizabeth M. | Perinatal anxiety: approach to diagnosis and management in the obstetric setting | Irrelevant results |
| 384 | 2013 | Daniele, Vittorio; Ostuni, Nicola | The burden of disease and the IQ of nations | Irrelevant results |
| 385 | 2005 | Bloomberg, Gordon R.; Chen, Edith | The relationship of psychologic stress with childhood asthma | Irrelevant results |
| 386 | 2020 | Reuter, Anja; Seddon, James A.; Marais, Ben J.; Furin, Jennifer | Preventing tuberculosis in children: A global health emergency | Irrelevant results |
| 387 | 2007 | Mosser, P.; Schmutzhard, E.; Winkler, A. S. | The pattern of epileptic seizures in rural Tanzania | Irrelevant results |
| 388 | 2013 | Mullany, Luke C.; Khatry, Subarna K.; Katz, Joanne; Stanton, Cynthia K.; Lee, Anne C. C.; Darmstadt, Gary L.; LeClerq, Steven C.; Tielsch, James M. | Injections during labor and intrapartum-related hypoxic injury and mortality in rural southern Nepal | Irrelevant results |
| 389 | 2019 | Roberts, Nicholas; James, Spencer; Delaney, Meghan; Fitzmaurice, Christina | The global need and availability of blood products: a modelling study | Irrelevant results |
| 390 | 2020 | Chaemsaithong, Piya; Sahota, Daljit Singh; Poon, Liona C. | First trimester preeclampsia screening and prediction | Irrelevant results |
| 391 | 2016 | Siribamrungwong, Monchai; Chinudomwong, Pawadee | Relation between acute kidney injury and pregnancy-related factors | Irrelevant results |
| 392 | 2003 | Foxman, Betsy | Epidemiology of urinary tract infections: Incidence, morbidity, and economic costs | Irrelevant results |
| 393 | 2010 | Kuhn, Louise; Aldrovandi, Grace | Survival and Health Benefits of Breastfeeding Versus Artificial Feeding in Infants of HIV-Infected Women: Developing Versus Developed World | Irrelevant results |
| 394 | 2019 | Heise, Lori; Greene, Margaret E; Opper, Neisha; Stavropoulou, Maria; Harper, Caroline; Nascimento, Marcos; and al | Gender inequality and restrictive gender norms: framing the challenges to health | Irrelevant results |
| 395 | 2021 | Araújo, Liubiana Arantes de; Veloso, Cássio Frederico; Souza, Matheus de Campos; Azevedo, João Marcos Coelho de; Tarro, Giulio | The potential impact of the COVID-19 pandemic on child growth and development: a systematic review | Irrelevant results |
| 396 | 2007 | Hunninghake, Gary M.; Soto-Quiros, Manuel E.; Avila, Lydiana; Ly, Ngoc P.; Liang, Catherine; Sylvia, Jody S.; Klanderman, Barbara J.; Silverman, Edwin K.; Celedón, Juan C. | Sensitization to Ascaris lumbricoides and severity of childhood asthma in Costa Rica | Irrelevant results |
| 397 | 2021 | Kahkoska, Anna R.; Dabelea, Dana | Diabetes in Youth: A Global Perspective | Irrelevant results |
| 398 | 2021 | Ferschke, Nicole | Preventing and Treating Tobacco Use | Irrelevant results |
| 399 | 2021 | Getahun, Darios; Fassett, Michael J.; Jacobsen, Steven J.; Sacks, David A.; Murali, Sameer B.; Peltier, Morgan R.; and al | Perinatal outcomes after bariatric surgery | Irrelevant results |
| 400 | 2013 | Tudehope, David; Vento, Maximo; Bhutta, Zulfiqar; Pachi, Paulo | Nutritional Requirements and Feeding Recommendations for Small for Gestational Age Infants | Irrelevant results |
| 401 | 2020 | Hilton, Ebony J.; Goff, Kristina L.; Sreedharan, Roshni; Lunardi, Nadia; Batakji, Mariam; Rosenberger, Dorothea S. | The Flaw of Medicine: Addressing Racial and Gender Disparities in Critical Care | Irrelevant results |
| 402 | 2020 | Tewabe, Tilahun; Ayalew, Tilksew; Abdanur, Abdulhakim; Jenbere, Demoze; Ayehu, Mastewal; Talema, Girma; Asmare, Eden | Contraceptive use and associated factors among sexually active reproductive age HIV positive women attending ART clinic at Felege Hiwot Referral Hospital, Northwest Ethiopia: A cross-sectional study | Irrelevant results |
| 403 | 2021 | Prosser, J. Drew; Holmes, Thomas W.; Seyyedi, Mohammad; Choo, Daniel I. | Congenital cytomegalovirus (CMV) for the pediatric otolaryngologist | Irrelevant results |
| 404 | 2017 | Ruducha, Jenny; Mann, Carlyn; Singh, Neha S; Gemebo, Tsegaye D; Tessema, Negussie S; Baschieri, Angela; and al | How Ethiopia achieved Millennium Development Goal 4 through multisectoral interventions: a Countdown to 2015 case study | Irrelevant results |
| 405 | 2007 | Worrall, Eve; Morel, Chantal; Yeung, Shunmay; Borghi, Jo; Webster, Jayne; Hill, Jenny; Wiseman, Virginia; Mills, Anne | The economics of malaria in pregnancy—a review of the evidence and research priorities | Irrelevant results |
| 406 | 2020 | Rahman, Mohammad Hifz Ur; Malik, Manzoor Ahmad; Chauhan, Shekhar; Patel, Ratna; Singh, Ashish; Mittal, Anshu | Examining the linkage between open defecation and child malnutrition in India | Irrelevant results |
| 407 | 2019 | Nambuusi, Betty Bukenya; Ssempiira, Julius; Makumbi, Fredrick E.; Kasasa, Simon; Vounatsou, Penelope | The effects and contribution of childhood diseases on the geographical distribution of all-cause under-five mortality in Uganda | Irrelevant results |
| 408 | 2017 | Deshpande, Neha A.; Kucirka, Lauren M.; Smith, Randi N.; Oxford, Corrina M. | Pregnant trauma victims experience nearly 2-fold higher mortality compared to their nonpregnant counterparts | Irrelevant results |
| 409 | 2002 | Strunk, Robert C.; Ford, Jean G.; Taggart, Virginia | Reducing disparities in asthma care: Priorities for research—National Heart, Lung, and Blood Institute Workshop Report | Irrelevant results |
| 410 | 2021 | Donker, Albertine E.; van der Staaij, Hilde; Swinkels, Dorine W. | The critical roles of iron during the journey from fetus to adolescent: Developmental aspects of iron homeostasis | Irrelevant results |
| 411 | 2018 | Finnerty, Fionnuala; George, Stefan; Eziefula, Alice Chi | The health of recent migrants from resource-poor countries | Irrelevant results |
| 412 | 2021 | Mackintosh, Nicola; Gong, Qian (Sarah); Hadjiconstantinou, Michelle; Verdezoto, Nervo | Digital mediation of candidacy in maternity care: Managing boundaries between physiology and pathology | Irrelevant results |
| 413 | 2012 | Johnson, Ari; Goss, Adeline; Beckerman, Jessica; Castro, Arachu | Hidden costs: The direct and indirect impact of user fees on access to malaria treatment and primary care in Mali | Irrelevant results |
| 414 | 2017 | Nguyen, T. K. P.; Tran, T. H.; Roberts, C. L.; Fox, G. J.; Graham, S. M.; Marais, B. J. | Risk factors for child pneumonia - focus on the Western Pacific Region | Irrelevant results |
| 415 | 2009 | Adhikari, M. | Tuberculosis and tuberculosis/HIV co-infection in pregnancy | Irrelevant results |
| 416 | 2016 | Baranov, Alexander; Namazova-Baranova, Leyla; Albitskiy, Valeriy; Ustinova, Natalia; Terletskaya, Rimma; Komarova, Olga | The Russian Child Health Care System | Irrelevant results |
| 417 | 2020 | Lao, Terence T. | Drug-induced liver injury in pregnancy | Irrelevant results |
| 418 | 2020 | Gurven, Michael D.; Lieberman, Daniel E. | WEIRD bodies: mismatch, medicine and missing diversity | Irrelevant results |
| 419 | 2010 | Hodgkinson, Stacy C.; Colantuoni, Elizabeth; Roberts, Debra; Berg-Cross, Linda; Belcher, Harolyn M. E. | Depressive Symptoms and Birth Outcomes among Pregnant Teenagers | Irrelevant results |
| 420 | 2019 | Mariani, Gonzalo L.; Vain, Nestor E. | The rising incidence and impact of non-medically indicated pre-labour cesarean section in Latin America | Irrelevant results |
| 421 | 2007 | Lipscomb, Hester J.; Dement, John M.; Epling, Carol A.; Gaynes, Bradley N.; McDonald, Mary Anne; Schoenfisch, Ashley L. | Depressive symptoms among working women in rural North Carolina: A comparison of women in poultry processing and other low-wage jobs | Irrelevant results |
| 422 | 2006 | Chaparro, Camila M; Neufeld, Lynnette M; Tena Alavez, Gilberto; Eguia-Líz Cedillo, Raúl; Dewey, Kathryn G | Effect of timing of umbilical cord clamping on iron status in Mexican infants: a randomised controlled trial | Irrelevant results |
| 423 | 2014 | Gayawan, Ezra | Spatial analysis of choice of place of delivery in Nigeria | Irrelevant results |
| 424 | 2012 | Burris, Heather H.; Rifas-Shiman, Sheryl L.; Camargo, Carlos A.; Litonjua, Augusto A.; Huh, Susanna Y.; Rich-Edwards, Janet W.; Gillman, Matthew W. | Plasma 25-hydroxyvitamin D during pregnancy and small-for-gestational age in black and white infants | Irrelevant results |
| 425 | 2018 | Desai, Meghna; Hill, Jenny; Fernandes, Silke; Walker, Patrick; Pell, Christopher; Gutman, Julie; Kayentao, Kassoum; Gonzalez, Raquel; Webster, Jayne; Greenwood, Brian; Cot, Michel; ter Kuile, Feiko O | Prevention of malaria in pregnancy | Irrelevant results |
| 426 | 2021 | Rhedin, Samuel; Lundholm, Cecilia; Osvald, Emma Caffrey; Almqvist, Catarina | Pneumonia in Infancy and Risk for Asthma: The Role of Familial Confounding and Pneumococcal Vaccination | Irrelevant results |
| 427 | 2016 | Vieira, Matias C.; Pasupathy, Dharmintra | Understanding perinatal mortality | Irrelevant results |
| 428 | 2021 | McIntyre, S.; Nelson, K. B.; Mulkey, S. B.; Lechpammer, M.; Molloy, E.; Badawi, N. | Neonatal encephalopathy: Focus on epidemiology and underexplored aspects of etiology | Irrelevant results |
| 429 | 2017 | Bachani, Abdulgafoor M.; Taber, Niloufer; Mehmood, Amber; Hung, Yuen Wai; Botchey, Isaac; Al-Kashmiri, Ammar; Hyder, Adnan A. | Adolescent and Young Adult Injuries in Developing Economies: A Comparative Analysis from Oman and Kenya | Irrelevant results |
| 430 | 2016 | Chabarria, Kristin C.; Racusin, Diana A.; Antony, Kathleen M.; Kahr, Maike; Suter, Melissa A.; Mastrobattista, Joan M.; Aagaard, Kjersti M. | Marijuana use and its effects in pregnancy | Irrelevant results |
| 431 | 2013 | Peck, Michael; Pressman, Melissa A. | The correlation between burn mortality rates from fire and flame and economic status of countries | Irrelevant results |
| 432 | 2013 | Fagundes, Christopher P.; Glaser, Ronald; Kiecolt-Glaser, Janice K. | Stressful early life experiences and immune dysregulation across the lifespan | Irrelevant results |
| 433 | 2020 | Huang, Junwen; Xu, Damin; Yang, Li | Acute Kidney Injury in Asia: Disease Burden | Irrelevant results |
| 434 | 2012 | Chen, Shou-Chien; Tan, Lia-Beng; Huang, Li-Min; Chen, Kow-Tong | Rotavirus infection and the current status of rotavirus vaccines | Irrelevant results |
| 435 | 2015 | Mazur, Natalie I; Martinón-Torres, Federico; Baraldi, Eugenio; Fauroux, Brigitte; Greenough, Anne; Heikkinen, Terho; Manzoni, Paolo; Mejias, Asuncion; Nair, Harish; Papadopoulos, Nikolaos G; Polack, Fernando P; Ramilo, Octavio; Sharland, Mike; Stein, Renato; Madhi, Shabir A; Bont, Louis | Lower respiratory tract infection caused by respiratory syncytial virus: current management and new therapeutics | Irrelevant results |
| 436 | 2017 | Williams, Edith M.; Egede, Leonard; Faith, Trevor; Oates, James | Effective Self-Management Interventions for Patients With Lupus: Potential Impact of Peer Mentoring | Irrelevant results |
| 437 | 2019 | Kesavan, Kalpashri; Devaskar, Sherin U. | Intrauterine Growth Restriction: Postnatal Monitoring and Outcomes | Irrelevant results |
| 438 | 2017 | Karim, Tasneem; Muhit, Mohammad; Khandaker, Gulam | Interventions to prevent respiratory diseases - Nutrition and the developing world | Irrelevant results |
| 439 | 2009 | Lee, Henry Chong; Gould, Jeffrey B. | Factors Influencing Breast Milk versus Formula Feeding at Discharge for Very Low Birth Weight Infants in California | Irrelevant results |
| 440 | 2004 | Russo, Denise; Purohit, Vishnudutt; Foudin, Laurie; Salin, Marvin | Workshop on alcohol use and health disparities 2002: a call to arms | Irrelevant results |
| 441 | 2021 | Ana, Yamuna; Prafulla, Shriyan; Deepa, Ravi; Babu, Giridhara R. | Emerging and Public Health Challenges Existing in Gestational Diabetes Mellitus and Diabetes in Pregnancy | Irrelevant results |
| 442 | 2020 | Kim, Jin Tack; Kim, Hwan Soo; Chun, Yoon Hong; Yoon, Jong-seo; Kim, Hyun Hee | Effect of multi-ethnicity and ancestry on prevalence of allergic disease | Irrelevant results |
| 443 | 2016 | Evans, Ceri; Jones, Christine E; Prendergast, Andrew J | HIV-exposed, uninfected infants: new global challenges in the era of paediatric HIV elimination | Irrelevant results |
| 444 | 2021 | Ghimire, Upama; Papabathini, Shireen Salome; Kawuki, Joseph; Obore, Nathan; Musa, Taha Hussein | Depression during pregnancy and the risk of low birth weight, preterm birth and intrauterine growth restriction- an updated meta-analysis | Irrelevant results |
| 445 | 2020 | Otieno, Peter O.; Wambiya, Elvis O. A.; Mohamed, Shukri M.; Mutua, Martin Kavao; Kibe, Peter M.; Mwangi, Bonventure; Donfouet, Hermann Pythagore Pierre | Access to primary healthcare services and associated factors in urban slums in Nairobi-Kenya | Irrelevant results |
| 446 | 2008 | Hendrix, Nancy; Berghella, Vincenzo | Non-Placental Causes of Intrauterine Growth Restriction | Irrelevant results |
| 447 | 2012 | Watts, Kimberly Danieli | Healthcare Inequalities in Paediatric Respiratory Diseases | Irrelevant results |
| 448 | 2020 | Meng, Fanyi; Zuo, Kevin J.; Amar-Zifkin, Alexandre; Baird, Robert; Cugno, Sabrina; Poenaru, Dan | Pediatric burn contractures in low- and lower middle-income countries: A systematic review of causes and factors affecting outcome | Irrelevant results |
| 449 | 2011 | Wadhwa, Pathik D.; Entringer, Sonja; Buss, Claudia; Lu, Michael C. | The Contribution of Maternal Stress to Preterm Birth: Issues and Considerations | Irrelevant results |
| 450 | 2021 | VanNoy, Brianna N.; Bowleg, Lisa; Marfori, Cherie; Moawad, Gaby; Zota, Ami R. | Black Women's Psychosocial Experiences with Seeking Surgical Treatment for Uterine Fibroids: Implications for Clinical Practice | Irrelevant results |
| 451 | 2016 | Ko, Young; Chee, Wonshik; Im, Eun-Ok | Factors Associated With Perceived Health Status of Multiracial/Ethnic Midlife Women in the United States | Irrelevant results |
| 452 | 2000 | Vimercati, Antonella; Greco, Pantaleo; Loverro, Giuseppe; Lopalco, Pietro Luigi; Pansini, Valeria; Selvaggi, Luigi | Maternal complications after caesarean section in HIV infected women | Irrelevant results |
| 453 | 2021 | Chaplin, Anna B.; Smith, Nick; Jones, Peter B.; Khandaker, Golam M. | Direction of association between Cardiovascular risk and depressive symptoms during the first 18 years of life: A prospective birth cohort study | Irrelevant results |
| 454 | 2021 | Hustedde, Carol | Adverse Childhood Experiences | Irrelevant results |
| 455 | 2021 | Gabbs, Melissa H. J.; Dart, Allison B.; Woo, Meaghan R.; Pinto, Teresa; Wicklow, Brandy A. | Poor sleep, increased stress, and metabolic co-morbidity in adolescents and youth with type 2 diabetes. | Irrelevant results |
| 456 | 2019 | Brink, C.; Isaacs, Q.; Scriba, M. F.; Nathire, M. E. H.; Rode, H.; Martinez, R. | Infant burns: A single institution retrospective review | Irrelevant results |
| 457 | 2016 | Kalhan, Satish C. | One carbon metabolism in pregnancy: Impact on maternal, fetal and neonatal health | Irrelevant results |
| 458 | 2017 | Haile, Zelalem T.; Kingori, Caroline; Teweldeberhan, Asli K.; Chavan, Bhakti | The relationship between history of hormonal contraceptive use and iron status among women in Tanzania: A population-based study | Irrelevant results |
| 459 | 2021 | Theiler, Regan N.; Wick, Myra; Mehta, Ramila; Weaver, Amy L.; Virk, Abinash; Swift, Melanie | Pregnancy and birth outcomes after SARS-CoV-2 vaccination in pregnancy | Irrelevant results |
| 460 | 2017 | Carter, Ebony B.; Conner, Shayna N.; Cahill, Alison G.; Rampersad, Roxane; Macones, George A.; Tuuli, Methodius G. | Impact of fetal growth on pregnancy outcomes in women with severe preeclampsia | Irrelevant results |
| 461 | 2011 | Miller, Elizabeth M. | Maternal health and knowledge and infant health outcomes in the Ariaal people of northern Kenya | Irrelevant results |
| 462 | 2021 | Merrison, Hannah; Mangtani, Anisha; Quick, Tom | The shifting demographics of birth-related brachial plexus injury: The impact of socio-economic status and ethnic groups | Irrelevant results |
| 463 | 2021 | Watson, Sarah N.; McElroy, Steven J. | Potential Prenatal Origins of Necrotizing Enterocolitis | Irrelevant results |
| 464 | 2021 | Johnston, Amy; Tseung, Victrine; Dancey, Sonia R.; Visintini, Sarah M.; Coutinho, Thais; Edwards, Jodi D. | Use of race, ethnicity, and national origin in studies assessing cardiovascular risk in women with a history of hypertensive disorders of pregnancy. | Irrelevant results |
| 465 | 2019 | Kowalchuk, Alicia Ann; Gonzalez, Sandra J.; Zoorob, Roger J. | Substance Use Issues Among the Underserved: United States and International Perspectives | Irrelevant results |
| 466 | 2021 | Faisal-Cury, Alexandre; Rocha, Alexandra Caires; Elise Machado Ribeiro Silotto, Ana; Maurício de Oliveira Rodrigues, Daniel | Prevalence and associated risk factors of antenatal depression among Brazilian pregnant women: A population-based study | Irrelevant results |
| 467 | 2012 | De Jesus, Lilia C.; Pappas, Athina; Shankaran, Seetha; Kendrick, Douglas; Das, Abhik; Higgins, Rosemary D.; Bell, Edward F.; Stoll, Barbara J.; Laptook, Abbot R.; Walsh, Michele C. | Risk Factors for Post-Neonatal Intensive Care Unit Discharge Mortality among Extremely Low Birth Weight Infants | Irrelevant results |
| 468 | 2020 | Tewabe, Tilahun; Belachew, Amare | Determinants of Nutritional Status in School-Aged Children in Mecha, Northwest Ethiopia | Irrelevant results |
| 469 | 2021 | Papatheodorou, Stefania; Yao, Weiyu; Vieira, Carolina L. Z.; Li, Longxiang; Wylie, Blair J.; Schwartz, Joel; Koutrakis, Petros | Residential radon exposure and hypertensive disorders of pregnancy in Massachusetts, USA: A cohort study | Irrelevant results |
| 470 | 2018 | Ntenda, Peter Austin Morton; Chuang, Ying-Chih | Analysis of individual-level and community-level effects on childhood undernutrition in Malawi | Irrelevant results |
| 471 | 2015 | Cohen, Jacqueline M.; Kramer, Michael S.; Platt, Robert W.; Basso, Olga; Evans, Rhobert W.; Kahn, Susan R. | The association between maternal antioxidant levels in midpregnancy and preeclampsia | Irrelevant results |
| 472 | 2006 | Matsui, Elizabeth C.; Eggleston, Peyton A.; Buckley, Timothy J.; Krishnan, Jerry A.; Breysse, Patrick N.; Rand, Cynthia S.; Diette, Gregory B. | Household mouse allergen exposure and asthma morbidity in inner-city preschool children | Irrelevant results |
| 473 | 2009 | Bryant-Stephens, Tyra | Asthma disparities in urban environments | Irrelevant results |
| 474 | 2003 | As-Sanie, Sawsan; Mercer, Brian; Moore, John | The association between respiratory distress and nonpulmonary morbidity at 34 to 36 weeks' gestation | Irrelevant results |
| 475 | 2012 | Dauby, Nicolas; Goetghebuer, Tessa; Kollmann, Tobias R; Levy, Jack; Marchant, Arnaud | Uninfected but not unaffected: chronic maternal infections during pregnancy, fetal immunity, and susceptibility to postnatal infections | Irrelevant results |
| 476 | 2019 | Matoba, Nana; Yallapragada, Sushmita; Davis, Matthew M.; Ernst, Linda M.; Collins, James W.; Mestan, Karen K. | Racial differences in placental pathology among very preterm births | Irrelevant results |
| 477 | 2010 | Khan, Yasir; Bhutta, Zulfiqar A. | Nutritional Deficiencies in the Developing World: Current Status and Opportunities for Intervention | Irrelevant results |
| 478 | 2009 | Lieb, David C.; Snow, Rodney E.; DeBoer, Mark D. | Socioeconomic Factors in the Development of Childhood Obesity and Diabetes | Irrelevant results |
| 479 | 2013 | Imdad, Aamer; Bhutta, Zulfiqar A. | Nutritional Management of the Low Birth Weight/Preterm Infant in Community Settings: A Perspective from the Developing World | Irrelevant results |
| 480 | 2008 | Conde-Agudelo, Agustín; Villar, José; Lindheimer, Marshall | Maternal infection and risk of preeclampsia: Systematic review and metaanalysis | Irrelevant results |
| 481 | 2021 | Kinney, Patrick L.; Asante, Kwaku-Poku; Lee, Alison G.; Ae-Ngibise, Kenneth A.; Burkart, Katrin; Boamah-Kaali, Ellen; and al | Prenatal and Postnatal Household Air Pollution Exposures and Pneumonia Risk: Evidence From the Ghana Randomized Air Pollution and Health Study | Irrelevant results |
| 482 | 2006 | Emerson, Eric; Graham, Hilary; Hatton, Chris | The Measurement of Poverty and Socioeconomic Position in Research Involving People with Intellectual Disability | Irrelevant results |
| 483 | 2016 | van Weert, Britte; van den Berg, Denice; Hrudey, E. Jessica; Oostvogels, Adriëtte J. J. M.; de Miranda, Esteriek; Vrijkotte, Tanja G. M. | Is first trimester vitamin D status in nulliparous women associated with pregnancy related hypertensive disorders? | Irrelevant results |
| 484 | 2007 | Kaminsky, Lilian M.; Ananth, Cande V.; Prasad, Vinay; Nath, Carl; Vintzileos, Anthony M. | The influence of maternal cigarette smoking on placental pathology in pregnancies complicated by abruption | Irrelevant results |
| 485 | 2014 | Thomas, Timothy N.; Gausman, Jewel; Lattof, Samantha R.; Wegner, Mary Nell; Kearns, Annie D.; Langer, Ana | Improved maternal health since the ICPD: 20 years of progress | Irrelevant results |
| 486 | 2020 | Slogrove, Amy L; Powis, Kathleen M; Johnson, Leigh F; Stover, John; Mahy, Mary | Estimates of the global population of children who are HIV-exposed and uninfected, 2000–18: a modelling study | Irrelevant results |
| 487 | 2011 | Bryce, Jennifer; Victora, Cesar G.; Boerma, Ties; Peters, David H.; Black, Robert E. | Evaluating the scale-up for maternal and child survival: a common framework | Irrelevant results |
| 488 | 2013 | Osborne, Lauren M.; Monk, Catherine | Perinatal depression—The fourth inflammatory morbidity of pregnancy?: Theory and literature review | Irrelevant results |
| 489 | 2016 | Herba, Catherine M; Glover, Vivette; Ramchandani, Paul G; Rondon, Marta B | Maternal depression and mental health in early childhood: an examination of underlying mechanisms in low-income and middle-income countries | Irrelevant results |
| 490 | 2018 | Luan, Yibo; Fischer, Günther; Wada, Yoshihide; Sun, Laixiang; Shi, Peijun | Quantifying the impact of diet quality on hunger and undernutrition | Irrelevant results |
| 491 | 2017 | Keats, Emily C; Ngugi, Anthony; Macharia, William; Akseer, Nadia; Khaemba, Emma Nelima; Bhatti, Zaid; Rizvi, Arjumand; Tole, John; Bhutta, Zulfiqar A | Progress and priorities for reproductive, maternal, newborn, and child health in Kenya: a Countdown to 2015 country case study | Irrelevant results |
| 492 | 2016 | Fineberg, Anna M.; Ellman, Lauren M.; Schaefer, Catherine A.; Maxwell, Seth D.; Shen, Ling; Chaudhury, Nashid H.; Cook, Aundrea L.; Bresnahan, Michaeline A.; Susser, Ezra S.; Brown, Alan S. | Fetal exposure to maternal stress and risk for schizophrenia spectrum disorders among offspring: Differential influences of fetal sex | Irrelevant results |
| 493 | 2002 | Snow, Robert W.; Marsh, Kevin | The consequences of reducing transmission of Plasmodium falciparum in Africa | Irrelevant results |
| 494 | 2018 | Pham, Diana; Cormick, Gabriela; Amyx, Melissa M.; Gibbons, Luz; Doty, Meitra; Brown, Asia; Norwood, Angel; Daray, Federico M.; Althabe, Fernando; Belizán, José M. | Factors associated with postpartum depression in women from low socioeconomic level in Argentina: A hierarchical model approach | Irrelevant results |
| 495 | 2014 | LeVasseur, Michael T.; Goldstein, Neal D.; Welles, Seth L. | A public health perspective on HIV/AIDS in Africa: Victories and unmet challenges | Irrelevant results |
| 496 | 2017 | Rubin, Lisa; Belmaker, Ilana; Somekh, Eli; Urkin, Jacob; Rudolf, Mary; Honovich, Mira; Bilenko, Natalya; Grossman, Zachi | Maternal and child health in Israel: building lives | Irrelevant results |
| 497 | 2017 | Janusek, Linda Witek; Tell, Dina; Gaylord-Harden, Noni; Mathews, Herbert L. | Relationship of childhood adversity and neighborhood violence to a proinflammatory phenotype in emerging adult African American men: An epigenetic link | Irrelevant results |
| 498 | 2015 | Velickovic, Ivan; Dalloul, Mudar; Wong, Karen A.; Bakare, Olufunke; Schweis, Franz; Garala, Maya; and al | Complement factor B activation in patients with preeclampsia | Irrelevant results |
| 499 | 2019 | Mungul, Sheetal; Maharaj, Shivesh | Microbiology of paediatric deep neck space infection | Irrelevant results |
| 500 | 2010 | Bryce, Jennifer; Gilroy, Kate; Jones, Gareth; Hazel, Elizabeth; Black, Robert E; Victora, Cesar G | The Accelerated Child Survival and Development programme in west Africa: a retrospective evaluation | Irrelevant results |
| 501 | 2020 | Mena-Meléndez, Lucrecia | Ethnoracial child health inequalities in Latin America: Multilevel evidence from Bolivia, Colombia, Guatemala, and Peru | Irrelevant results |
| 502 | 2016 | Minjares-Granillo, Ramón O.; Reza-López, Sandra A.; Caballero-Valdez, Selene; Levario-Carrillo, Margarita; Chávez-Corral, Dora Virginia | Maternal and Perinatal Outcomes Among Adolescents and Mature Women: A Hospital-Based Study in the North of Mexico | Irrelevant results |
| 503 | 2017 | Smith-Greenaway, Emily; Clark, Shelley | Variation in the link between parental divorce and children’s health disadvantage in low and high divorce settings | Irrelevant results |
| 504 | 2017 | Malley, Christopher S.; Kuylenstierna, Johan C. I.; Vallack, Harry W.; Henze, Daven K.; Blencowe, Hannah; Ashmore, Mike R. | Preterm birth associated with maternal fine particulate matter exposure: A global, regional and national assessment | Irrelevant results |
| 505 | 2017 | Marchant, Arnaud; Sadarangani, Manish; Garand, Mathieu; Dauby, Nicolas; Verhasselt, Valerie; Pereira, Lenore; and al | Maternal immunisation: collaborating with mother nature | Irrelevant results |
| 506 | 2021 | Krishnan, Vanitha; Zaki, Rafdzah Ahmad; Nahar, Azmi Mohamed; Jalaludin, Muhammad Yazid; Majid, Hazreen Abdul | The longitudinal relationship between nutritional status and anaemia among Malaysian adolescents | Irrelevant results |
| 507 | 2016 | Faucett, Allison M.; Metz, Torri D.; DeWitt, Peter E.; Gibbs, Ronald S. | Effect of obesity on neonatal outcomes in pregnancies with preterm premature rupture of membranes | Irrelevant results |
| 508 | 2001 | Walraven, Gijs; Scherf, Caroline; West, Beryl; Ekpo, Gloria; Paine, Katie; Coleman, Rosalind; Bailey, Robin; Morison, Linda | The burden of reproductive-organ disease in rural women in The Gambia, West Africa | Irrelevant results |
| 509 | 2013 | Dong, Maolong; Zheng, Qijun; Ford, Stephen P.; Nathanielsz, Peter W.; Ren, Jun | Maternal obesity, lipotoxicity and cardiovascular diseases in offspring | Irrelevant results |
| 510 | 2020 | Fagbamigbe, Adeniyi F.; Kandala, Ngianga-Bakwin; Uthman, Olalekan A. | Severe acute malnutrition among under-5 children in low- and middle-income countries: A hierarchical analysis of associated risk factors | Irrelevant results |
| 511 | 2016 | Ganatra, Bela; Faundes, Anibal | Role of birth spacing, family planning services, safe abortion services and post-abortion care in reducing maternal mortality | Irrelevant results |
| 512 | 2017 | Li, Xinning; Veltre, David R.; Cusano, Antonio; Yi, Paul; Sing, David; Gagnier, Joel J.; Eichinger, Josef K.; Jawa, Andrew; Bedi, Asheesh | Insurance status affects postoperative morbidity and complication rate after shoulder arthroplasty | Irrelevant results |
| 513 | 2013 | Morgan, Jamie; Roberts, Scott | Maternal Sepsis | Irrelevant results |
| 514 | 2018 | Price, Alison J; Crampin, Amelia C; Amberbir, Alemayehu; Kayuni-Chihana, Ndoliwe; Musicha, Crispin; Tafatatha, Terence; and al | Prevalence of obesity, hypertension, and diabetes, and cascade of care in sub-Saharan Africa: a cross-sectional, population-based study in rural and urban Malawi | Irrelevant results |
| 515 | 2016 | Gelaye, Bizu; Rondon, Marta B; Araya, Ricardo; Williams, Michelle A | Epidemiology of maternal depression, risk factors, and child outcomes in low-income and middle-income countries | Irrelevant results |
| 516 | 2020 | Garry, N.; Farooq, I.; Milne, S.; Lindow, SW; Regan, C. | Trends in obstetric management of extreme preterm birth at 23 to 27 weeks’ gestation in a tertiary obstetric unit: A 10-year retrospective review | Irrelevant results |
| 517 | 2002 | Gilbert, Leah; Walker, Liz | Treading the path of least resistance: HIV/AIDS and social inequalities—a South African case study | Irrelevant results |
| 518 | 2000 | Obaro, Steven K | Prospects for pneumococcal vaccination in African children | Irrelevant results |
| 519 | 2021 | Chappell, Lucy C; Cluver, Catherine A; Kingdom, John; Tong, Stephen | Pre-eclampsia | Irrelevant results |
| 520 | 2021 | Bagattini, Angela M.; Policena, Gabriela; Minamisava, Ruth; Andrade, Ana Lucia S.; Nishioka, Sérgio de A.; Sinha, Anushua; Russell, Louise B.; Toscano, Cristiana M. | The data used to build the models: Pertussis morbidity and mortality burden considering various Brazilian data sources | Irrelevant results |
| 521 | 2011 | Ferrie, Joseph; Rolf, Karen | Socioeconomic status in childhood and health after age 70: A new longitudinal analysis for the U.S., 1895–2005 | Irrelevant results |
| 522 | 2020 | Dey, Nutifafa Eugene Yaw; Dziwornu, Emmanuel; Frimpong-Manso, Kwabena; Duah, Henry Ofori; Agbadi, Pascal | Correlates of child functional difficulties status in Ghana: A further analysis of the 2017/18 multiple indicator cluster survey | Irrelevant results |
| 523 | 2020 | Romagano, Matthew P.; Williams, Shauna F.; Apuzzio, Joseph J.; Sachdev, Devika; Flint, Matthew; Gittens-Williams, Lisa | Factors associated with attendance at the postpartum blood pressure visit in pregnancies complicated by hypertension | Irrelevant results |
| 524 | 2017 | Nilses, Carin; Persson, Margareta; Lindkvist, Marie; Petersson, Kerstin; Mogren, Ingrid | High weight gain during pregnancy increases the risk for emergency caesarean section – Population-based data from the Swedish Maternal Health Care Register 2011–2012 | Irrelevant results |
| 525 | 2018 | Pérez, J. Carola; Coo, Soledad; Irarrázaval, Matías | Is maternal depression related to mother and adolescent reports of family functioning? | Irrelevant results |
| 526 | 2018 | Ajibola, Gbolahan; Leidner, Jean; Mayondi, Gloria K.; van Widenfelt, Erik; Madidimalo, Tebogo; Petlo, Chipo; and al | HIV Exposure and Formula Feeding Predict Under-2 Mortality in HIV-Uninfected Children, Botswana | Irrelevant results |
| 527 | 2015 | Smith-Greenaway, Emily; Madhavan, Sangeetha | Maternal migration and child health: An analysis of disruption and adaptation processes in Benin | Irrelevant results |
| 528 | 2021 | Meghji, Jamilah; Mortimer, Kevin; Agusti, Alvar; Allwood, Brian W; Asher, Innes; Bateman, Eric D; and al | Improving lung health in low-income and middle-income countries: from challenges to solutions | Irrelevant results |
| 529 | 2020 | Miyazaki, Dai; Fukagawa, Kazumi; Okamoto, Shigeki; Fukushima, Atsuki; Uchio, Eiichi; Ebihara, Nobuyuki; Shoji, Jun; Namba, Kenichi; Shimizu, Yumiko | Epidemiological aspects of allergic conjunctivitis | Irrelevant results |
| 530 | 2021 | Espinosa, Camilo; Becker, Martin; Marić, Ivana; Wong, Ronald J.; Shaw, Gary M.; Gaudilliere, Brice; and al | Data-Driven Modeling of Pregnancy-Related Complications | Irrelevant results |
| 531 | 2013 | Tusting, Lucy S; Willey, Barbara; Lucas, Henry; Thompson, John; Kafy, Hmooda T; Smith, Richard; Lindsay, Steve W | Socioeconomic development as an intervention against malaria: a systematic review and meta-analysis | Irrelevant results |
| 532 | 2009 | Miller, Robin June; Sullivan, Mary C.; Hawes, Katheleen; Marks, Amy Kerivan | The Effects of Perinatal Morbidity and Environmental Factors on Health Status of Preterm Children at Age 12 | Irrelevant results |
| 533 | 2020 | Akubuilo, U. C.; Iloh, K. K.; Onu, J. U.; Iloh, O. N.; Ubesie, A. C.; Ikefuna, A. N. | Nutritional status of primary school children: Association with intelligence quotient and academic performance | Irrelevant results |
| 534 | 2015 | Aderoba, Adeniyi K.; Iribhogbe, Oseihie I.; Olagbuji, Biodun N.; Olokor, Oghenefegor E.; Ojide, Chiedozie K.; Ande, Adedapo B. | Prevalence of helminth infestation during pregnancy and its association with maternal anemia and low birth weight | Irrelevant results |
| 535 | 2014 | Plaza, Sonia Hernández; Padilla, Beatriz; Ortiz, Alejandra; Rodrigues, Elsa | The value of grounded theory for disentangling inequalities in maternal-child healthcare in contexts of diversity: A psycho-sociopolitical approach | Irrelevant results |
| 536 | 2008 | Fawcus, Susan R. | Maternal mortality and unsafe abortion | Irrelevant results |
| 537 | 2000 | Kalipeni, Ezekiel | Health and disease in southern Africa: a comparative and vulnerability perspective | Irrelevant results |
| 538 | 2015 | Kalu, Eziyi Iche; Ojide, Chiedozie Kingsley; Nwadike, Victor Ugochukwu; Korie, Francis Chukwuma; Ibeneme, Chikaodili Adaeze; Okafor, Godwin Chukwuebuka | Childhood tuberculosis in sub-Saharan Africa: A call to action | Irrelevant results |
| 539 | 2013 | Sanz-Ramos, Marta; Manno, Daniela; Kapambwe, Mirriam; Ndumba, Ida; Musonda, Kunda G.; Bates, Matthew; Chibumbya, Julia; Siame, Joshua; Monze, Mwaka; Filteau, Suzanne; Gompels, Ursula A. | Reduced Poliovirus vaccine neutralising-antibody titres in infants with maternal HIV-exposure | Irrelevant results |
| 540 | 2003 | Dasanayake, Ananda P; Russell, Shirley; Boyd, Doryck; Madianos, Phoebus N; Forster, Teri; Hill, Ed | Preterm low birth weight and periodontal disease among African Americans | Irrelevant results |
| 541 | 2012 | Rai, Rajesh Kumar; Singh, Prashant Kumar; Singh, Lucky | Utilization of Maternal Health Care Services among Married Adolescent Women: Insights from the Nigeria Demographic and Health Survey, 2008 | Irrelevant results |
| 542 | 2006 | Elizabeth Jesse, D.; Graham, Marilyn; Swanson, Mel | Psychosocial and Spiritual Factors Associated With Smoking and Substance Use During Pregnancy in African American and White Low-Income Women | Irrelevant results |
| 543 | 2009 | Al-Saleh, Eyad; Di Renzo, Gian Carlo | Actions needed to improve maternal health | Irrelevant results |
| 544 | 2011 | Hines, Annette Baker | Asthma: A Health Disparity Among African American Children: The Impact and Implications for Pediatric Nurses | Irrelevant results |
| 545 | 2014 | Darling, Anne Marie; Liu, Enju; Aboud, Said; Urassa, Willy; Spiegelman, Donna; Fawzi, Wafaie | Maternal hyperglycemia and adverse pregnancy outcomes in Dar es Salaam, Tanzania | Irrelevant results |
| 546 | 2018 | Acuña-Cordero, Ranniery; Sossa-Briceño, Monica P.; Rodríguez-Martínez, Carlos E. | Predictors of hospitalization for acute lower respiratory infections during the first two years of life in a population of preterm infants with bronchopulmonary dysplasia | Irrelevant results |
| 547 | 2016 | Olveda, Remigio M; Acosta, Luz P; Tallo, Veronica; Baltazar, Palmera I; Lesiguez, Jenny Lind S; Estanislao, Georgette G; and al | Efficacy and safety of praziquantel for the treatment of human schistosomiasis during pregnancy: a phase 2, randomised, double-blind, placebo-controlled trial | Irrelevant results |
| 548 | 2016 | O’Donnell, Melissa; Maclean, Miriam; Sims, Scott; Brownell, Marni; Ekuma, Okechukwu; Gilbert, Ruth | Entering out-of-home care during childhood: Cumulative incidence study in Canada and Australia | Irrelevant results |
| 549 | 2007 | Breeze, Andrew C. G.; Lees, Christoph C. | Prediction and perinatal outcomes of fetal growth restriction | Irrelevant results |
| 550 | 2015 | Salvador, Sérgio; Henriques, João Carlos; Munguambe, Missael; Vaz, Rui M. C.; Barros, Henrique P. | Challenges in the Management of Hydrocephalic Children in Northern Mozambique | Irrelevant results |
| 551 | 2010 | Rogers, Dennie T.; Molokie, Robert | Sickle Cell Disease in Pregnancy | Irrelevant results |
| 552 | 2019 | Kshatriya, Gautam K.; Acharya, Subhendu K. | Prevalence and risks of hypertension among Indian tribes and its status among the lean and underweight individuals | Irrelevant results |
| 553 | 2001 | Robinson, Julian N.; Regan, Joan A.; Norwitz, Errol R. | The epidemiology of preterm labor | Irrelevant results |
| 554 | 2006 | Hill, Jenny; Lines, Jo; Rowland, Mark | Insecticide-Treated Nets | Irrelevant results |
| 555 | 2021 | Yadav, Jeetendra; Nilima, Nilima | Geographic variation and factors associated with anemia among under-fives in India: A multilevel approach | Irrelevant results |
| 556 | 2016 | Husarova, V.; Donnelly, G.; Doolan, A.; Garstka, M.; Ni Ainle, F.; McCaul, C. | Preferences of Jehovah’s Witnesses regarding haematological supports in an obstetric setting: experience of a single university teaching hospital | Irrelevant results |
| 557 | 2003 | Cusson, Regina M. | Factors Influencing Language Development in Preterm Infants | Irrelevant results |
| 558 | 2017 | Oyston, Charlotte; Rueda-Clausen, Christian F.; Baker, Philip N. | Current challenges in pregnancy-related mortality | Irrelevant results |
| 559 | 2018 | Masoomi, Reza; Shah, Zubair; Arany, Zoltan; Gupta, Kamal | Peripartum cardiomyopathy: An epidemiologic study of early and late presentations | Irrelevant results |
| 560 | 2008 | Saggar, Anand K.; Bittles, Alan H. | Consanguinity and child health | Irrelevant results |
| 561 | 2021 | Atyeo, Caroline; Pullen, Krista M.; Bordt, Evan A.; Fischinger, Stephanie; Burke, John; Michell, Ashlin; and al | Compromised SARS-CoV-2-specific placental antibody transfer | Irrelevant results |
| 562 | 2014 | McCuskee, Sarah; Brickley, Elizabeth B.; Wood, Angela; Mossialos, Elias | Malaria and Macronutrient Deficiency as Correlates of Anemia in Young Children: A Systematic Review of Observational Studies | Irrelevant results |
| 563 | 2012 | Ogu, Rosemary; Okonofua, Friday; Hammed, Afolabi; Okpokunu, Edoja; Mairiga, Abdulkarim; Bako, Abubakar; Abass, Tajudeen; Garba, Danjuma; Alani, Akinyade; Agholor, Kingsley | Outcome of an intervention to improve the quality of private sector provision of postabortion care in northern Nigeria | Irrelevant results |
| 564 | 2020 | Paoletti, Monica; Raffler, Gabriele; Gaffi, Maria Sole; Antounians, Lina; Lauriti, Giuseppe; Zani, Augusto | Prevalence and risk factors for congenital diaphragmatic hernia: A global view | Irrelevant results |
| 565 | 2021 | Kay, Vanessa R.; Wedel, Naomi; Smith, Graeme N. | Family History of Hypertension, Cardiovascular Disease, or Diabetes and Risk of Developing Preeclampsia: A Systematic Review | Irrelevant results |
| 566 | 2021 | Anderson, Cassandra; Li, Helen; Cheboiwo, Vivian; Fisher, Sarah; Chepkemoi, Eunice; Rutto, Emmy; Carpenter, Kyle; Keung, Connie; Saula, Peter; Gray, Brian | Uncomplicated Gastroschisis Care in the US and Kenya: Treatment at Two Tertiary Care Centers | Irrelevant results |
| 567 | 2016 | Kotwal, Atul | Iron deficiency anaemia among children in South East Asia: Determinants, importance, prevention and control strategies | Irrelevant results |
| 568 | 2021 | Reichman, Vicky; Brachio, Sandhya S.; Madu, Chinonyerem R.; Montoya-Williams, Diana; Peña, Michelle-Marie | Using rising tides to lift all boats: Equity-focused quality improvement as a tool to reduce neonatal health disparities | Irrelevant results |
| 569 | 2019 | Kerpen, Kate; Koutrolou-Sotiropoulou, Paraskevi; Zhu, Chencan; Yang, Jie; Lyon, Jennifer-A.; Lima, Fabio V.; Stergiopoulos, Kathleen | Disparities in death rates in women with peripartum cardiomyopathy between advanced and developing countries: A systematic review and meta-analysis | Irrelevant results |
| 570 | 2001 | Lugina, Helen I; Christensson, Kyllike; Massawe, Siriel; Nystrom, Lennarth; Lindmark, Gunilla | Change in maternal concerns during the 6 weeks postpartum period: a study of primiparous mothers in Dar Es Salaam, Tanzania | Irrelevant results |
| 571 | 2019 | Malin, Gemma L.; Wallace, Suzanne VF. | Cardiac disease in pregnancy | Irrelevant results |
| 572 | 2017 | Song, Ye Kyung; Nunez Lopez, Omar; Mehta, Hemalkumar B.; Bohanon, Fredrick J.; Rojas-Khalil, Yesenia; Bowen-Jallow, Kanika A.; Radhakrishnan, Ravi S. | Race and outcomes in gastroschisis repair: a nationwide analysis | Irrelevant results |
| 573 | 2016 | Cortese, Francesca; Scicchitano, Pietro; Gesualdo, Michele; Filaninno, Antonella; De Giorgi, Elsa; Schettini, Federico; Laforgia, Nicola; Ciccone, Marco Matteo | Early and Late Infections in Newborns: Where Do We Stand? A Review | Irrelevant results |
| 574 | 2009 | Elders, M. Joycelyn | Role of Endocrinologists In Eliminating Health Care Disparities | Irrelevant results |
| 575 | 2019 | Bellver, José; Mariani, Giulia | Impact of parental over- and underweight on the health of offspring | Irrelevant results |
| 576 | 2020 | Jajoo, Namrata S.; Shelke, Anup U.; Bajaj, Rajat S.; Patil, Priyanka P.; Patil, Manali A. | Association of periodontitis with pre term low birth weight – A review | Irrelevant results |
| 577 | 2020 | Subramanyam, Rajeev; Tapia, Ignacio E.; Zhang, Bingqing; Mensinger, Janell L.; Garcia-Marcinkiewicz, Annery; Jablonka, Denis H.; Gálvez, Jorge A; Arnez, Karina; Schnoll, Robert | Secondhand Smoke exposure and risk of Obstructive Sleep Apnea in Children | Irrelevant results |
| 578 | 2019 | Coleman, Amaziah T.; Teach, Stephen J.; Sheehan, William J. | Inner-City Asthma in Childhood | Irrelevant results |
| 579 | 2008 | Fuchs, Karin; Gyamfi, Cynthia | The Influence of Obstetric Practices on Late Prematurity | Irrelevant results |
| 580 | 2012 | Deak, Teresa M.; Moskovitz, Joshua B. | Hypertension and Pregnancy | Irrelevant results |
| 581 | 2003 | Kaufman, Jay S.; Dole, Nancy; Savitz, David A.; Herring, Amy H. | Modeling Community-level Effects on Preterm Birth | Irrelevant results |
| 582 | 2004 | Zareian, Z. | Hypertensive disorders of pregnancy | Irrelevant results |
| 583 | 2019 | Bellizzi, Saverio; Ali, Mohamed M.; Cleland, John | Long-Term Trends in Reproductive Behavior Among Young Women in Four Countries, 1995–2009 | Irrelevant results |
| 584 | 2017 | Sinha, Aakanksha; McRoy, Ruth G.; Berkman, Barbara; Sutherland, Melissa | Drivers of change: Examining the effects of gender equality on child nutrition | Irrelevant results |
| 585 | 2012 | Senturk, Vesile; Hanlon, Charlotte; Medhin, Girmay; Dewey, Michael; Araya, Mesfin; Alem, Atalay; Prince, Martin; Stewart, Robert | Impact of perinatal somatic and common mental disorder symptoms on functioning in Ethiopian women: The P-MaMiE population-based cohort study | Irrelevant results |
| 586 | 2004 | Grischkan, Jonathan; Storfer-Isser, Amy; Rosen, Carol L; Larkin, Emma K; Kirchner, H. Lester; South, Andrew; Wilson-Costello, Deanne C; Martin, Richard J; Redline, Susan | Variation in childhood asthma among former preterm infants | Irrelevant results |
| 587 | 2013 | Gold, Katherine J.; Spangenberg, Kathryn; Wobil, Priscilla; Schwenk, Thomas L. | Depression and risk factors for depression among mothers of sick infants in Kumasi, Ghana | Irrelevant results |
| 588 | 2020 | Testa, Domenico; DI Bari, Matteo; Nunziata, Michele; Cristofaro, Generoso DE.; Massaro, Giuseppe; Marcuccio, Giuseppina; Motta, Gaetano | Allergic rhinitis and asthma assessment of risk factors in pediatric patients: A systematic review | Irrelevant results |
| 589 | 2011 | Coghill, Anna E.; Hansen, Susanne; Littman, Alyson J. | Risk factors for eclampsia: a population-based study in Washington State, 1987–2007 | Irrelevant results |
| 590 | 2014 | Theodoratou, Evropi; McAllister, David A; Reed, Craig; Adeloye, Davies O; Rudan, Igor; Muhe, Lulu M; Madhi, Shabir A; Campbell, Harry; Nair, Harish | Global, regional, and national estimates of pneumonia burden in HIV-infected children in 2010: a meta-analysis and modelling study | Irrelevant results |
| 591 | 2011 | Alio, Amina P.; Mbah, Alfred K.; Grunsten, Ryan A.; Salihu, Hamisu M. | Teenage Pregnancy and the Influence of Paternal Involvement on Fetal Outcomes | Irrelevant results |
| 592 | 2018 | Habib, Abdulrazaq G.; Brown, Nicholas I. | The snakebite problem and antivenom crisis from a health-economic perspective | Irrelevant results |
| 593 | 2008 | Moore, Lisa E. | Recurrent Risk of Adverse Pregnancy Outcome | Irrelevant results |
| 594 | 2021 | Shattuck, Eric C. | Networks, cultures, and institutions: Toward a social immunology | Irrelevant results |
| 595 | 2013 | Soofi, Sajid; Cousens, Simon; Iqbal, Saleem P; Akhund, Tauseef; Khan, Javed; Ahmed, Imran; Zaidi, Anita KM; Bhutta, Zulfiqar A | Effect of provision of daily zinc and iron with several micronutrients on growth and morbidity among young children in Pakistan: a cluster-randomised trial | Irrelevant results |
| 596 | 2011 | Firoz, Tabassum; Sanghvi, Harshad; Merialdi, Mario; von Dadelszen, Peter | Pre-eclampsia in low and middle income countries | Irrelevant results |
| 597 | 2012 | Langie, S. A. S.; Lara, J.; Mathers, J. C. | Early determinants of the ageing trajectory | Irrelevant results |
| 598 | 2018 | Di Renzo, Gian Carlo; Tosto, Valentina; Giardina, Irene | The biological basis and prevention of preterm birth | Irrelevant results |
| 599 | 2003 | Chervin, Ronald D.; Clarke, Dave F.; Huffman, Jennifer L.; Szymanski, Erica; Ruzicka, Deborah L.; Miller, Vnona; Nettles, Arie L.; Sowers, MaryFran R.; Giordani, Bruno J. | School performance, race, and other correlates of sleep-disordered breathing in children | Irrelevant results |
| 600 | 2011 | Brown, Alan S. | The environment and susceptibility to schizophrenia | Irrelevant results |
| 601 | 2009 | Flax, Valerie L.; Thakwalakwa, Chrissie; Phuka, John; Ashorn, Ulla; Cheung, Yin Bun; Maleta, Kenneth; Ashorn, Per | Malawian mothers’ attitudes towards the use of two supplementary foods for moderately malnourished children | Irrelevant results |
| 602 | 2004 | Rogo, K. | Improving technologies to reduce abortion-related morbidity and mortality | Irrelevant results |
| 603 | 2021 | Glatthorn, Haley N.; Sauer, Mark V.; Brandt, Justin S.; Ananth, Cande V. | Infertility treatment and the risk of small for gestational age births: a population-based study in the United States | Irrelevant results |
| 604 | 2021 | Salem, Yasmin; Oestreich, Marc-Alexander; Fuchs, Oliver; Usemann, Jakob; Frey, Urs; Surbek, Daniel; Amylidi-Mohr, Sofia; Latzin, Philipp; Ramsey, Kathryn; Yammine, Sophie | Are children born by cesarean delivery at higher risk for respiratory sequelae? | Irrelevant results |
| 605 | 2021 | Adair, Linda S.; Carba, Delia B.; Lee, Nanette R.; Borja, Judith B. | Stunting, IQ, and final school attainment in the Cebu Longitudinal Health and Nutrition Survey birth cohort | Irrelevant results |
| 606 | 2009 | McCowan, Lesley; Horgan, Richard P. | Risk factors for small for gestational age infants | Irrelevant results |
| 607 | 2016 | Petherick, Emily S.; Pearce, Neil; Sunyer, Jordi; Wright, John | Ethnic and socio-economic differences in the prevalence of wheeze, severe wheeze, asthma, eczema and medication usage at 4 years of age: Findings from the Born in Bradford birth cohort | Irrelevant results |
| 608 | 2020 | Muche, Achenef Asmamaw; Olayemi, Oladapo O.; Gete, Yigzaw Kebede | Gestational diabetes mellitus increased the risk of adverse neonatal outcomes: A prospective cohort study in Northwest Ethiopia | Irrelevant results |
| 609 | 2021 | Dooley, Leanne M.; Ahmad, Tarek B.; Pandey, Manisha; Good, Michael F.; Kotiw, Michael | Rheumatic heart disease: A review of the current status of global research activity | Irrelevant results |
| 610 | 2014 | Oyston, Charlotte; Rueda-Clausen, Christian F.; Baker, Philip N. | Current challenges in pregnancy-related mortality | Irrelevant results |
| 611 | 2017 | Lewis, Kimberly A.; Brown, Sharon A.; Tiziani, Stefano; Carrasco, Ruy | Sociocultural Considerations in Juvenile Arthritis: A Review | Irrelevant results |
| 612 | 2014 | Stein, Alan; Pearson, Rebecca M; Goodman, Sherryl H; Rapa, Elizabeth; Rahman, Atif; McCallum, Meaghan; Howard, Louise M; Pariante, Carmine M | Effects of perinatal mental disorders on the fetus and child | Irrelevant results |
| 613 | 2003 | Richter, Joachim | The impact of chemotherapy on morbidity due to schistosomiasis | Irrelevant results |
| 614 | 2015 | Mehta, Puja K.; Wei, Janet; Wenger, Nanette K. | Ischemic heart disease in women: A focus on risk factors | Irrelevant results |
| 615 | 2007 | Friedman, Jennifer F.; Mital, Priya; Kanzaria, Hemal K.; Olds, G. Richard; Kurtis, Jonathan D. | Schistosomiasis and pregnancy | Irrelevant results |
| 616 | 2018 | Pennington, Andy; Orton, Lois; Nayak, Shilpa; Ring, Adele; Petticrew, Mark; Sowden, Amanda; White, Martin; Whitehead, Margaret | The health impacts of women's low control in their living environment: A theory-based systematic review of observational studies in societies with profound gender discrimination | Irrelevant results |
| 617 | 2015 | Kramer, Christine V.; Allen, Stephen | Malnutrition in developing countries | Irrelevant results |
| 618 | 2012 | Paulson, James F.; Chauhan, Suneet P.; Hill, James B.; Abuhamad, Alfred Z. | Severe small size for gestational age and cognitive function: catch-up phenomenon possible | Irrelevant results |
| 619 | 2017 | Nabwera, Helen M; Fulford, Anthony J; Moore, Sophie E; Prentice, Andrew M | Growth faltering in rural Gambian children after four decades of interventions: a retrospective cohort study | Irrelevant results |
| 620 | 2021 | Sun, Jiahong; Wu, Han; Zhao, Min; Magnussen, Costan G.; Xi, Bo | Prevalence and changes of anemia among young children and women in 47 low- and middle-income countries, 2000-2018 | Irrelevant results |
| 621 | 2020 | Saito, Makoto; Briand, Valérie; Min, Aung Myat; McGready, Rose | Deleterious effects of malaria in pregnancy on the developing fetus: a review on prevention and treatment with antimalarial drugs | Irrelevant results |
| 622 | 2019 | Ware, Lisa J.; Prioreschi, Alessandra; Bosire, Edna; Cohen, Emmanuel; Draper, Catherine E.; Lye, Stephen J.; Norris, Shane A. | Environmental, Social, and Structural Constraints for Health Behavior: Perceptions of Young Urban Black Women During the Preconception Period—A Healthy Life Trajectories Initiative | Irrelevant results |
| 623 | 2020 | Fenta, Haile Mekonnen; Workie, Demeke Lakew; Zike, Dereje Tesfaye; Taye, Belaynew Wassie; Swain, Prafulla Kumar | Determinants of stunting among under-five years children in Ethiopia from the 2016 Ethiopia demographic and Health Survey: Application of ordinal logistic regression model using complex sampling designs | Irrelevant results |
| 624 | 2002 | Rungreangkulkij, Somporn; Chafetz, Linda; Chesla, Catherine; Gilliss, Catherine | Psychological morbidity of Thai families of a person with schizophrenia | Irrelevant results |
| 625 | 2018 | Attah, Caleb Joseph; Oguche, Stephen; Egah, Daniel; Ishaya, Tokkit Nandi; Banwat, Mathilda; Adgidzi, Adgidzi Godwin | Risk factors associated with paediatric tuberculosis in an endemic setting | Irrelevant results |
| 626 | 2015 | Hlimi, Tina | Association of anemia, pre-eclampsia and eclampsia with seasonality: A realist systematic review | Irrelevant results |
| 627 | 2010 | Kidman, Rachel; Hanley, James A.; Subramanian, S. V.; Foster, Geoff; Heymann, Jody | AIDS in the family and community: The impact on child health in Malawi | Irrelevant results |
| 628 | 2021 | Moodley, Amaran; Payton, Kurlen S. E. | The Term Newborn: Congenital Infections | Irrelevant results |
| 629 | 2011 | Saxton, Katherine B.; John-Henderson, Neha; Reid, Matthew W.; Francis, Darlene D. | The social environment and IL-6 in rats and humans | Irrelevant results |
| 630 | 2013 | Sillah, Famara; Ho, Hsin-Jung; Chao, Jane C-J. | The use of oral rehydration salt in managing children under 5 y old with diarrhea in the Gambia: Knowledge, attitude, and practice | Irrelevant results |
| 631 | 2015 | Hameed, Afshan B.; Lawton, Elizabeth S.; McCain, Christy L.; Morton, Christine H.; Mitchell, Connie; Main, Elliott K.; Foster, Elyse | Pregnancy-related cardiovascular deaths in California: beyond peripartum cardiomyopathy | Irrelevant results |
| 632 | 2020 | Church, James A.; Chasekwa, Bernard; Rukobo, Sandra; Govha, Margaret; Lee, Benjamin; Carmolli, Marya P.; and al | Predictors of oral rotavirus vaccine immunogenicity in rural Zimbabwean infants | Irrelevant results |
| 633 | 2017 | Hanson, Mark; Barker, Mary; Dodd, Jodie M; Kumanyika, Shiriki; Norris, Shane; Steegers, Eric; Stephenson, Judith; Thangaratinam, Shakila; Yang, Huixia | Interventions to prevent maternal obesity before conception, during pregnancy, and post partum | Irrelevant results |
| 634 | 2018 | Sutton, Amelia L. M.; Harper, Lorie M.; Tita, Alan T. N. | Hypertensive Disorders in Pregnancy | Irrelevant results |
| 635 | 2015 | Singla, Daisy R; Kumbakumba, Elias; Aboud, Frances E | Effects of a parenting intervention to address maternal psychological wellbeing and child development and growth in rural Uganda: a community-based, cluster-randomised trial | Irrelevant results |
| 636 | 2022 | Sun, Yi; Li, Xia; Benmarhnia, Tarik; Chen, Jiu-Chiuan; Avila, Chantal; Sacks, David A.; Chiu, Vicki; Slezak, Jeff; Molitor, John; Getahun, Darios; Wu, Jun | Exposure to air pollutant mixture and gestational diabetes mellitus in Southern California: Results from electronic health record data of a large pregnancy cohort | Irrelevant results |
| 637 | 2020 | Azmeh, Roua; Greydanus, Donald E.; Agana, Marisha G.; Dickson, Cheryl A.; Patel, Dilip R.; Ischander, Mariam M.; Lloyd, Robert D. | Update in Pediatric Asthma: Selected Issues | Irrelevant results |
| 638 | 2013 | Kramer, Michael S.; Kahn, Susan R.; Dahhou, Mourad; Otvos, James; Genest, Jacques; Platt, Robert W.; Evans, Rhobert W. | Maternal Lipids and Small for Gestational Age Birth at Term | Irrelevant results |
| 639 | 2009 | Klein, Nicola P.; Kissner, Jennifer; Aguirre, Ameth; Sparks, Robert; Campbell, Scott; Edwards, Kathryn M.; Dekker, Cornelia L.; Shui, Irene; Gust, Deborah A. | Differential maternal responses to a newly developed vaccine information pamphlet | Irrelevant results |
| 640 | 2021 | Naicker, Sara N.; Norris, Shane A.; Richter, Linda M. | Secondary analysis of retrospective and prospective reports of adverse childhood experiences and mental health in young adulthood: Filtered through recent stressors | Irrelevant results |
| 641 | 2019 | Adepoju, Akinlolu A.; Allen, Stephen | Malnutrition in developing countries: nutrition disorders, a leading cause of ill health in the world today | Irrelevant results |
| 642 | 2017 | Keller, Roberta L.; Feng, Rui; DeMauro, Sara B.; Ferkol, Thomas; Hardie, William; Rogers, Elizabeth E.; and al | Bronchopulmonary Dysplasia and Perinatal Characteristics Predict 1-Year Respiratory Outcomes in Newborns Born at Extremely Low Gestational Age: A Prospective Cohort Study | Irrelevant results |
| 643 | 2010 | Cumberland, Phillippa M.; Pathai, Sophia; Rahi, Jugnoo S. | Prevalence of Eye Disease in Early Childhood and Associated Factors: Findings from the Millennium Cohort Study | Irrelevant results |
| 644 | 2018 | Prophet, Javon; Kelly, Kalifa; Domingo, Julian; Ayeni, Helen; Mekouguem, Xaviera Pascale Djoko; Dockery, Breana; Allam, Farida; Kaur, Manvir; Artis, Javon; Spooner, Kiara K.; Salemi, Jason L.; Olaleye, Omonike A.; Salihu, Hamisu M. | Severe pre-eclampsia among pregnant women with sickle cell disease and HIV | Irrelevant results |
| 645 | 2015 | James, Scott H.; Kimberlin, David W. | Neonatal Herpes Simplex Virus Infection | Irrelevant results |
| 646 | 2010 | Zaman, K; Anh, Dang Duc; Victor, John C; Shin, Sunheang; Yunus, Md; Dallas, Michael J; and al | Efficacy of pentavalent rotavirus vaccine against severe rotavirus gastroenteritis in infants in developing countries in Asia: a randomised, double-blind, placebo-controlled trial | Irrelevant results |
| 647 | 2012 | Mukhopadhyay, Sagori; Puopolo, Karen M. | Risk Assessment in Neonatal Early Onset Sepsis | Irrelevant results |
| 648 | 2009 | Canino, Glorisa; McQuaid, Elizabeth L.; Rand, Cynthia S. | Addressing asthma health disparities: A multilevel challenge | Irrelevant results |
| 649 | 2013 | Alhusen, Jeanne L.; Lucea, Marguerite B.; Bullock, Linda; Sharps, Phyllis | Intimate Partner Violence, Substance Use, and Adverse Neonatal Outcomes among Urban Women | Irrelevant results |
| 650 | 2005 | Heinzer, Marjorie M. | Obesity in Infancy: Questions, More Questions, and Few Answers | Irrelevant results |
| 651 | 2018 | Manuck, Tracy A.; Fry, Rebecca C.; McFarlin, Barbara L. | Quality Improvement in Perinatal Medicine and Translation of Preterm Birth Research Findings into Clinical Care | Irrelevant results |
| 652 | 2018 | Wimalawansa, Sunil J. | Non-musculoskeletal benefits of vitamin D | Irrelevant results |
| 653 | 2001 | Letamo, Gobopamang; Majelantle, Rolang G | Health implications of early childbearing on pregnancy outcome in Botswana: insights from the institutional records | Irrelevant results |
| 654 | 2018 | Piccoli, Giorgina B.; Alrukhaimi, Mona; Liu, Zhi-Hong; Zakharova, Elena; Levin, Adeera | What we do and do not know about women and kidney diseases; questions unanswered and answers unquestioned: Reflection on World Kidney Day and International Woman's Day | Irrelevant results |
| 655 | 2016 | Ewing, Alexander C.; Datwani, Hema M.; Flowers, Lisa M.; Ellington, Sascha R.; Jamieson, Denise J.; Kourtis, Athena P. | Trends in hospitalizations of pregnant HIV-infected women in the United States: 2004 through 2011 | Irrelevant results |
| 656 | 2018 | Koye, Digsu N.; Magliano, Dianna J.; Nelson, Robert G.; Pavkov, Meda E. | The Global Epidemiology of Diabetes and Kidney Disease | Irrelevant results |
| 657 | 2006 | Rodrigues, Amabelia; Fischer, Thea K.; Valentiner-Branth, Palle; Nielsen, Jens; Steinsland, Hans; Perch, Michael; Garly, May-Lill; Mølbak, Kåre; Aaby, Peter | Community cohort study of rotavirus and other enteropathogens: Are routine vaccinations associated with sex-differential incidence rates? | Irrelevant results |
| 658 | 2006 | Cleland, John; Bernstein, Stan; Ezeh, Alex; Faundes, Anibal; Glasier, Anna; Innis, Jolene | Family planning: the unfinished agenda | Irrelevant results |
| 659 | 2008 | Ekman, Björn; Pathmanathan, Indra; Liljestrand, Jerker | Integrating health interventions for women, newborn babies, and children: a framework for action | Irrelevant results |
| 660 | 2018 | Theilen, Lauren H.; Meeks, Huong; Fraser, Alison; Esplin, M. Sean; Smith, Ken R.; Varner, Michael W. | Long-term mortality risk and life expectancy following recurrent hypertensive disease of pregnancy | Irrelevant results |
| 661 | 2014 | Howard, Louise M; Molyneaux, Emma; Dennis, Cindy-Lee; Rochat, Tamsen; Stein, Alan; Milgrom, Jeannette | Non-psychotic mental disorders in the perinatal period | Irrelevant results |
| 662 | 2021 | Kang, Yunhee; Cho, Mokryeon; Rahman, Md. Mezanur; Cho, Yoonho; Han, Seungheon; Dutta, Makhan L. | Design of a collaborative monitoring and evaluation system for a community-based nutrition project in rural Bangladesh | Irrelevant results |
| 663 | 2015 | Long, Chao; Titus Ngwa Tagang, Ebogo; Popat, Rita A.; Lawong, Ernest K.; Brown, James A.; Wren, Sherry M. | Factors associated with delays to surgical presentation in North-West Cameroon | Irrelevant results |
| 664 | 2019 | Stergiopoulos, Kathleen; Lima, Fabio V. | Peripartum cardiomyopathy-diagnosis, management, and long term implications | Irrelevant results |
| 665 | 2007 | Carter-Pokras, Olivia; Zambrana, Ruth E.; Poppell, Carolyn F.; Logie, Laura A.; Guerrero-Preston, Rafael | The Environmental Health of Latino Children | Irrelevant results |
| 666 | 2002 | Griffiths, Paula; Matthews, Zoë; Hinde, Andrew | Gender, family, and the nutritional status of children in three culturally contrasting states of India | Irrelevant results |
| 667 | 2013 | Qadri, Firdausi; Bhuiyan, Taufiqur Rahman; Sack, David A.; Svennerholm, Ann-Mari | Immune responses and protection in children in developing countries induced by oral vaccines | Irrelevant results |
| 668 | 2018 | Fanzo, Jessica; Davis, Claire; McLaren, Rebecca; Choufani, Jowel | The effect of climate change across food systems: Implications for nutrition outcomes | Irrelevant results |
| 669 | 2017 | Wagijo, Mary-ann; Sheikh, Aziz; Duijts, Liesbeth; Been, Jasper V. | Reducing tobacco smoking and smoke exposure to prevent preterm birth and its complications | Irrelevant results |
| 670 | 2021 | Ansah, Edward W.; Ankomah-Appiah, Emmanuel; Amoadu, Mustapha; Sarfo, Jacob O. | Climate change, health and safety of workers in developing economies: A scoping review | Irrelevant results |
| 671 | 2018 | Piccoli, Giorgina B.; Alrukhaimi, Mona; Liu, Zhi-Hong; Zakharova, Elena; Levin, Adeera | What we do and do not know about women and kidney diseases; questions unanswered and answers unquestioned: Reflection on World Kidney Day and International Woman's Day | Irrelevant results |
| 672 | 2018 | Aldridge, Robert W; Nellums, Laura B; Bartlett, Sean; Barr, Anna Louise; Patel, Parth; Burns, Rachel; Hargreaves, Sally; Miranda, J Jaime; Tollman, Stephen; Friedland, Jon S; Abubakar, Ibrahim | Global patterns of mortality in international migrants: a systematic review and meta-analysis | Irrelevant results |
| 673 | 2006 | Kothari, Anjali; Mahadevan, Neila; Girling, Joanna | Tuberculosis and pregnancy—Results of a study in a high prevalence area in London | Irrelevant results |
| 674 | 2020 | Givens, D. I. | MILK Symposium review: The importance of milk and dairy foods in the diets of infants, adolescents, pregnant women, adults, and the elderly* | Irrelevant results |
| 675 | 2020 | Weaver, Rupert; Nguyen, Cattram D.; Chan, Jocelyn; Vilivong, Keoudomphone; Lai, Jana Y. R.; Lim, Ruth; and al | The effectiveness of the 13-valent pneumococcal conjugate vaccine against hypoxic pneumonia in children in Lao People's Democratic Republic: An observational hospital-based test-negative study | Irrelevant results |
| 676 | 2020 | Baetzel, Anne Elizabeth; Holman, Ashlee; Dobija, Nicole; Reynolds, Paul Irvin; Nafiu, Olubukola Olugbenga | Racial Disparities in Pediatric Anesthesia | Irrelevant results |
| 677 | 2016 | da Matta Felisberto Fernandes, Maria Luiza; Kawachi, Ichiro; Fernandes, Alexandre Moreira; Corrêa-Faria, Patrícia; Paiva, Saul Martins; Pordeus, Isabela Almeida | Oral health-related quality of life of children and teens with sickle cell disease | Irrelevant results |
| 678 | 2019 | Charuvila, Somy; Davidson, Sarah E; Thachil, Jecko; Lakhoo, Kokila | Surgical decision making around paediatric preoperative anaemia in low-income and middle-income countries | Irrelevant results |
| 679 | 2012 | Villegas, Susy; Pecora, Peter J. | Mental health outcomes for adults in family foster care as children: An analysis by ethnicity | Irrelevant results |
| 680 | 2018 | Vikram, Kriti | Social capital and child nutrition in India: The moderating role of development | Irrelevant results |
| 681 | 2013 | Richards, Esther; Theobald, Sally; George, Asha; Kim, Julia C.; Rudert, Christiane; Jehan, Kate; Tolhurst, Rachel | Going beyond the surface: Gendered intra-household bargaining as a social determinant of child health and nutrition in low and middle income countries | Irrelevant results |
| 682 | 2006 | Grady, Sue C. | Racial disparities in low birthweight and the contribution of residential segregation: A multilevel analysis | Irrelevant results |
| 683 | 2008 | Handa, Rohini; Ali Kalla, Asgar; Maalouf, Ghassan | Osteoporosis in developing countries | Irrelevant results |
| 684 | 2014 | Rosenberg, Stacy L.; Miller, Gregory E.; Brehm, John M.; Celedón, Juan C. | Stress and asthma: Novel insights on genetic, epigenetic, and immunologic mechanisms | Irrelevant results |
| 685 | 2017 | Yaméogo, Charles W.; Cichon, Bernardette; Fabiansen, Christian; Iuel-Brockdorf, Ann-Sophie; Shepherd, Susan; Filteau, Suzanne; and al | Correlates of Physical Activity among Young Children with Moderate Acute Malnutrition | Irrelevant results |
| 686 | 2012 | Szefler, Stanley J. | Advances in pediatric asthma in 2011: Moving forward | Irrelevant results |
| 687 | 2018 | Sparks, Teresa N.; Caughey, Aaron B. | How should costs and cost-effectiveness be considered in prenatal genetic testing? | Irrelevant results |
| 688 | 2008 | Zimmermann, Michael B; Jooste, Pieter L; Pandav, Chandrakant S | Iodine-deficiency disorders | Irrelevant results |
| 689 | 2012 | Ashford, Kristin; Westneat, Susan | Prenatal Hair Nicotine Analysis in Homes with Multiple Smokers | Irrelevant results |
| 690 | 2016 | Althoff, Robert R.; Ametti, Merelise; Bertmann, Farryl | The role of food insecurity in developmental psychopathology | Irrelevant results |
| 691 | 2009 | Smaldone, Arlene; Honig, Judy C.; Byrne, Mary W. | Does Assessing Sleep Inadequacy Across Its Continuum Inform Associations With Child and Family Health? | Irrelevant results |
| 692 | 2019 | Swope, Carolyn B.; Hernández, Diana | Housing as a determinant of health equity: A conceptual model | Irrelevant results |
| 693 | 2021 | Tinajero, Maria G.; Malik, Vasanti S. | An Update on the Epidemiology of Type 2 Diabetes: A Global Perspective | Irrelevant results |
| 694 | 2019 | Yamashiro, Kaeli J.; Galganski, Laura A.; Hirose, Shinjiro | Fetal myelomeningocele repair | Irrelevant results |
| 695 | 2016 | Al-Ghannami, Samia S.; Sedlak, Eva; Hussein, Izzeldin S.; Min, Yoeju; Al-Shmmkhi, Saleh M.; Al-Oufi, Hamed S.; Al-Mazroui, Ahmed; Ghebremeskel, Kebreab | Lipid-soluble nutrient status of healthy Omani school children before and after intervention with oily fish meal or re-esterified triacylglycerol fish oil | Irrelevant results |
| 696 | 2015 | Fletcher, Teresa M.; Markley, Laura A.; Nelson, Dana; Crane, Stephen S.; Fitzgibbon, James J. | Pregnant Adolescents Admitted to an Inpatient Child and Adolescent Psychiatric Unit: An Eight-Year Review | Irrelevant results |
| 697 | 2016 | Lain, Samantha J; Bentley, Jason P; Wiley, Veronica; Roberts, Christine L; Jack, Michelle; Wilcken, Bridget; Nassar, Natasha | Association between borderline neonatal thyroid-stimulating hormone concentrations and educational and developmental outcomes: a population-based record-linkage study | Irrelevant results |
| 698 | 2009 | Tolle, Michael A. | Mosquito-borne Diseases | Irrelevant results |
| 699 | 2007 | Enriquez, Maithe; Farnan, Rose; Simpson, Kathryn; Grantello, Sandra; Miles, Margaret Shandor | Pregnancy, Poverty, and HIV | Irrelevant results |
| 700 | 2021 | Kleinitz, Pauline; Sabariego, Carla; Cieza, Alarcos | Development of the WHO STARS: A Tool for the Systematic Assessment of Rehabilitation Situation | Irrelevant results |
| 701 | 2020 | Webb, Glynn W.; Kelly, Sophie; Dalton, Harry R. | Hepatitis A and Hepatitis E: Clinical and Epidemiological Features, Diagnosis, Treatment, and Prevention | Irrelevant results |
| 702 | 2021 | Rohan, Jennifer M.; Winter, Marcia A. | Ethical considerations in pediatric chronic illness: The relationship between psychological factors, treatment adherence, and health outcomes | Irrelevant results |
| 703 | 2019 | Vincent, Leah R.; Jerse, Ann E. | Biological feasibility and importance of a gonorrhea vaccine for global public health | Irrelevant results |
| 704 | 2005 | Bonds, Duane Robina | Three decades of innovation in the management of sickle cell disease: the road to understanding the sickle cell disease clinical phenotype | Irrelevant results |
| 705 | 2017 | Tolcher, Mary Catherine; Chu, Derrick M.; Hollier, Lisa M.; Mastrobattista, Joan M.; Racusin, Diana A.; Ramin, Susan M.; Sangi-Haghpeykar, Haleh; Aagaard, Kjersti M. | Impact of USPSTF recommendations for aspirin for prevention of recurrent preeclampsia | Irrelevant results |
| 706 | 2016 | Lumsden, Rebecca H.; Akwanalo, Constantine; Chepkwony, Stella; Kithei, Anne; Omollo, Vincent; Holland, Thomas L.; Bloomfield, Gerald S.; O'Meara, Wendy P. | Clinical and geographic patterns of rheumatic heart disease in outpatients attending cardiology clinic in western Kenya | Irrelevant results |
| 707 | 2011 | Beydoun, Hind; Ugwu, Bethrand; Oehninger, Sergio | Assisted reproduction for the validation of gestational age assessment methods | Irrelevant results |
| 708 | 2012 | Fotso, Jean Christophe; Madise, Nyovani; Baschieri, Angela; Cleland, John; Zulu, Eliya; Kavao Mutua, Martin; Essendi, Hildah | Child growth in urban deprived settings: Does household poverty status matter? At which stage of child development? | Irrelevant results |
| 709 | 2019 | Bernardes, Thomas P.; Mol, Ben W.; Ravelli, Anita C. J.; van den Berg, Paul P.; Boezen, H. Marike; Groen, Henk | Recurrence risk of preeclampsia in a linked population-based cohort: Effects of first pregnancy maximum diastolic blood pressure and gestational age | Irrelevant results |
| 710 | 2016 | Haile, Zelalem T.; Teweldeberhan, Asli K.; Chertok, Ilana R. A. | Association between oral contraceptive use and markers of iron deficiency in a cross-sectional study of Tanzanian women | Irrelevant results |
| 711 | 2021 | Buttermore, Emily; Campanella, Veronica; Priefer, Ronny | The increasing trend of Type 2 diabetes in youth: An overview | Irrelevant results |
| 712 | 2016 | Ho, Alison; Flynn, Angela C.; Pasupathy, Dharmintra | Nutrition in pregnancy | Irrelevant results |
| 713 | 2017 | Steinhoff, Mark C; Katz, Joanne; Englund, Janet A; Khatry, Subarna K; Shrestha, Laxman; Kuypers, Jane; and al | Year-round influenza immunisation during pregnancy in Nepal: a phase 4, randomised, placebo-controlled trial | Irrelevant results |
| 714 | 2017 | Candler, Toby; Costa, Silvia; Heys, Michelle; Costello, Anthony; Viner, Russell M. | Prevalence of Thinness in Adolescent Girls in Low- and Middle-Income Countries and Associations With Wealth, Food Security, and Inequality | Irrelevant results |
| 715 | 2017 | Nulu, Shanti; Bukhman, Gene; Kwan, Gene F. | Rheumatic Heart Disease: The Unfinished Global Agenda | Irrelevant results |
| 716 | 2014 | McClure, Elizabeth M.; Meshnick, Steven R.; Lazebnik, Noam; Mungai, Peter; King, Christopher L.; Hudgens, Michael; and al | A cohort study of Plasmodium falciparum malaria in pregnancy and associations with uteroplacental blood flow and fetal anthropometrics in Kenya | Irrelevant results |
| 717 | 2015 | Ashimi, Adewale O.; Amole, Taiwo G.; Iliyasu, Zubairu | Prevalence and predictors of female genital mutilation among infants in a semi urban community in northern Nigeria | Irrelevant results |
| 718 | 2021 | Alenezi, Shuliweeh; Alnamnakani, Mahdi; Temsah, Mohamad-Hani; Murshid, Rozan; Alfahad, Fahad; Alqurashi, Haitham; Alonazy, Hana; Alothman, Mohamad; Aleissa, Majid A. | Evaluation of the impact of the COVID-19 pandemic on the reporting of maltreatment cases to the National Family Safety Program in Saudi Arabia | Irrelevant results |
| 719 | 2021 | Galler, Janina R; Bringas-Vega, Maria L; Tang, Qin; Rabinowitz, Arielle G; Musa, Kamarul Imran; Chai, Wen Jia; and al | Neurodevelopmental effects of childhood malnutrition: A neuroimaging perspective | Irrelevant results |
| 720 | 2016 | Lazzerini, Marzia; Seward, Nadine; Lufesi, Norman; Banda, Rosina; Sinyeka, Sophie; Masache, Gibson; and al | Mortality and its risk factors in Malawian children admitted to hospital with clinical pneumonia, 2001–12: a retrospective observational study | Irrelevant results |
| 721 | 2014 | Yousafzai, Aisha K; Rasheed, Muneera A; Rizvi, Arjumand; Armstrong, Robert; Bhutta, Zulfiqar A | Effect of integrated responsive stimulation and nutrition interventions in the Lady Health Worker programme in Pakistan on child development, growth, and health outcomes: a cluster-randomised factorial effectiveness trial | Irrelevant results |
| 722 | 2006 | Bobetsis, Yiorgos A.; Barros, Silvana P.; Offenbacher, Steven | Exploring the relationship between periodontal disease and pregnancy complications | Irrelevant results |
| 723 | 2004 | Cho, June; Holditch-Davis, Diane; Belyea, Michael | Gender, ethnicity, and the interactions of prematurely born children and their mothers | Irrelevant results |
| 724 | 2010 | O'Keefe, Catherine | Viral Infections in the Neonate | Irrelevant results |
| 725 | 2012 | Goyal, Deepika; Wang, Elsie J.; Shen, Jeremy; Wong, Eric C.; Palaniappan, Latha P. | Clinically Identified Postpartum Depression in Asian American Mothers | Irrelevant results |
| 726 | 2021 | Xu, Zhi-Fei; Ni, Xin | Debates in pediatric obstructive sleep apnea treatment | Irrelevant results |
| 727 | 2011 | Walton, Emily; Allen, Stephen | Malnutrition in developing countries | Irrelevant results |
| 728 | 2010 | Faúndes, Anibal | Unsafe abortion – the current global scenario | Irrelevant results |
| 729 | 2020 | Hammad, Ibrahim A.; Meeks, Huong; Fraser, Alison; Theilen, Lauren H.; Esplin, M. Sean; Smith, Ken R.; Varner, Michael W. | Risks of cause-specific mortality in offspring of pregnancies complicated by hypertensive disease of pregnancy | Irrelevant results |
| 730 | 2006 | King, Cheryl A.; Knox, Michele S.; Henninger, Nathan; Nguyen, Tuan Anh; Ghaziuddin, Neera; Maker, Azmaira; Hanna, Gregory L. | Major depressive disorder in adolescents: Family psychiatric history predicts severe behavioral disinhibition | Irrelevant results |
| 731 | 2008 | Mathai, Matthews | Working with communities, governments and academic institutions to make pregnancy safer | Irrelevant results |
| 732 | 2021 | Woodside, Ashley; Bradford, Heather | Exercise and the Prevention of Gestational Diabetes Mellitus | Irrelevant results |
| 733 | 2021 | Song, Jian; Pan, Rubing; Yi, Weizhuo; Wei, Qiannan; Qin, Wei; Song, Shasha; Tang, Chao; He, Yangyang; Liu, Xiangguo; Cheng, Jian; Su, Hong | Ambient high temperature exposure and global disease burden during 1990–2019: An analysis of the Global Burden of Disease Study 2019 | Irrelevant results |
| 734 | 2008 | Cannon, Tyrone D.; Yolken, Robert; Buka, Stephen; Torrey, E. Fuller | Decreased Neurotrophic Response to Birth Hypoxia in the Etiology of Schizophrenia | Irrelevant results |
| 735 | 2012 | Sherwin, Justin C.; Reacher, Mark H.; Dean, William H.; Ngondi, Jeremiah | Epidemiology of vitamin A deficiency and xerophthalmia in at-risk populations | Irrelevant results |
| 736 | 2020 | Haldeman, Matthew S.; Nolan, Melissa S.; Ng'habi, Kija R. N. | Human hookworm infection: Is effective control possible? A review of hookworm control efforts and future directions | Irrelevant results |
| 737 | 2018 | McQuaid, Elizabeth L. | Barriers to medication adherence in asthma: The importance of culture and context | Irrelevant results |
| 738 | 2018 | Mersky, Joshua P.; Janczewski, Colleen E. | Racial and ethnic differences in the prevalence of adverse childhood experiences: Findings from a low-income sample of U.S. women | Irrelevant results |
| 739 | 2014 | Yuen, Jiasong; Painter, Ian; Abraham, Linta; Melian, Mercedes; Denno, Donna M. | A comparison of trends in cesarean delivery in Paraguay between 1995 and 2008 | Irrelevant results |
| 740 | 2020 | Scott, Georgia; Gillon, Tessa E.; Pels, Anouk; von Dadelszen, Peter; Magee, Laura A. | Guidelines—similarities and dissimilarities: a systematic review of international clinical practice guidelines for pregnancy hypertension | Irrelevant results |
| 741 | 2021 | Kelly, Sarah Dion | Diabetes Prevention: Focusing on Lifestyle and Behavior Change | Irrelevant results |
| 742 | 2017 | Rayment-Jones, Hannah; Butler, Eleanor; Miller, Chelsie; Nay, Christine; O’Dowd, Jennifer | A multisite audit to assess how women with complex social factors access and engage with maternity services | Irrelevant results |
| 743 | 2013 | Lee, Anne CC; Katz, Joanne; Blencowe, Hannah; Cousens, Simon; Kozuki, Naoko; Vogel, Joshua P; and al | National and regional estimates of term and preterm babies born small for gestational age in 138 low-income and middle-income countries in 2010 | Irrelevant results |
| 744 | 2016 | Alonge, Olakunle; Khan, Uzma R.; Hyder, Adnan A. | Our Shrinking Globe: Implications for Child Unintentional Injuries | Irrelevant results |
| 745 | 2008 | Kohler, Mark J.; van den Heuvel, Cameron J. | Is there a clear link between overweight/obesity and sleep disordered breathing in children? | Irrelevant results |
| 746 | 2010 | Jessee, Mary Ann | Stool Studies: Tried, True, and New | Irrelevant results |
| 747 | 2011 | Kawar, Nadia; Alrayyes, Sahar | Periodontitis in Pregnancy: The Risk of Preterm Labor and Low Birth Weight | Irrelevant results |
| 748 | 2015 | Murray, Christopher J L; Barber, Ryan M; Foreman, Kyle J; Ozgoren, Ayse Abbasoglu; Abd-Allah, Foad; Abera, Semaw F and al | Global, regional, and national disability-adjusted life years (DALYs) for 306 diseases and injuries and healthy life expectancy (HALE) for 188 countries, 1990–2013: quantifying the epidemiological transition | Irrelevant results |
| 749 | 2007 | Murphy, Deirdre J. | Epidemiology and environmental factors in preterm labour | Irrelevant results |
| 750 | 2017 | Lawley, Claire M.; Lain, Samantha J.; Figtree, Gemma A.; Sholler, Gary F.; Winlaw, David S.; Roberts, Christine L. | Mortality, rehospitalizations and costs in children undergoing a cardiac procedure in their first year of life in New South Wales, Australia | Irrelevant results |
| 751 | 2015 | Song, Qi-Ying; Luo, Wei-Ping; Zhang, Cai-Xia | High serum iron level is associated with an increased risk of hypertensive disorders during pregnancy: a meta-analysis of observational studies | Irrelevant results |
| 752 | 2013 | Han, Yueh-Ying; Blatter, Josh; Brehm, John M; Forno, Erick; Litonjua, Augusto A; Celedón, Juan C | Diet and asthma: vitamins and methyl donors | Irrelevant results |
| 753 | 2011 | Guyer, Amanda E.; Choate, Victoria R.; Grimm, Kevin J.; Pine, Daniel S.; Keenan, Kate | Emerging Depression Is Associated With Face Memory Deficits in Adolescent Girls | Irrelevant results |
| 754 | 2010 | Chacín-Bonilla, Leonor | Epidemiology of Cyclospora cayetanensis: A review focusing in endemic areas | Irrelevant results |
| 755 | 2008 | Pattussi, Marcos Pascoal; Lalloo, Ratilal; Bassani, Diego Garcia; Olinto, Maria Teresa Anselmo | The role of psychosocial, behavioural and emotional factors on self-reported major injuries in Brazilian adolescents: A case-control study | Irrelevant results |
| 756 | 2014 | Amegah, Adeladza Kofi; Jaakkola, Jouni J. K. | Work as a street vendor, associated traffic-related air pollution exposures and risk of adverse pregnancy outcomes in Accra, Ghana | Irrelevant results |
| 757 | 2001 | Norwitz, Errol R.; Robinson, Julian N. | A systematic approach to the management of preterm labor | Irrelevant results |
| 758 | 2006 | Garenne, Michel; Gakusi, Albert Enéas | Vulnerability and Resilience: Determinants of Under-Five Mortality Changes in Zambia | Irrelevant results |
| 759 | 2018 | Wenger, Nanette K.; Arnold, Anita; Bairey Merz, C. Noel; Cooper-DeHoff, Rhonda M.; Ferdinand, Keith C.; Fleg, Jerome L.; and al | Hypertension Across a Woman’s Life Cycle | Irrelevant results |
| 760 | 2020 | Parikh, Samir V.; Almaani, Salem; Brodsky, Sergey; Rovin, Brad H. | Update on Lupus Nephritis: Core Curriculum 2020 | Irrelevant results |
| 761 | 2019 | Forrester, Sarah; Jacobs, David; Zmora, Rachel; Schreiner, Pamela; Roger, Veronique; Kiefe, Catarina I. | Racial differences in weathering and its associations with psychosocial stress: The CARDIA study | Irrelevant results |
| 762 | 2012 | Wong, Cynthia J.; Moxey-Mims, Marva; Jerry-Fluker, Judith; Warady, Bradley A.; Furth, Susan L. | CKiD (CKD in Children) Prospective Cohort Study: A Review of Current Findings | Irrelevant results |
| 763 | 2012 | Das, Jishnu; Das, Ranendra Kumar; Das, Veena | The mental health gender-gap in urban India: Patterns and narratives | Irrelevant results |
| 764 | 2020 | Santos, Soraya Silva; de Araújo, Renan Vinicius; Giarolla, Jeanine; Seoud, Omar El; Ferreira, Elizabeth Igne | Searching for drugs for Chagas disease, leishmaniasis and schistosomiasis: a review | Irrelevant results |
| 765 | 2007 | Crittenden, Kathleen S.; Manfredi, Clara; Cho, Young I.; Dolecek, Therese A. | Smoking cessation processes in low-SES women: The impact of time-varying pregnancy status, health care messages, stress, and health concerns | Irrelevant results |
| 766 | 2019 | Hydes, Theresa; Gilmore, William; Sheron, Nick; Gilmore, Ian | Treating alcohol-related liver disease from a public health perspective | Irrelevant results |
| 767 | 2019 | Bilibio, João P.; Beltrão, Alinne M.; Vargens, Ana C.; Gama, Thiago B.; Lorenzzoni, Pânila L. | Gastroschisis during gestation: prognostic factors of neonatal mortality from prenatal care to postsurgery | Irrelevant results |
| 768 | 2021 | Fatouros, Selina; Capetola, Teresa | Examining Gendered Expectations on Women's Vulnerability to Natural Hazards in Low to Middle Income Countries: A critical Literature Review | Irrelevant results |
| 769 | 2020 | Cieza, Alarcos; Causey, Kate; Kamenov, Kaloyan; Hanson, Sarah Wulf; Chatterji, Somnath; Vos, Theo | Global estimates of the need for rehabilitation based on the Global Burden of Disease study 2019: a systematic analysis for the Global Burden of Disease Study 2019 | Irrelevant results |
| 770 | 2007 | Falagas, Matthew E.; Mourtzoukou, Eleni G.; Vardakas, Konstantinos Z. | Sex differences in the incidence and severity of respiratory tract infections | Irrelevant results |
| 771 | 2001 | Mace, Sharon E. | Asthma Therapy in the Observation Unit | Irrelevant results |
| 772 | 2009 | Hastings-Tolsma, Marie; Vincent, Deborah; Park, Jeong-Hwan; Pan, Dongmei | Treatment patterns and outcomes in a low-risk nurse-midwifery practice | Irrelevant results |
| 773 | 2016 | Weaver, Scott C.; Costa, Federico; Garcia-Blanco, Mariano A.; Ko, Albert I.; Ribeiro, Guilherme S.; Saade, George; Shi, Pei-Yong; Vasilakis, Nikos | Zika virus: History, emergence, biology, and prospects for control | Irrelevant results |
| 774 | 2019 | Yalın Sapmaz, Şermin; Şen, Semra; Özkan, Yekta; Kandemir, Hasan | Relationship between Toxoplasma gondii seropositivity and depression in children and adolescents | Irrelevant results |
| 775 | 2009 | Latendresse, Gwen | The Interaction Between Chronic Stress and Pregnancy: Preterm Birth from A Biobehavioral Perspective | Irrelevant results |
| 776 | 2012 | Abramovici, Adi; Cantu, Jessica; Jenkins, Sheri M. | Tocolytic Therapy for Acute Preterm Labor | Irrelevant results |
| 777 | 2006 | Obaro, Stephen K; Madhi, Shabir A | Bacterial pneumonia vaccines and childhood pneumonia: are we winning, refining, or redefining? | Irrelevant results |
| 778 | 2011 | Lorenz, Veronique; Karanis, Panagiotis | Malaria vaccines: looking back and lessons learnt | Irrelevant results |
| 779 | 2014 | Wenger, Nanette K. | Recognizing Pregnancy-Associated Cardiovascular Risk Factors | Irrelevant results |
| 780 | 2013 | Ernst, Kacey C.; Phillips, Beth S.; Duncan, Burris “Duke” | Slums Are Not Places for Children to Live: Vulnerabilities, Health Outcomes, and Possible Interventions | Irrelevant results |
| 781 | 2002 | Shohat, Tamy; Green, Manfred S.; Davidson, Yael; Livne, Irit; Tamir, Rami; Garty, Ben-Zion | Differences in the prevalence of asthma and current wheeze between Jews and Arabs: results from a national survey of schoolchildren in Israel | Irrelevant results |
| 782 | 2013 | Barua, Maan; Bhagwat, Shonil A.; Jadhav, Sushrut | The hidden dimensions of human–wildlife conflict: Health impacts, opportunity and transaction costs | Irrelevant results |
| 783 | 2009 | Srinivas, Sindhu K.; Sammel, Mary D.; Stamilio, David M.; Clothier, Bonnie; Jeffcoat, Marjorie K.; Parry, Samuel; Macones, George A.; Elovitz, Michal A.; Metlay, Joshua | Periodontal disease and adverse pregnancy outcomes: is there an association? | Irrelevant results |
| 784 | 2019 | Linehan, Laura A.; Morris, Aoife G.; Meaney, Sarah; O’Donoghue, Keelin | Subsequent pregnancy outcomes following second trimester miscarriage—A prospective cohort study | Irrelevant results |
| 785 | 2012 | Corbella, S.; Del Fabbro, M.; Taschieri, S.; Francetti, L. | Periodontal disease and adverse pregnancy outcomes: a systematic review | Irrelevant results |
| 786 | 2020 | Werner, Elizabeth A.; Aloisio, Cara E.; Butler, Ashlie D.; D'Antonio, Kristina M.; Kenny, Jennifer M.; Mitchell, Anika; Ona, Samsiya; Monk, Catherine | Addressing mental health in patients and providers during the COVID-19 pandemic | Irrelevant results |
| 787 | 2020 | Wolfe, Kelly R.; Liptzin, Deborah R.; Brigham, Dania; Kelly, Sarah L.; Rafferty, Carey; Albertz, Megan; Younoszai, Adel K.; Di Maria, Michael V. | Relationships between Physiologic and Neuropsychologic Functioning after Fontan | Irrelevant results |
| 788 | 2017 | dos Santos da Silva, Luciana Lourenço Gomes; Saunders, Cláudia; Campos, Aline Bull Ferreira; Belfort, Gabriella Pinto; de Carvalho Padilha, Patricia; Pereira, Rosangela Alves; Bornia, Rita Guérios | Hypertensive disorders of pregnancy in women with gestational diabetes mellitus from Rio de Janeiro, Brazil | Irrelevant results |
| 789 | 2021 | Agako, Arela; Donegan, Eleanor; McCabe, Randi E.; Frey, Benicio N.; Streiner, David; Green, Sheryl | The role of emotion dysregulation in cognitive behavioural group therapy for perinatal anxiety: Results from a randomized controlled trial and routine clinical care | Irrelevant results |
| 790 | 2017 | Faber, Timor; Kumar, Arun; Mackenbach, Johan P; Millett, Christopher; Basu, Sanjay; Sheikh, Aziz; Been, Jasper V | Effect of tobacco control policies on perinatal and child health: a systematic review and meta-analysis | Irrelevant results |
| 791 | 2001 | Paz, Ido; Laor, Arie; Gale, Rena; Harlap, Susan; Stevenson, David K.; Seidman, Daniel S. | Term infants with fetal growth restriction are not at increased risk for low intelligence scores at age 17 years | Irrelevant results |
| 792 | 2017 | Walsh, Edward E. | Respiratory Syncytial Virus Infection: An Illness for All Ages | Irrelevant results |
| 793 | 2013 | Karaye, Kamilu M.; Henein, Michael Y. | Peripartum cardiomyopathy: A review article | Irrelevant results |
| 794 | 2010 | Colatrella, Antonietta; Loguercio, Valentina; Mattei, Luca; Trappolini, Massimo; Festa, Camilla; Stoppo, Michela; Napoli, Angela | Hypertension in diabetic pregnancy: impact and long-term outlook | Irrelevant results |
| 795 | 2015 | Othman, Ahmad A.; Soliman, Rasha H. | Schistosomiasis in Egypt: A never-ending story? | Irrelevant results |
| 796 | 2015 | Liu, Li; Oza, Shefali; Hogan, Daniel; Perin, Jamie; Rudan, Igor; Lawn, Joy E; Cousens, Simon; Mathers, Colin; Black, Robert E | Global, regional, and national causes of child mortality in 2000–13, with projections to inform post-2015 priorities: an updated systematic analysis | Irrelevant results |
| 797 | 2018 | White, Katie D.; Abe, Riichiro; Ardern-Jones, Michael; Beachkofsky, Thomas; Bouchard, Charles; Carleton, Bruce; and al | SJS/TEN 2017: Building Multidisciplinary Networks to Drive Science and Translation | Irrelevant results |
| 798 | 2017 | Glasziou, Paul; Straus, Sharon; Brownlee, Shannon; Trevena, Lyndal; Dans, Leonila; Guyatt, Gordon; Elshaug, Adam G; Janett, Robert; Saini, Vikas | Evidence for underuse of effective medical services around the world | Irrelevant results |
| 799 | 2004 | Brooker, Simon; Bethony, Jeffrey; Hotez, Peter J. | Human Hookworm Infection in the 21st Century | Irrelevant results |
| 800 | 2020 | Jiang, Hai-yin; Zhang, Xue; Pan, Li-ya; Ma, Yong-chun | Childhood infection and subsequent risk of psychotic disorders in adults: A systematic review and meta-analysis | Irrelevant results |
| 801 | 2019 | Galasso, Emanuela; Weber, Ann M; Stewart, Christine P; Ratsifandrihamanana, Lisy; Fernald, Lia C H | Effects of nutritional supplementation and home visiting on growth and development in young children in Madagascar: a cluster-randomised controlled trial | Irrelevant results |
| 802 | 2012 | Rosenstein, Melissa G.; Cheng, Yvonne W.; Snowden, Jonathan M.; Nicholson, James M.; Doss, Amy E.; Caughey, Aaron B. | The risk of stillbirth and infant death stratified by gestational age in women with gestational diabetes | Irrelevant results |
| 803 | 2015 | Bialas, Kristy M.; Swamy, Geeta K.; Permar, Sallie R. | Perinatal Cytomegalovirus and Varicella Zoster Virus Infections: Epidemiology, Prevention, and Treatment | Irrelevant results |
| 804 | 2021 | Ferraz, Leslie Raphael de Moura; Silva, Laysa Creusa Paes Barreto Barros; de Souza, Myla Lôbo; Alves, Larissa Pereira; Sales, Victor de Albuquerque Wanderley; Barbosa, Ilka do Nascimento Gomes; and al | DRUG ASSOCIATIONS AS ALTERNATIVE AND COMPLEMENTARY THERAPY FOR NEGLECTED TROPICAL DISEASES | Irrelevant results |
| 805 | 2016 | Adebayo, Samson B.; Gayawan, Ezra; Heumann, Christian; Seiler, Christian | Joint modeling of Anaemia and Malaria in children under five in Nigeria | Irrelevant results |
| 806 | 2015 | De Jesus, Lilia C.; Sood, Beena G.; Shankaran, Seetha; Kendrick, Douglas; Das, Abhik; Bell, Edward F.; and al | Antenatal magnesium sulfate exposure and acute cardiorespiratory events in preterm infants | Irrelevant results |
| 807 | 2006 | Glynn, Laura M.; Sandman, Curt A. | The Influence of Prenatal Stress and Adverse Birth Outcome on Human Cognitive and Neurological Development* | Irrelevant results |
| 808 | 2012 | Mwaniki, Michael K; Atieno, Maurine; Lawn, Joy E; Newton, Charles RJC | Long-term neurodevelopmental outcomes after intrauterine and neonatal insults: a systematic review | Irrelevant results |
| 809 | 2001 | Addo-Yobo, Emmanuel O. D.; Custovic, Adnan; Taggart, Simon C. O.; Craven, Mark; Bonnie, Baffoe; Woodcock, Ashley | Risk factors for asthma in urban Ghana | Irrelevant results |
| 810 | 2019 | McAuley, Ryan | Clinical Tools for Working Abroad with Migrants | Irrelevant results |
| 811 | 2016 | Roy, Arunima; Hechtman, Lily; Arnold, L. Eugene; Sibley, Margaret H.; Molina, Brooke S. G.; Swanson, James M.; and al | Childhood Factors Affecting Persistence and Desistence of Attention-Deficit/Hyperactivity Disorder Symptoms in Adulthood: Results From the MTA | Irrelevant results |
| 812 | 2014 | O’Connor, Thomas G.; Tang, Wan; Gilchrist, Michelle A.; Moynihan, Jan A.; Pressman, Eva K.; Blackmore, Emma Robertson | Diurnal cortisol patterns and psychiatric symptoms in pregnancy: Short-term longitudinal study | Irrelevant results |
| 813 | 2019 | Hoyt, Lindsay Till; Sabol, Terri J.; Chaku, Natasha; Kessler, Courtenay L. | Family income from birth through adolescence: Implications for positive youth development | Irrelevant results |
| 814 | 2009 | Gracey, Michael; King, Malcolm | Indigenous health part 1: determinants and disease patterns | Irrelevant results |
| 815 | 2016 | Lee, Bandy X. | Causes and cures VII: Structural violence | Irrelevant results |
| 816 | 2021 | Palmer, Kirsten R; Tanner, Michael; Davies-Tuck, Miranda; Rindt, Andrea; Papacostas, Kerrie; Giles, Michelle L and al | Widespread implementation of a low-cost telehealth service in the delivery of antenatal care during the COVID-19 pandemic: an interrupted time-series analysis | Irrelevant results |
| 817 | 2012 | Newfield, Emily | Third-Trimester Pregnancy Complications | Irrelevant results |
| 818 | 2013 | Pallitto, Christina C.; García-Moreno, Claudia; Jansen, Henrica A. F. M.; Heise, Lori; Ellsberg, Mary; Watts, Charlotte | Intimate partner violence, abortion, and unintended pregnancy: Results from the WHO Multi-country Study on Women's Health and Domestic Violence | Irrelevant results |
| 819 | 2020 | Cuevas, Adolfo G.; Ong, Anthony D.; Carvalho, Keri; Ho, Thao; Chan, Sze Wan (Celine); Allen, Jennifer D.; Chen, Ruijia; Rodgers, Justin; Biba, Ursula; Williams, David R. | Discrimination and systemic inflammation: A critical review and synthesis | Irrelevant results |
| 820 | 2012 | Azria, Elie; Le Meaux, Jean-Patrick; Khoshnood, Babak; Alexander, Sophie; Subtil, Damien; Goffinet, François | Factors associated with adverse perinatal outcomes for term breech fetuses with planned vaginal delivery | Irrelevant results |
| 821 | 2002 | Fallo, Aurelia A.; Dobrzanski-Nisiewicz, Wanda; Sordelli, Nora; Alejandra Cattaneo, María; Scott, Gwendolyn; López, Eduardo L. | Clinical and epidemiologic aspects of human immunodeficiency virus-1-infected children in Buenos Aires, Argentina | Irrelevant results |
| 822 | 2016 | Demombynes, Gabriel; Trommlerová, Sofia Karina | What has driven the decline of infant mortality in Kenya in the 2000s? | Irrelevant results |
| 823 | 2015 | Breymann, Christian | Iron Deficiency Anemia in Pregnancy | Irrelevant results |
| 824 | 2002 | Morkjaroenpong, V.; Rand, Cynthia S.; Butz, Arlene M.; Huss, Karen; Eggleston, Peyton; Malveaux, Floyd J.; Bartlett, Susan J. | Environmental tobacco smoke exposure and nocturnal symptoms among inner-city children with asthma | Irrelevant results |
| 825 | 2018 | Chung, Hye Won; Kim, Eun Mee; Lee, Ji-Eun | Comprehensive understanding of risk and protective factors related to adolescent pregnancy in low- and middle-income countries: A systematic review | Irrelevant results |
| 826 | 2009 | Duncan, Burris | Global Child Health: Promises Made to Children—Not Yet Kept | Irrelevant results |
| 827 | 2018 | Hodges, Ashley L.; Holland, Aimee Chism | Common Sexually Transmitted Infections in Women | Irrelevant results |
| 828 | 2020 | Amha, Haile; Fente, Worku; Sintayehu, Mezinew; Tesfaye, Bekele; Yitayih, Mulat | Depression and associated factors among old age population in Dega damot district, North West Ethiopia. A cross-sectional study | Irrelevant results |
| 829 | 2020 | Lee, Kai Wei; Ching, Siew Mooi; Hoo, Fan Kee; Ramachandran, Vasudevan; Chong, Seng Choi; Tusimin, Maiza; Mohd Nordin, Noraihan; Devaraj, Navin Kumar; Cheong, Ai Theng; Chia, Yook Chin | Neonatal outcomes and its association among gestational diabetes mellitus with and without depression, anxiety and stress symptoms in Malaysia: A cross-sectional study | Irrelevant results |
| 830 | 2008 | de Sherbinin, Alex; VanWey, Leah K.; McSweeney, Kendra; Aggarwal, Rimjhim; Barbieri, Alisson; Henry, Sabine; Hunter, Lori M.; Twine, Wayne; Walker, Robert | Rural household demographics, livelihoods and the environment | Irrelevant results |
| 831 | 2017 | Fall, Tove; Mendelson, Michael; Speliotes, Elizabeth K. | Recent Advances in Human Genetics and Epigenetics of Adiposity: Pathway to Precision Medicine? | Irrelevant results |
| 832 | 2003 | Kennedy, Suzanne; Stone, Amy; Rachelefsky, Gary | Factors associated with emergency department use in asthma: acute care interventions improving chronic disease outcomes | Irrelevant results |
| 833 | 2007 | Mangan, Joan M. | The Potential for Reducing Asthma Disparities Through Improved Family and Social Function and Modified Health Behaviors | Irrelevant results |
| 834 | 2017 | Kabisch, Nadja; van den Bosch, Matilda; Lafortezza, Raffaele | The health benefits of nature-based solutions to urbanization challenges for children and the elderly – A systematic review | Irrelevant results |
| 835 | 2018 | Rosenberg, Adam M.; Maluccio, John A.; Harris, Jody; Mwanamwenge, Marjolein; Nguyen, Phuong H.; Tembo, Gelson; Rawat, Rahul | Nutrition-sensitive agricultural interventions, agricultural diversity, food access and child dietary diversity: Evidence from rural Zambia | Irrelevant results |
| 836 | 2002 | Simkin, Penny P.; O'Hara, MaryAnn | Nonpharmacologic relief of pain during labor: Systematic reviews of five methods | Irrelevant results |
| 837 | 2015 | Ruiter, Laura; Ravelli, Anita C. J.; de Graaf, Irene M.; Mol, Ben Willem J.; Pajkrt, Eva | Incidence and recurrence rate of placental abruption: a longitudinal linked national cohort study in the Netherlands | Irrelevant results |
| 838 | 2000 | Iyengar, G. Venkatesh; Nair, Padmanabhan P. | Global outlook on nutrition and the environment: meeting the challenges of the next millennium | Irrelevant results |
| 839 | 2011 | Lee, Robert; Schwartz, Robert A. | Human T-lymphotrophic virus type 1–associated infective dermatitis: A comprehensive review | Irrelevant results |
| 840 | 2011 | Baron, Ida Sue; Erickson, Kristine; Ahronovich, Margot D.; Baker, Robin; Litman, Fern R. | Cognitive deficit in preschoolers born late-preterm | Irrelevant results |
| 841 | 2016 | Martins, Ana Luisa Oenning; da Silva Fernandes Nascimento, Deisy; Schneider, Ione Jayce Ceola; Schuelter-Trevisol, Fabiana | Incidence of community-acquired infections of lower airways among infants | Irrelevant results |
| 842 | 2016 | Bourke, Claire D.; Berkley, James A.; Prendergast, Andrew J. | Immune Dysfunction as a Cause and Consequence of Malnutrition | Irrelevant results |
| 843 | 2019 | Lusvan, Munkh-Erdene; Debellut, Frédéric; Clark, Andrew; Demberelsuren, Sodbayar; Otgonbayar, Dashpagam; Batjargal, Tselkhaasuren; Purevsuren, Sugarmaa; Groman, Devin; Tate, Jacqueline; Pecenka, Clint | Projected impact, cost-effectiveness, and budget implications of rotavirus vaccination in Mongolia | Irrelevant results |
| 844 | 2017 | More, Neena Shah; Das, Sushmita; Bapat, Ujwala; Alcock, Glyn; Manjrekar, Shreya; Kamble, Vikas; Sawant, Rijuta; Shende, Sushma; Daruwalla, Nayreen; Pantvaidya, Shanti; Osrin, David | Community resource centres to improve the health of women and children in informal settlements in Mumbai: a cluster-randomised, controlled trial | Irrelevant results |
| 845 | 2004 | Walton, Shelley F.; Holt, Deborah C.; Currie, Bart J.; Kemp, David J. | Scabies: New Future for a Neglected Disease | Irrelevant results |
| 846 | 2006 | Eapen, Valsamma; Mabrouk, Abdel Azim; Bin-Othman, Salem | Disordered eating attitudes and symptomatology among adolescent girls in the United Arab Emirates | Irrelevant results |
| 847 | 2008 | Keenan, Kate; Hipwell, Alison; Feng, Xin; Babinski, Dara; Hinze, Amanda; Rischall, Michal; Henneberger, Angela | Subthreshold Symptoms of Depression in Preadolescent Girls Are Stable and Predictive of Depressive Disorders | Irrelevant results |
| 848 | 2014 | Singh, Kaleshwar Prasad; Jain, Parul; Prakash, Om; Khan, Danish Nasar; Gupta, Shikha; Prakash, Shantanu; Singh, Desh Deepak; Jain, Amita | Outbreaks of measles and chickenpox in eastern Uttar Pradesh, India | Irrelevant results |
| 849 | 2017 | Sordillo, Joanne E.; Zhou, Yanjiao; McGeachie, Michael J.; Ziniti, John; Lange, Nancy; Laranjo, Nancy; and al | Factors influencing the infant gut microbiome at age 3-6 months: Findings from the ethnically diverse Vitamin D Antenatal Asthma Reduction Trial (VDAART) | Irrelevant results |
| 850 | 2000 | Sprich, SUSAN; Biederman, JOSEPH; Crawford, MARGARET HARDING; Mundy, ELIZABETH; Faraone, STEPHEN V. | Adoptive and Biological Families of Children and Adolescents With ADHD | Irrelevant results |
| 851 | 2016 | Cullins, Lisa M.; Gabriel, Mary; Solages, Martine; Call, David; McKnight, Shalice; Concepcion, Milangel; Cho, Jang | Pediatric Community Mental Health | Irrelevant results |
| 852 | 2019 | Reid, Brie M.; Coe, Christopher L.; Doyle, Colleen M.; Sheerar, Dagna; Slukvina, Alla; Donzella, Bonny; Gunnar, Megan R. | Persistent skewing of the T-cell profile in adolescents adopted internationally from institutional care | Irrelevant results |
| 853 | 2011 | Gould Rothberg, Bonnie E.; Magriples, Urania; Kershaw, Trace S.; Rising, Sharon Schindler; Ickovics, Jeannette R. | Gestational weight gain and subsequent postpartum weight loss among young, low-income, ethnic minority women | Irrelevant results |
| 854 | 2012 | Edmond, Karen M; Kortsalioudaki, Christina; Scott, Susana; Schrag, Stephanie J; Zaidi, Anita KM; Cousens, Simon; Heath, Paul T | Group B streptococcal disease in infants aged younger than 3 months: systematic review and meta-analysis | Irrelevant results |
| 855 | 2011 | Merwat, Shehzad N.; Vierling, John M. | HIV Infection and the Liver: The Importance of HCV-HIV Coinfection and Drug-Induced Liver Injury | Irrelevant results |
| 856 | 2017 | Wildeman, Christopher; Wang, Emily A | Mass incarceration, public health, and widening inequality in the USA | Irrelevant results |
| 857 | 2000 | Clancy, Robert R.; McGaurn, Susan A.; Wernovsky, Gil; Spray, Thomas L.; Norwood, William I.; Jacobs, Marshall L.; Murphy, John D.; Gaynor, J. William; Goin, James E. | Preoperative risk-of-death prediction model in heart surgery with deep hypothermic circulatory arrest in the neonate | Irrelevant results |
| 858 | 2003 | Federico, Monica J; Liu, Andrew H | Overcoming childhood asthma disparities of the inner-city poor | Irrelevant results |
| 859 | 2002 | Schulz, Richard; Drayer, Rebecca A; Rollman, Bruce L | Depression as a risk factor for non-suicide mortality in the elderly | Irrelevant results |
| 860 | 2010 | Godoy, Ricardo; Magvanjav, Oyunbileg; Nyberg, Colleen; Eisenberg, Dan T. A.; McDade, Thomas W.; Leonard, William R.; Reyes-García, Victoria; Huanca, Tomás; Tanner, Susan; Gravlee, Clarence | Why no adult stunting penalty or height premium?: Estimates from native Amazonians in Bolivia | Irrelevant results |
| 861 | 2017 | Hendrickson, Timothy J.; Mueller, Bryon A.; Sowell, Elizabeth R.; Mattson, Sarah N.; Coles, Claire D.; Kable, Julie A.; Jones, Kenneth L.; Boys, Christopher J.; Lim, Kelvin O.; Riley, Edward P.; Wozniak, Jeffrey R. | Cortical gyrification is abnormal in children with prenatal alcohol exposure | Irrelevant results |
| 862 | 2000 | Andolsek, Kathryn M.; Kelton, Gaylen M. | RISK ASSESSMENT | Irrelevant results |
| 863 | 2017 | Owada, Kei; Nielsen, Mark; Lau, Colleen L.; Clements, Archie C. A.; Yakob, Laith; Soares Magalhães, Ricardo J. | Chapter One - Measuring the Effect of Soil-Transmitted Helminth Infections on Cognitive Function in Children: Systematic Review and Critical Appraisal of Evidence | Irrelevant results |
| 864 | 2012 | Salomon, Joshua A; Wang, Haidong; Freeman, Michael K; Vos, Theo; Flaxman, Abraham D; Lopez, Alan D; Murray, Christopher JL | Healthy life expectancy for 187 countries, 1990–2010: a systematic analysis for the Global Burden Disease Study 2010 | Irrelevant results |
| 865 | 2015 | Mehta, Ravindra L; Cerdá, Jorge; Burdmann, Emmanuel A; Tonelli, Marcello; García-García, Guillermo; Jha, Vivekanand; and al | International Society of Nephrology's 0by25 initiative for acute kidney injury (zero preventable deaths by 2025): a human rights case for nephrology | Irrelevant results |
| 866 | 2009 | Torjesen, Kristine; Olness, Karen | International Child Health: State of the Art | Irrelevant results |
| 867 | 2009 | Waldman, H. Barry; Hasan, Faysal M.; Perlman, Steven | Down Syndrome and Sleep-Disordered Breathing: The Dentist's Role | Irrelevant results |
| 868 | 2013 | Niessen, Linda C.; Gibson, Gretchen; Kinnunen, Taru H. | Women’s Oral Health: Why Sex and Gender Matter | Irrelevant results |
| 869 | 2001 | Walker-Bone, Karen; Dennison, Elaine; Cooper, Cyrus | Epidemiology of Osteoporosis | Irrelevant results |
| 870 | 2018 | Ekker, Merel S; Boot, Esther M; Singhal, Aneesh B; Tan, Kay Sin; Debette, Stephanie; Tuladhar, Anil M; de Leeuw, Frank-Erik | Epidemiology, aetiology, and management of ischaemic stroke in young adults | Irrelevant results |
| 871 | 2018 | Nebel, Rebecca A.; Aggarwal, Neelum T.; Barnes, Lisa L.; Gallagher, Aimee; Goldstein, Jill M.; Kantarci, Kejal; Mallampalli, Monica P.; Mormino, Elizabeth C.; Scott, Laura; Yu, Wai Haung; Maki, Pauline M.; Mielke, Michelle M. | Understanding the impact of sex and gender in Alzheimer's disease: A call to action | Irrelevant results |
| 872 | 2019 | Fiatal, Szilvia; Pikó, Péter; Kósa, Zsigmond; Sándor, János; Ádány, Róza | Genetic profiling revealed an increased risk of venous thrombosis in the Hungarian Roma population | Irrelevant results |
| 873 | 2014 | Gordon, Stephen B; Bruce, Nigel G; Grigg, Jonathan; Hibberd, Patricia L; Kurmi, Om P; Lam, Kin-bong Hubert; and al | Respiratory risks from household air pollution in low and middle income countries | Irrelevant results |
| 874 | 2015 | Mazumder, Sarmila; Taneja, Sunita; Bhatia, Kiran; Yoshida, Sachiyo; Kaur, Jasmine; Dube, Brinda; Toteja, G S; Bahl, Rajiv; Fontaine, Olivier; Martines, Jose; Bhandari, Nita | Efficacy of early neonatal supplementation with vitamin A to reduce mortality in infancy in Haryana, India (Neovita): a randomised, double-blind, placebo-controlled trial | Irrelevant results |
| 875 | 2004 | Boyer, Sue G.; Boyer, Kenneth M. | Update on TORCH infections in the newborn infant | Irrelevant results |
| 876 | 2016 | Rollins, Nigel C; Bhandari, Nita; Hajeebhoy, Nemat; Horton, Susan; Lutter, Chessa K; Martines, Jose C; Piwoz, Ellen G; Richter, Linda M; Victora, Cesar G | Why invest, and what it will take to improve breastfeeding practices? | Irrelevant results |
| 877 | 2017 | Thirlwall, Kerstin; Cooper, Peter; Creswell, Cathy | Guided parent-delivered cognitive behavioral therapy for childhood anxiety: Predictors of treatment response | Irrelevant results |
| 878 | 2002 | Hatton, Chris | People with intellectual disabilities from ethnic minority communities in the United States and the United Kingdom | Irrelevant results |
| 879 | 2006 | Berry, Diane; Urban, Andrea; Grey, Margaret | Understanding the Development and Prevention of Type 2 Diabetes in Youth (Part 1) | Irrelevant results |
| 880 | 2016 | du Toit, George; Tsakok, Teresa; Lack, Simon; Lack, Gideon | Prevention of food allergy | Irrelevant results |
| 881 | 2019 | Blum, Robert Wm; Li, Mengmeng; Naranjo-Rivera, Gia | Measuring Adverse Child Experiences Among Young Adolescents Globally: Relationships With Depressive Symptoms and Violence Perpetration | Irrelevant results |
| 882 | 2010 | Tornheim, Jeffrey A.; Manya, Ayub S.; Oyando, Norbert; Kabaka, Stewart; O’Reilly, Ciara E.; Breiman, Robert F.; Feikin, Daniel R. | The epidemiology of hospitalization with diarrhea in rural Kenya: the utility of existing health facility data in developing countries | Irrelevant results |
| 883 | 2018 | Pahel, Bhavna T.; Rowan-Legg, Anne; Quinonez, Rocio B. | A Developmental Approach to Pediatric Oral Health | Irrelevant results |
| 884 | 2017 | Druetz, Thomas; Bicaba, Abel; Some, Telesphore; Kouanda, Seni; Ly, Antarou; Haddad, Slim | Effect of interrupting free healthcare for children: Drawing lessons at the critical moment of national scale-up in Burkina Faso | Irrelevant results |
| 886 | 2019 | Humphrey, Jean H; Mbuya, Mduduzi N N; Ntozini, Robert; Moulton, Lawrence H; Stoltzfus, Rebecca J; Tavengwa, Naume V; and al | Independent and combined effects of improved water, sanitation, and hygiene, and improved complementary feeding, on child stunting and anaemia in rural Zimbabwe: a cluster-randomised trial | Irrelevant results |
| 887 | 2002 | Lachman, Peter; Poblete, Ximena; Ebigbo, Peter O; Nyandiya-Bundy, Sally; Bundy, Robert P; Killian, Bev; Doek, Jaap | Challenges facing child protection | Irrelevant results |
| 888 | 2009 | Suskind, David L. | Nutritional Deficiencies During Normal Growth | Irrelevant results |
| 889 | 2013 | George, James W.; Skaggs, Clayton D.; Thompson, Paul A.; Nelson, D. Michael; Gavard, Jeffrey A.; Gross, Gilad A. | A randomized controlled trial comparing a multimodal intervention and standard obstetrics care for low back and pelvic pain in pregnancy | Irrelevant results |
| 890 | 2004 | Howard, Louise Michele; Goss, Claudia; Leese, Morven; Appleby, Louis; Thornicroft, Graham | The psychosocial outcome of pregnancy in women with psychotic disorders | Irrelevant results |
| 891 | 2003 | Köhler, Carsten; Tebo, Anne E.; Dubois, Beatrice; Deloron, Philippe; Kremsner, Peter G.; 1-951C study team; Luty, Adrian J. F. | Temporal variations in immune responses to conserved regions of Plasmodium falciparum merozoite surface proteins related to the severity of a prior malaria episode in Gabonese children | Irrelevant results |
| 892 | 2018 | Prabhakaran, Dorairaj; Singh, Kavita; Roth, Gregory A.; Banerjee, Amitava; Pagidipati, Neha J.; Huffman, Mark D. | Cardiovascular Diseases in India Compared With the United States | Irrelevant results |
| 893 | 2020 | Bell, Scott C; Mall, Marcus A; Gutierrez, Hector; Macek, Milan; Madge, Susan; Davies, Jane C; and al | The future of cystic fibrosis care: a global perspective | Irrelevant results |
| 894 | 2013 | Dunham-Snary, Kimberly J.; Ballinger, Scott W. | Mitochondrial genetics and obesity: evolutionary adaptation and contemporary disease susceptibility | Irrelevant results |
| 895 | 2019 | Sikander, Siham; Ahmad, Ikhlaq; Atif, Najia; Zaidi, Ahmed; Vanobberghen, Fiona; Weiss, Helen A; and al | Delivering the Thinking Healthy Programme for perinatal depression through volunteer peers: a cluster randomised controlled trial in Pakistan | Irrelevant results |
| 896 | 2008 | Visootsak, Jeannie; Sherman, Stephanie L. | Chapter 3 Trisomy 21: Causes and Consequences | Irrelevant results |
| 897 | 2007 | Tanumihardjo, Sherry A.; Anderson, Cheryl; Kaufer-Horwitz, Martha; Bode, Lars; Emenaker, Nancy J.; Haqq, Andrea M.; Satia, Jessie A.; Silver, Heidi J.; Stadler, Diane D. | Poverty, Obesity, and Malnutrition: An International Perspective Recognizing the Paradox | Irrelevant results |
| 898 | 2018 | Greydanus, Donald E.; Agana, Marisha; Kamboj, Manmohan K.; Shebrain, Saad; Soares, Neelkamal; Eke, Ransome; Patel, Dilip R. | Pediatric obesity: Current concepts | Irrelevant results |
| 899 | 2016 | Desai, Priti P.; Rivera, Abigail Torres; Backes, Emily M. | Latino Caregiver Coping With Children's Chronic Health Conditions: An Integrative Literature Review | Irrelevant results |
| 900 | 2015 | Fernandez, Isabel Diana; Groth, Susan W.; Reschke, Jennifer E.; Graham, Meredith L.; Strawderman, Myla; Olson, Christine M. | eMoms: Electronically-mediated weight interventions for pregnant and postpartum women. Study design and baseline characteristics | Irrelevant results |
| 901 | 2000 | Guyatt, H. | Do Intestinal Nematodes Affect Productivity in Adulthood? | Irrelevant results |
| 902 | 2014 | Powell-Jackson, Timothy; Hanson, Kara; Whitty, Christopher J. M.; Ansah, Evelyn K. | Who benefits from free healthcare? Evidence from a randomized experiment in Ghana | Irrelevant results |
| 903 | 2021 | Asadi-Pooya, Ali A.; Brigo, Francesco; Kozlowska, Kasia; Perez, David L.; Pretorius, Chrisma; Sawchuk, Tyson; Saxena, Aneeta; Tolchin, Benjamin; Valente, Kette D. | Social aspects of life in patients with functional seizures: Closing the gap in the biopsychosocial formulation | Irrelevant results |
| 904 | 2001 | Larson, Elaine L.; Aiello, Allison E. | Hygiene and health: An epidemiologic link? | Irrelevant results |
| 905 | 2015 | Anders, Katherine L.; Thompson, Corinne N.; Thuy, Nguyen Thi Van; Nguyet, Nguyen Minh; Tu, Le Thi Phuong; Dung, Tran Thi Ngoc; and al | The epidemiology and aetiology of diarrhoeal disease in infancy in southern Vietnam: a birth cohort study | Irrelevant results |
| 906 | 2000 | Harlow, Siobán D.; Campbell, Oona M. R. | Menstrual dysfunction: A missed opportunity for improving reproductive health in developing countries | Irrelevant results |
| 907 | 2002 | Wachsler-Felder, Jana L.; Golden, Charles J. | Neuropsychological consequences of HIV in children: A review of current literature | Irrelevant results |
| 908 | 2016 | Matthews, Karen A.; Pantesco, Elizabeth J. M. | Sleep characteristics and cardiovascular risk in children and adolescents: an enumerative review | Irrelevant results |
| 909 | 2000 | Cacioppo, John T; Ernst, John M; Burleson, Mary H; McClintock, Martha K; Malarkey, William B; Hawkley, Louise C; and al | Lonely traits and concomitant physiological processes: the MacArthur social neuroscience studies | Irrelevant results |
| 910 | 2012 | Jansson, L. M.; Di Pietro, J. A.; Elko, A.; Williams, E. L.; Milio, L.; Velez, M. | Pregnancies exposed to methadone, methadone and other illicit substances, and poly-drugs without methadone: A comparison of fetal neurobehaviors and infant outcomes | Irrelevant results |
| 911 | 2002 | Flack, John M; Peters, Rosalind; Mehra, Vishal C; Nasser, Samar A | Hypertension in special populations | Irrelevant results |
| 912 | 2016 | Chu, Helen Y.; Katz, Joanne; Tielsch, James; Khatry, Subarna K.; Shrestha, Laxman; LeClerq, Steven C.; Magaret, Amalia; Kuypers, Jane; Steinhoff, Mark; Englund, Janet A. | Respiratory syncytial virus infection in infants in rural Nepal | Irrelevant results |
| 913 | 2005 | Walsh, Michele C.; Morris, Brenda H.; Wrage, Lisa A.; Vohr, Betty R.; Poole, W. Kenneth; Tyson, Jon E.; Wright, Linda L.; Ehrenkranz, Richard A.; Stoll, Barbara J.; Fanaroff, Avroy A. | Extremely Low Birthweight Neonates with Protracted Ventilation: Mortality and 18-Month Neurodevelopmental Outcomes | Irrelevant results |
| 914 | 2007 | Mazaki-Tovi, Shali; Romero, Roberto; Kusanovic, Juan Pedro; Erez, Offer; Pineles, Beth L.; Gotsch, Francesca; Mittal, Pooja; Gabor Than, Nandor; Espinoza, Jimmy; Hassan, Sonia S. | Recurrent Preterm Birth | Irrelevant results |
| 915 | 2018 | Stroud, Laura R.; Papandonatos, George D.; McCallum, Meaghan; Kehoe, Tessa; Salisbury, Amy L.; Huestis, Marilyn A. | Prenatal tobacco and marijuana co-use: Impact on newborn neurobehavior | Irrelevant results |
| 916 | 2015 | Sibak, Mohammed; Moussa, Ibrahim; El-Tantawy, Nasr; Badr, Shaza; Chaudhri, Irtaza; Allam, Essam; Baxter, Louise; Abo Freikha, Saiyed; Hoestlandt, Céline; Lara, Carlos; Hajjeh, Rana; Munier, Aline | Cost-effectiveness analysis of the introduction of the pneumococcal conjugate vaccine (PCV-13) in the Egyptian national immunization program, 2013 | Irrelevant results |
| 917 | 2016 | Harrison, Margo S.; Eckert, Linda O.; Cutland, Clare; Gravett, Michael; Harper, Diane M.; McClure, Elizabeth M.; and al | Pathways to preterm birth: Case definition and guidelines for data collection, analysis, and presentation of immunization safety data | Irrelevant results |
| 918 | 2007 | Marmot, Michael | Achieving health equity: from root causes to fair outcomes | Irrelevant results |
| 919 | 2016 | Gulack, Brian C.; Laughon, Matthew M.; Clark, Reese H.; Burgess, Terrance; Robinson, Sybil; Muhammad, Abdurrauf; and al | Enteral Feeding with Human Milk Decreases Time to Discharge in Infants following Gastroschisis Repair | Irrelevant results |
| 920 | 2009 | Nguyen, Uyen-Sa D. T.; Rothman, Kenneth J.; Demissie, Serkalem; Jackson, Debra J.; Lang, Janet M.; Ecker, Jeffrey L. | Transfers Among Women Intending A Birth Center Delivery in the San Diego Birth Center Study | Irrelevant results |
| 921 | 2014 | Andro, Armelle; Cambois, Emmanuelle; Lesclingand, Marie | Long-term consequences of female genital mutilation in a European context: Self perceived health of FGM women compared to non-FGM women | Irrelevant results |
| 922 | 2016 | Ahmad, Nadia N.; Butsch, Winfield Scott; Aidarous, Sabina | Clinical Management of Obesity in Women: Addressing a Lifecycle of Risk | Irrelevant results |
| 923 | 2009 | Webster, Daniel P; Farrar, Jeremy; Rowland-Jones, Sarah | Progress towards a dengue vaccine | Irrelevant results |
| 924 | 2017 | Alaofè, Halimatou; Asaolu, Ibitola; Ehiri, Jennifer; Moretz, Hayley; Asuzu, Chisom; Balogun, Mobolanle; Abosede, Olayinka; Ehiri, John | Community Health Workers in Diabetes Prevention and Management in Developing Countries | Irrelevant results |
| 925 | 2021 | Xu, Lulu; Boama-Nyarko, Esther; Masters, Grace A.; Moore Simas, Tiffany A.; Ulbricht, Christine M.; Byatt, Nancy | Perspectives on barriers and facilitators to mental health support after a traumatic birth among a sample of primarily White and privately insured patients | Irrelevant results |
| 926 | 2007 | Goldman, Armond S.; Hopkinson, Judy M.; Rassin, David K. | Benefits and Risks of Breastfeeding | Irrelevant results |
| 927 | 2007 | Owens, Judith | Classification and Epidemiology of Childhood Sleep Disorders | Irrelevant results |
| 928 | 2014 | Nerenberg, Kara; Daskalopoulou, Stella S.; Dasgupta, Kaberi | Gestational Diabetes and Hypertensive Disorders of Pregnancy as Vascular Risk Signals: An Overview and Grading of the Evidence | Irrelevant results |
| 929 | 2017 | Pratt, Charlotte A.; Loria, Catherine M.; Arteaga, Sonia S.; Nicastro, Holly L.; Lopez-Class, Maria; de Jesus, Janet M.; and al | A Systematic Review of Obesity Disparities Research | Irrelevant results |
| 930 | 2014 | Lin, Ming-Jen; Liu, Elaine M. | Does in utero exposure to Illness matter? The 1918 influenza epidemic in Taiwan as a natural experiment | Irrelevant results |
| 931 | 2009 | Schleiss, Mark R. | Persistent and Recurring Viral Infections: The Human Herpesviruses | Irrelevant results |
| 932 | 2011 | Alsan, Marcella M.; Westerhaus, Michael; Herce, Michael; Nakashima, Koji; Farmer, Paul E. | Poverty, Global Health, and Infectious Disease: Lessons from Haiti and Rwanda | Irrelevant results |
| 933 | 2010 | Cameron, Noël; Hawley, Nicola L. | Should the UK use WHO growth charts? | Irrelevant results |
| 934 | 2015 | Ordóñez, Anna E.; Collins, Pamela Y. | Advancing Research to Action in Global Child Mental Health | Irrelevant results |
| 935 | 2008 | Trigg, Bruce G.; Kerndt, Peter R.; Aynalem, Getahun | Sexually Transmitted Infections and Pelvic Inflammatory Disease in Women | Irrelevant results |
| 936 | 2011 | Wilson, Lynda; Bodin, Mary Beth; Fernandez, Patricia; Godoy, Guillermo; Sambuceti, Carolina; Squarre, Regis; and | Neonatal Intensive Care: A Global Perspective of Similarities and Differences in Selected Neonatal Intensive Care Units in Brazil, Chile, the United States, and Zambia | Irrelevant results |
| 937 | 2016 | Liu, Andrew H.; Anderson, William C.; Dutmer, Cullen M.; Searing, Daniel A.; Szefler, Stanley J. | Advances in asthma 2015: Across the lifespan | Irrelevant results |
| 938 | 2016 | Hansen, Caitlin; Paintsil, Elijah | Infectious Diseases of Poverty in Children: A Tale of Two Worlds | Irrelevant results |
| 939 | 2010 | Szefler, Stanley J. | Advances in pediatric asthma in 2009: Gaining control of childhood asthma | Irrelevant results |
| 940 | 2010 | Chowa, Gina; Ansong, David; Masa, Rainier | Assets and child well-being in developing countries: A research review | Irrelevant results |
| 941 | 2016 | Kovacs, Maria; Obrosky, Scott; George, Charles | The course of major depressive disorder from childhood to young adulthood: Recovery and recurrence in a longitudinal observational study | Irrelevant results |
| 942 | 2019 | Anderson, Elizabeth; Durstine, J. Larry | Physical activity, exercise, and chronic diseases: A brief review | Irrelevant results |
| 943 | 2012 | Rakhmanina, Natella; Phelps, B. Ryan | Pharmacotherapy of Pediatric HIV Infection | Irrelevant results |
| 944 | 2012 | Bloch, Joan Rosen; Webb, David A.; Mathew, Leny; Culhane, Jennifer F. | Pregnancy Intention and Contraceptive Use at Six Months Postpartum Among Women With Recent Preterm Delivery | Irrelevant results |
| 945 | 2006 | Sliwa, Karen; Fett, James; Elkayam, Uri | Peripartum cardiomyopathy | Irrelevant results |
| 946 | 2010 | Scialli, Anthony R.; Ang, Robert; Breitmeyer, James; Royal, Mike A. | Childhood asthma and use during pregnancy of acetaminophen. A critical review | Irrelevant results |
| 947 | 2020 | Stolldorf, Deonni; Germack, Hayley D.; Harrison, Jordan; Riman, Kathryn; Brom, Heather; Cary, Michael; Gilmartin, Heather; Jones, Tammie; Norful, Allison; Squires, Allison | Health Equity Research in Nursing and Midwifery: Time to Expand Our Work | Irrelevant results |
| 948 | 2003 | Flack, John M; Peters, Rosalind; Mehra, Vishal C; Nasser, Samar A | Hypertension in special populations | Irrelevant results |
| 949 | 2020 | Hsu, Chiun Yu; Lehman, Heather K.; Wood, Beatrice L.; Benipal, Jaspreet; Humayun, Quratulain; Miller, Bruce D. | Comorbid Obesity and Depressive Symptoms in Childhood Asthma: A Harmful Synergy | Irrelevant results |
| 950 | 2015 | Obermeyer, Carla Makhlouf; Bott, Sarah; Sassine, Anniebelle J. | Arab Adolescents: Health, Gender, and Social Context | Irrelevant results |
| 951 | 2017 | Jannini, Emmanuele A. | SM = SM: The Interface of Systems Medicine and Sexual Medicine for Facing Non-Communicable Diseases in a Gender-Dependent Manner | Irrelevant results |
| 952 | 2018 | Boyd, Brian A.; Iruka, Iheoma U.; Pierce, Nigel P. | Chapter One - Strengthening Service Access for Children of Color With Autism Spectrum Disorders: A Proposed Conceptual Framework | Irrelevant results |
| 953 | 2010 | Mourão, Ana Filipa; Blyth, Fiona M.; Branco, Jaime C. | Generalised musculoskeletal pain syndromes | Irrelevant results |
| 954 | 2015 | Steiber, Alison; Hegazi, Refaat; Herrera, Marianella; Landy Zamor, Marie; Chimanya, Kudakwashe; Pekcan, Ayla Gülden; Redondo-Samin, Divina Cristy D.; Correia, Maria Isabel T. D.; Ojwang, Alice A. | Spotlight on Global Malnutrition: A Continuing Challenge in the 21st Century | Irrelevant results |
| 955 | 2004 | Asfaw, Abay; Braun, Joachim von; Klasen, Stephan | How Big is the Crowding-Out Effect of User Fees in the Rural Areas of Ethiopia? Implications for Equity and Resources Mobilization | Irrelevant results |
| 956 | 2017 | Hanxhiu, Anida; McKay, Karen; Singh-Grewal, Davinder; Fitzgerald, Dominic A. | Question 10: Could the Burden of Care with Cystic Fibrosis Impact on Educational Outcomes? | Irrelevant results |
| 957 | 2001 | Gidding, Samuel S. | Active and passive tobacco exposure | Irrelevant results |
| 958 | 2018 | Lascar, Nadia; Brown, James; Pattison, Helen; Barnett, Anthony H; Bailey, Clifford J; Bellary, Srikanth | Type 2 diabetes in adolescents and young adults | Irrelevant results |
| 959 | 2017 | Padhi, Tapas Ranjan; Das, Sujata; Sharma, Savitri; Rath, Soveeta; Rath, Suryasnata; Tripathy, Devjyoti; Panda, Krushna Gopal; Basu, Soumyava; Besirli, Cagri G. | Ocular parasitoses: A comprehensive review | Irrelevant results |
| 960 | 2004 | Semba, Richard D.; Bloem, Martin W. | Measles blindness | Irrelevant results |
| 961 | 2016 | Austad, Steven N.; Fischer, Kathleen E. | Sex Differences in Lifespan | Irrelevant results |
| 962 | 2002 | von Mutius, Erika | Worldwide asthma epidemic | Irrelevant results |
| 963 | 2004 | Flores, Glenn | Culture, Ethnicity, and Linguistic Issues in Pediatric Care: Urgent Priorities and Unanswered Questions | Irrelevant results |
| 964 | 2011 | Lee, Alfred Ian; Okam, Maureen M. | Anemia in Pregnancy | Irrelevant results |
| 965 | 2020 | Mantel, Carsten; Chu, Susan Y.; Hyde, Terri B.; Lambach, Philipp | Seasonal influenza vaccination in middle-income countries: Assessment of immunization practices in Belarus, Morocco, and Thailand | Irrelevant results |
| 966 | 2006 | Szaflarski, Magdalena; Szaflarski, Jerzy P.; Privitera, Michael D.; Ficker, David M.; Horner, Ronnie D. | Racial/ethnic disparities in the treatment of epilepsy: What do we know? What do we need to know? | Irrelevant results |
| 967 | 2017 | Hamprecht, Klaus; Goelz, Rangmar | Postnatal Cytomegalovirus Infection Through Human Milk in Preterm Infants: Transmission, Clinical Presentation, and Prevention | Irrelevant results |
| 968 | 2015 | D’Amato, Gennaro; Holgate, Stephen T.; Pawankar, Ruby; Ledford, Dennis K.; Cecchi, Lorenzo; Al-Ahmad, Mona; and al | Meteorological conditions, climate change, new emerging factors, and asthma and related allergic disorders. A statement of the World Allergy Organization | Irrelevant results |
| 969 | 2013 | Chang, Tammy; Choi, HwaJung; Richardson, Caroline R.; Davis, Matthew M. | Implications of teen birth for overweight and obesity in adulthood | Irrelevant results |
| 970 | 2010 | Miller, Assia; Siffel, Csaba; Lu, Chengxing; Riehle-Colarusso, Tiffany; Frías, Jaime L.; Correa, Adolfo | Long-Term Survival of Infants with Atrioventricular Septal Defects | Irrelevant results |
| 971 | 2015 | Trautmann, Jennifer; Alhusen, Jeanne; Gross, Deborah | Impact of deployment on military families with young children: A systematic review | Irrelevant results |
| 972 | 2012 | Vohr, Betty; Topol, Deborah; Girard, Nicole; St. Pierre, Lucille; Watson, Victoria; Tucker, Richard | Language outcomes and service provision of preschool children with congenital hearing loss | Irrelevant results |
| 973 | 2015 | Bastek, Jamie A.; Sammel, Mary D.; Jackson, Tara D.; Ryan, Meghan E.; McShea, Meghan A.; Elovitz, Michal A. | Environmental variables as potential modifiable risk factors of preterm birth in Philadelphia, PA | Irrelevant results |
| 974 | 2005 | Smith, Jeffrey; Haile-Mariam, Tenagne | Priorities in global emergency medicine development | Irrelevant results |
| 975 | 2004 | Cohen, Meryl S. | Fetal and childhood onset of adult cardiovascular diseases | Irrelevant results |
| 976 | 2015 | Malhotra, Savita; Padhy, Susanta Kumar | Challenges in Providing Child and Adolescent Psychiatric Services in Low Resource Countries | Irrelevant results |
| 977 | 2014 | Fanzo, Jessica | Strengthening the engagement of food and health systems to improve nutrition security: Synthesis and overview of approaches to address malnutrition | Irrelevant results |
| 978 | 2002 | Lipman, Terri H.; Deatrick, Janet A.; Treston, Carole S.; Lischner, Harold W.; Logan, Jeanne; Hassey, Kelly; Hale, Paula M.; Singer-Granick, Carol | Assessment of Growth and Immunologic Function in HIV-Infected and Exposed Children | Irrelevant results |
| 979 | 2004 | Perez-Stable, Eliseo J.; Salazar, Rene | Issues in achieving compliance with antihypertensive treatment in the Latino population | Irrelevant results |
| 980 | 2001 | Styne, Dennis M. | CHILDHOOD AND ADOLESCENT OBESITY: Prevalence and Significance | Irrelevant results |
| 981 | 2010 | Ganatra, Hammad A.; Stoll, Barbara J.; Zaidi, Anita K. M. | International Perspective on Early-Onset Neonatal Sepsis | Irrelevant results |
| 982 | 2004 | Hardy, Lynda R.; Harrell, Joanne S.; Bell, Ronny A. | Overweight in Children: Definitions, Measurements, Confounding Factors, and Health Consequences | Irrelevant results |
| 983 | 2008 | Apter, Andrea J. | Advances in the care of adults with asthma and allergy in 2007 | Irrelevant results |
| 984 | 2001 | Lawrence, Robert M.; Lawrence, Ruth A. | Given the Benefits of Breastfeeding, what Contraindications Exist? | Irrelevant results |
| 985 | 2014 | Dang, Khoi; Tribble, Alison C. | Strategies in Infectious Disease Prevention and Management Among US-Bound Refugee Children | Irrelevant results |
| 986 | 2018 | Saha, Samir K; Schrag, Stephanie J; El Arifeen, Shams; Mullany, Luke C; Shahidul Islam, Mohammad; Shang, Nong; and al | Causes and incidence of community-acquired serious infections among young children in south Asia (ANISA): an observational cohort study | Irrelevant results |
| 987 | 2011 | Szefler, Stanley J. | Advances in pediatric asthma in 2010: Addressing the major issues | Irrelevant results |
| 988 | 2010 | Istaphanous, George K.; Ward, Christopher G.; Loepke, Andreas W. | The impact of the perioperative period on neurocognitive development, with a focus on pharmacological concerns | Irrelevant results |
| 989 | 2014 | Hall, Kelli Stidham; Kusunoki, Yasamin; Gatny, Heather; Barber, Jennifer | Stress Symptoms and Frequency of Sexual Intercourse Among Young Women | Irrelevant results |
| 990 | 2017 | Onarheim, Kristine Husøy; Sisay, Mitike Molla; Gizaw, Muluken; Moland, Karen Marie; Miljeteig, Ingrid | What if the baby doesn't survive? Health-care decision making for ill newborns in Ethiopia | Irrelevant results |
| 991 | 2013 | Kramer, Michael R.; Williamson, Rebecca | Multivariate bayesian spatial model of preterm birth and cardiovascular disease among georgia women: Evidence for life course social determinants of health | Irrelevant results |
| 992 | 2012 | Gardner, Elizabeth; Chang, Myong; Mancuso, Peggy; Chaney, Susan E. | NEUROCYSTICERCOSIS in Pregnancy | Irrelevant results |
| 993 | 2019 | Benton, Tami D.; Kee Ng, Warren Yiu; Leung, Denise; Canetti, Alexandra; Karnik, Niranjan | Depression among Youth Living with HIV/AIDS | Irrelevant results |
| 994 | 2010 | Mapengo, Marta Artemisa Abel; Marsicano, Juliane Avansine; de Moura, Patrícia Garcia; Sales-Peres, Arsenio; Hobdell, Martin; Sales-Peres, Sílvia Helena de Carvalho | Dental caries in adolescents from public schools in Maputo, Mozambique | Irrelevant results |
| 995 | 2004 | Pillay, T; Khan, M; Moodley, J; Adhikari, M; Coovadia, H | Perinatal tuberculosis and HIV-1: considerations for resource-limited settings | Irrelevant results |
| 996 | 2013 | Jolliffe, David A.; Griffiths, Christopher J.; Martineau, Adrian R. | Vitamin D in the prevention of acute respiratory infection: Systematic review of clinical studies | Irrelevant results |
| 997 | 2021 | Aziz, Asma Binte; Zaman, K.; Kim, Deok Ryun; Park, Ju Yeon; Im, Justin; Ali, Mohammad; and al | Re-evaluation of population-level protection conferred by a rotavirus vaccine using the ‘fried-egg’ approach in a rural setting in Bangladesh | Irrelevant results |
| 998 | 2002 | Lambert, Michael C.; Sarnms-Vaughan, Maureen E.; Schmitt, Neal; Kirsch, Beth; Paneth, Nigel; Russ, Chad M. | Effects of environmental factors on psychological adjustment in very low birthweight and higher birthweight jamaican adolescents | Irrelevant results |
| 999 | 2004 | Grisaru, Sorina; Samueloff, Arnon | Primary nonmedically indicated cesarean section (“section on request”): evidence based or modern vogue? | Irrelevant results |
| 1000 | 2017 | Castillo, Eliana; McIsaac, Corrine; MacDougall, Bhreagh; Wilson, Douglas; Kohr, Rosemary | Post-Caesarean Section Surgical Site Infection Surveillance Using an Online Database and Mobile Phone Technology | Irrelevant results |
| 1001 | 2003 | Moizeau, Catherine E | The problem of preterm birth: effective primary prevention | Irrelevant results |
| 1002 | 2014 | McGovern, Mark E. | Comparing the relationship between stature and later life health in six low and middle income countries | Irrelevant results |
| 1003 | 2017 | Zombré, David; De Allegri, Manuela; Ridde, Valéry | Immediate and sustained effects of user fee exemption on healthcare utilization among children under five in Burkina Faso: A controlled interrupted time-series analysis | Irrelevant results |
| 1004 | 2015 | Kendig, Sue | Implications for Policy to Support Healthy Weight for Women | Irrelevant results |
| 1005 | 2012 | Cerdan, Noelle S.; Alpert, Patricia T.; Moonie, Sheniz; Cyrkiel, Dianne; Rue, Shona | Asthma severity in children and the quality of life of their parents | Irrelevant results |
| 1006 | 2009 | Fischer, Avi; Fuster, Valentin | The Changing Epidemiology of Sudden Cardiac Death | Irrelevant results |
| 1007 | 2007 | Walker, Neff; Bryce, Jennifer; Black, Robert E | Interpreting health statistics for policymaking: the story behind the headlines | Irrelevant results |
| 1008 | 2016 | Adeniran, Abiodun S.; Aboyeji, Abiodun P.; Fawole, Adegboyega A.; Balogun, Olayinka R.; Adesina, Kikelomo T.; Isiaka-Lawal, Salamat | Evaluation of parturient perception and aversion before and after primary cesarean delivery in a low-resource country | Irrelevant results |
| 1009 | 2008 | Read, Jennifer S.; Cannon, Michael J.; Stanberry, Lawrence R.; Schuval, Susan | Prevention of Mother-to-Child Transmission of Viral Infections | Irrelevant results |
| 1010 | 2013 | Szefler, Stanley J. | Advances in pediatric asthma in 2012: Moving toward asthma prevention | Irrelevant results |
| 1011 | 2016 | Greene, Michelle; Patra, Kousiki | Part C early intervention utilization in preterm infants: Opportunity for referral from a NICU follow-up clinic | Irrelevant results |
| 1012 | 2019 | Holland, Cara; Rammohan, Anu | Rural women’s empowerment and children’s food and nutrition security in Bangladesh | Irrelevant results |
| 1013 | 2010 | Butts, Samantha F.; Seifer, David B. | Racial and ethnic differences in reproductive potential across the life cycle | Irrelevant results |
| 1014 | 2017 | van Crevel, Reinout; van de Vijver, Steven; Moore, David A J | The global diabetes epidemic: what does it mean for infectious diseases in tropical countries? | Irrelevant results |
| 1015 | 2011 | Chinchure, Swati; Kesavadas, Chandrasekharan | Neuroimaging in Epilepsy in Tropics | Irrelevant results |
| 1016 | 2021 | Tucker Edmonds, Brownsyne; Hoffman, Shelley M; Laitano, Tatiana; Coleman-Phox, Kimberly; Castillo, Esperanza; Kuppermann, Miriam | User-testing of a decision-support tool for parents facing threatened periviable delivery: The Periviable GOALS decision aid | Irrelevant results |
| 1017 | 2016 | Mace, Sharon E. | Global Threats to Child Safety | Irrelevant results |
| 1018 | 2021 | González Rodríguez, Paz; Pérez-Moneo Agapito, Begoña; Albi Rodríguez, María Salomé; Aizpurua Galdeano, Pilar; Aparicio Rodrigo, María; Fernández Rodríguez, María Mercedes; Esparza Olcina, María Jesús; Ochoa Sangrador, Carlos | COVID-19: Critical appraisal of the evidence | Irrelevant results |
| 1019 | 2006 | Waxmonsky, JAMES; Wood, BEATRICE L.; Stern, TRUDY; Ballow, MARK; Lillis, KATHLEEN; Cramer-benjamin, DARCI; Mador, JEFFREY; Miller, BRUCE D. | Association of Depressive Symptoms and Disease Activity in Children With Asthma: Methodological and Clinical Implications | Irrelevant results |
| 1020 | 2016 | Caraballo, Luis; Zakzuk, Josefina; Lee, Bee Wah; Acevedo, Nathalie; Soh, Jian Yi; Sánchez-Borges, Mario; Hossny, Elham; García, Elizabeth; Rosario, Nelson; Ansotegui, Ignacio; Puerta, Leonardo; Sánchez, Jorge; Cardona, Victoria | Particularities of allergy in the Tropics | Irrelevant results |
| 1021 | 2021 | Gabryszewski, Stanislaw J.; Hill, David A. | One march, many paths: Insights into allergic march trajectories | Irrelevant results |
| 1022 | 2009 | Wells, Susan J.; Merritt, Lani M.; Briggs, Harold E. | Bias, racism and evidence-based practice: The case for more focused development of the child welfare evidence base | Irrelevant results |
| 1023 | 2010 | O’Neill, Sean; Cervera, Ricard | Systemic lupus erythematosus | Irrelevant results |
| 1024 | 2013 | Nyarko, Kwame A.; Lopez-Camelo, Jorge; Castilla, Eduardo E.; Wehby, George L. | Does the Relationship between Prenatal Care and Birth Weight Vary by Oral Clefts? Evidence Using South American and United States Samples | Irrelevant results |
| 1025 | 2014 | Zhou, Sherry; Rosenthal, David G.; Sherman, Scott; Zelikoff, Judith; Gordon, Terry; Weitzman, Michael | Physical, Behavioral, and Cognitive Effects of Prenatal Tobacco and Postnatal Secondhand Smoke Exposure | Irrelevant results |
| 1026 | 2011 | Jackson, Alan A. | Feeding the normal infant, child and adolescent | Irrelevant results |
| 1027 | 2017 | Cesario, Sandra K.; Moran, Barbara | Empowering the Girl Child, Improving Global Health | Irrelevant results |
| 1028 | 2014 | Shah, Iqbal H.; Åhman, Elisabeth; Ortayli, Nuriye | Access to safe abortion: progress and challenges since the 1994 International Conference on Population and Development (ICPD) | Irrelevant results |
| 1029 | 2008 | McWilliam, Andrew; Smith, Andrew | National UK audit projects in anaesthesia | Irrelevant results |
| 1030 | 2018 | Lalan, Shwetal; Jiang, Shuai; Ng, Derek K.; Kupferman, Fernanda; Warady, Bradley A.; Furth, Susan; Mitsnefes, Mark M. | Cardiometabolic Risk Factors, Metabolic Syndrome, and Chronic Kidney Disease Progression in Children | Irrelevant results |
| 1031 | 2000 | Howard, MATTHEW; Hodes, MATTHEW | Psychopathology, Adversity, and Service Utilization of Young Refugees | Irrelevant results |
| 1032 | 2018 | Santos, Hudson P.; Nephew, Benjamin C.; Bhattacharya, Arjun; Tan, Xianming; Smith, Laura; Alyamani, Reema Abdulrahman S.; and al | Discrimination exposure and DNA methylation of stress-related genes in Latina mothers | Irrelevant results |
| 1033 | 2014 | Moreno-Macias, Hortensia; Romieu, Isabelle | Effects of antioxidant supplements and nutrients on patients with asthma and allergies | Irrelevant results |
| 1034 | 2001 | De Bellis, Michael D; Broussard, Elsie R; Herring, David J; Wexler, Sandra; Moritz, Grace; Benitez, John G | Psychiatric co-morbidity in caregivers and children involved in maltreatment: a pilot research study with policy implications | Irrelevant results |
| 1035 | 2006 | Nash, Denis; Elul, Batya | The impact of HIV on cities in the era of treatment: A global perspective | Irrelevant results |
| 1036 | 2013 | Goss, Paul E; Lee, Brittany L; Badovinac-Crnjevic, Tanja; Strasser-Weippl, Kathrin; Chavarri-Guerra, Yanin; Louis, Jessica St; and al | Planning cancer control in Latin America and the Caribbean | Irrelevant results |
| 1037 | 2008 | Rosenberg, Adam | The IUGR Newborn | Irrelevant results |
| 1038 | 2021 | Elawar, Farah; Oraby, Ahmed K.; Kieser, Quinten; Jensen, Lionel D.; Culp, Tyce; West, Frederick G.; Marchant, David J. | Pharmacological targets and emerging treatments for respiratory syncytial virus bronchiolitis | Irrelevant results |
| 1039 | 2000 | Dodds, Sally; Blaney, Nancy T; Nuehring, Elane M; Blakley, Theresa; Lizzotte, Jean-Marie; Potter, JoNell E; O’Sullivan, Mary J | Integrating mental health services into primary care for HIV-infected pregnant and non-pregnant women: whole life—a theoretically derived model for clinical care and outcomes assessment | Irrelevant results |
| 1040 | 2003 | Hamaoui, Elie; Hamaoui, Michal | Nutritional assessment and support during pregnancy | Irrelevant results |
| 1041 | 2008 | Reddy, Aditya; Fried, Bernard | Chapter 3 Atopic Disorders and Parasitic Infections | Irrelevant results |
| 1042 | 2016 | Sharma, Krishan; Gupta, Puneet; Shandilya, Shailza | Age related changes in pelvis size among adolescent and adult females with reference to parturition from Naraingarh, Haryana (India) | Irrelevant results |
| 1043 | 2006 | Pillai, Vijayan K.; Gupta, Rashmi | Cross-national analysis of a model of reproductive health in developing countries | Irrelevant results |
| 1044 | 2015 | Rieger, Matthias; Wagner, Natascha | Child health, its dynamic interaction with nutrition and health memory – Evidence from Senegal | Irrelevant results |
| 1045 | 2004 | Gahagan, Sheila | Child and adolescent obesity | Irrelevant results |
| 1046 | 2020 | Corpuz, Randy; Bugental, Daphne | Life history and individual differences in male testosterone: Mixed evidence for early environmental calibration of testosterone response to first-time fatherhood | Irrelevant results |
| 1047 | 2009 | Leong, Rupert W. | Differences in Peptic Ulcer Between the East and the West | Irrelevant results |
| 1048 | 2020 | Miller, Jeffrey H.; Bardo, Dianna M. E.; Cornejo, Patricia | Neonatal Neuroimaging | Irrelevant results |
| 1049 | 2008 | Lewiecki, E. Michael; Bilezikian, John P.; Cooper, Cyrus; Hochberg, Marc C.; Luckey, Marjorie M.; Maricic, Michael; Miller, Paul D. | Proceedings of the Eighth Annual Santa Fe Bone Symposium, August 3–4, 2007 | Irrelevant results |
| 1050 | 2001 | Paul Schultz, T. | Chapter 8 Women's roles in the agricultural household: Bargaining and human capital investments | Irrelevant results |
| 1051 | 2004 | Connolly, Dana; McClowry, Sandra; Hayman, Laura; Mahony, Lynn; Artman, Michael | Posttraumatic stress disorder in children after cardiac surgery | Irrelevant results |
| 1052 | 2006 | Langkamp, Diane L.; Girardet, Rebecca G. | Primary Care for Twins and Higher Order Multiples | Irrelevant results |
| 1053 | 2016 | Njau, Joseph D.; Cairns, Lisa K. | A literature review on the economic benefits of vaccines in low and middle income countries: Evaluating progress in the era of ‘a decade of vaccines’ initiative | Irrelevant results |
| 1054 | 2000 | Manhart, Lisa E; Dialmy, Abdessamad; Ryan, Caroline A; Mahjour, Jaouad | Sexually transmitted diseases in Morocco: gender influences on prevention and health care seeking behavior | Irrelevant results |
| 1055 | 2011 | Seshia, Shashi S.; Bingham, William T.; Kirkham, Fenella J.; Sadanand, Venkatraman | Nontraumatic Coma in Children and Adolescents: Diagnosis and Management | Irrelevant results |
| 1056 | 2020 | Che, Ruochen; Quadri, Mohammed Mazheruddin; Zhang, Aihua | The Epidemiology and Management of Pediatric AKI in Asia | Irrelevant results |
| 1057 | 2006 | Kwon, Helen L.; Triche, Elizabeth W.; Belanger, Kathleen; Bracken, Michael B. | The Epidemiology of Asthma During Pregnancy: Prevalence, Diagnosis, and Symptoms | Irrelevant results |
| 1058 | 2011 | Moxon, E Richard; Siegrist, Claire-Anne | The next decade of vaccines: societal and scientific challenges | Irrelevant results |
| 1059 | 2005 | Fay, Marianne; Leipziger, Danny; Wodon, Quentin; Yepes, Tito | Achieving child-health-related Millennium Development Goals: The role of infrastructure | Irrelevant results |
| 1060 | 2013 | Eckerle, Judith K.; Howard, Cynthia R.; John, Chandy C. | Infections in Internationally Adopted Children | Irrelevant results |
| 1061 | 2013 | Lentz, Erin C.; Barrett, Christopher B. | The economics and nutritional impacts of food assistance policies and programs | Irrelevant results |
| 1062 | 2009 | Harpham, Trudy | Urban health in developing countries: What do we know and where do we go? | Irrelevant results |
| 1063 | 2003 | Cohen, Larry; Miller, Ted; Sheppard, Monique A.; Gordon, Emily; Gantz, Toni; Atnafou, Rebkha | Bridging the gap: Bringing together intentional and unintentional injury prevention efforts to improve health and well being | Irrelevant results |
| 1064 | 2015 | Mock, Charles N; Donkor, Peter; Gawande, Atul; Jamison, Dean T; Kruk, Margaret E; Debas, Haile T | Essential surgery: key messages from Disease Control Priorities, 3rd edition | Irrelevant results |
| 1065 | 2003 | Koch, Kenneth L; Frissora, Christine L | Nausea and vomiting during pregnancy | Irrelevant results |
| 1066 | 2011 | Preidis, Geoffrey A.; Hill, Colin; Guerrant, Richard L.; Ramakrishna, B. S.; Tannock, Gerald W.; Versalovic, James | Probiotics, Enteric and Diarrheal Diseases, and Global Health | Irrelevant results |
| 1067 | 2009 | Thorpe, Barbara M. | Integrating Osteoporosis Prevention and Treatment into Clinical Practice | Irrelevant results |
| 1068 | 2011 | Miller, Wilhelmine D.; Sadegh-Nobari, Tabashir; Lillie-Blanton, Marsha | Healthy Starts for All: Policy Prescriptions | Irrelevant results |
| 1069 | 2006 | Molyneux, David H. | Control of Human Parasitic Diseases: Context and Overview | Irrelevant results |
| 1070 | 2005 | Gessner, Bradford D; Sutanto, Agustinus; Linehan, Mary; Djelantik, I Gusti Gede; Fletcher, Tracy; Gerudug, I Komang; and al | Incidences of vaccine-preventable Haemophilus influenzae type b pneumonia and meningitis in Indonesian children: hamlet-randomised vaccine-probe trial | Irrelevant results |
| 1071 | 2013 | Nichols, Sharon L.; Bethel, James; Garvie, Patricia A.; Patton, Doyle E.; Thornton, Sarah; Kapogiannis, Bill G.; Ren, Weijia; Major-Wilson, Hanna; Puga, Ana; Woods, Steven P. | Neurocognitive Functioning in Antiretroviral Therapy–Naïve Youth With Behaviorally Acquired Human Immunodeficiency Virus | Irrelevant results |
| 1072 | 2002 | Lashley, Felissa R. | Newborn screening: New opportunities and new challenges | Irrelevant results |
| 1073 | 2005 | Feja, Kristina; Saiman, Lisa | Tuberculosis in Children | Irrelevant results |
| 1074 | 2011 | Chen, Melissa J.; Grobman, William A.; Gollan, Jackie K.; Borders, Ann E. B. | The use of psychosocial stress scales in preterm birth research | Irrelevant results |
| 1075 | 2017 | Blanchet, Karl; Ramesh, Anita; Frison, Severine; Warren, Emily; Hossain, Mazeda; Smith, James; and al | Evidence on public health interventions in humanitarian crises | Irrelevant results |
| 1076 | 2009 | Catenacci, Victoria A.; Hill, James O.; Wyatt, Holly R. | The Obesity Epidemic | Irrelevant results |
| 1077 | 2020 | Beuermann, Diether W.; Pecha, Camilo J. | The effect of eliminating health user fees on adult health and labor supply in Jamaica | Irrelevant results |
| 1078 | 2000 | Toppelberg, CLAUDIO O.; Shapiro, THEODORE | Language Disorders: A 10-Year Research Update Review | Irrelevant results |
| 1079 | 2010 | Waage, Jeff; Banerji, Rukmini; Campbell, Oona; Chirwa, Ephraim; Collender, Guy; Dieltiens, Veerle; and al | The Millennium Development Goals: a cross-sectoral analysis and principles for goal setting after 2015: Lancet and London International Development Centre Commission | Irrelevant results |
| 1080 | 2013 | Bardsley, Martha Zeger; Kowal, Karen; Levy, Carly; Gosek, Ania; Ayari, Natalie; Tartaglia, Nicole; Lahlou, Najiba; Winder, Breanna; Grimes, Shannon; Ross, Judith L. | 47,XYY Syndrome: Clinical Phenotype and Timing of Ascertainment | Irrelevant results |
| 1081 | 2010 | Misra, Subhasis; Solomon, Naveenraj L.; Moffat, Frederick L.; Koniaris, Leonidas G. | Screening Criteria for Breast Cancer | Irrelevant results |
| 1082 | 2015 | Yung, Chee-Fu; Chan, Siew Pang; Soh, Sally; Tan, Adriana; Thoon, Koh Cheng | Intussusception and Monovalent Rotavirus Vaccination in Singapore: Self-Controlled Case Series and Risk-Benefit Study | Irrelevant results |
| 1083 | 2010 | Giannoni, Peggy P.; Kass, Philip H. | Risk factors of children who exited from an early intervention program without an identified disability and returned with a developmental disability | Irrelevant results |
| 1084 | 2005 | Mosher, Catherine E.; Danoff-Burg, Sharon | Psychosocial impact of parental cancer in adulthood: A conceptual and empirical review | Irrelevant results |
| 1085 | 2003 | Thompson, Lisa; Kaufman, Lawrence M | The visually impaired child | Irrelevant results |
| 1086 | 2006 | Chutkan, Robynne | Colonoscopy Issues Related to Women | Irrelevant results |
| 1087 | 2003 | De Vera, Michelle J.; Drapkin, Sol; Moy, James N. | Association of recurrent wheezing with sensitivity to cockroach allergen in inner-city children | Irrelevant results |
| 1088 | 2016 | Russ, Karin; Howard, Sarah | Developmental Exposure to Environmental Chemicals and Metabolic Changes in Children | Irrelevant results |
| 1089 | 2016 | Andalón, Mabel; Azevedo, João Pedro; Rodríguez-Castelán, Carlos; Sanfelice, Viviane; Valderrama-González, Daniel | Weather Shocks and Health at Birth in Colombia | Irrelevant results |
| 1090 | 2018 | Klepac, Petra; Locatelli, Igor; Korošec, Sara; Künzli, Nino; Kukec, Andreja | Ambient air pollution and pregnancy outcomes: A comprehensive review and identification of environmental public health challenges | Irrelevant results |
| 1091 | 2014 | Maslan, Jonathan; Mims, James W. | What is Asthma? Pathophysiology, Demographics, and Health Care Costs | Irrelevant results |
| 1092 | 2013 | Harvey, Kayla A.; Kovalesky, Andrea; Woods, Ronald K.; Loan, Lori A. | Experiences of mothers of infants with congenital heart disease before, during, and after complex cardiac surgery | Irrelevant results |
| 1093 | 2016 | Rohra, Himanshi; Taneja, Ajay | Indoor air quality scenario in India—An outline of household fuel combustion | Irrelevant results |
| 1094 | 2005 | Mittal, Pooja; Wing, Deborah A. | Urinary Tract Infections in Pregnancy | Irrelevant results |
| 1095 | 2021 | Lee, Christopher R.; Chen, Alon; Tye, Kay M. | The neural circuitry of social homeostasis: Consequences of acute versus chronic social isolation | Irrelevant results |
| 1096 | 2016 | Nagel, Corey L.; Kirby, Miles A.; Zambrano, Laura D.; Rosa, Ghislane; Barstow, Christina K.; Thomas, Evan A.; Clasen, Thomas F. | Study design of a cluster-randomized controlled trial to evaluate a large-scale distribution of cook stoves and water filters in Western Province, Rwanda | Irrelevant results |
| 1097 | 2001 | Maimbolwa, Margaret C; Sikazwe, Nsama; Yamba, Bawa; Diwan, Vinod; Ransjö-Arvidson, Anna-Berit | Views on involving a social support person during labor in zambian maternities | Irrelevant results |
| 1098 | 2012 | Fazel, Mina; Reed, Ruth V; Panter-Brick, Catherine; Stein, Alan | Mental health of displaced and refugee children resettled in high-income countries: risk and protective factors | Irrelevant results |
| 1099 | 2019 | Enas, Enas A.; Varkey, Basil; Dharmarajan, T. S.; Pare, Guillaume; Bahl, Vinay K. | Lipoprotein(a): An underrecognized genetic risk factor for malignant coronary artery disease in young Indians | Irrelevant results |
| 1100 | 2000 | Sun, Joannie D; Weatherly, Robert A; Koopmann, Charles F; Carey, Thomas E | Mucosal swabs detect HPV in laryngeal papillomatosis patients but not family members | Irrelevant results |
| 1101 | 2015 | Joshi, Paramjit T.; Leventhal, Bennett L.; Fuentes, Joaquin | Partnering for the World's Children: Why Collaborations Are Important | Irrelevant results |
| 1102 | 2017 | Johnson, Christine C.; Ownby, Dennis R. | The infant gut bacterial microbiota and risk of pediatric asthma and allergic diseases | Irrelevant results |
| 1103 | 2013 | Thompson, David; Berger, Howard; Feig, Denice; Gagnon, Robert; Kader, Tina; Keely, Erin; Kozak, Sharon; Ryan, Edmond; Sermer, Mathew; Vinokuroff, Christina | Diabetes and Pregnancy | Irrelevant results |
| 1104 | 2009 | Zimran, Ari; Morris, Elizabeth; Mengel, Eugen; Kaplan, Paige; Belmatoug, Nadia; Hughes, Derralynn A.; Malinova, Vera; Heitner, Rene; Sobreira, Elisa; Mrsić, Mirando; Granovsky-Grisaru, Sorina; Amato, Dominick; vom Dahl, Stephan | The female Gaucher patient: The impact of enzyme replacement therapy around key reproductive events (menstruation, pregnancy and menopause) | Irrelevant results |
| 1105 | 2002 | McLENNAN, JOHN D.; Offord, DAVID R. | Should Postpartum Depression Be Targeted to Improve Child Mental Health? | Irrelevant results |
| 1106 | 2014 | Radhakrishnan, Dhenuka Kannan; Dell, Sharon D.; Guttmann, Astrid; Shariff, Salimah Z.; Liu, Kuan; To, Teresa | Trends in the age of diagnosis of childhood asthma | Irrelevant results |
| 1107 | 2009 | Szefler, Stanley J. | Advances in pediatric asthma in 2008: Where do we go now? | Irrelevant results |
| 1108 | 2014 | Winters, Brian R.; Walsh, Thomas J. | The Epidemiology of Male Infertility | Irrelevant results |
| 1109 | 2006 | Schleiss, Mark R.; Choo, Daniel I. | Mechanisms of congenital cytomegalovirus-induced deafness | Irrelevant results |
| 1110 | 2006 | Chen, Henian; Cohen, Patricia; Kasen, Stephanie; Johnson, Jeffrey G. | Adolescent Axis I and Personality Disorders Predict Quality of Life During Young Adulthood | Irrelevant results |
| 1111 | 2015 | Teaford, Dominique; Goyal, Deepika; McNeish, Susan G. | Identification of Postpartum Depression in an Online Community | Irrelevant results |
| 1112 | 2017 | Bischoff, Stephan C.; Boirie, Yves; Cederholm, Tommy; Chourdakis, Michael; Cuerda, Cristina; Delzenne, Nathalie M.; Deutz, Nicolaas E.; and al | Towards a multidisciplinary approach to understand and manage obesity and related diseases | Irrelevant results |
| 1113 | 2016 | Pérez-Moreno, Salvador; Blanco-Arana, María C.; Bárcena-Martín, Elena | Economic cycles and child mortality: A cross-national study of the least developed countries | Irrelevant results |
| 1114 | 2004 | Jones, Susan D.; Ehiri, John; Anyanwu, Ebere | Female genital mutilation in developing countries: an agenda for public health response | Irrelevant results |
| 1115 | 2002 | Stefanaki, Christina; Stratigos, Alexander J; Stratigos, John D | Skin manifestations of HIV-1 infection in children | Irrelevant results |
| 1116 | 2018 | Hendrickson, Timothy J.; Mueller, Bryon A.; Sowell, Elizabeth R.; Mattson, Sarah N.; Coles, Claire D.; Kable, Julie A.; Jones, Kenneth L.; Boys, Christopher J.; Lee, Susanne; Lim, Kelvin O.; Riley, Edward P.; Wozniak, Jeffrey R. | Two-year cortical trajectories are abnormal in children and adolescents with prenatal alcohol exposure | Irrelevant results |
| 1117 | 2007 | Kunii, Osamu | The Okinawa Infectious Diseases Initiative | Irrelevant results |
| 1118 | 2009 | Zender, Robynn; Olshansky, Ellen | Promoting Wellness in Women Across the Life Span | Irrelevant results |
| 1119 | 2002 | Islam, Anwar; Zaffar Tahir, M. | Health sector reform in South Asia: new challenges and constraints | Irrelevant results |
| 1120 | 2017 | McCord, Gordon C.; Conley, Dalton; Sachs, Jeffrey D. | Malaria ecology, child mortality & fertility | Irrelevant results |
| 1121 | 2020 | Vahedi, Luissa; Bartels, Susan; Lee, Sabine | “His Future will not be Bright”: A qualitative analysis of mothers’ lived experiences raising peacekeeper-fathered children in Haiti | Irrelevant results |
| 1122 | 2005 | Phipatanakul, Wanda | Allergic Rhinoconjunctivitis: Epidemiology | Irrelevant results |
| 1123 | 2015 | Robertson, Eva K. | “To be taken seriously” : women's reflections on how migration and resettlement experiences influence their healthcare needs during childbearing in Sweden | Irrelevant results |
| 1124 | 2001 | Flesckler, Robin G.; Knight, Sally A.; Ray, Glenda | Severity Risk Adjusting Relating to Obstetric Outcomes DRG Assignment, and Reimbursement | Irrelevant results |
| 1125 | 2011 | Mummert, Amanda; Esche, Emily; Robinson, Joshua; Armelagos, George J. | Stature and robusticity during the agricultural transition: Evidence from the bioarchaeological record | Irrelevant results |
| 1126 | 2002 | Stehman-Breen, Catherine O.; Levine, Richard J.; Qian, Cong; Morris, Cynthia D.; Catalano, Patrick M.; Curet, Luis B.; Sibai, Baha M. | Increased risk of preeclampsia among nulliparous pregnant women with idiopathic hematuria | Irrelevant results |
| 1127 | 2005 | Newton, Edward R. | Preterm Labor, Preterm Premature Rupture of Membranes, and Chorioamnionitis | Irrelevant results |
| 1128 | 2004 | Dhala, Atiya; Pinsker, Kenneth; Prezant, David J. | Respiratory health consequences of environmental tobacco smoke | Irrelevant results |
| 1129 | 2009 | Ewig, Christina; Bello, Amparo Hernández | Gender equity and health sector reform in Colombia: Mixed state-market model yields mixed results | Irrelevant results |
| 1130 | 2004 | Brennan, Lance; McDonald, John; Shlomowitz, Ralph | Infant feeding practices and chronic child malnutrition in the Indian states of Karnataka and Uttar Pradesh | Irrelevant results |
| 1131 | 2015 | Kajeepeta, Sandhya; Gelaye, Bizu; Jackson, Chandra L.; Williams, Michelle A. | Adverse childhood experiences are associated with adult sleep disorders: a systematic review | Irrelevant results |
| 1132 | 2013 | Walker, S. Y.; Pierre, R. B.; Christie, C. D. C.; Chang, S. M. | Neurocognitive function in HIV-positive children in a developing country | Irrelevant results |
| 1133 | 2013 | Sockol, Laura E.; Epperson, C. Neill; Barber, Jacques P. | Preventing postpartum depression: A meta-analytic review | Irrelevant results |
| 1134 | 2003 | Putnam, FRANK W. | Ten-Year Research Update Review: Child Sexual Abuse | Irrelevant results |
| 1135 | 2014 | Teng, Kathryn; Acheson, Louise S. | Genomics in Primary Care Practice | Irrelevant results |
| 1136 | 2009 | Nguyen, Carolyn T.; Tran, Tram T. | Hepatitis Vaccination and Prophylaxis | Irrelevant results |
| 1137 | 2018 | Hogan, Vijaya K.; de Araujo, Edna M.; Caldwell, Kia L.; Gonzalez-Nahm, Sarah N.; Black, Kristin Z. | “We black women have to kill a lion everyday”: An intersectional analysis of racism and social determinants of health in Brazil | Irrelevant results |
| 1138 | 2009 | White-Traut, Rosemary; Norr, Kathleen | An Ecological Model for Premature Infant Feeding | Irrelevant results |
| 1139 | 2009 | Charlton, Karen E.; Kawana, Beatrice M.; Hendricks, Michael K. | An assessment of the effectiveness of growth monitoring and promotion practices in the Lusaka district of Zambia | Irrelevant results |
| 1140 | 2016 | Zar, Heather J; Barnett, Whitney; Stadler, Attie; Gardner-Lubbe, Sugnet; Myer, Landon; Nicol, Mark P | Aetiology of childhood pneumonia in a well vaccinated South African birth cohort: a nested case-control study of the Drakenstein Child Health Study | Irrelevant results |
| 1141 | 2020 | Zerbo, Alexandre; Castro Delgado, Rafael; Arcos González, Pedro | Aedes-borne viral infections and risk of emergence/resurgence in Sub-Saharan African urban areas | Irrelevant results |
| 1142 | 2021 | Damhuis, Stefanie E.; Ganzevoort, Wessel; Gordijn, Sanne J. | Abnormal Fetal Growth: Small for Gestational Age, Fetal Growth Restriction, Large for Gestational Age: Definitions and Epidemiology | Irrelevant results |
| 1143 | 2018 | Yuma-Guerrero, Paula; Orsi, Rebecca; Lee, Ping-Tzu; Cubbin, Catherine | A systematic review of socioeconomic status measurement in 13 years of U.S. injury research | Irrelevant results |
| 1144 | 2021 | Lopreite, Milena; Puliga, Michelangelo; Riccaboni, Massimo; De Rosis, Sabina | A social network analysis of the organizations focusing on tuberculosis, malaria and pneumonia | Irrelevant results |
| 1145 | 2017 | Nguyen, Jennifer | A Literature Review of Alternative Therapies for Postpartum Depression | Irrelevant results |
| 1146 | 2008 | Li, Xiaoming; Naar-King, Sylvie; Barnett, Douglas; Stanton, Bonita; Fang, Xiaoyi; Thurston, Celia | A Developmental Psychopathology Framework of the Psychosocial Needs of Children Orphaned by HIV | Irrelevant results |
| 1147 | 2007 | Patel, Jayendra K.; Ralston, Timothy E.; Wong, Eileen | 4 Drugs of abuse | Irrelevant results |
| 1148 | 2009 | Rööst, M.; Liljestrand, J.; Essén, B. | O807 Maternal near-miss morbidity in La Paz, Bolivia: Frequencies and the importance of antenatal care | Excluded for out of context |
| 1149 | 2012 | Tuncalp, O.; Hindin, M.; Adu-Bonsaffoh, K.; Adanu, R. | O704 MATERNAL NEAR MISS: RESULTS FROM A HOSPITAL-BASED STUDY IN ACCRA, GHANA | Irrelevant results |
| 1150 | 2012 | Nelissen, E.; Ersdal, H. L.; Mduma, E.; Evjen-Olsen, B.; van Roosmalen, J.; Stekelenburg, J. | O490 MATERNAL NEAR MISS AND MORTALITY IN A RURAL REFERRAL HOSPITAL IN NORTHERN TANZANIA USING THE WHO NEAR MISS CRITERIA | Irrelevant results |
| 1151 | 2009 | Pattinson, Robert | Near miss audit in obstetrics | Irrelevant results |
| 1152 | 2017 | Owolabi, Onikepe O.; Cresswell, Jenny A.; Vwalika, Bellington; Osrin, David; Filippi, Veronique | Incidence of abortion-related near-miss complications in Zambia: cross-sectional study in Central, Copperbelt and Lusaka Provinces | Irrelevant results |
| 1153 | 2009 | Pattinson, R. | I264 Changing practice using near-miss audits | Irrelevant results |
| 1154 | 2017 |  | Index | Irrelevant results |
| 1155 | 2015 |  | Free Communication (Oral) Presentations | Irrelevant results |
| 1156 | 2013 | McMaster, Romy-Leigh; Wei, Shu-Qin; Fraser, William D. | Chapter 22 - Labor and Delivery | Irrelevant results |
| 1157 | 2017 |  | Abstracts of free papers presented at the annual meeting of the Obstetric Anaesthetists' Association, Brussels, May 18-X 2017 | Irrelevant results |
| 1158 | 2015 |  | Poster Presentations | Irrelevant results |
| 1159 | 1998 | Kwast, Barbara E. | Quality of care in reproductive health programmes: Monitoring and evaluation of quality improvement | Irrelevant results |
| 1160 | 2013 |  | International News - December | Irrelevant results |
| 1161 | 2004 | Thompson, Anne | Bridging the gap: teaching ethics in midwifery practice | Irrelevant results |
| 1162 | 2012 | Zanconato, G.; Cavaliere, E.; Iacovella, C.; Angeli, A.; Mariotto, O.; Franchi, M. | O320 OBSTETRIC NEAR MISS IN A TERTIARY CARE HOSPITAL OF NORTHERN ITALY: IMPACT ON FETO-MATERNAL HEALTH | Irrelevant results |
| 1163 | 2012 | Elmir, Rakime; Schmied, Virginia; Jackson, Debra; Wilkes, Lesley | Between life and death: Women’s experiences of coming close to death, and surviving a severe postpartum haemorrhage and emergency hysterectomy | Irrelevant results |
| 1164 | 2015 | Vogel, Joshua P; Betrán, Ana Pilar; Vindevoghel, Nadia; Souza, João Paulo; Torloni, Maria Regina; Zhang, Jun; and al | Use of the Robson classification to assess caesarean section trends in 21 countries: a secondary analysis of two WHO multicountry surveys | Irrelevant results |
| 1165 | 2009 | Dorssers, E.; Wassen, M.; Winkens, B.; Roumen, F. | O809 Epidural analgesia during labor: Effect on maternal and neonatal temperature | Irrelevant results |
| 1166 | 2012 | Neyatani, N.; Yamaguchi, N.; Takagi, H.; Fujii, R.; Makinoda, S. | O493 CLINICAL STUDY ON FERTILITY IN WOMEN RECEIVED RENAL TRANSPLANTATION | Irrelevant results |
| 1167 | 2021 | Romero, Roberto | Giants in Obstetrics and Gynecology Series: a profile of Robert L. Goldenberg, MD | Irrelevant results |
| 1168 | 2012 | Hu, Y.; Qu, P. | O318 HPV TYPING DETECTION BY PYROSEQUENCING | Irrelevant results |
| 1169 | 2009 | Rosenberg, R.; Rosenberg, A. | O808 Women with major depression treated with the SNRI duluxetine prior to conception through entire pregnancy | Irrelevant results |
| 1170 | 2012 | Natale, A.; Bosco, P.; Bonato, S.; Alabiso, G.; Austoni, V. | O489 EXPERIENCE OF A UNIT FOR THE PATHOLOGY OF EARLY PREGNANCY AND ECTOPIC PREGNANCY | Irrelevant results |
| 1171 | 2012 | Nargund, A. M.; Rich, D. | O488 MULTIPLE SCLEROSIS IN PREGNANCY | Irrelevant results |
| 1172 | 2015 | de Miguel Sesmero, José Ramón; Muñoz Cacho, Pedro; Solano, Alberto Muñoz; Odriozola Feu, Juan M.; González Gómez, Mónica; Puertas Prieto, Alberto; González González, Nieves L.; Lailla Vicens, Josep María | Mortalidad materna en España en el periodo 2010-2012: resultados de la encuesta de la Sociedad Española de Ginecología (SEGO) | Irrelevant results |
| 1173 | 2009 | Rokita, W.; Stanislawska, M.; Nowak-Markwitz, E.; Kedzia, W.; Spaczyński, M.; Karowicz-Bilinska, A.; Bednarek, W. | O806 Diagnostic hysteroscopy in women with endometrial hyperplasia and endometrial cancer – 9 years of experience, analysis of 142 cases | Irrelevant results |
| 1174 | 2012 | Nanayakkara, K. K.; Rodrigo, G.; Nanayakkara, C. D. | O487 PRE-NATAL DIAGNOSIS OF THALASSAEMIA BY AMNIOCENTESIS: KANDY THALASSAEMIA CENTRE, SRI LANKA | Irrelevant results |
| 1175 | 2013 | Dumont, Alexandre; Bouvier-Colle, Marie-Hélène | Care assessment's difficult relation with maternal mortality | Irrelevant results |
| 1176 | 2012 | Huda, F. A.; Ford, E. R.; Johnston, H. | O319 IMPLEMENTING NEW INDICATORS TO DOCUMENT HEALTH SYSTEMS CAPACITY TO REDUCE ABORTION-RELATED MATERNAL MORTALITY IN BANGLADESH | Irrelevant results |
| 1177 | 2016 | Mullan, Zoë | Stillbirths: still neglected? | Irrelevant results |
| 1178 | 2020 | Ahmed, Salma A. E.; Wangamati, Cynthia Khamala; Thorsen, Viva Combs | Childbirth experiences of Sudanese women living with obstetric fistula – A qualitative study | Irrelevant results |
| 1179 | 2016 |  | Subject Index | Irrelevant results |
| 1180 | 2021 | Brizuela, Vanessa; Cuesta, Cristina; Bartolelli, Gino; Abdosh, Abdulfetah Abdulkadir; Abou Malham, Sabina; Assarag, Bouchra; and | Availability of facility resources and services and infection-related maternal outcomes in the WHO Global Maternal Sepsis Study: a cross-sectional study | Irrelevant results |
| 1181 | 2016 |  | Subject Index | Irrelevant results |
| 1182 | 2020 | van den Broek, Nynke R. | 16 - Maternal and Newborn Health | Irrelevant results |
| 1183 | 2016 | Kerr, Robbie; Eckert, Linda O.; Winikoff, Beverly; Durocher, Jill; Meher, Shireen; Fawcus, Sue; Mundle, Shuchita; Mol, Ben; Arulkumaran, Sabaratnam; Khan, Khalid; Wandwabwa, Julius; Kochhar, Sonali; Weeks, Andrew | Postpartum haemorrhage: Case definition and guidelines for data collection, analysis, and presentation of immunization safety data | Irrelevant results |
| 1184 | 2012 | Trivellizzi, I. N.; Ricci, C.; Conte, C.; Scarciglia, M. L.; Scirpa, P.; Scambia, G.; De Vincenzo, R. | O702 A 4-YEAR EXPERIENCE OF CATCH-UP HPV VACCINATION AMONG 12–26 YEARS AGED WOMEN AND PILOT FOLLOW UP DATA | Irrelevant results |
| 1185 | 2009 | Paltieli, Y. | I263 A new technique combining ultrasound and position tracker for non-invasive assessment of labour progress | Irrelevant results |
| 1186 | 2015 |  | Subject Index | Irrelevant results |
| 1187 | 2009 | Pattinson, R. | I265 Essential steps in the management of obstetric emergencies: A training strategy for health professionals | Irrelevant results |
| 1188 | 2010 | Pattinson, Robert | Improving emergency obstetric care | Irrelevant results |
| 1189 | 2020 | Aboungo, Victoria; Kaselitz, Elizabeth; Aborigo, Raymond; Williams, John; James, Kat; Moyer, Cheryl | Why do community members believe mothers and babies are dying? Behavioral versus situational attribution in rural northern Ghana | Irrelevant results |
| 1190 | 2009 | Pattinson, R. | I266 Improving quality of care for MNH | Irrelevant results |
| 1191 | 2012 | Palermo, T. M.; Troncoso, E.; Schiavon, R.; Sanhueza, P.; Huebner, R. Meiner | O703 EVALUATION OF ABORTION SERVICES AND PATIENT CHARACTERISTICS IN A MEXICO CITY PUBLIC HOSPITAL AFTER LEGALIZATION | Irrelevant results |
| 1192 | 2014 |  | Subject Index | Irrelevant results |
| 1193 | 2017 | Nathan, Robert O.; Swanson, Jonathan O.; Swanson, David L.; McClure, Elizabeth M.; Bolamba, Victor Lokomba; Lokangaka, Adrien; and al | Evaluation of Focused Obstetric Ultrasound Examinations by Health Care Personnel in the Democratic Republic of Congo, Guatemala, Kenya, Pakistan, and Zambia | Irrelevant results |
| 1194 | 2020 | Påfs, Jessica; Rulisa, Stephen; Klingberg-Allvin, Marie; Binder-Finnema, Pauline; Musafili, Aimable; Essén, Birgitta | Implementing the liberalized abortion law in Kigali, Rwanda: Ambiguities of rights and responsibilities among health care providers | Irrelevant results |
| 1195 | 2009 | Pattinson, R. | I267 Reducing intrapartum stillbirths – Experience in South Africa | Irrelevant results |
| 1196 | 2005 | Subtil, D.; Sommé, A.; Ardiet, E.; Depret-Mosser, S. | Hémorragies du post-partum : fréquence, conséquences en termes de santé et facteurs de risque avant l’accouchement | Irrelevant results |
| 1197 | 2015 | Malloy-Weir, Leslie J.; Charles, Cathy; Gafni, Amiram; Entwistle, Vikki A. | Empirical relationships between health literacy and treatment decision making: A scoping review of the literature | Irrelevant results |
| 1198 | 2021 | Schwartz, Nadav; Mhajna, Muhammad; Moody, Heather L.; Zahar, Yael; Shkolnik, Ketty; Reches, Amit; Lowery, Curtis L. | Novel Uterine Contraction Monitoring to Enable Remote, Self-administered Non-stress Testing | Irrelevant results |
| 1199 | 2020 | Psara, Elina; Pentieva, Kristina; Ward, Mary; McNulty, Helene | Critical review of nutrition, blood pressure and risk of hypertension through the lifecycle: do B vitamins play a role? | Irrelevant results |
| 1200 | 2004 | Karnad, Dilip R.; Guntupalli, Kalpalatha K. | Critical illness and pregnancy: review of a global problem | Irrelevant results |
| 1201 | 2018 | Rao, Swati; Jim, Belinda | Acute Kidney Injury in Pregnancy: The Changing Landscape for the 21st Century | Irrelevant results |
| 1202 | 2021 | Erinc, Abigail; Davis, Melinda B.; Padmanabhan, Vasantha; Langen, Elizabeth; Goodrich, Jaclyn M. | Considering environmental exposures to per- and polyfluoroalkyl substances (PFAS) as risk factors for hypertensive disorders of pregnancy | Irrelevant results |
| 1203 | 2013 | Råssjö, Eva Britta; Byrskog, Ulrika; Samir, Raghad; Klingberg-Allvin, Marie | Somali women’s use of maternity health services and the outcome of their pregnancies: A descriptive study comparing Somali immigrants with native-born Swedish women | Irrelevant results |
| 1204 | 2012 | Callaghan, William M. | Overview of Maternal Mortality in the United States | Irrelevant results |
| 1205 | 2015 | Sentilhes, L.; Lasocki, S.; Ducloy-Bouthors, A. S.; Deruelle, P.; Dreyfus, M.; Perrotin, F.; Goffinet, F.; Deneux-Tharaux, C. | Tranexamic acid for the prevention and treatment of postpartum haemorrhage | Irrelevant results |
| 1206 | 2019 | Leavitt, Karla; Običan, Sarah; Yankowitz, Jerome | Treatment and Prevention of Hypertensive Disorders During Pregnancy | Irrelevant results |
| 1207 | 2017 | Tsai, Hsiu-Ting; Wu, Chia-Hsun | Vaginal birth after cesarean section—The world trend and local experience in Taiwan | Irrelevant results |
| 1208 | 2008 | Wylie, Blair J.; Mirza, Fadi G. | Cesarean Delivery in the Developing World | Irrelevant results |
| 1209 | 2020 | Falletta, Lynn; Abbruzzese, Stephanie; Fischbein, Rebecca; Shura, Robin; Eng, Abbey; Alemagno, Sonia | Work Reentry After Childbirth: Predictors of Self-Rated Health in Month One Among a Sample of University Faculty and Staff | Irrelevant results |
| 1210 | 2015 | Smid, Marcela; Ahmed, Yusuf; Ivester, Thomas | Special considerations—Induction of labor in low-resource settings | Irrelevant results |
| 1211 | 2020 | Lee King, Patricia Ann; Henderson, Zsakeba T.; Borders, Ann E. B. | Advances in Maternal Fetal Medicine: Perinatal Quality Collaboratives Working Together to Improve Maternal Outcomes | Irrelevant results |
| 1212 | 2017 | Schneider, Patrick D.; Sabol, Bethany A.; Lee King, Patricia Ann; Caughey, Aaron B.; Borders, Ann E. B. | The Hard Work of Improving Outcomes for Mothers and Babies: Obstetric and Perinatal Quality Improvement Initiatives Make a Difference at the Hospital, State, and National Levels | Irrelevant results |
| 1213 | 2019 | Wouldes, Trecia A.; Lester, Barry M. | Stimulants: How big is the problem and what are the effects of prenatal exposure? | Irrelevant results |
| 1214 | 2021 | Smith, Julia; Velez, Maria P.; Dayan, Natalie | Infertility, Infertility Treatment and Cardiovascular Disease: An Overview | Irrelevant results |
| 1215 | 2009 | McLintock, Claire | Obstetric haemorrhage | Irrelevant results |
| 1216 | 2016 | Cain, Mary Ashley; Louis, Judette M. | Sleep Disordered Breathing and Adverse Pregnancy Outcomes | Irrelevant results |
| 1217 | 2021 | Combs, C. Andrew; Allbert, John R.; Hameed, Afshan B.; Main, Elliott; Taylor, Isabel; Allen, Christie | Society for Maternal-Fetal Medicine Special Statement: A Quality Metric for Evaluating Timely Treatment of Severe Hypertension | Irrelevant results |
| 1218 | 2019 | Uwubamwen, Nosakhare A.; Verma, Dipali | Antenatal anaesthetic assessment of high risk obstetric patients | Irrelevant results |
| 1219 | 2012 | Ghulmiyyah, Labib; Sibai, Baha | Maternal Mortality From Preeclampsia/Eclampsia | Irrelevant results |
| 1220 | 2000 |  | Abstracts | Irrelevant results |
| 1221 | 2011 | Fernandes, Cláudia Regina; Fonseca, Neuber Martins; Rosa, Deise Martins; Simões, Cláudia Marquez; Duarte, Nádia Maria da Conceição | Brazilian Society of Anesthesiology Recommendations for Safety in Regional Anesthesia | Irrelevant results |
| 1222 | 2013 |  | A Review of JACC Journal Articles on the Topic of Cardiac Imaging: 2011–2012 | Irrelevant results |
| 1223 | 2010 | Fiocchi, Alessandro; Brozek, Jan; Schünemann, Holger; Bahna, Sami L; von Berg, Andrea; Beyer, Kirsten; and al | World Allergy Organization (WAO) Diagnosis and Rationale for Action against Cow's Milk Allergy (DRACMA) Guidelines | Irrelevant results |
| 1224 | 2000 | World Health Organization | Severe falciparum malaria | Irrelevant results |
| 1225 | 2001 | August, Gerald J.; Realmuto, George M.; Winters, Ken C.; Hektner, Joel M. | Prevention of adolescent drug abuse: Targeting high-risk children with a multifaceted intervention model—The Early Risers “Skills for Success” Program | Irrelevant results |
| 1226 | 2008 | Calañas-Continente, Alfonso; José Arrizabalaga, Juan; Caixàs, Assumpta; Cuatrecasas, Guillem; Jesús Díaz-Fernández, M.; Pablo García-Luna, Pedro; and al | Comorbilidades del exceso ponderal en el adolescente | Irrelevant results |
| 1227 | 2015 |  | Proceedings of the 16th World Meeting on Sexual Medicine, São Paulo, Brazil, October 8–12, 2014 | Irrelevant results |
| 1228 | 2010 | Nolan, Jerry P.; Hazinski, Mary Fran; Billi, John E.; Boettiger, Bernd W.; Bossaert, Leo; de Caen, Allan R.; and al | Part 1: Executive summary: 2010 International Consensus on Cardiopulmonary Resuscitation and Emergency Cardiovascular Care Science With Treatment Recommendations | Irrelevant results |
| 1229 | 2016 | Dittrich, Christian; Kosty, Michael; Jezdic, Svetlana; Pyle, Doug; Berardi, Rossana; Bergh, Jonas; and al | ESMO / ASCO Recommendations for a Global Curriculum in Medical Oncology Edition 2016 | Irrelevant results |
| 1230 | 2003 | Bartosz, Grzegorz | Total antioxidant capacity | Irrelevant results |
| 1231 | 2009 |  | APhA2009 abstracts of contributed papers | Irrelevant results |
| 1232 | 2005 | Gori, Stefania; Porrozzi, Stella; Roila, Fausto; Gatta, Gemma; De Giorgi, Ugo; Marangolo, Maurizio | Germ cell tumours of the testis | Irrelevant results |
| 1233 | 2003 |  | 2003 Abstracts of Contributed Papers | Irrelevant results |
| 1234 | 2015 | Gürtler, Ricardo E.; Cardinal, M. V. | Reservoir host competence and the role of domestic and commensal hosts in the transmission of Trypanosoma cruzi | Irrelevant results |
| 1235 | 2002 | Mayer, David A; Fried, Bernard | Aspects of human parasites in which surgical intervention may be important | Irrelevant results |
| 1236 | 2016 | Hossny, Elham; Rosario, Nelson; Lee, Bee Wah; Singh, Meenu; El-Ghoneimy, Dalia; Soh, Jian Yi; Le Souef, Peter | The use of inhaled corticosteroids in pediatric asthma: update | Irrelevant results |
| 1237 | 2011 | Yu-Wai-Man, Patrick; Griffiths, Philip G.; Chinnery, Patrick F. | Mitochondrial optic neuropathies – Disease mechanisms and therapeutic strategies | Irrelevant results |
| 1238 | 2002 | Hibbeln, Joseph R.; Makino, Kevin K. | Chapter 5 Omega-3 fats in depressive disorders and violence: the context of evolution and cardiovascular health | Irrelevant results |
| 1239 | 2005 | Brown, Deborah L. | Congenital bleeding disorders | Irrelevant results |
| 1240 | 2005 | Bleyer, Archie | The adolescent and young adult gap in cancer care and outcome | Irrelevant results |
| 1241 | 1999 | Ernst, John M.; Cacioppo, John T. | Lonely hearts: Psychological perspectives on loneliness | Irrelevant results |
| 1242 | 2002 | Vargas, Claudia MarÍa | Women in Sustainable Development: Empowerment through Partnerships for Healthy Living | Irrelevant results |
| 1243 | 2006 | Ireland, Dolly | Unique Concerns of the Pediatric Surgical Patient: Pre-, Intra-, and Postoperatively | Irrelevant results |
| 1244 | 2003 | Tedstone, Josephine E; Tarrier, Nicholas | Posttraumatic stress disorder following medical illness and treatment | Irrelevant results |
| 1245 | 2001 | Leventhal, Tama; Brooks-Gunn, Jeanne | Changing neighborhoods and child well-being: Understanding how children may be affected in the coming century | Irrelevant results |
| 1246 | 2013 | Gonzalez-Campoy, J. Michael; Castorino, Kristin; Ebrahim, Ayesha; Hurley, Dan; Jovanovic, Lois; Mechanick, Jeffrey I.; and al | Clinical Practice Guidelines for Healthy Eating for the Prevention and Treatment of Metabolic and Endocrine Diseases in Adults: Cosponsored by the American Association of Clinical Endocrinologists/The American College of Endocrinology and the Obesity Society | Irrelevant results |
| 1247 | 2001 | Higgins, Daryl J; McCabe, Marita P | Multiple forms of child abuse and neglect: adult retrospective reports | Irrelevant results |
| 1248 | 2018 | Sharif, Rabab; Bak-Nielsen, Sashia; Hjortdal, Jesper; Karamichos, Dimitrios | Pathogenesis of Keratoconus: The intriguing therapeutic potential of Prolactin-inducible protein | Irrelevant results |
| 1249 | 2002 | Winters, NANCY C.; Myers, KATHLEEN; Proud, LAURA | Ten-Year Review of Rating Scales. III: Scales Assessing Suicidality, Cognitive Style, and Self-Esteem | Irrelevant results |
| 1250 | 2015 | Jacobson, Terry A.; Maki, Kevin C.; Orringer, Carl E.; Jones, Peter H.; Kris-Etherton, Penny; Sikand, Geeta; and al | National Lipid Association Recommendations for Patient-Centered Management of Dyslipidemia: Part 2 | Irrelevant results |
| 1251 | 2011 | Patel, Kavita Kirankumar; Caramelli, Bruno; Silva, Mauricio Rocha e | Original research articles on the cardiopulmonary system recently appeared in Brazilian clinical and surgical journals | Irrelevant results |
| 1252 | 2017 |  | Abstracts | Irrelevant results |
| 1253 | 2001 | Lauritzen, L; Hansen, H. S; Jørgensen, M. H; Michaelsen, K. F | The essentiality of long chain n-3 fatty acids in relation to development and function of the brain and retina | Irrelevant results |
| 1254 | 2003 | Harper, Caroline; Marcus, Rachel; Moore, Karen | Enduring Poverty and the Conditions of Childhood: Lifecourse and Intergenerational Poverty Transmissions | Irrelevant results |
| 1255 | 2019 | Perera, F.; Ashrafi, A.; Kinney, P.; Mills, D. | Towards a fuller assessment of benefits to children's health of reducing air pollution and mitigating climate change due to fossil fuel combustion | Irrelevant results |
| 1256 | 2012 | Greydanus, Donald E.; Pratt, Helen D.; Patel, Dilip R. | Concepts of Contraception for Adolescent and Young Adult Women with Chronic Illness and Disability | Irrelevant results |
| 1257 | 2005 | Wise, Gilbert J.; Roorda, Andrew K.; Kalter, Robert | Male breast disease1 | Irrelevant results |
| 1258 | 2014 | Maenz, M.; Schlüter, D.; Liesenfeld, O.; Schares, G.; Gross, U.; Pleyer, U. | Ocular toxoplasmosis past, present and new aspects of an old disease | Irrelevant results |
| 1259 | 2018 | Epel, Elissa S.; Crosswell, Alexandra D.; Mayer, Stefanie E.; Prather, Aric A.; Slavich, George M.; Puterman, Eli; Mendes, Wendy Berry | More than a feeling: A unified view of stress measurement for population science | Irrelevant results |
| 1260 | 2011 | Patel, Kavita Kirankumar; Caramelli, Bruno; e Silva, Mauricio Rocha | Original research articles on the cardiopulmonary system recently appeared in Brazilian clinical and surgical journals | Irrelevant results |
| 1261 | 2003 | Batliwala, Srilatha; Reddy, Amulya K. N. | Energy for women and women for energy (engendering energy and empowering women)1 1A rudimentary version of this paper was presented at the Brainstorming Meeting of ENERGIA: Women and Energy Network on June 4-5, 1996, at the University of Twente, Enschede, the Netherlands (cf. [Batliwala and Reddy, 1996]). | Irrelevant results |
| 1262 | 2007 | Arosarena, Oneida A. | Cleft Lip and Palate | Irrelevant results |
| 1263 | 2016 | di Mauro, Giuseppe; Bernardini, Roberto; Barberi, Salvatore; Capuano, Annalisa; Correra, Antonio; de’ Angelis, Gian Luigi and al | Prevention of food and airway allergy: consensus of the Italian Society of Preventive and Social Paediatrics, the Italian Society of Paediatric Allergy and Immunology, and Italian Society of Pediatrics | Irrelevant results |
| 1264 | 2004 | Althuisius, Sietske; Dekker, Gus | Controversies regarding cervical incompetence, short cervix, and the need for cerclage | Irrelevant results |
| 1265 | 2004 | Butt, Adeel A; Aldridge, Kenneth E; Sanders, Charles V | Infections related to the ingestion of seafood Part I: viral and bacterial infections | Irrelevant results |
| 1266 | 2006 | Lobel, Melissa K.; Somasundaram, Priya; Morton, Cynthia C. | The Genetic Heterogeneity of Uterine Leiomyomata | Irrelevant results |
| 1267 | 2017 | Eggert, Julie | Genetics and Genomics in Oncology Nursing: What Does Every Nurse Need to Know? | Irrelevant results |
| 1268 | 2013 | Markle, William; Conti, Tracey; Kad, Manjusha | Sexually Transmitted Diseases | Irrelevant results |
| 1269 | 2005 | Bloom, David E.; Zaidi, Anita K. M.; Yeh, Ethan | The demographic impact of biomass fuel use | Irrelevant results |
| 1270 | 2011 | John, T Jacob; Dandona, Lalit; Sharma, Vinod P; Kakkar, Manish | Continuing challenge of infectious diseases in India | Irrelevant results |
| 1271 | 2012 | Laslett, Lawrence J.; Alagona, Peter; Clark, Bernard A.; Drozda, Joseph P.; Saldivar, Frances; Wilson, Sean R.; Poe, Chris; Hart, Menolly | The Worldwide Environment of Cardiovascular Disease: Prevalence, Diagnosis, Therapy, and Policy Issues: A Report From the American College of Cardiology | Irrelevant results |
| 1272 | 2009 | Reading, Richard; Bissell, Susan; Goldhagen, Jeffrey; Harwin, Judith; Masson, Judith; Moynihan, Sian; Parton, Nigel; Pais, Marta Santos; Thoburn, June; Webb, Elspeth | Promotion of children's rights and prevention of child maltreatment | Irrelevant results |
| 1273 | 2005 | Sharma, Surendra Kumar; Mohan, Alladi; Sharma, Anju; Mitra, Dipendra Kumar | Miliary tuberculosis: new insights into an old disease | Irrelevant results |
| 1274 | 2018 | McEwen, Bruce S. | Redefining neuroendocrinology: Epigenetics of brain-body communication over the life course | Irrelevant results |
| 1275 | 2012 | Lea, Jayanthi S.; Lin, Ken Y. | Cervical Cancer | Irrelevant results |
| 1276 | 2010 | Broder, Samuel | The development of antiretroviral therapy and its impact on the HIV-1/AIDS pandemic | Irrelevant results |
| 1277 | 2008 | Abubaker, Roohi; Alaerts, Maaike; Allman, Ava-Ann; Barnett, Jennifer; Belujon, Pauline; and al | Summary of the 1st Schizophrenia International Research Society Conference oral sessions, Venice, Italy, June 21–25, 2008: The rapporteur reports | Irrelevant results |
| 1278 | 2010 | Jen, Melinda; Yan, Albert C. | Syndromes associated with nutritional deficiency and excess | Irrelevant results |
| 1279 | 2018 | Stovner, Lars Jacob; Nichols, Emma; Steiner, Timothy J; Abd-Allah, Foad; Abdelalim, Ahmed; Al-Raddadi, Rajaa M; and al | Global, regional, and national burden of migraine and tension-type headache, 1990–2016: a systematic analysis for the Global Burden of Disease Study 2016 | Irrelevant results |
| 1280 | 2010 | Molini, Vasco; Nubé, Maarten; van den Boom, Bart | Adult BMI as a Health and Nutritional Inequality Measure: Applications at Macro and Micro Levels | Irrelevant results |
| 1281 | 2006 | Ustianowski, Andrew P; Lawn, Stephen D; Lockwood, Diana NJ | Interactions between HIV infection and leprosy: a paradox | Irrelevant results |
| 1282 | 2015 | Sullivan, Richard; Alatise, Olusegun Isaac; Anderson, Benjamin O; Audisio, Riccardo; Autier, Philippe; Aggarwal, Ajay; and al | Global cancer surgery: delivering safe, affordable, and timely cancer surgery | Irrelevant results |
| 1283 | 2012 | Esquivel-Santoveña, Esteban Eugenio; Dixon, Louise | Investigating the true rate of physical intimate partner violence: A review of nationally representative surveys | Irrelevant results |
| 1284 | 2012 | Keeton, Victoria; Soleimanpour, Samira; Brindis, Claire D. | School-Based Health Centers in an Era of Health Care Reform: Building on History | Irrelevant results |
| 1285 | 2013 | Ruiz-Núñez, Begoña; Pruimboom, Leo; Dijck-Brouwer, D. A. Janneke; Muskiet, Frits A. J. | Lifestyle and nutritional imbalances associated with Western diseases: causes and consequences of chronic systemic low-grade inflammation in an evolutionary context | Irrelevant results |
| 1286 | 2005 | Sarfaty, Suzanne; Arnold, L. Kristian | Preparing for international medical service | Irrelevant results |
| 1287 | 2004 | Buford, Terry A | Transfer of asthma management responsibility from parents to their school-age children | Irrelevant results |
| 1288 | 2017 | McKay, Kyla A.; Jahanfar, Shayesteh; Duggan, Tom; Tkachuk, Stacey; Tremlett, Helen | Factors associated with onset, relapses or progression in multiple sclerosis: A systematic review | Irrelevant results |
| 1289 | 2008 | Freeman, JENNIFER B.; Garcia, ABBE M.; Coyne, LISA; Ale, CHELSEA; Przeworski, AMY; Himle, MICHAEL; Compton, SCOTT; Leonard, HENRIETTA L. | Early Childhood OCD: Preliminary Findings From a Family-Based Cognitive-Behavioral Approach | Irrelevant results |
| 1290 | 2010 | Levy, Lauren B.; O'Hara, Michael W. | Psychotherapeutic interventions for depressed, low-income women: A review of the literature | Irrelevant results |
| 1291 | 2003 | Kalter, Harold | Teratology in the 20th century: Environmental causes of congenital malformations in humans and how they were established | Irrelevant results |
| 1292 | 2009 | Sim, Myung Shin K.; Cumberland, William G.; Duan, Naihua; Bryson, Yvonne J. | Modeling vertical transmission of HIV: Imperfect vaccines can be of benefit | Irrelevant results |
| 1293 | 2015 | Sathe, Kiran Prakash; Nagral, Aabha | Metabolic liver disease in developing world with special reference to Indian children – A review | Irrelevant results |
| 1294 | 2013 | Wosu, Adaeze C.; Valdimarsdóttir, Unnur; Shields, Alexandra E.; Williams, David R.; Williams, Michelle A. | Correlates of cortisol in human hair: implications for epidemiologic studies on health effects of chronic stress | Irrelevant results |
| 1295 | 2015 | Rocha, Rudi; Soares, Rodrigo R. | Water scarcity and birth outcomes in the Brazilian semiarid | Irrelevant results |
| 1296 | 2005 | Van Oostdam, J.; Donaldson, S. G.; Feeley, M.; Arnold, D.; Ayotte, P.; Bondy, G.; Chan, L.; Dewaily, É.; Furgal, C. M.; Kuhnlein, H.; Loring, E.; Muckle, G.; Myles, E.; Receveur, O.; Tracy, B.; Gill, U.; Kalhok, S. | Human health implications of environmental contaminants in Arctic Canada: A review | Irrelevant results |
| 1297 | 2021 | Hricak, Hedvig; Abdel-Wahab, May; Atun, Rifat; Lette, Miriam Mikhail; Paez, Diana; Brink, James A; and al | Medical imaging and nuclear medicine: a Lancet Oncology Commission | Irrelevant results |
| 1298 | 2015 | Cohen, Jonathan; Vincent, Jean-Louis; Adhikari, Neill K J; Machado, Flavia R; Angus, Derek C; Calandra, Thierry; Jaton, Katia; Giulieri, Stefano; Delaloye, Julie; Opal, Steven; Tracey, Kevin; van der Poll, Tom; Pelfrene, Eric | Sepsis: a roadmap for future research | Irrelevant results |
| 1299 | 2013 | Stamou, Marianna; Streifel, Karin M.; Goines, Paula E.; Lein, Pamela J. | Neuronal connectivity as a convergent target of gene × environment interactions that confer risk for Autism Spectrum Disorders | Irrelevant results |
| 1300 | 2013 | Murray, Joseph; Cerqueira, Daniel Ricardo de Castro; Kahn, Tulio | Crime and violence in Brazil: Systematic review of time trends, prevalence rates and risk factors | Irrelevant results |
| 1301 | 2001 | Mohler, Beat | Cross-cultural Issues in Research On Child Mental Health | Irrelevant results |
| 1302 | 2018 | Landrigan, Philip J; Fuller, Richard; Acosta, Nereus J R; Adeyi, Olusoji; Arnold, Robert; Basu, Niladri (Nil); and al | The Lancet Commission on pollution and health | Irrelevant results |
| 1303 | 2016 | Finello, Karen Moran; Terteryan, Araksi; Riewerts, Robert J. | Home Visiting Programs: What the Primary Care Clinician Should Know | Irrelevant results |
| 1304 | 2012 | Ducci, Francesca; Goldman, David | The Genetic Basis of Addictive Disorders | Irrelevant results |
| 1305 | 2002 | Elrick, Harold; Samaras, Thomas T; Demas, Antonia | Missing links in the obesity epidemic | Irrelevant results |
| 1306 | 2005 | Volkow, Nora D.; Li, Ting-Kai | Drugs and alcohol: Treating and preventing abuse, addiction and their medical consequences | Irrelevant results |
| 1307 | 2004 | Zametkin, ALAN J.; Zoon, CHRISTINE K.; Klein, HANNAH W.; Munson, SUZANNE | Psychiatric Aspects of Child and Adolescent Obesity: A Review of the Past 10 Years | Irrelevant results |
| 1308 | 2002 | Hayat, M. A. | 1 Prostate carcinoma: An introduction | Irrelevant results |
| 1309 | 2020 |  | NCSBN’s Environmental Scan A Portrait of Nursing and Healthcare in 2020 and Beyond | Irrelevant results |
| 1310 | 2011 | Rizo, Cynthia F.; Macy, Rebecca J. | Help seeking and barriers of Hispanic partner violence survivors: A systematic review of the literature | Irrelevant results |
| 1311 | 2012 | Dahan-Oliel, Noémi; Mazer, Barbara; Majnemer, Annette | Preterm birth and leisure participation: A synthesis of the literature | Irrelevant results |
| 1312 | 2015 | Admani, Shehla; Jinna, Sphoorthi; Friedlander, Sheila Fallon; Sloan, Brett | Cutaneous infectious diseases: Kids are not just little people | Irrelevant results |
| 1313 | 2009 | Weismiller, David G. | Menopause | Irrelevant results |
| 1314 | 2012 | Vearrier, David; Greenberg, Michael I.; Miller, Susan Ney; Okaneku, Jolene T.; Haggerty, David A. | Methamphetamine: History, Pathophysiology, Adverse Health Effects, Current Trends, and Hazards Associated with the Clandestine Manufacture of Methamphetamine | Irrelevant results |
| 1315 | 2003 | Jacobs, Peter; Wood, Lucille | Hematology of malnutrition, part one | Irrelevant results |
| 1316 | 2020 | Atun, Rifat; Bhakta, Nickhill; Denburg, Avram; Frazier, A Lindsay; Friedrich, Paola; Gupta, Sumit; and al | Sustainable care for children with cancer: a Lancet Oncology Commission | Irrelevant results |
| 1317 | 2014 |  | The Egyptian Hypertension Society: EGYPTIAN HYPERTENSION GUIDELINES | Irrelevant results |
| 1318 | 2014 | Shochat, Tamar; Cohen-Zion, Mairav; Tzischinsky, Orna | Functional consequences of inadequate sleep in adolescents: A systematic review | Irrelevant results |
| 1319 | 2019 | Cooke, Graham S; Andrieux-Meyer, Isabelle; Applegate, Tanya L; Atun, Rifat; Burry, Jessica R; Cheinquer, Hugo; Dusheiko, Geoff; and al | Accelerating the elimination of viral hepatitis: a Lancet Gastroenterology & Hepatology Commission | Irrelevant results |
| 1320 | 2015 | Villain, Patricia; Gonzalez, Paula; Almonte, Maribel; Franceschi, Silvia; Dillner, Joakim; Anttila, Ahti; Park, Jin Young; De Vuyst, Hugo; Herrero, Rolando | European Code against Cancer 4th Edition: Infections and Cancer | Irrelevant results |
| 1321 | 2017 | Cullen, Alexis E.; Tappin, Ben M.; Zunszain, Patricia A.; Dickson, Hannah; Roberts, Ruth E.; Nikkheslat, Naghmeh; Khondoker, Mizan; Pariante, Carmine M.; Fisher, Helen L.; Laurens, Kristin R. | The relationship between salivary C-reactive protein and cognitive function in children aged 11–14years: Does psychopathology have a moderating effect? | Irrelevant results |
| 1322 | 2003 | Silverio, Perrotta; Bruno, Nobili; Francesca, Rossi; Daniela Di, Pinto; Valeria, Cucciolla; Adriana, Borriello; Adriana, Oliva; Fulvio Della, Ragione | Vitamin A and Infancy: Biochemical, Functional, and Clinical Aspects | Irrelevant results |
| 1323 | 2017 | Harris, Stewart B.; Tompkins, Jordan W.; TeHiwi, Braden | Call to action: A new path for improving diabetes care for Indigenous peoples, a global review | Irrelevant results |
| 1324 | 2014 | Ottersen, Ole Petter; Dasgupta, Jashodhara; Blouin, Chantal; Buss, Paulo; Chongsuvivatwong, Virasakdi; Frenk, Julio; and al | The political origins of health inequity: prospects for change | Irrelevant results |
| 1325 | 2001 | Schifrin, Emily | An overview of women’s health issues in the United States and United Kingdom | Irrelevant results |
| 1326 | 2005 | Hollier, Lisa M.; Workowski, Kimberly | Treatment of Sexually Transmitted Infections in Pregnancy | Irrelevant results |
| 1327 | 2009 | Yurcisin, Basil M.; Gaddor, Moataz M.; DeMaria, Eric J. | Obesity and Bariatric Surgery | Irrelevant results |
| 1328 | 2003 | Tan, Filemon K | Systemic sclerosis: the susceptible host (genetics and environment) | Irrelevant results |
| 1329 | 2004 | Director, Tara D.; Linden, Judith A. | Domestic violence: an approach to identification and intervention | Irrelevant results |
| 1330 | 2008 | Chipps, Bradley E. | Asthma in Infants and Children | Irrelevant results |
| 1331 | 2017 | Ross, Misha; Mason, Georgia J. | The effects of preferred natural stimuli on humans’ affective states, physiological stress and mental health, and the potential implications for well-being in captive animals | Irrelevant results |
| 1332 | 2013 | Neifert, Marianne; Bunik, Maya | Overcoming Clinical Barriers to Exclusive Breastfeeding | Irrelevant results |
| 1333 | 2013 | Milbrath, Constance | Socio-cultural selection and the sculpting of the human genome: Cultures’ directional forces on evolution and development | Irrelevant results |
| 1334 | 2000 | Budd, Karen S.; Heilman, Nancy E.; Kane, Denise | Psychosocial correlates of child abuse potential in multiply disadvantaged adolescent mothers | Irrelevant results |
| 1335 | 2016 | Pascale, Alisa; Beal, Margaret W.; Fitzgerald, Thérèse | Rethinking the Well Woman Visit: A Scoping Review to Identify Eight Priority Areas for Well Woman Care in the Era of the Affordable Care Act | Irrelevant results |
| 1336 | 2017 | Levin, Adeera; Tonelli, Marcello; Bonventre, Joseph; Coresh, Josef; Donner, Jo-Ann; Fogo, Agnes B; and al | Global kidney health 2017 and beyond: a roadmap for closing gaps in care, research, and policy | Irrelevant results |
| 1337 | 2009 | Popovich, Debbie; McAlhany, Allison; Adewumi, Abimbola O.; Barnes, Marilyn McKim | Scurvy: Forgotten But Definitely Not Gone | Irrelevant results |
| 1338 | 2019 | Barry, Y.; Deneux-Tharaux, C.; Saucedo, M.; Goulet, V.; Guseva-Canu, I.; Regnault, N.; Chantry, A. A. | Maternal admissions to intensive care units in France: Trends in rates, causes and severity from 2010 to 2014 | Irrelevant results |
| 1339 | 2012 | Madukaku, Chukwuocha Uchechukwu; Nosike, Dozie Ikechukwu; Nneoma, Chukwuocha Adanna | Malaria and its burden among pregnant women in parts of the Niger Delta area of Nigeria | Irrelevant results |
| 1340 | 2021 | Lane, Charlotte; Adair, Linda; Bobrow, Emily; Ndayisaba, Gilles F.; Asiimwe, Anita; Mugwaneza, Placidie | Longitudinal interrelationship between HIV viral suppression, maternal weight change, breastfeeding, and length in HIV-exposed and uninfected infants participating in the Kabeho study in Kigali, Rwanda | Irrelevant results |
| 1341 | 2005 | Li, Ling; Fowler, David; Liu, Liang; Ripple, Mary G.; Lambros, Zoe; Smialek, John E. | Investigation of sudden infant deaths in the State of Maryland (1990–2000) | Irrelevant results |
| 1342 | 2018 | Gajaria, Amy; Ravindran, Arun V. | Interventions for perinatal depression in low and middle-income countries: A systematic review | Irrelevant results |
| 1343 | 2006 | Albonico, Marco; Montresor, Antonio; Crompton, D. W. T.; Savioli, Lorenzo | Intervention for the Control of Soil-Transmitted Helminthiasis in the Community | Irrelevant results |
| 1344 | 2015 | Kayar, Nezahat Arzu; Alptekin, Nilgün Özlem; Erdal, Mehmet Emin | Interleukin-1 receptor antagonist gene polymorphism, adverse pregnancy outcome and periodontitis in Turkish women | Irrelevant results |
| 1345 | 2011 | Bhalotra, Sonia; Rawlings, Samantha B. | Intergenerational persistence in health in developing countries: The penalty of gender inequality? | Irrelevant results |
| 1346 | 2017 | Godfrey, Keith M; Reynolds, Rebecca M; Prescott, Susan L; Nyirenda, Moffat; Jaddoe, Vincent W V; Eriksson, Johan G; Broekman, Birit F P | Influence of maternal obesity on the long-term health of offspring | Irrelevant results |
| 1347 | 2020 | le Roux, Stanzi M; Abrams, Elaine J; Donald, Kirsten A; Brittain, Kirsty; Phillips, Tamsin K; Zerbe, Allison; le Roux, David M; Kroon, Max; Myer, Landon | Infectious morbidity of breastfed, HIV-exposed uninfected infants under conditions of universal antiretroviral therapy in South Africa: a prospective cohort study | Irrelevant results |
| 1348 | 2020 | Moreno Oliveras, Luis; Llácer Ortega, José Luis; Leidinger, Andreas; Haji, Mohamed Ali; Chisbert Genovés, María Pilar; Piquer Belloch, José | Infant hydrocephalus in sub-Saharan Africa: Impact of perioperative care in the Zanzibar archipelago | Irrelevant results |
| 1349 | 2021 | Camargos, Paulo; Watts, Kimberly Danieli | Inequalities and Inequities in Pediatric Respiratory Diseases | Irrelevant results |
| 1350 | 2020 | Raju, Sarath; Siddharthan, Trishul; McCormack, Meredith C. | Indoor Air Pollution and Respiratory Health | Irrelevant results |
| 1351 | 2020 | Sadat, Roa; Hall, Patricia L.; Wittenauer, Angela L.; Vengoechea, Elizabeth D.; Park, Kevin; Hagar, Arthur F.; Singh, Rani; Moore, Reneé H.; Gambello, Michael J. | Increased parental anxiety and a benign clinical course: Infants identified with short-chain acyl-CoA dehydrogenase deficiency and isobutyryl-CoA dehydrogenase deficiency through newborn screening in Georgia | Irrelevant results |
| 1352 | 2015 | le Roux, David M; Myer, Landon; Nicol, Mark P; Zar, Heather J | Incidence and severity of childhood pneumonia in the first year of life in a South African birth cohort: the Drakenstein Child Health Study | Irrelevant results |
| 1353 | 2018 | Bonkoungou, Isidore Juste O.; Aliabadi, Negar; Leshem, Eyal; Kam, Madibèlè; Nezien, Désiré; Drabo, Maxime K.; and al | Impact and effectiveness of pentavalent rotavirus vaccine in children <5 years of age in Burkina Faso | Irrelevant results |
| 1354 | 2021 | Artz, Lillian; Swanepoel, Magdaleen; Nagdee, Mohammed; Combrinck, Helene; Kaliski, Sean; Stein, Dan J.; Butterworth, J. | ICD-11 Paraphilic Disorders: A South African Analysis of Its Utility in the Medico-Legal Context | Irrelevant results |
| 1355 | 2019 | Wilkerson, R. Gentry; Ogunbodede, Adeolu C. | Hypertensive Disorders of Pregnancy | Irrelevant results |
| 1356 | 2013 | Ota, M. O. C.; Idoko, O. T.; Ogundare, E. O.; Afolabi, M. O. | Human immune responses to vaccines in the first year of life: Biological, socio-economic and ethical issues – A viewpoint | Irrelevant results |
| 1357 | 2021 | Fagbamigbe, A. F. | How soon does modern contraceptive use starts after sexual debut in Africa? Survival analysis of timing and associated factors among never-in-union women | Irrelevant results |
| 1358 | 2021 | O'Neil, Adrienne; Russell, Josephine D.; Murphy, Barbara | How Does Mental Health Impact Women's Heart Health? | Irrelevant results |
| 1359 | 2015 | Vanker, Aneesa; Barnett, Whitney; Nduru, Polite M.; Gie, Robert P.; Sly, Peter D.; Zar, Heather J. | Home environment and indoor air pollution exposure in an African birth cohort study | Irrelevant results |
| 1360 | 2014 | Kaminsky, Rina Girard; Ault, Steven K.; Castillo, Phillip; Serrano, Kenton; Troya, Guillermo | High prevalence of soil-transmitted helminths in Southern Belize-highlighting opportunity for control interventions | Irrelevant results |
| 1361 | 2018 | Grasty, Madison A.; Ittenbach, Richard F.; Knightly, Carol; Solot, Cynthia B.; Gerdes, Marsha; Bernbaum, Judy C.; and al | Hearing Loss after Cardiac Surgery in Infancy: An Unintended Consequence of Life-Saving Care | Irrelevant results |
| 1362 | 2021 | Spearman, C Wendy; Abdo, Abdelmounem; Ambali, Aggrey; Awuku, Yaw A; Kassianides, Chris; Lesi, Olufunmilayo A; and al | Health-care provision and policy for non-alcoholic fatty liver disease in sub-Saharan Africa | Irrelevant results |
| 1363 | 2010 | Boulet, Sheree L.; Yanni, Emad A.; Creary, Melissa S.; Olney, Richard S. | Health Status and Healthcare Use in a National Sample of Children with Sickle Cell Disease | Irrelevant results |
| 1364 | 2012 | Mayosi, Bongani M; Lawn, Joy E; van Niekerk, Ashley; Bradshaw, Debbie; Abdool Karim, Salim S; Coovadia, Hoosen M | Health in South Africa: changes and challenges since 2009 | Irrelevant results |
| 1365 | 2021 | Dey, Dzifa; Sciascia, Savino; Pons-Estel, Guillermo J.; Ding, Huihua; Shen, Nan | Health Disparities in Rheumatic Diseases: Understanding Global Challenges in Africa, Europe, Latin America, and Asia and Proposing Strategies for Improvement | Irrelevant results |
| 1366 | 2021 | Smitherman, Lynn C.; Golden, William Christopher; Walton, Jennifer R. | Health Disparities and Their Effects on Children and Their Caregivers During the Coronavirus Disease 2019 Pandemic | Irrelevant results |
| 1367 | 2018 | Dolan, Gerry; Benson, Gary; Duffy, Anne; Hermans, Cedric; Jiménez-Yuste, Victor; Lambert, Thierry; Ljung, Rolf; Morfini, Massimo; Zupančić Šalek, Silva | Haemophilia B: Where are we now and what does the future hold? | Irrelevant results |
| 1368 | 2007 | Mannino, David M; Buist, A Sonia | Global burden of COPD: risk factors, prevalence, and future trends | Irrelevant results |
| 1369 | 2018 | Seng, Julia S.; Li, Yang; Yang, James J.; King, Anthony P.; Kane Low, Lisa M.; Sperlich, Mickey; Rowe, Heather; Lee, Hyunhwa; Muzik, Maria; Ford, Julian D.; Liberzon, Israel | Gestational and Postnatal Cortisol Profiles of Women With Posttraumatic Stress Disorder and the Dissociative Subtype | Irrelevant results |
| 1370 | 2010 | Winkler, Cheryl A.; Nelson, George; Oleksyk, Taras K.; Nava, M. Berenice; Kopp, Jeffrey B. | Genetics of Focal Segmental Glomerulosclerosis and Human Immunodeficiency Virus–Associated Collapsing Glomerulopathy: The Role of MYH9 Genetic Variation | Irrelevant results |
| 1371 | 2007 | Cho, June; Holditch-Davis, Diane; Belyea, Michael | Gender and Racial Differences in the Looking and Talking Behaviors of Mothers and Their 3-Year-Old Prematurely Born Children | Irrelevant results |
| 1372 | 2004 | Madhavan, Sangeetha | Fosterage patterns in the age of AIDS: continuity and change | Irrelevant results |
| 1373 | 2016 | Adekanmbi, Victor T.; Kandala, Ngianga-Bakwin; Stranges, Saverio; Uthman, Olalekan A. | Factors That Predict Differences in Childhood Mortality in Nigerian Communities: A Prognostic Model | Irrelevant results |
| 1374 | 2021 | Abukari, Alhassan Sibdow; Awuni, Nathaniel; Yakubu, Ibrahim; Mohammed, Shamsudeen; Yakubu, Adam; Yakubu, Sumani | Factors associated with low fifth minute Apgar score in term and preterm singleton live births in a Ghanaian hospital | Irrelevant results |
| 1375 | 2020 | Rai, Rashmi; Rai, Ambarish Kumar | Exploring the sexual coercion and mental health among young female psychiatric patients in India | Irrelevant results |
| 1376 | 2018 | Shah, Mahek; Ram, Pradhum; Lo, Kevin Bryan; Patnaik, Soumya; Patel, Brijesh; Tripathi, Byomesh; Patil, Shantanu; Lu, Marvin; Jorde, Ulrich P.; Figueredo, Vincent M. | Etiologies, Predictors, and Economic Impact of 30-Day Readmissions Among Patients With Peripartum Cardiomyopathy | Irrelevant results |
| 1377 | 2002 | Rushton, JERRY L.; Forcier, MICHELLE; Schectman, ROBIN M. | Epidemiology of Depressive Symptoms in the National Longitudinal Study of Adolescent Health | Irrelevant results |
| 1378 | 2016 | Younger, David S. | Epidemiology of Childhood and Adult Mental Illness | Irrelevant results |
| 1379 | 2011 | Ndyomugyenyi, Richard; Clarke, Siân E.; Hutchison, Coll L.; Hansen, Kristian Schultz; Magnussen, Pascal | Efficacy of malaria prevention during pregnancy in an area of low and unstable transmission: an individually-randomised placebo-controlled trial using intermittent preventive treatment and insecticide-treated nets in the Kabale Highlands, southwestern Uganda | Irrelevant results |
| 1380 | 2019 | Schuck-Paim, Cynthia; Taylor, Robert J; Alonso, Wladimir J; Weinberger, Daniel M; Simonsen, Lone | Effect of pneumococcal conjugate vaccine introduction on childhood pneumonia mortality in Brazil: a retrospective observational study | Irrelevant results |
| 1381 | 2005 | Semba, Richard D.; Ndugwa, Christopher; Perry, Robert T.; Clark, Tamara D.; Jackson, J. Brooks; Melikian, George; Tielsch, James; Mmiro, Francis | Effect of periodic vitamin A supplementation on mortality and morbidity of human immunodeficiency virus–infected children in Uganda: A controlled clinical trial | Irrelevant results |
| 1382 | 2021 | Singh, Dharmendra P.; Biradar, Rajeshwari A.; Halli, Shiva S.; Dwivedi, Laxmi Kant | Effect of maternal nutritional status on children nutritional status in India | Irrelevant results |
| 1383 | 2021 | Kim, Eunsoo Timothy; Opiyo, Tobias; Acayo, Pauline S.; Lillie, Margaret; Gallis, John; Zhou, Yunji; Ochieng, Michael; Okuro, Samwel; Hembling, John; McEwan, Elena; Baumgartner, Joy Noel | Effect of a lay counselor delivered integrated maternal mental health and early childhood development group-based intervention in Siaya County, Kenya: A quasi-experimental longitudinal study | Irrelevant results |
| 1384 | 2020 | Zar, Heather J; Nduru, Polite; Stadler, Jacob A M; Gray, Diane; Barnett, Whitney; Lesosky, Maia; Myer, Landon; Nicol, Mark P | Early-life respiratory syncytial virus lower respiratory tract infection in a South African birth cohort: epidemiology and effect on lung health | Irrelevant results |
| 1385 | 2020 | Li, Yanqi; Shen, René Liang; Ayede, Adejumoke I.; Berrington, Janet; Bloomfield, Frank H.; Busari, Olubunmi O.; Cormack, Barbara E.; and al | Early Use of Antibiotics Is Associated with a Lower Incidence of Necrotizing Enterocolitis in Preterm, Very Low Birth Weight Infants: The NEOMUNE-NeoNutriNet Cohort Study | Irrelevant results |
| 1386 | 2015 | Coffey, Diane | Early life mortality and height in Indian states | Irrelevant results |
| 1387 | 2004 | Vial, Thierry; Descotes, Jacques; Ludwig, Corinna; Behrend, Matthias | Drugs acting on the immune system | Irrelevant results |
| 1388 | 2018 | Esterhuizen, Alina I.; Mefford, Heather C.; Ramesar, Rajkumar S.; Wang, Shuyu; Carvill, Gemma L.; Wilmshurst, Jo M. | Dravet syndrome in South African infants: Tools for an early diagnosis | Irrelevant results |
| 1389 | 2021 | Vink, Martijn; Upadhaya, Nawaraj; Amin, Hazrat Amin; Liwal, Mohammad Gul; Siddiqui, Abdul Majeed; Naseem, Mohammad; Syawash, Aziza; Schim van der Loeff, Maarten | Does support to Private Health Practitioners increase childhood vaccination coverage? Findings from a comparative study in Afghanistan | Irrelevant results |
| 1390 | 2015 | Dagher, Rada K.; Green, Kerry M. | Does depression and substance abuse co-morbidity affect socioeconomic status? Evidence from a prospective study of urban African Americans | Irrelevant results |
| 1391 | 2021 | Huryk, Kathryn M.; Drury, Catherine R.; Loeb, Katharine L. | Diseases of affluence? A systematic review of the literature on socioeconomic diversity in eating disorders | Irrelevant results |
| 1392 | 2016 | Goldstein, Nira A.; Gorynski, Michael; Yip, Candice; Harounian, Jonathan; Huberman, Harris; Weedon, Jeremy | Developmental delay in young children with sleep-disordered breathing before and after tonsil and adenoid surgery | Irrelevant results |
| 1393 | 2015 | Pathirana, Jayani; Nkambule, Jerome; Black, Steven | Determinants of maternal immunization in developing countries | Irrelevant results |
| 1394 | 2021 | Ngwenya, Solwayo; Jones, Brian; Mwembe, Desmond; Nare, Hausitoe; Heazell, Alexander E. P. | Determinants of eclampsia in women with severe preeclampsia at Mpilo Central Hospital, Bulawayo, Zimbabwe | Irrelevant results |
| 1395 | 2012 | Bacharier, Leonard B.; Cohen, Rebecca; Schweiger, Toni; Yin-DeClue, Huiquing; Christie, Chandrika; Zheng, Jie; Schechtman, Kenneth B.; Strunk, Robert C.; Castro, Mario | Determinants of asthma after severe respiratory syncytial virus bronchiolitis | Irrelevant results |
| 1396 | 2021 | Windi, Restu; Efendi, Ferry; Qona'ah, Arina; Adnani, Qorinah Estiningtyas Sakilah; Ramadhan, Kadar; Almutairi, Wedad M. | Determinants of Acute Respiratory Infection Among Children Under-Five Years in Indonesia | Irrelevant results |
| 1397 | 2017 | Rosman, Lindsey; Salmoirago-Blotcher, Elena; Cahill, John; Wuensch, Karl L.; Sears, Samuel F. | Depression and health behaviors in women with Peripartum Cardiomyopathy | Irrelevant results |
| 1398 | 2020 | Maharaj, Shivesh; Mungul, Sheetal; Ahmed, Sumaya | Deep Neck Space Infections: Changing Trends in Pediatric Versus Adult Patients | Irrelevant results |
| 1399 | 2014 | Avery, Melissa D. | Current Resources for Evidence‐Based Practice, March/April 2014 | Irrelevant results |
| 1400 | 2021 | Gosset, Andréa; Diallo, Mamadou Yaya; Betsem, Edouard; Schaeffer, Laura; Meda, Nicolas; Vray, Muriel; Sombie, Roger; Shimakawa, Yusuke; Boyer, Sylvie | Cost-effectiveness of adding a birth dose of hepatitis B vaccine in the Dafra district of the Hauts-Bassins Region in Burkina Faso (NéoVac Study) | Irrelevant results |
| 1401 | 2016 | Kuo, Kelly; Caughey, Aaron B. | Contemporary outcomes of sickle cell disease in pregnancy | Irrelevant results |
| 1402 | 2003 | Abbrescia, Kelly; Sheridan, Barry | Complications of second and third trimester pregnancies | Irrelevant results |
| 1403 | 2008 | Rosato, Mikey; Laverack, Glenn; Grabman, Lisa Howard; Tripathy, Prasanta; Nair, Nirmala; Mwansambo, Charles; Azad, Kishwar; Morrison, Joanna; Bhutta, Zulfiqar; Perry, Henry; Rifkin, Susan; Costello, Anthony | Community participation: lessons for maternal, newborn, and child health | Irrelevant results |
| 1404 | 2017 | Simon, Erica; Long, Brit; Koyfman, Alex | Clinical Mimics: An Emergency Medicine–Focused Review of Influenza Mimics | Irrelevant results |
| 1405 | 2020 | Karaye, Kamilu M.; Sa’idu, Hadiza; Balarabe, Sulaiman A.; Ishaq, Naser A.; Adamu, Umar G.; Mohammed, Idris Y.; and al | Clinical Features and Outcomes of Peripartum Cardiomyopathy in Nigeria | Irrelevant results |
| 1406 | 2020 | Thiede, Brian C.; Strube, Johann | Climate variability and child nutrition: Findings from sub-Saharan Africa | Irrelevant results |
| 1407 | 2021 | Pacheco, Susan E.; Guidos, Guillermo; Annesi-Maesano, Isabella; Pawankar, Ruby; Amato, Gennaro D'; Latour-Staffeld, Patricia; Urrutia-Pereira, Marylin; Kesic, Matthew J.; Hernandez, Michelle L. | Climate Change and Global Issues in Allergy and Immunology | Irrelevant results |
| 1408 | 2020 | Frigati, Lisa J; Ameyan, Wole; Cotton, Mark F; Gregson, Celia L; Hoare, Jacqueline; Jao, Jennifer; Majonga, Edith D; Myer, Landon; Penazzato, Martina; Rukuni, Ruramayi; Rowland-Jones, Sarah; Zar, Heather J; Ferrand, Rashida A | Chronic comorbidities in children and adolescents with perinatally acquired HIV infection in sub-Saharan Africa in the era of antiretroviral therapy | Irrelevant results |
| 1409 | 2018 | Barr, Peter B.; Silberg, Judy; Dick, Danielle M.; Maes, Hermine H. | Childhood socioeconomic status and longitudinal patterns of alcohol problems: Variation across etiological pathways in genetic risk | Irrelevant results |
| 1410 | 2021 | Pool, Lindsay R.; Aguayo, Liliana; Brzezinski, Michal; Perak, Amanda M.; Davis, Matthew M.; Greenland, Philip; Hou, Lifang; Marino, Bradley S.; Van Horn, Linda; Wakschlag, Lauren; Labarthe, Darwin; Lloyd-Jones, Donald; Allen, Norrina B. | Childhood Risk Factors and Adulthood Cardiovascular Disease: A Systematic Review | Irrelevant results |
| 1411 | 2013 | Barry, Meagan A.; Weatherhead, Jill E.; Hotez, Peter J.; Woc-Colburn, Laila | Childhood Parasitic Infections Endemic to the United States | Irrelevant results |
| 1412 | 2017 | Nguyen, T. K. P.; Tran, T. H.; Roberts, C. L.; Graham, S. M.; Marais, B. J. | Child pneumonia – focus on the Western Pacific Region | Irrelevant results |
| 1413 | 2016 | Oberg, Charles; Colianni, Sonja; King-Schultz, Leslie | Child Health Disparities in the 21st Century | Irrelevant results |
| 1414 | 2020 | Mostafa, Gamal A. E.; Al-Dosseri, Abdullah S.; Al-Badr, Abdullah A. | Chapter Seven - Piroxicam | Irrelevant results |
| 1415 | 2017 | Urbano, Richard C.; Epstein, Richard A.; Cull, Michael J.; Vehorn, Alison; Warren, Zachary | Chapter One - Autism and Child Maltreatment: A Population-Based, Record Linkage Methodology for Studying Children Referred to Child Protective Services | Irrelevant results |
| 1416 | 2007 | Strauss, John; Thomas, Duncan | Chapter 54 Health over the Life Course | Irrelevant results |
| 1417 | 2013 | Patil, Crystal L.; Abrams, Elizabeth T.; Klima, Carrie; Kaponda, Chrissie P. N.; Leshabari, Sebalda C.; Vonderheid, Susan C.; Kamanga, Martha; Norr, Kathleen F. | CenteringPregnancy-Africa: A pilot of group antenatal care to address Millennium Development Goals | Irrelevant results |
| 1418 | 2020 | Finaret, Amelia B.; Masters, William A. | Can shorter mothers have taller children? Nutritional mobility, health equity and the intergenerational transmission of relative height | Irrelevant results |
| 1419 | 2011 | Kavita, Rajesh; Girish, Nagarajarao; Gururaj, Gopalkrishna | Burden, Characteristics, and Outcome of Injury among Females: Observations from Bengaluru, India | Irrelevant results |
| 1420 | 2021 | Patel, Kamalesh Kumar; Vijay, Jyoti; Mangal, Abha; Mangal, Daya Krishan; Gupta, Shiv Dutt | Burden of anaemia among children aged 6–59 months and its associated risk factors in India – Are there gender differences? | Irrelevant results |
| 1421 | 2020 | Herrera Cuenca, Marianella; Proaño, Gabriela V.; Blankenship, Jeanne; Cano-Gutierrez, Carlos; Chew, Samuel T. H.; Fracassi, Patrizia; Keller, Heather; Venkatesh Mannar, M. G.; Mastrilli, Valeria; Milewska, Magdalena; Steiber, Alison | Building Global Nutrition Policies in Health Care: Insights for Tackling Malnutrition from the Academy of Nutrition and Dietetics 2019 Global Nutrition Research and Policy Forum | Irrelevant results |
| 1422 | 2019 | Ryan, Rita M.; Feng, Rui; Bazacliu, Catalina; Ferkol, Thomas W.; Ren, Clement L.; Mariani, Thomas J.; and al | Black Race Is Associated with a Lower Risk of Bronchopulmonary Dysplasia | Irrelevant results |
| 1423 | 2020 | Prather, Susan L.; Foronda, Cynthia L.; Kelley, Courtney N.; Nadeau, Catherine; Prather, Khaila | Barriers and Facilitators of Asthma Management as Experienced by African American Caregivers of Children with Asthma: An Integrative Review | Irrelevant results |
| 1424 | 2020 | MacGinty, RP; Kariuki, SM; Barnett, W; Wedderburn, CJ; Hardy, A; Hoffman, N; Newton, CR; Zar, HJ; Donald, KA; Stein, DJ | Associations of antenatal maternal psychological distress with infant birth and development outcomes: Results from a South African birth cohort | Irrelevant results |
| 1425 | 2021 | Sevenoaks, Tatum; Wedderburn, Catherine J.; Donald, Kirsten A.; Barnett, Whitney; Zar, Heather J.; Stein, Dan J.; Naudé, Petrus J. W. | Association of maternal and infant inflammation with neurodevelopment in HIV-exposed uninfected children in a South African birth cohort | Irrelevant results |
| 1426 | 2015 | Wing, Robyn; Gjelsvik, Annie; Nocera, Mariann; McQuaid, Elizabeth L. | Association between adverse childhood experiences in the home and pediatric asthma | Irrelevant results |
| 1427 | 2020 | Shalaby, Aly; Obeida, Alaa; Khairy, Dalia; Bahaaeldin, Khaled | Assessment of gastroschisis risk factors in Egypt | Irrelevant results |
| 1428 | 2001 | Crompton, D. W. T. | Ascaris and ascariasis | Irrelevant results |
| 1429 | 2012 | Yancey, Joel B.; Nussbaum, Marcy L.; Elliot, Mollie C.; Kullstam, Susan M.; Franco, Albert | Antenatal sexually transmitted infection screening in private and indigent clinics in a community hospital system | Irrelevant results |
| 1430 | 2010 | Peacock, Ann S.; Bogossian, Fiona | Antenatal screening and predicting hypertension in pregnancy for midwives | Irrelevant results |
| 1431 | 2021 | Jacques, Nadège; Mesenburg, Marilia Arndt; Murray, Joseph; Bertoldi, Andréa Dâmaso; Domingues, Marlos Rodrigues; Stein, Alan; Silveira, Mariangela Freitas | Antenatal and Postnatal Maternal Depressive Symptoms and Trajectories and Child Hospitalization up to 24 Months of Life: Findings From the 2015 Pelotas (Brazil) Birth Cohort Study | Irrelevant results |
| 1432 | 2000 | Misra, Dawn P; Grason, Holly; Weisman, Carol | An intersection of women’s and perinatal health: the role of chronic conditions | Irrelevant results |
| 1433 | 2019 | WHO | Mortalité maternelle | Irrelevant results |
| 1434 | 2016 | Tanimia, Hilda; Jayaratnam, Skandarupan; Mola, Glen L.; Amoa, Apeawusu B.; de Costa, Caroline | Near-misses at the Port Moresby General Hospital: a descriptive study | Irrelevant results |
| 1435 | 2021 | Geze Tenaw, Shegaw; Girma Fage, Sagni; Assefa, Nega; Kenay Tura, Abera | Determinants of maternal near-miss in private hospitals in eastern Ethiopia: A nested case-control study | Included |
| 1436 | 2021 | Tolesa, Dereje | Prevalence and Associated Factors with Maternal Near-Miss among Pregnant Women at Hawassa University Comprehensive Specialized Hospital, Sidama Region, Ethiopia | Included |
| 1437 | 2014 | Oud, Lavi | Pregnancy-Associated Severe Sepsis: Contemporary State and Future Challenges | Irrelevant results |
| 1438 | 2013 | Chan, Grace J.; Lee, Anne CC; Baqui, Abdullah H.; Tan, Jingwen; Black, Robert E. | Risk of Early-Onset Neonatal Infection with Maternal Infection or Colonization: A Global Systematic Review and Meta-Analysis | Irrelevant results |
| 1439 | 2016 | Chou, Doris; Tunçalp, Özge; Firoz, Tabassum; Barreix, Maria; Filippi, Veronique; von Dadelszen, Peter; van den Broek, Nynke; Cecatti, Jose Guilherme; Say, Lale; on behalf of the Maternal Morbidity Working Group | Constructing maternal morbidity – towards a standard tool to measure and monitor maternal health beyond mortality | Irrelevant results |
| 1440 | 2017 | Bonet, Mercedes; Nogueira Pileggi, Vicky; Rijken, Marcus J.; Coomarasamy, Arri; Lissauer, David; Souza, João Paulo; Gülmezoglu, Ahmet Metin | Towards a consensus definition of maternal sepsis: results of a systematic review and expert consultation | Irrelevant results |
| 1441 | 2016 | Kalisa, Richard; Rulisa, Stephen; van den Akker, Thomas; van Roosmalen, Jos | Maternal Near Miss and quality of care in a rural Rwandan hospital | Irrelevant results |
| 1442 | 2020 | Owolabi, Onikepe; Riley, Taylor; Juma, Kenneth; Mutua, Michael; Pleasure, Zoe H.; Amo-Adjei, Joshua; Bangha, Martin | Incidence of maternal near-miss in Kenya in 2018: findings from a nationally representative cross-sectional study in 54 referral hospitals | Irrelevant results |
| 1443 | 2017 | Liyew, Ewnetu Firdawek; Yalew, Alemayehu Worku; Afework, Mesganaw Fantahun; Essén, Birgitta | Incidence and causes of maternal near-miss in selected hospitals of Addis Ababa, Ethiopia | Irrelevant results |
| 1444 | 2017 | Hanson, Claudia; Pembe, Andrea B.; Alwy, Fadhlun; Atuhairwe, Susan; Leshabari, Sebalda; Morris, Jessica; Kaharuza, Frank; Marrone, Gaetano; HMS BAB study team | Evaluating the effect of the Helping Mothers Survive Bleeding after Birth (HMS BAB) training in Tanzania and Uganda: study protocol for a randomised controlled trial | Irrelevant results |
| 1445 | 2019 | Ononuju, Chidiebere Nwakamma; Ogbe, Adejo Emmanuel; Changkat, Lucky Lohnan; Okwaraoha, Blaise Ogedi; Chinaka, Uzoma Emmanuel | Ectopic pregnancy in Dalhatu Araf Specialist Hospital Lafia Nigeria - A 5-year review | Irrelevant results |
| 1446 | 2021 | Tekola, Anteneh Fikrie; Baye, Genet; Amaje, Elias; Tefera, Kebede | Neonatal near misses and associated factors among mother's who give a live neonate at Hawassa City governmental hospitals, 2019: a facility based cross-sectional study design | Irrelevant results |
| 1447 | 2019 | Okunowo, Adeyemi Adebola; Ohazurike, Ephraim Okwudiri; Habeebu-Adeyemi, Fatimah Murtazha | Undiagnosed placenta praevia percreta: A rare case report and review of management | Irrelevant results |
| 1448 | 2013 | Adeoye, Ikeola A.; Onayade, Adedeji A.; Fatusi, Adesegun O. | Incidence, determinants and perinatal outcomes of near miss maternal morbidity in Ile-Ife Nigeria: a prospective case control study | Included |
| 1449 | 2019 | Hirose, Atsumi; Alwy, Fadhlun; Atuhairwe, Susan; Morris, Jessica L.; Pembe, Andrea B.; Kaharuza, Frank; Marrone, Gaetano; Hanson, Claudia | Disentangling the contributions of maternal and fetal factors to estimate stillbirth risks for intrapartum adverse events in Tanzania and Uganda | Irrelevant results |
| 1450 | 2012 | Storeng, Katerini T.; Drabo, Seydou; Ganaba, Rasmané; Sundby, Johanne; Calvert, Clara; Filippi, Véronique | Mortality after near-miss obstetric complications in Burkina Faso: medical, social and health-care factors | Irrelevant results |
| 1451 | 2016 | Rwabizi, Denis; Rulisa, Stephen; Findlater, Aidan; Small, Maria | Maternal near miss and mortality due to postpartum infection: a cross-sectional analysis from Rwanda | Irrelevant results |
| 1452 | 2015 | Assarag, Bouchra; Dujardin, Bruno; Delamou, Alexandre; Meski, Fatima-Zahra; De Brouwere, Vincent | Determinants of maternal near-miss in Morocco: too late, too far, too sloppy? | Included |
| 1453 | 2020 | Awowole, I. O.; Omitinde, O. S.; Ayegbusi, E. O.; Kolawole, O. O.; Ijarotimi, A. O.; Badejoko, O. O. | Severe Maternal Outcomes Associated with Abortion-Related Sepsis at the Obafemi Awolowo University Teaching Hospitals Complex (OAUTHC), Ile-Ife, Nigeria: Experience from the Last Decade of Millennium Development Goal Era | Irrelevant results |
| 1454 | 2017 | Herklots, Tanneke; van Acht, Lieke; Meguid, Tarek; Franx, Arie; Jacod, Benoit | Severe maternal morbidity in Zanzibar's referral hospital: Measuring the impact of in-hospital care | Irrelevant results |
| 1455 | 2020 | Chama, Calvin M.; Etuk, Saturday J.; Oladapo, Olufemi T. | The Pattern and Spectrum of Severe Maternal Morbidities in Nigerian tertiary Hospitals | Irrelevant results |
| 1456 | 2015 | Zafar, Shamsa; Jean-Baptiste, Rachel; Rahman, Atif; Neilson, James P.; van den Broek, Nynke R. | Non-Life Threatening Maternal Morbidity: Cross Sectional Surveys from Malawi and Pakistan | Irrelevant results |
| 1457 | 2018 | Flynn, Patricia M.; Taha, Taha E.; Cababasay, Mae; Fowler, Mary Glenn; Mofenson, Lynne M.; Owor, Maxensia; and al | Prevention of HIV-1 Transmission Through Breastfeeding: Efficacy and Safety of Maternal Antiretroviral Therapy Versus Infant Nevirapine Prophylaxis for Duration of Breastfeeding in HIV-1-Infected Women With High CD4 Cell Count (IMPAACT PROMISE): A Randomized, Open-Label, Clinical Trial | Irrelevant results |
| 1458 | 2018 | Ahmed, Seid Mussa; Nordeng, Hedvig; Sundby, Johanne; Aragaw, Yesuf Ahmed; de Boer, Hugo J. | The use of medicinal plants by pregnant women in Africa: A systematic review | Irrelevant results |
| 1459 | 2006 | Stewart, D. E.; Ashraf, I. J.; Munce, S. E. | Women's mental health: A silent cause of mortality and morbidity | Irrelevant results |
| 1460 | 2021 | Ameyaw, Edward Kwabena; Amoah, Roberta Mensima; Njue, Carolyne; Tran, Nguyen Toan; Dawson, Angela | Women's experiences and satisfaction with maternal referral service in Northern Ghana: A qualitative inquiry | Irrelevant results |
| 1461 | 2005 | Smith, Lisa C.; Ruel, Marie T.; Ndiaye, Aida | Why Is Child Malnutrition Lower in Urban Than in Rural Areas? Evidence from 36 Developing Countries | Irrelevant results |
| 1462 | 2006 | Mangiaterra, Viviana; Mattero, Minna; Dunkelberg, Erika | Why and how to invest in neonatal health | Irrelevant results |
| 1463 | 2007 | Harper, Margaret; Dugan, Elizabeth; Espeland, Mark; Martinez-Borges, Anibal; McQuellon, Cynthia | Why African-American Women Are at Greater Risk for Pregnancy-Related Death | Irrelevant results |
| 1464 | 2020 | Vivanti, Alexandre J.; Monier, Isabelle; Salakos, Eleonora; Elie, Caroline; Tsatsaris, Vassilis; Senat, Marie-Victoire; and al | Vitamin D and pregnancy outcomes: Overall results of the FEPED study | Irrelevant results |
| 1465 | 2000 | Fawzi, Wafaie W.; Mbise, Roger; Spiegelman, Donna; Fataki, Maulidi; Hertzmark, Ellen; Ndossi, Godwin | Vitamin A supplements and diarrheal and respiratory tract infections among children in Dar es Salaam, Tanzania | Irrelevant results |
| 1466 | 2018 | Gurgel, Cristiane Santos Sânzio; Grilo, Evellyn C.; Lira, Larissa Q.; Assunção, Débora G. F.; Oliveira, Priscila G.; Melo, Larisse R. M. de; de Medeiros, Silvia V.; Pessanha, Luanna C.; Dimenstein, Roberto; Lyra, Clélia O. | Vitamin A nutritional status in high- and low-income postpartum women and its effect on colostrum and the requirements of the term newborn | Irrelevant results |
| 1467 | 2009 | Srinivasan, Usha; Misra, Dawn; Marazita, Mary L.; Foxman, Betsy | Vaginal and oral microbes, host genotype and preterm birth | Irrelevant results |
| 1468 | 2007 | Fotso, Jean-Christophe | Urban–rural differentials in child malnutrition: Trends and socioeconomic correlates in sub-Saharan Africa | Irrelevant results |
| 1469 | 2021 | Sebghati, Mercede; Khalil, Asma | Uptake of vaccination in pregnancy | Irrelevant results |
| 1470 | 2012 | Cook, Rebecca J.; Dickens, Bernard M. | Upholding pregnant women's right to life | Irrelevant results |
| 1471 | 2019 | Boe, Brendan; Barbour, Linda A.; Allshouse, Amanda A.; Heyborne, Kent D. | Universal early pregnancy glycosylated hemoglobin A1c as an adjunct to Carpenter-Coustan screening: an observational cohort study | Irrelevant results |
| 1472 | 2011 | Molina, Kristine M.; Kiely, Michele | Understanding Depressive Symptoms among High-Risk, Pregnant, African-American Women | Irrelevant results |
| 1473 | 2010 | Zinn, Andrew R.; Palmer, Biff F. | Unconventional Wisdom About the Obesity Epidemic Symbol | Irrelevant results |
| 1474 | 2015 | Petrone, Patrizio; Marini, Corrado P. | Trauma in pregnant patients | Irrelevant results |
| 1475 | 2019 | Sakamoto, Jeffrey; Michels, Collin; Eisfelder, Bryn; Joshi, Nikita | Trauma in Pregnancy | Irrelevant results |
| 1476 | 2006 | Mullany, Luke C; Darmstadt, Gary L; Khatry, Subarna K; Katz, Joanne; LeClerq, Steven C; Shrestha, Shardaram; Adhikari, Ramesh; Tielsch, James M | Topical applications of chlorhexidine to the umbilical cord for prevention of omphalitis and neonatal mortality in southern Nepal: a community-based, cluster-randomised trial | Irrelevant results |
| 1477 | 2011 | Pham, Thao; Bachelez, Hervé; Berthelot, Jean-Marie; Blacher, Jacques; Bouhnik, Yoram; Claudepierre, Pascal; and al | TNF alpha antagonist therapy and safety monitoring | Irrelevant results |
| 1478 | 2008 | Garly, May-Lill; Trautner, Sisse Lecanda; Marx, Charlotte; Danebod, Kamilla; Nielsen, Jens; Ravn, Henrik; Martins, Cesário Lourenco; Balé, Carlito; Aaby, Peter; Lisse, Ida Maria | Thymus Size at 6 Months of Age and Subsequent Child Mortality | Irrelevant results |
| 1479 | 2007 | Tsui, A. O.; Creanga, A. A.; Ahmed, S. | The role of delayed childbearing in the prevention of obstetric fistulas | Irrelevant results |
| 1480 | 2001 | Shalowitz, Madeleine U.; Berry, Carolyn A.; Quinn, Kelly A.; Wolf, Raoul L. | The Relationship of Life Stressors and Maternal Depression to Pediatric Asthma Morbidity in a Subspecialty Practice | Irrelevant results |
| 1481 | 2015 | Neggers, Yasmin H. | The relationship between preterm birth and underweight in Asian women | Irrelevant results |
| 1482 | 2019 | Katzow, Michelle; Messito, Mary Jo; Mendelsohn, Alan L.; Scott, Marc A.; Gross, Rachel S. | The Protective Effect of Prenatal Social Support on Infant Adiposity in the First 18 Months of Life | Irrelevant results |
| 1483 | 2004 | Lewis Wall, L; Karshima, Jonathan A; Kirschner, Carolyn; Arrowsmith, Steven D | The obstetric vesicovaginal fistula: Characteristics of 899 patients from Jos, Nigeria | Irrelevant results |
| 1484 | 2017 | DiPietro, J. A.; Voegtline, K. M. | The gestational foundation of sex differences in development and vulnerability | Irrelevant results |
| 1485 | 2017 | Maswime, T. S.; Buchmann, E. | Near-miss maternal morbidity from severe haemorrhage at caesarean section: A process and structure audit of system deficiencies in South Africa | Irrelevant results |
| 1486 | 2015 | Naderi, Tayebeh; Foroodnia, Shohreh; Omidi, Samaneh; Samadani, Faezeh; Nakhaee, Nouzar | Incidence and Correlates of Maternal Near Miss in Southeast Iran | Excluded for out of context |
| 1487 | 2012 | Woolf, Anthony D.; Erwin, Jo; March, Lyn | The need to address the burden of musculoskeletal conditions | Irrelevant results |
| 1488 | 2021 | Burton, Matthew J; Ramke, Jacqueline; Marques, Ana Patricia; Bourne, Rupert R A; Congdon, Nathan; Jones, Iain; and al | The Lancet Global Health Commission on Global Eye Health: vision beyond 2020 | Irrelevant results |
| 1489 | 2014 | Kerac, Marko; Postels, Douglas G.; Mallewa, Mac; Alusine Jalloh, Alhaji; Voskuijl, Wieger P.; Groce, Nora; Gladstone, Melissa; Molyneux, Elizabeth | The Interaction of Malnutrition and Neurologic Disability in Africa | Irrelevant results |
| 1490 | 2020 | Baral, Ranju; Li, Xiao; Willem, Lander; Antillon, Marina; Vilajeliu, Alba; Jit, Mark; Beutels, Philippe; Pecenka, Clint | The impact of maternal RSV vaccine to protect infants in Gavi-supported countries: Estimates from two models | Irrelevant results |
| 1491 | 2002 | Horner, Sharon D.; Surratt, Dawn; Smith, Susan B. | The impact of asthma risk factors on home management of childhood asthma | Irrelevant results |
| 1492 | 2014 | Eltoukhi, Heba M.; Modi, Monica N.; Weston, Meredith; Armstrong, Alicia Y.; Stewart, Elizabeth A. | The health disparities of uterine fibroid tumors for African American women: a public health issue | Irrelevant results |
| 1493 | 2017 | van de Wijgert, Janneke H. H. M.; Jespers, Vicky | The global health impact of vaginal dysbiosis | Irrelevant results |
| 1494 | 2017 | Dheda, Keertan; Gumbo, Tawanda; Maartens, Gary; Dooley, Kelly E; McNerney, Ruth; Murray, Megan; and al | The epidemiology, pathogenesis, transmission, diagnosis, and management of multidrug-resistant, extensively drug-resistant, and incurable tuberculosis | Irrelevant results |
| 1495 | 2021 | de Souza, Daniela Carla; Gonçalves Martin, Joelma; Soares Lanziotti, Vanessa; de Oliveira, Cláudio Flauzino; Tonial, Cristian; de Carvalho, Werther Brunow; and al | The epidemiology of sepsis in paediatric intensive care units in Brazil (the Sepsis PREvalence Assessment Database in Pediatric population, SPREAD PED): an observational study | Irrelevant results |
| 1496 | 2020 | Nelson, Karin B. | The epidemiology of FIRS in term and late preterm births | Irrelevant results |
| 1497 | 2019 | Lindsey, Benjamin B; Armitage, Edwin P; Kampmann, Beate; de Silva, Thushan I | The efficacy, effectiveness, and immunogenicity of influenza vaccines in Africa: a systematic review | Irrelevant results |
| 1498 | 2018 | Wood, Beatrice L.; Brown, E. Sherwood; Lehman, Heather K.; Khan, David A.; Lee, Min Jung; Miller, Bruce D. | The effects of caregiver depression on childhood asthma: Pathways and mechanisms | Irrelevant results |
| 1499 | 2014 | Ardington, Cally; Bärnighausen, Till; Case, Anne; Menendez, Alicia | The economic consequences of AIDS mortality in South Africa | Irrelevant results |
| 1500 | 2005 | Kavanaugh, Megan; McMillen, Robert C.; Pascoe, John M.; Hill Southward, Linda; Winickoff, Jonathan P.; Weitzman, Michael | The Co-Occurrence of Maternal Depressive Symptoms and Smoking in a National Survey of Mothers | Irrelevant results |
| 1501 | 2018 | Murphy, Karly A.; Ellison-Barnes, Alejandra; Johnson, Erica N.; Cooper, Lisa A. | The Clinical Examination and Socially At-Risk Populations: The Examination Matters for Health Disparities | Irrelevant results |
| 1502 | 2014 | Mehta, Shobha H.; Kerver, Jean M.; Sokol, Robert J.; Keating, Daniel P.; Paneth, Nigel | The Association between Maternal Obesity and Neurodevelopmental Outcomes of Offspring | Irrelevant results |
| 1503 | 2016 | Amegah, A. Kofi; Rezza, Giovanni; Jaakkola, Jouni J. K. | Temperature-related morbidity and mortality in Sub-Saharan Africa: A systematic review of the empirical evidence | Irrelevant results |
| 1504 | 2019 | Herring, Sharon J.; Albert, Jessica J.; Darden, Niesha; Bailer, Brooke; Cruice, Jane; Hassan, Sarmina; Bennett, Gary G.; Goetzl, Laura; Yu, Daohai; Kilby, Linda M.; Foster, Gary D. | Targeting pregnancy-related weight gain to reduce disparities in obesity: Baseline results from the Healthy Babies trial | Irrelevant results |
| 1505 | 2009 | Odierna, Donna H.; Bero, Lisa A. | Systematic reviews reveal unrepresentative evidence for the development of drug formularies for poor and nonwhite populations | Irrelevant results |
| 1506 | 2014 | Lanzieri, Tatiana M.; Dollard, Sheila C.; Bialek, Stephanie R.; Grosse, Scott D. | Systematic review of the birth prevalence of congenital cytomegalovirus infection in developing countries | Irrelevant results |
| 1507 | 2020 | Restrepo-Posada, Deisy Cristina; Carmona-Fonseca, Jaime; Cardona-Arias, Jaiberth Antonio | Systematic review of microeconomic analysis of pregnancy-associated malaria | Irrelevant results |
| 1508 | 2018 | Lancaster, Kathryn E.; Hetrick, Angela; Jaquet, Antoine; Adedimeji, Adebola; Atwoli, Lukoye; Colby, Donn J.; Mayor, Angel M.; Parcesepe, Angela; Syvertsen, Jennifer | Substance use and universal access to HIV testing and treatment in sub-Saharan Africa: implications and research priorities | Irrelevant results |
| 1509 | 2021 | Verma, Pradyuman; Prasad, Jang Bahadur | Stunting, wasting and underweight as indicators of under-nutrition in under five children from developing Countries: A systematic review | Irrelevant results |
| 1510 | 2007 | Black, Kathleen D. | Stress, Symptoms, Self‐Monitoring Confidence, Well‐Being, and Social Support in the Progression of Preeclampsia/Gestational Hypertension | Irrelevant results |
| 1511 | 2017 | Jain, Joses; Moroz, Leslie | Strategies to reduce disparities in maternal morbidity and mortality: Patient and provider education | Irrelevant results |
| 1512 | 2016 | Nybo Andersen, Anne-Marie; Gundlund, Anna; Villadsen, Sarah Fredsted | Stillbirth and congenital anomalies in migrants in Europe | Irrelevant results |
| 1513 | 2020 | Blitz, Jeanna; Swisher, Jenna; Sweitzer, BobbieJean | Special Considerations Related to Race, Sex, Gender, and Socioeconomic Status in the Preoperative Evaluation: Part 1: Race, History of Incarceration, and Health Literacy | Irrelevant results |
| 1514 | 2013 | Messina, Jane P.; Mwandagalirwa, Kashamuka; Taylor, Steve M.; Emch, Michael; Meshnick, Steven R. | Spatial and social factors drive anemia in Congolese women | Irrelevant results |
| 1515 | 2018 | Mbusa-Kambale, R.; Mihigo-Akonkwa, M.; Francisca-Isia, N.; Zigabe-Mushamuka, S.; Bwija-Kasengi, J.; Nyakasane-Muhimuzi, A.; Battisti, O.; Mungo-Masumbuko, B. | Somatic growth from birth to 6 months in low birth weight, in Bukavu, South Kivu, Democratic Republic of the Congo | Irrelevant results |
| 1516 | 2008 | Abubakar, Amina; Van de Vijver, Fons; Van Baar, Anneloes; Mbonani, Leonard; Kalu, Raphael; Newton, Charles; Holding, Penny | Socioeconomic status, anthropometric status, and psychomotor development of Kenyan children from resource-limited settings: A path-analytic study | Irrelevant results |
| 1517 | 2021 | Redmond, Charlene; Akinoso-Imran, Abdul Qadr; Heaney, Liam G.; Sheikh, Aziz; Kee, Frank; Busby, John | Socioeconomic disparities in asthma healthcare utilization, exacerbations and mortality: A systematic review and meta-analysis | Irrelevant results |
| 1518 | 2012 | Taborda-Barata, Luís; Potter, Paul C. | Socio-epidemiological Aspects of Respiratory Allergic Diseases in Southern Africa | Irrelevant results |
| 1519 | 2021 | Schmidt, Kim L.; Merrill, Sarah M.; Gill, Randip; Miller, Gregory E.; Gadermann, Anne M.; Kobor, Michael S. | Society to cell: How child poverty gets “Under the Skin” to influence child development and lifelong health | Irrelevant results |
| 1520 | 2009 | Roush, Karen M. | Social Implications of Obstetric Fistula: An Integrative Review | Irrelevant results |
| 1521 | 2021 | Schejter, Dr Yael Dinur; Stepensky, Prof. Polina | Social determinants of health and primary immunodeficiency | Irrelevant results |
| 1522 | 2011 | Yusuf, Hussain R.; Lloyd-Puryear, Michele A.; Grant, Althea M.; Parker, Christopher S.; Creary, Melissa S.; Atrash, Hani K. | Sickle Cell Disease: The Need for a Public Health Agenda | Irrelevant results |
| 1523 | 2014 | Ortayli, Nuriye; Ringheim, Karin; Collins, Lynn; Sladden, Tim | Sexually transmitted infections: progress and challenges since the 1994 International Conference on Population and Development (ICPD) | Irrelevant results |
| 1524 | 2020 | Olaleye, Atinuke O.; Babah, Ochuwa A.; Osuagwu, Chioma S.; Ogunsola, Folasade T.; Afolabi, Bosede B. | Sexually transmitted infections in pregnancy – An update on Chlamydia trachomatis and Neisseria gonorrhoeae | Irrelevant results |
| 1525 | 2018 | Bruckner, Tim A.; Catalano, Ralph | Selection in utero and population health: Theory and typology of research | Irrelevant results |
| 1526 | 2019 | Sheikh, Sana; Qureshi, Rahat Najam; Raza, Farrukh; Memon, Javed; Ahmed, Imran; Vidler, Marianne; Payne, Beth A; Lee, Tang; Sawchuck, Diane; Magee, Laura; von Dadelszen, Peter; Bhutta, Zulfiqar | Self-reported maternal morbidity: Results from the community level interventions for pre-eclampsia (CLIP) baseline survey in Sindh, Pakistan | Irrelevant results |
| 1527 | 2016 | Aya, A. G. M.; Ondze, B.; Ripart, J.; Cuvillon, P. | Seizures in the peripartum period: Epidemiology, diagnosis and management | Irrelevant results |
| 1528 | 2005 | Higgins, Pamela Sangeloty; Wakefield, Dorothy; Cloutier, Michelle M. | Risk Factors for Asthma and Asthma Severity in Nonurban Children in Connecticut | Irrelevant results |
| 1529 | 2018 | Gaccioli, Francesca; Sovio, Ulla; Cook, Emma; Hund, Martin; Charnock-Jones, D Stephen; Smith, Gordon C S | Screening for fetal growth restriction using ultrasound and the sFLT1/PlGF ratio in nulliparous women: a prospective cohort study | Irrelevant results |
| 1530 | 2019 | Back Nielsen, Maj; Carlsson, Jessica; Køster Rimvall, Martin; Petersen, Jørgen Holm; Norredam, Marie | Risk of childhood psychiatric disorders in children of refugee parents with post-traumatic stress disorder: a nationwide, register-based, cohort study | Irrelevant results |
| 1531 | 2008 | Lewis, Gwyneth | Reviewing maternal deaths to make pregnancy safer | Irrelevant results |
| 1532 | 2001 | Berman, Susan; Richardson, Douglas K.; Cohen, Amy P.; Pursley, DeWayne M.; Lieberman, Ellice | Relationship of race and severity of neonatal illness | Irrelevant results |
| 1533 | 2015 | Collins, Bradley N.; Nair, Uma S.; Hovell, Melbourne F.; DiSantis, Katie I.; Jaffe, Karen; Tolley, Natalie M.; Wileyto, E. Paul; Audrain-McGovern, Janet | Reducing Underserved Children’s Exposure to Tobacco Smoke: A Randomized Counseling Trial With Maternal Smokers | Irrelevant results |
| 1534 | 2017 | Luyckx, Valerie A.; Tuttle, Katherine R.; Garcia-Garcia, Guillermo; Gharbi, Mohammed Benghanem; Heerspink, Hiddo J. L.; Johnson, David W.; and al | Reducing major risk factors for chronic kidney disease | Irrelevant results |
| 1535 | 2015 | Lee, Haeok; Kim, Susie; DeMarco, Rosanna; Aronowitz, Teri; Mtengezo, Jasintha; Kang, Younhee; Yang, Youngran; Touch, Chhan; Fitzpatrick, Joyce J. | Recognizing global disparities in health and in health transitions in the 21st century: what can nurses do? | Irrelevant results |
| 1536 | 2021 | Scheftelowitz Cohen, Rachel; Chodik, Gabriel; Eisenberg, Vered H. | Re-evaluating Perinatal Group B Streptococcal screening in Israel – Is it time for a change in policy? | Irrelevant results |
| 1537 | 2011 | Rowland Hogue, Carol J.; Silver, Robert M. | Racial and Ethnic Disparities in United States: Stillbirth Rates: Trends, Risk Factors, and Research Needs | Irrelevant results |
| 1538 | 2021 | Mpotora, Juliana C.; Yahaya, James J.; Ngw'eshemi, Secilia K.; Mwampagatwa, Ipyana H. | Rationale of indications for caesarean delivery and associated factors among primigravidae in Tanzania | Irrelevant results |
| 1539 | 2021 | Fanta, Meghan; Ladzekpo, Deawodi; Unaka, Ndidi | Racism and pediatric health outcomes | Irrelevant results |
| 1540 | 2019 | Guilbert, Theresa; Zeiger, Robert S.; Haselkorn, Tmirah; Iqbal, Ahmar; Alvarez, Cynthia; Mink, David R.; Chipps, Bradley E.; Szefler, Stanley J. | Racial Disparities in Asthma-Related Health Outcomes in Children with Severe/Difficult-to-Treat Asthma | Irrelevant results |
| 1541 | 2000 | Joseph, Christine L. M.; Ownby, Dennis R.; Peterson, Edward L.; Johnson, Christine C. | Racial Differences in Physiologic Parameters Related to Asthma Among Middle-class Children | Irrelevant results |
| 1542 | 2017 | Oland, Alyssa A.; Booster, Genery D.; Bender, Bruce G. | Psychological and lifestyle risk factors for asthma exacerbations and morbidity in children | Irrelevant results |
| 1543 | 2021 | Twilhaar, E. Sabrina; Pierrat, Véronique; Marchand-Martin, Laetitia; Benhammou, Valérie; Kaminski, Monique; Ancel, Pierre-Yves | Profiles of Functioning in 5.5-Year-Old Very Preterm Born Children in France: The EPIPAGE-2 Study | Irrelevant results |
| 1544 | 2005 | Ikossi, Danagra G.; Lazar, Ann A.; Morabito, Diane; Fildes, John; Knudson, M. Margaret | Profile of mothers at risk: An analysis of injury and pregnancy loss in 1,195 trauma patients | Irrelevant results |
| 1545 | 2000 | Bulterys, Marc; Fowler, Mary Glenn | PREVENTION OF HIV INFECTION IN CHILDREN | Irrelevant results |
| 1546 | 2014 | Musa, Taha H.; Musa, Hassan H.; Ali, Elrasheed A.; Musa, Nazik E. | Prevalence of malnutrition among children under five years old in Khartoum State, Sudan | Irrelevant results |
| 1547 | 2021 | Josias, Kasereka Songya; Bangirana, Paul; Rujumba, Joseph; Kakooza-Mwesige, Angelina | Prevalence and factors associated with behavioural problems in children with epilepsy attending Mulago hospital, Uganda: A cross-sectional study | Irrelevant results |
| 1548 | 2021 | Okoji, Uchenna K.; Agim, Nnenna G.; Heath, Candrice R. | Presentation of Common Skin Disorders in Pediatric Patients with Skin of Color | Irrelevant results |
| 1549 | 2021 | Sanjuan, Pilar M.; Fokas, Kathryn; Tonigan, J. Scott; Henry, Melissa C.; Christian, Korinna; Rodriguez, Andrea; Larsen, Jessica; Yonke, Nicole; Leeman, Lawrence | Prenatal maternal posttraumatic stress disorder as a risk factor for adverse birth weight and gestational age outcomes: A systematic review and meta-analysis | Irrelevant results |
| 1550 | 2012 | Channa, Kalavati; Röllin, Halina B.; Nøst, Therese H.; Odland, Jon Ø.; Sandanger, Torkjel M. | Prenatal exposure to DDT in malaria endemic region following indoor residual spraying and in non-malaria coastal regions of South Africa | Irrelevant results |
| 1551 | 2021 | Volk, Heather E.; Perera, Frederica; Braun, Joseph M.; Kingsley, Samantha L.; Gray, Kimberly; Buckley, Jessie; and al | Prenatal air pollution exposure and neurodevelopment: A review and blueprint for a harmonized approach within ECHO | Irrelevant results |
| 1552 | 2016 | Ferranti, Erin P.; Jones, Emily J.; Hernandez, Teri L. | Pregnancy Reveals Evolving Risk for Cardiometabolic Disease in Women | Irrelevant results |
| 1553 | 2011 | Diao, Maboury; Kane, Adama; Ndiaye, Mouhamadou Bamba; Mbaye, Alassane; Bodian, Malick; Dia, Mouhamadoul Mounir; Sarr, Moustapha; Kane, Abdoul; Monsuez, Jean-Jacques; Ba, Serigne Abdou | Pregnancy in women with heart disease in sub-Saharan Africa | Irrelevant results |
| 1554 | 2021 | Briller, JOAN E.; Mogos, MULUBRHAN F.; Muchira, JAMES M.; Piano, MARIANN R. | Pregnancy Associated Heart Failure With Preserved Ejection Fraction: Risk Factors and Maternal Morbidity | Excluded for out of context |
| 1555 | 2021 | Auriti, Cinzia; De Rose, Domenico Umberto; Santisi, Alessandra; Martini, Ludovica; Piersigilli, Fiammetta; Bersani, Iliana; Ronchetti, Maria Paola; Caforio, Leonardo | Pregnancy and viral infections: Mechanisms of fetal damage, diagnosis and prevention of neonatal adverse outcomes from cytomegalovirus to SARS-CoV-2 and Zika virus | Irrelevant results |
| 1556 | 2020 | Naudé, Nadia; Horak, Tracey A.; Fawcus, Susan; Stewart, Chantal J.; Lindow, Stephen W. | Preference for mode of delivery in a low-risk population in Cape Town, South Africa | Irrelevant results |
| 1557 | 2020 | Oikonomou, P.; Tsonis, O.; Paxinos, A.; Gkrozou, F.; Korantzopoulos, P.; Paschopoulos, M. | Preeclampsia and long-term coronary artery disease: How to minimize the odds? | Irrelevant results |
| 1558 | 2012 | Safirstein, Jordan G.; Ro, Angela S.; Grandhi, Sreeram; Wang, Lin; Fett, James D.; Staniloae, Cezar | Predictors of left ventricular recovery in a cohort of peripartum cardiomyopathy patients recruited via the internet | Irrelevant results |
| 1559 | 2013 | Tamesis, Grace P.; Covar, Ronina A.; Strand, Matthew; Liu, Andrew H.; Szefler, Stanley J.; Klinnert, Mary D. | Predictors for Asthma at Age 7 Years for Low-Income Children Enrolled in the Childhood Asthma Prevention Study | Irrelevant results |
| 1560 | 2016 | Poston, Lucilla; Caleyachetty, Rishi; Cnattingius, Sven; Corvalán, Camila; Uauy, Ricardo; Herring, Sharron; Gillman, Matthew W | Preconceptional and maternal obesity: epidemiology and health consequences | Irrelevant results |
| 1561 | 2002 | Barash, Joshua H; Weinstein, Lara Carson | Preconception and prenatal care | Irrelevant results |
| 1562 | 2021 | Kranzler, Henry R.; Washio, Yukiko; Zindel, Leah R.; Wileyto, E. Paul; Srinivas, Sindhu; Hand, Dennis J.; Hoffman, Matthew; Oncken, Cheryl; Schnoll, Robert A. | Placebo-controlled trial of bupropion for smoking cessation in pregnant women | Irrelevant results |
| 1563 | 2021 | Butler, Margaret S.; Young, Sera L.; Tuthill, Emily L. | Perinatal depressive symptoms and breastfeeding behaviors: A systematic literature review and biosocial research agenda | Irrelevant results |
| 1564 | 2017 | Phillips, Cathi; Boyd, Margaret (Peggy) | Perinatal and Neonatal Implications of Sickle Cell Disease | Irrelevant results |
| 1565 | 2018 | Kassile, Telemu; Mmbando, Bruno P.; Lokina, Razack; Mujinja, Phares | Perceptions of caretakers with different socioeconomic status about the harmful outcomes of fever in under-five children in Dodoma region, central Tanzania: A cross-sectional study | Irrelevant results |
| 1566 | 2019 | Scholaske, Laura; Brose, Annette; Spallek, Jacob; Entringer, Sonja | Perceived discrimination and risk of preterm birth among Turkish immigrant women in Germany | Irrelevant results |
| 1567 | 2005 | Marishane, T.; Moodley, J. | Parasuicide in pregnancy | Irrelevant results |
| 1568 | 2005 | Sarto, Gloria | Of disparities and diversity: Where are we? | Irrelevant results |
| 1569 | 2009 | Davis, Esa; Olson, Christine | Obesity in Pregnancy | Irrelevant results |
| 1570 | 2018 | Ruel, Marie T.; Quisumbing, Agnes R.; Balagamwala, Mysbah | Nutrition-sensitive agriculture: What have we learned so far? | Irrelevant results |
| 1571 | 2017 | Britto, Pia R; Lye, Stephen J; Proulx, Kerrie; Yousafzai, Aisha K; Matthews, Stephen G; Vaivada, Tyler; and al | Nurturing care: promoting early childhood development | Irrelevant results |
| 1572 | 2017 | Mendenhall, Emily; Kohrt, Brandon A; Norris, Shane A; Ndetei, David; Prabhakaran, Dorairaj | Non-communicable disease syndemics: poverty, depression, and diabetes among low-income populations | Irrelevant results |
| 1573 | 2019 | Wedderburn, Catherine J; Yeung, Shunmay; Rehman, Andrea M; Stadler, Jacob A M; Nhapi, Raymond T; Barnett, Whitney; Myer, Landon; Gibb, Diana M; Zar, Heather J; Stein, Dan J; Donald, Kirsten A | Neurodevelopment of HIV-exposed uninfected children in South Africa: outcomes from an observational birth cohort study | Irrelevant results |
| 1574 | 2011 | Berti, C.; Biesalski, H. K.; Gärtner, R.; Lapillonne, A.; Pietrzik, K.; Poston, L.; Redman, C.; Koletzko, B.; Cetin, I. | Micronutrients in pregnancy: Current knowledge and unresolved questions | Irrelevant results |
| 1575 | 2020 | Feig, Denice S; Donovan, Lois E; Zinman, Bernard; Sanchez, J Johanna; Asztalos, Elizabeth; Ryan, Edmond A; and al | Metformin in women with type 2 diabetes in pregnancy (MiTy): a multicentre, international, randomised, placebo-controlled trial | Irrelevant results |
| 1576 | 2008 | Grady, Sue C.; Ramírez, Iván J. | Mediating medical risk factors in the residential segregation and low birthweight relationship by race in New York City | Irrelevant results |
| 1577 | 2011 | Acuin, Cecilia S; Khor, Geok Lin; Liabsuetrakul, Tippawan; Achadi, Endang L; Htay, Thein Thein; Firestone, Rebecca; Bhutta, Zulfiqar A | Maternal, neonatal, and child health in southeast Asia: towards greater regional collaboration | Irrelevant results |
| 1578 | 2021 | Hcini, Najeh; Maamri, Fatma; Picone, Olivier; Carod, Jean-Francois; Lambert, Véronique; Mathieu, Meredith; Carles, Gabriel; Pomar, Léo | Maternal, fetal and neonatal outcomes of large series of SARS-CoV-2 positive pregnancies in peripartum period: A single-center prospective comparative study | Irrelevant results |
| 1579 | 2019 | Sotunde, Olusola F.; Laliberte, Alexandra; Weiler, Hope A. | Maternal risk factors and newborn infant vitamin D status: a scoping literature review | Irrelevant results |
| 1580 | 2018 | Cook, Natalie; Ayers, Susan; Horsch, Antje | Maternal posttraumatic stress disorder during the perinatal period and child outcomes: A systematic review | Irrelevant results |
| 1581 | 2014 | Nove, Andrea; Matthews, Zoë; Neal, Sarah; Camacho, Alma Virginia | Maternal mortality in adolescents compared with women of other ages: evidence from 144 countries | Irrelevant results |
| 1582 | 2005 | Goldenberg, Robert L.; Culhane, Jennifer F.; Johnson, Derek C. | Maternal Infection and Adverse Fetal and Neonatal Outcomes | Irrelevant results |
| 1583 | 2021 | Qu, Yanji; Lin, Shao; Bloom, Michael S.; Wang, Ximeng; Ye, Bo; Nie, Zhiqiang; Ou, Yanqiu; Mai, Jinzhuang; Wu, Yong; Gao, Xiangmin; Xiao, Xiaohua; Tan, Hongzhuan; Liu, Xiaoqing; Chen, Jimei; Zhuang, Jian | Maternal folic acid supplementation mediates the associations between maternal socioeconomic status and congenital heart diseases in offspring | Irrelevant results |
| 1584 | 2021 | Wilson, R. D.; O'Connor, D. L. | Maternal folic acid and multivitamin supplementation: International clinical evidence with considerations for the prevention of folate-sensitive birth defects | Irrelevant results |
| 1585 | 2019 | Kumari, Shweta; Garg, Neelima; Kumar, Amod; Guru, Pawas Kumar Indra; Ansari, Sharafat; Anwar, Shadab; Singh, Krishn Pratap; and al | Maternal and severe anaemia in delivering women is associated with risk of preterm and low birth weight: A cross sectional study from Jharkhand, India | Irrelevant results |
| 1586 | 2013 | Lindquist, Anthea; Knight, Marian; Kurinczuk, Jennifer J. | Variation in severe maternal morbidity according to socioeconomic position: a UK national case–control study | Irrelevant results |
| 1587 | 2020 | Tura, Abera Kenay; Scherjon, Sicco; van Roosmalen, Jos; Zwart, Joost; Stekelenburg, Jelle; van den Akker, Thomas | Surviving mothers and lost babies - burden of stillbirths and neonatal deaths among women with maternal near miss in eastern Ethiopia: a prospective cohort study | Irrelevant results |
| 1588 | 2015 | Nakimuli, Annettee; Mbalinda, Scovia N.; Nabirye, Rose C.; Kakaire, Othman; Nakubulwa, Sarah; Osinde, Michael O.; Kakande, Nelson; Kaye, Dan K. | Still births, neonatal deaths and neonatal near miss cases attributable to severe obstetric complications: a prospective cohort study in two referral hospitals in Uganda | Irrelevant results |
| 1589 | 2019 | Fernandes, K. G.; Costa, M. L.; Haddad, S. M.; Parpinelli, M. A.; Sousa, M. H.; Cecatti, J. G.; Group, the Brazilian Network for Surveillance of Severe Maternal Morbidity Study | Skin Color and Severe Maternal Outcomes: Evidence from the Brazilian Network for Surveillance of Severe Maternal Morbidity | Irrelevant results |
| 1590 | 2005 | Filippi, Veronique; Ronsmans, Carine; Gohou, Valerie; Goufodji, Sourou; Lardi, Mohamed; Sahel, Amina; Saizonou, Jacques; Brouwere, Vincent De | Maternity wards or emergency obstetric rooms? Incidence of near-miss events in African hospitals | Irrelevant results |
| 1591 | 2020 | Heemelaar, Steffie; Josef, Mirjam; Diener, Zoe; Chipeio, Melody; Stekelenburg, Jelle; van den Akker, Thomas; Mackenzie, Shonag | Maternal near-miss surveillance, Namibia | Irrelevant results |
| 1592 | 2016 | Nakimuli, Annettee; Nakubulwa, Sarah; Kakaire, Othman; Osinde, Michael O.; Mbalinda, Scovia N.; Nabirye, Rose C.; Kakande, Nelson; Kaye, Dan K. | Maternal near misses from two referral hospitals in Uganda: a prospective cohort study on incidence, determinants and prognostic factors | Irrelevant results |
| 1593 | 2020 | Manyahi, Jane R.; Mgaya, Hans; Said, Ali | Maternal near miss and mortality attributable to hypertensive disorders in a tertiary hospital, Tanzania; a cross-sectional study | Irrelevant results |
| 1594 | 2018 | Woldeyes, Wondimagegnehu Sisay; Asefa, Dejene; Muleta, Geremew | Incidence and determinants of severe maternal outcome in Jimma University teaching hospital, south-West Ethiopia: a prospective cross-sectional study | Not specific to MMS |
| 1595 | 2020 | Tekelab, Tesfalidet; Chojenta, Catherine; Smith, Roger; Loxton, Deborah | Incidence and determinants of neonatal near miss in south Ethiopia: a prospective cohort study | Irrelevant results |
| 1596 | 2015 | Peters, Micah D. J.; Godfrey, Christina M.; Khalil, Hanan; McInerney, Patricia; Parker, Deborah; Soares, Cassia Baldini | Guidance for conducting systematic scoping reviews | Irrelevant results |
| 1597 | 2019 | Mersha, Abera; Bante, Agegnehu; Shibiru, Shitaye | Factors associated with neonatal near-miss in selected hospitals of Gamo and Gofa zones, southern Ethiopia: nested case-control study | Irrelevant results |
| 1598 | 2020 | Dessalegn, Fikadu Nugusu; Astawesegn, Feleke Hailemichael; Hankalo, Nana Chea | Factors Associated with Maternal Near Miss among Women Admitted in West Arsi Zone Public Hospitals, Ethiopia: Unmatched Case-Control Study | Included |
| 1599 | 2014 | Nair, Manisha; Kurinczuk, Jennifer J.; Knight, Marian | Ethnic Variations in Severe Maternal Morbidity in the UK– A Case Control Study | Irrelevant results |
| 1600 | 2020 | Kumela, Lemi; Tilahun, Temesgen; Kifle, Demeke | Determinants of maternal near miss in Western Ethiopia | Included |
| 1601 | 2020 | ELMeneza, Safaa; AbuShady, Mariam | Anonymous reporting of medical errors from The Egyptian Neonatal Safety Training Network | Irrelevant results |
| 1602 | 2021 | Atuhairwe, Susan; Gemzell-Danielsson, Kristina; Byamugisha, Josaphat; Kaharuza, Frank; Tumwesigye, Nazarius Mbona; Hanson, Claudia | Abortion-related near-miss morbidity and mortality in 43 health facilities with differences in readiness to provide abortion care in Uganda | Irrelevant results |
| 1603 | 2016 | Tricco, Andrea C.; Lillie, Erin; Zarin, Wasifa; O’Brien, Kelly; Colquhoun, Heather; Kastner, Monika; Levac, Danielle; Ng, Carmen; Sharpe, Jane Pearson; Wilson, Katherine; Kenny, Meghan; Warren, Rachel; Wilson, Charlotte; Stelfox, Henry T.; Straus, Sharon E. | A scoping review on the conduct and reporting of scoping reviews | Irrelevant results |
| 1604 | 2011 | Bramham, Kate; Briley, Annette L.; Seed, Paul; Poston, Lucilla; Shennan, Andrew H.; Chappell, Lucy C. | Adverse maternal and perinatal outcomes in women with previous preeclampsia: a prospective study | Irrelevant results |
| 1605 | 2016 | Ouédraogo, Adja M.; Ouédraogo, Henri G.; Baguiya, Adama; Millogo, Tieba; Somé, Anthony; Kouanda, Seni | A case–control study of risk factors for maternal mortality in Burkina Faso in 2014 | Irrelevant results |
| 1606 | 2005 | Conde-Agudelo, Agustin; Belizán, José M.; Lammers, Cristina | Maternal-perinatal morbidity and mortality associated with adolescent pregnancy in Latin America: Cross-sectional study | Excluded for out of context |
| 1607 | 2015 | Allen, Victoria M.; Baskett, Thomas F.; O'Connell, Colleen M. | Type of Labour in the First Pregnancy and Cumulative Maternal Morbidity | Excluded for out of context |
| 1608 | 2016 | Litorp, Helena; Rööst, Mattias; Kidanto, Hussein L.; Nyström, Lennarth; Essén, Birgitta | The effects of previous cesarean deliveries on severe maternal and adverse perinatal outcomes at a university hospital in Tanzania | Irrelevant results |
| 1609 | 2017 | Marocchini, M.; Lauféron, J.; Quantin, C.; Sagot, P. | Postpartum hemorrhage with transfusion: Trends, near misses, risk factors and management at the scale of a perinatal network | Excluded for out of context |
| 1610 | 2021 | Fonseca, Andreia; Ayres de Campos, Diogo | Maternal morbidity and mortality due to placenta accreta spectrum disorders | Irrelevant results |
| 1611 | 2015 | Mazhar, Syeda B.; Batool, Afshan; Emanuel, Angela; Khan, Arif T.; Bhutta, Shireen | Severe maternal outcomes and their predictors among Pakistani women in the WHO Multicountry Survey on Maternal and Newborn Health | Irrelevant results |
| 1612 | 2022 | Teal, E. Nicole; Appiagyei, Ashley; Sheffield-Abdullah, Karen; Manuck, Tracy A. | Differences in disease severity and delivery gestational age between black and white patients with hypertensive disorders of pregnancy | Irrelevant results |
| 1613 | 2018 | Gupta, Megha; Greene, Naomi; Kilpatrick, Sarah J. | Timely treatment of severe maternal hypertension and reduction in severe maternal morbidity | Excluded for out of context |
| 1614 | 2014 | Kilpatrick, Sara J.; Berg, Cynthia; Bernstein, Peter; Bingham, Debra; Delgado, Ana; Callaghan, Willia M.; Harris, Karen; Lanni, Susan; Mahoney, Jeanne; Main, Elliot; Nacht, Amy; Schellpfeffer, Michael; Westover, Thomas; Harper, Margaret | Standardized Severe Maternal Morbidity Review: Rationale and Process | Irrelevant results |
| 1615 | 2022 | Malhamé, Isabelle; Bublitz, Margaret H.; Wilson, Danielle; Sanapo, Laura; Rochin, Elizabeth; Bourjeily, Ghada | Sleep disordered breathing and the risk of severe maternal morbidity in women with preeclampsia: A population-based study | Excluded for out of context |
| 1616 | 2022 | Sabr, Yasser; Lisonkova, Sarka; Skoll, Amanda; Brant, Rollin; Velez, Maria P.; Joseph, K. S. | Severe Maternal Morbidity and Maternal Mortality Associated with Assisted Reproductive Technology | Excluded for out of context |
| 1617 | 2010 | Pacagnella, Rodolfo C.; Cecatti, Jose G.; Camargo, Rodrigo P.; Silveira, Carla; Zanardi, Dulce T.; Souza, Joao P.; Parpinelli, Mary A.; Haddad, Samira M. | Rationale for a Long-term Evaluation of the Consequences of Potentially Life-threatening Maternal Conditions and Maternal “Near-miss” Incidents Using a Multidimensional Approach | Irrelevant results |
| 1618 | 2020 | Sirgant, Delphine; Rességuier, Noémie; d’Ercole, Claude; Auquier, Pascal; Tosello, Barthélémy; Blanc, Julie | Lower gestational age is associated with severe maternal morbidity of preterm cesarean delivery | Excluded for out of context |
| 1619 | 2010 | Liu, Shiliang; Joseph, K. S.; Bartholomew, Sharon; Fahey, John; Lee, Lily; Allen, Alexander C.; Kramer, Michael S.; Sauve, Reg; Young, David C.; Liston, Robert M. | Temporal Trends and Regional Variations in Severe Maternal Morbidity in Canada, 2003 to 2007 | Excluded for out of context |
| 1620 | 2019 | Dzakpasu, Susie; Deb-Rinker, Paromita; Arbour, Laura; Darling, Elizabeth K.; Kramer, Michael S.; Liu, Shiliang; Luo, Wei; Murphy, Phil A.; Nelson, Chantal; Ray, Joel G.; Scott, Heather; VandenHof, Michiel; Joseph, K. S. | Severe Maternal Morbidity in Canada: Temporal Trends and Regional Variations, 2003-2016 | Excluded for out of context |
| 1621 | 2010 | Joseph, K. S.; Liu, Shiliang; Rouleau, Jocelyn; Kirby, Russell S.; Kramer, Michael S.; Sauve, Reg; Fraser, William D.; Young, David C.; Liston, Robert M. | Severe Maternal Morbidity in Canada, 2003 to 2007: Surveillance Using Routine Hospitalization Data and ICD-10CA Codes | Excluded for out of context |
| 1622 | 2022 | Snelgrove, John W.; Simpson, Andrea N.; Sutradhar, Rinku; Everett, Karl; Liu, Ning; Baxter, Nancy N. | Preeclampsia and Severe Maternal Morbidity During the COVID-19 Pandemic: A Population-Based Cohort Study in Ontario, Canada | Irrelevant results |
| 1623 | 2021 | Ueda, Akihiko; Chigusa, Yoshitsugu; Mogami, Haruta; Nakita, Baku; Ohtera, Shosuke; Kato, Genta; Horie, Akihito; Mandai, Masaki; Kondoh, Eiji | Maternal near-miss attributable to haemorrhagic stroke in patients with hypertensive disorders of pregnancy in Japan: A national cohort study | Excluded for out of context |
| 1624 | 2010 | Allen, Victoria M.; Campbell, Melanie; Carson, George; Fraser, William; Liston, Robert M.; Walker, Mark; Barrett, Jon | Maternal Mortality and Severe Maternal Morbidity Surveillance in Canada | Excluded for out of context |
| 1625 | 2013 | Shen, Fang-Rong; Liu, Ming; Zhang, Xia; Yang, Weiwen; Chen, You-Guo | Factors associated with maternal near-miss morbidity and mortality in Kowloon Hospital, Suzhou, China | Excluded for out of context |
| 1626 | 2011 | Clark, Erin A. S.; Silver, Robert M. | Long-term maternal morbidity associated with repeat cesarean delivery | Irrelevant results |
| 1627 | 2016 | Gibson, Kelly S.; Waters, Thaddeus P.; Bailit, Jennifer L. | A risk of waiting: the weekly incidence of hypertensive disorders and associated maternal and neonatal morbidity in low-risk term pregnancies | Irrelevant results |
| 1628 | 2022 | Detlefs, Sarah E.; Jochum, Michael D.; Salmanian, Bahram; McKinney, Jennifer R.; Aagaard, Kjersti M. | The impact of response to iron therapy on maternal and neonatal outcomes among pregnant women with anemia | Irrelevant results |
| 1629 | 2014 | Pinas Carillo, Ana; Chandraharan, Edwin | Postpartum haemorrhage and haematological management | Irrelevant results |
| 1630 | 2013 | Fawcus, Sue; Moodley, Jagidesa | Postpartum haemorhage associated with caesarean section and caesarean hysterectomy | Irrelevant results |
| 1631 | 2020 | Freese, Kyle E.; Bodnar, Lisa M.; Brooks, Maria M.; McTigue, Kathleen; Himes, Katherine P. | Population-attributable fraction of risk factors for severe maternal morbidity | Irrelevant results |
| 1632 | 2008 | Souza, Joao P.; Parpinelli, Mary A.; Amaral, Eliana; Cecatti, Jose G. | Population surveys using validated questionnaires provided useful information on the prevalence of maternal morbidities | Irrelevant results |
| 1633 | 2016 | Puthussery, Shuby | Perinatal outcomes among migrant mothers in the United Kingdom: Is it a matter of biology, behaviour, policy, social determinants or access to health care? | Irrelevant results |
| 1634 | 2021 | Banke-Thomas, Aduragbemi; Avoka, Cephas; Olaniran, Abimbola; Balogun, Mobolanle; Wright, Ololade; Ekerin, Olabode; Benova, Lenka | Patterns, travel to care and factors influencing obstetric referral: Evidence from Nigeria's most urbanised state | Irrelevant results |
| 1635 | 2021 | Peahl, Alex F.; Powell, Allison; Berlin, Hanna; Smith, Roger D.; Krans, Elizabeth; Waljee, Jennifer; Dalton, Vanessa K.; Heisler, Michele; Moniz, Michelle H. | Patient and provider perspectives of a new prenatal care model introduced in response to the coronavirus disease 2019 pandemic | Irrelevant results |
| 1636 | 2020 | Zuckerwise, Lisa C.; Craig, Amanda M.; Newton, JM.; Zhao, Shillin; Bennett, Kelly A.; Crispens, Marta A. | Outcomes following a clinical algorithm allowing for delayed hysterectomy in the management of severe placenta accreta spectrum | Irrelevant results |
| 1637 | 2016 | Ozimek, John A.; Eddins, Rhonda M.; Greene, Naomi; Karagyozyan, Daniela; Pak, Sujane; Wong, Melissa; Zakowski, Mark; Kilpatrick, Sarah J. | Opportunities for improvement in care among women with severe maternal morbidity | Irrelevant results |
| 1638 | 2021 | McGregor, Alecia J.; Hung, Peiyin; Garman, David; Amutah-Onukagha, Ndidiamaka; Cooper, Joy A. | Obstetrical unit closures and racial and ethnic differences in severe maternal morbidity in the state of New Jersey | Excluded for out of context |
| 1639 | 2006 | Wall, L Lewis | Obstetric vesicovaginal fistula as an international public-health problem | Irrelevant results |
| 1640 | 2009 | Zheng, Alice X.; Anderson, Frank W. J. | Obstetric fistula in low-income countries | Irrelevant results |
| 1641 | 2022 | Leonard, Stephanie A.; Main, Elliott K.; Lyell, Deirdre J.; Carmichael, Suzan L.; Kennedy, Chris J.; Johnson, Christina; Mujahid, Mahasin S. | Obstetric comorbidity scores and disparities in severe maternal morbidity across marginalized groups | Excluded for out of context |
| 1642 | 2010 | Paech, Michael; Sinha, Aneeta | Obstetric audit and its implications for obstetric anaesthesia | Irrelevant results |
| 1643 | 2007 | Prince, Martin; Patel, Vikram; Saxena, Shekhar; Maj, Mario; Maselko, Joanna; Phillips, Michael R; Rahman, Atif | No health without mental health | Irrelevant results |
| 1644 | 2017 | Rosenbloom, Joshua I.; Stout, Molly J.; Tuuli, Methodius G.; Woolfolk, Candice L.; López, Julia D.; Macones, George A.; Cahill, Alison G. | New labor management guidelines and changes in cesarean delivery patterns | Irrelevant results |
| 1645 | 2010 | Doumouchtsis, Stergios K.; Arulkumaran, Sabaratnam | Morbidly adherent placenta | Irrelevant results |
| 1646 | 2015 | Creanga, Andreea A.; Bateman, Brian T.; Butwick, Alexander J.; Raleigh, Lindsay; Maeda, Ayumi; Kuklina, Elena; Callaghan, William M. | Morbidity associated with cesarean delivery in the United States: is placenta accreta an increasingly important contributor? | Excluded for out of context |
| 1647 | 2006 | Geller, Stacie E.; Cox, Suzanne M.; Callaghan, William M.; Berg, Cynthia J. | Morbidity and mortality in pregnancy: Laying the Groundwork for Safe Motherhood | Irrelevant results |
| 1648 | 2010 | Widmer, Mariana; Blum, Jennifer; Hofmeyr, G Justus; Carroli, Guillermo; Abdel-Aleem, Hany; Lumbiganon, Pisake; and al | Misoprostol as an adjunct to standard uterotonics for treatment of post-partum haemorrhage: a multicentre, double-blind randomised trial | Irrelevant results |
| 1649 | 2002 | Björklund, Kenneth | Minimally invasive surgery for obstructed labour: a review of symphysiotomy during the twentieth century (including 5000 cases) | Irrelevant results |
| 1650 | 2010 | Gagnon, Anita J.; Zimbeck, Meg; Zeitlin, Jennifer | Migration and perinatal health surveillance: An international Delphi survey | Irrelevant results |
| 1651 | 2004 | Dudgeon, Matthew R; Inhorn, Marcia C | Men's influences on women's reproductive health: medical anthropological perspectives | Irrelevant results |
| 1652 | 2016 | El-Messidi, Amira; Czuzoj-Shulman, Nicholas; Spence, Andrea R.; Abenhaim, Haim Arie | Medical and obstetric outcomes among pregnant women with tuberculosis: a population-based study of 7.8 million births | Irrelevant results |
| 1653 | 2016 | Lim, Stephen S; Allen, Kate; Bhutta, Zulfiqar A; Dandona, Lalit; Forouzanfar, Mohammad H; Fullman, Nancy; and al | Measuring the health-related Sustainable Development Goals in 188 countries: a baseline analysis from the Global Burden of Disease Study 2015 | Irrelevant results |
| 1654 | 2017 | Fullman, Nancy; Barber, Ryan M; Abajobir, Amanuel Alemu; Abate, Kalkidan Hassen; Abbafati, Cristiana; Abbas, Kaja M; and al | Measuring progress and projecting attainment on the basis of past trends of the health-related Sustainable Development Goals in 188 countries: an analysis from the Global Burden of Disease Study 2016 | Irrelevant results |
| 1655 | 2013 | Chaiworapongsa, Tinnakorn; Romero, Roberto; Korzeniewski, Steven J.; Kusanovic, Juan Pedro; Soto, Eleazar; Lam, Jennifer; Dong, Zhong; Than, Nandor G.; Yeo, Lami; Hernandez-Andrade, Edgar; Conde-Agudelo, Agustín; Hassan, Sonia S. | Maternal plasma concentrations of angiogenic/antiangiogenic factors in the third trimester of pregnancy to identify the patient at risk for stillbirth at or near term and severe late preeclampsia | Irrelevant results |
| 1656 | 2014 | Adu-Bonsaffoh, Kwame; Obed, Samuel A.; Seffah, Joseph D. | Maternal outcomes of hypertensive disorders in pregnancy at Korle Bu Teaching Hospital, Ghana | Irrelevant results |
| 1657 | 2016 | O’Malley, Eimer G.; Popivanov, Petar; Fergus, Ann; Tan, Terry; Byrne, Bridgette | Maternal near miss: what lies beneath? | Irrelevant results |
| 1658 | 2008 | Drife, James | Maternal mortality in well-resourced countries: is there still a need for confidential enquiries? | Irrelevant results |
| 1659 | 2012 | O'Dwyer, Vicky; Hogan, Jennifer L.; Farah, Nadine; Kennelly, Mairead M.; Fitzpatrick, Christopher; Turner, Michael J. | Maternal mortality and the rising cesarean rate | Irrelevant results |
| 1660 | 2011 | Dekker, Ruth R.; Schutte, Joke M.; Stekelenburg, Jelle; Zwart, Joost J.; van Roosmalen, Jos | Maternal mortality and severe maternal morbidity from acute fatty liver of pregnancy in the Netherlands | Excluded for out of context |
| 1661 | 2015 | Lesage, N.; Deneux Tharaux, C.; Saucedo, M.; Habibi, A.; Galacteros, F.; Girot, R.; Bouvier Colle, M. H.; Kayem, G. | Maternal mortality among women with sickle-cell disease in France, 1996–2009 | Irrelevant results |
| 1662 | 2009 | Neilson, James P. | Maternal mortality | Irrelevant results |
| 1663 | 2022 | Sklar, Ariel; Sheeder, Jeanelle; Davis, Anne R.; Wilson, Carrie; Teal, Stephanie B. | Maternal morbidity after preterm premature rupture of membranes at <24 weeks’ gestation | Irrelevant results |
| 1664 | 2020 | Romagano, Matthew P.; Fofah, Onajovwe; Apuzzio, Joseph J.; Williams, Shauna F.; Gittens-Williams, Lisa | Maternal morbidity after early preterm delivery (23–28 weeks) | Irrelevant results |
| 1665 | 2006 | Filippi, Véronique; Ronsmans, Carine; Campbell, Oona MR; Graham, Wendy J; Mills, Anne; Borghi, Jo; Koblinsky, Marjorie; Osrin, David | Maternal health in poor countries: the broader context and a call for action | Irrelevant results |
| 1666 | 2022 | Martin, Jane; Croteau, Angelica; Velasco-Gonzalez, Cruz; Gastanaduy, Mariella; Huttner, Madelyn; Saeed, Rula; Niazi, Sahar; Chisholm, Sarah; Mussarat, Naiha; Morgan, John; Williams, F. B. Will; Biggio, Joseph | Maternal early warning criteria predict postpartum severe maternal morbidity and mortality after delivery hospitalization discharge: a case–control study | Irrelevant results |
| 1667 | 2013 | Adu-Bonsaffoh, Kwame; Oppong, Samuel A.; Binlinla, Godwin; Obed, Samuel A. | Maternal deaths attributable to hypertensive disorders in a tertiary hospital in Ghana | Irrelevant results |
| 1668 | 2021 | Harrison, Rachel K.; Lauhon, Samantha R.; Colvin, Zachary A.; McIntosh, Jennifer J. | Maternal anemia and severe maternal morbidity in a US cohort | Excluded for out of context |
| 1669 | 2015 | Crane, Joan M. G.; Magee, Laura A.; Lee, Tang; Synnes, Anne; von Dadelszen, Peter; Dahlgren, Leanne; De Silva, Dane A.; Liston, Robert | Maternal and Perinatal Outcomes of Pregnancies Delivered at 23 Weeks’ Gestation | Irrelevant results |
| 1670 | 2019 | Sobhy, Soha; Arroyo-Manzano, David; Murugesu, Nilaani; Karthikeyan, Gayathri; Kumar, Vinoth; Kaur, Inderjeet; Fernandez, Evita; Gundabattula, Sirisha Rao; Betran, Ana Pilar; Khan, Khalid; Zamora, Javier; Thangaratinam, Shakila | Maternal and perinatal mortality and complications associated with caesarean section in low-income and middle-income countries: a systematic review and meta-analysis | Irrelevant results |
| 1671 | 2008 | Black, Robert E; Allen, Lindsay H; Bhutta, Zulfiqar A; Caulfield, Laura E; de Onis, Mercedes; Ezzati, Majid; Mathers, Colin; Rivera, Juan | Maternal and child undernutrition: global and regional exposures and health consequences | Irrelevant results |
| 1672 | 2008 | Victora, Cesar G; Adair, Linda; Fall, Caroline; Hallal, Pedro C; Martorell, Reynaldo; Richter, Linda; Sachdev, Harshpal Singh | Maternal and child undernutrition: consequences for adult health and human capital | Irrelevant results |
| 1673 | 2013 | Black, Robert E; Victora, Cesar G; Walker, Susan P; Bhutta, Zulfiqar A; Christian, Parul; de Onis, Mercedes; Ezzati, Majid; Grantham-McGregor, Sally; Katz, Joanne; Martorell, Reynaldo; Uauy, Ricardo | Maternal and child undernutrition and overweight in low-income and middle-income countries | Irrelevant results |
| 1674 | 2022 | Carr, Rebecca C.; McKinney, David N.; Cherry, Amy L.; Defranco, Emily A. | Maternal age-specific drivers of severe maternal morbidity | Excluded for out of context |
| 1675 | 2018 | Sheen, Jean-Ju; Wright, Jason D.; Goffman, Dena; Kern-Goldberger, Adina R.; Booker, Whitney; Siddiq, Zainab; D’Alton, Mary E.; Friedman, Alexander M. | Maternal age and risk for adverse outcomes | Irrelevant results |
| 1676 | 2011 | McIntyre, H. David; Oats, Jeremy J. N.; Zeck, Willibald; Seshiah, V.; Hod, Moshe | Matching diagnosis and management of diabetes in pregnancy to local priorities and resources: An international approach | Irrelevant results |
| 1677 | 2015 | Metz, Torri D.; Stickrath, Elaine H. | Marijuana use in pregnancy and lactation: a review of the evidence | Irrelevant results |
| 1678 | 2018 | Dominguez, Jennifer E.; Street, Linda; Louis, Judette | Management of Obstructive Sleep Apnea in Pregnancy | Irrelevant results |
| 1679 | 2017 | Seligman, K.; Ramachandran, B.; Hegde, P.; Riley, E. T.; El-Sayed, Y. Y.; Nelson, L. M.; Butwick, A. J. | Obstetric interventions and maternal morbidity among women who experience severe postpartum hemorrhage during cesarean delivery | Irrelevant results |
| 1680 | 2016 | Agostino, Margaret-Rose; Wilson, Barbara; Byfield, Renee | Identifying Potentially Preventable Elements in Severe Adverse Maternal Events | Irrelevant results |
| 1681 | 2016 | Bingham, Debra; Cornell, Andria | Expert Panel to Track Nurses' Effect on Maternal Morbidity and Mortality | Irrelevant results |
| 1682 | 2021 | Attali, Emmanuel; Yogev, Yariv | The impact of advanced maternal age on pregnancy outcome | Irrelevant results |
| 1683 | 2020 | Makokha-Sandell, Henrik; Mgaya, Andrew; Belachew, Johanna; Litorp, Helena; Hussein, Kidanto; Essén, Birgitta | Low use of vacuum extraction: Health care Professionals’ Perspective in a University Hospital, Dar es Salaam | Irrelevant results |
| 1684 | 2010 | Karolinski, Ariel; Mazzoni, Agustina; Belizán, José M.; Althabe, Fernando; Bergel, Eduardo; Buekens, Pierre | Lost opportunities for effective management of obstetric conditions to reduce maternal mortality and severe maternal morbidity in Argentina and Uruguay | Excluded for out of context |
| 1685 | 2021 | Garg, Bharti; Darney, Blair; Pilliod, Rachel A.; Caughey, Aaron B. | Long and short interpregnancy intervals increase severe maternal morbidity | Excluded for out of context |
| 1686 | 2022 | Jeffers, Noelene K.; Berger, Blair O.; Marea, Christina X.; Gemmill, Alison | Investigating the impact of structural racism on black birthing people - associations between racialized economic segregation, incarceration inequality, and severe maternal morbidity | Irrelevant results |
| 1687 | 2021 | Bergo, Cara Jane; Handler, Arden; Geller, Stacie; Grobman, William A.; Awadalla, Saria; Rankin, Kristin | Interpregnancy Interval and Severe Maternal Morbidity in Iowa, 2009 to 2014 | Excluded for out of context |
| 1688 | 2011 | McEniry, Mary | Infant mortality, season of birth and the health of older Puerto Rican adults | Irrelevant results |
| 1689 | 2020 | Murugappan, Gayathree; Li, Shufeng; Lathi, Ruth B.; Baker, Valerie L.; Luke, Barbara; Eisenberg, Michael L. | Increased risk of severe maternal morbidity among infertile women: analysis of US claims data | Excluded for out of context |
| 1690 | 2013 | Lisonkova, Sarka; Joseph, K. S. | Incidence of preeclampsia: risk factors and outcomes associated with early- versus late-onset disease | Irrelevant results |
| 1691 | 2016 | Tang, Jennifer H.; Kaliti, Charlotte; Bengtson, Angela; Hayat, Sumera; Chimala, Eveles; MacLeod, Rachel; Kaliti, Stephen; Sisya, Fanny; Mwale, Mwawi; Wilkinson, Jeffrey | Improvement and retention of emergency obstetrics and neonatal care knowledge and skills in a hospital mentorship program in Lilongwe, Malawi | Irrelevant results |
| 1692 | 2011 | James, Alison; Endacott, Ruth; Stenhouse, Elizabeth | Identifying women requiring maternity high dependency care | Irrelevant results |
| 1693 | 2009 | Filippi, Véronique; Richard, Fabienne; Lange, Isabelle; Ouattara, Fatoumata | Identifying barriers from home to the appropriate hospital through near-miss audits in developing countries | Irrelevant results |
| 1694 | 2020 | Kantorowska, Agata; Heiselman, Cassandra J.; Halpern, Tara A.; Akerman, Meredith B.; Elsayad, Ashley; Muscat, Jolene C.; Sicuranza, Genevieve B.; Vintzileos, Anthony M.; Heo, Hye J. | Identification of factors associated with delayed treatment of obstetric hypertensive emergencies | Irrelevant results |
| 1695 | 2015 | Poulter, Neil R; Prabhakaran, Dorairaj; Caulfield, Mark | Hypertension | Irrelevant results |
| 1696 | 2020 | Leppälä, Satu; Lamminpää, Reeta; Gissler, Mika; Vehviläinen-Julkunen, Katri | Humanitarian migrant women's experiences of maternity care in Nordic countries: A systematic integrative review of qualitative research | Irrelevant results |
| 1697 | 2010 | Jayaweera, Hiranthi; Quigley, Maria A. | Health status, health behaviour and healthcare use among migrants in the UK: Evidence from mothers in the Millennium Cohort Study | Irrelevant results |
| 1698 | 2007 | Filippi, Véronique; Ganaba, Rasmané; Baggaley, Rebecca F; Marshall, Tom; Storeng, Katerini T; Sombié, Issiaka; Ouattara, Fatoumata; Ouedraogo, Thomas; Akoum, Mélanie; Meda, Nicolas | Health of women after severe obstetric complications in Burkina Faso: a longitudinal study | Irrelevant results |
| 1699 | 2014 | Kassebaum, Nicholas J; Bertozzi-Villa, Amelia; Coggeshall, Megan S; Shackelford, Katya A; Steiner, Caitlyn; Heuton, Kyle R; | Global, regional, and national levels and causes of maternal mortality during 1990–2013: a systematic analysis for the Global Burden of Disease Study 2013 | Irrelevant results |
| 1700 | 2015 | Vos, Theo; Barber, Ryan M; Bell, Brad; Bertozzi-Villa, Amelia; Biryukov, Stan; Bolliger, Ian; and al | Global, regional, and national incidence, prevalence, and years lived with disability for 301 acute and chronic diseases and injuries in 188 countries, 1990–2013: a systematic analysis for the Global Burden of Disease Study 2013 | Irrelevant results |
| 1701 | 2017 | Hay, Simon I; Abajobir, Amanuel Alemu; Abate, Kalkidan Hassen; Abbafati, Cristiana; Abbas, Kaja M; Abd-Allah, Foad; and al | Global, regional, and national disability-adjusted life-years (DALYs) for 333 diseases and injuries and healthy life expectancy (HALE) for 195 countries and territories, 1990–2016: a systematic analysis for the Global Burden of Disease Study 2016 | Irrelevant results |
| 1702 | 2018 | Boerma, Ties; Ronsmans, Carine; Melesse, Dessalegn Y; Barros, Aluisio J D; Barros, Fernando C; Juan, Liang; Moller, Ann-Beth; Say, Lale; Hosseinpoor, Ahmad Reza; Yi, Mu; de Lyra Rabello Neto, Dácio; Temmerman, Marleen | Global epidemiology of use of and disparities in caesarean sections | Irrelevant results |
| 1703 | 2013 | Nair, Harish; Simões, Eric AF; Rudan, Igor; Gessner, Bradford D; Azziz-Baumgartner, Eduardo; Zhang, Jian Shayne F; and al | Global and regional burden of hospital admissions for severe acute lower respiratory infections in young children in 2010: a systematic analysis | Irrelevant results |
| 1704 | 2013 | Liu, Shiliang; Joseph, K. S.; Hutcheon, Jennifer A.; Bartholomew, Sharon; León, Juan Andrés; Walker, Mark; Kramer, Michael S.; Liston, Robert M. | Gestational age–specific severe maternal morbidity associated with labor induction | Excluded for out of context |
| 1705 | 2021 | Holcomb, Denisse S.; Pengetnze, Yolande; Steele, Ashley; Karam, Albert; Spong, Catherine; Nelson, David B. | Geographic barriers to prenatal care access and their consequences | Irrelevant results |
| 1706 | 2002 | Høj, L; da Silva, D; Hedegaard, K; Sandström, A; Aaby, P | Factors associated with maternal mortality in rural Guinea-Bissau. A longitudinal population-based study | Irrelevant results |
| 1707 | 2009 | Oliveira Neto, Antonio F.; Parpinelli, Mary A.; Cecatti, José G.; Souza, João P.; Sousa, Maria H. | Factors associated with maternal death in women admitted to an intensive care unit with severe maternal morbidity | Irrelevant results |
| 1708 | 2021 | Deshmukh, Uma S.; Lundsberg, Lisbet S.; Culhane, Jennifer F.; Partridge, Caitlin; Reddy, Uma M.; Merriam, Audrey A.; Son, Moeun | Factors associated with appropriate treatment of acute-onset severe obstetrical hypertension | Irrelevant results |
| 1709 | 2001 | Kirby, James B | Exposure, resistance, and recovery: a three-dimensional framework for the study of mortality from infectious disease | Irrelevant results |
| 1710 | 2013 | Bhutta, Zulfiqar A; Das, Jai K; Rizvi, Arjumand; Gaffey, Michelle F; Walker, Neff; Horton, Susan; Webb, Patrick; Lartey, Anna; Black, Robert E | Evidence-based interventions for improvement of maternal and child nutrition: what can be done and at what cost? | Irrelevant results |
| 1711 | 2006 | Ananth, Cande V.; Oyelese, Yinka; Prasad, Vinay; Getahun, Darios; Smulian, John C. | Evidence of placental abruption as a chronic process: Associations with vaginal bleeding early in pregnancy and placental lesions | Irrelevant results |
| 1712 | 2016 | MacDonald, Evelyn Jane; Geller, Stacie E.; Lawton, Beverley | Establishment of a national severe maternal morbidity preventability review in New Zealand | Excluded for out of context |
| 1713 | 2007 | Renaudin, P.; Prual, A.; Vangeenderhuysen, C.; Ould Abdelkader, M.; Ould Mohamed Vall, M.; Ould El Joud, D. | Ensuring financial access to emergency obstetric care: Three years of experience with Obstetric Risk Insurance in Nouakchott, Mauritania | Irrelevant results |
| 1714 | 2010 | Kirkwood, Betty R; Hurt, Lisa; Amenga-Etego, Seeba; Tawiah, Charlotte; Zandoh, Charles; Danso, Samuel; Hurt, Chris; Edmond, Karen; Hill, Zelee; ten Asbroek, Guus; Fenty, Justin; Owusu-Agyei, Seth; Campbell, Oona; Arthur, Paul | Effect of vitamin A supplementation in women of reproductive age on maternal survival in Ghana (ObaapaVitA): a cluster-randomised, placebo-controlled trial | Irrelevant results |
| 1715 | 2012 | Seal, Subrata Lall; Ghosh, Debdutta; Kamilya, Gourisankar; Mukherji, Joydev; Hazra, Avijit; Garain, Pratima | Does route of delivery affect maternal and perinatal outcome in women with eclampsia? A randomized controlled pilot study | Excluded for out of context |
| 1716 | 2016 | Suplee, Patricia D.; Kleppel, Lisa; Bingham, Debra | Discharge Education on Maternal Morbidity and Mortality Provided by Nurses to Women in the Postpartum Period | Irrelevant results |
| 1717 | 2014 | Magee, Laura A.; Pels, Anouk; Helewa, Michael; Rey, Evelyne; von Dadelszen, Peter | Diagnosis, evaluation, and management of the hypertensive disorders of pregnancy | Irrelevant results |
| 1718 | 2010 | Ramachandran, Ambady; Wan Ma, Ronald Ching; Snehalatha, Chamukuttan | Diabetes in Asia | Irrelevant results |
| 1719 | 2015 | Behling, Diana J.; Renaud, Michelle | Development of an Obstetric Vital Sign Alert to Improve Outcomes in Acute Care Obstetrics | Irrelevant results |
| 1720 | 2022 | Teshome, Hana Nigussie; Ayele, Esubalew Tesfahun; Hailemeskel, Solomon; Yimer, Osman; Mulu, Getaneh Baye; Tadese, Mesfin | Determinants of maternal near-miss among women admitted to public hospitals in North Shewa Zone, Ethiopia: A case-control study | Included |
| 1721 | 2022 | Fuxe, Vendela; Brismar Wendel, Sophia; Bohm-Starke, Nina; Mühlrad, Hanna | Delivery mode and severe maternal and neonatal morbidity among singleton term breech births: A population-based cohort study | Irrelevant results |
| 1722 | 2002 | Lumbiganon, Pisake; Villar, José; Piaggio, Gilda; Metin Gülmezoglu, A; Adetoro, Lekan; Carroli, Guillermo | Side effects of oral misoprostol during the first 24 hours after administration in the third stage of labour | Irrelevant results |
| 1723 | 2018 | Sandall, Jane; Tribe, Rachel M; Avery, Lisa; Mola, Glen; Visser, Gerard HA; Homer, Caroline SE; Gibbons, Deena; Kelly, Niamh M; Kennedy, Holly Powell; Kidanto, Hussein; Taylor, Paul; Temmerman, Marleen | Short-term and long-term effects of caesarean section on the health of women and children | Irrelevant results |
| 1724 | 2002 | Jenkins, Sheri M.; Head, Barbara B.; Hauth, John C. | Severe preeclampsia at <25 weeks of gestation: Maternal and neonatal outcomes | Irrelevant results |
| 1725 | 2016 | Kilpatrick, Sarah K.; Ecker, Jeffrey L. | Severe maternal morbidity: screening and review | Irrelevant results |
| 1726 | 2021 | Blanc, Julie; Rességuier, Noémie; Loundou, Anderson; Boyer, Laurent; Auquier, Pascal; Tosello, Barthélémy; d’Ercole, Claude | Severe maternal morbidity in preterm cesarean delivery: A systematic review and meta-analysis | Excluded for out of context |
| 1727 | 2022 | Gulersen, Moti; Rochelson, Burton; Shan, Weiwei; Wetcher, Cara S.; Nimaroff, Michael; Blitz, Matthew J. | Severe maternal morbidity in pregnant patients with SARS-CoV-2 infection | Excluded for out of context |
| 1728 | 2016 | Kilpatrick, Sarah J.; Abreo, Anisha; Greene, Naomi; Melsop, Kathryn; Peterson, Nancy; Shields, Larry E.; Main, Elliot K. | Severe maternal morbidity in a large cohort of women with acute severe intrapartum hypertension | Excluded for out of context |
| 1729 | 2012 | Santana, Danielly S.; Cecatti, José G.; Parpinelli, Mary A.; Haddad, Samira M.; Costa, Maria L.; Sousa, Maria H.; Souza, João P.; Camargo, Rodrigo S.; Pacagnella, Rodolfo C.; Surita, Fernanda G.; Pinto e Silva, João L. | Severe maternal morbidity due to abortion prospectively identified in a surveillance network in Brazil | Excluded for out of context |
| 1730 | 2015 | Rocha Filho, Edilberto A.; Costa, Maria L.; Cecatti, Jose G.; Parpinelli, Mary A.; Haddad, Samira M.; Pacagnella, Rodolfo C.; Sousa, Maria H.; Melo, Elias F.; Surita, Fernanda G.; Souza, Joao P. | Severe maternal morbidity and near miss due to postpartum hemorrhage in a national multicenter surveillance study | Excluded for out of context |
| 1731 | 2022 | Interrante, Julia D.; Tuttle, Mariana S.; Admon, Lindsay K.; Kozhimannil, Katy B. | Severe Maternal Morbidity and Mortality Risk at the Intersection of Rurality, Race and Ethnicity, and Medicaid | Irrelevant results |
| 1732 | 2021 | Platner, Marissa H.; Ackerman, Christina M.; Howland, Renata E.; Illuzzi, Jessica; Reddy, Uma M.; Bourjeily, Ghada; Xu, Xiao; Lipkind, Heather S. | Severe maternal morbidity and mortality during delivery hospitalization of class I, II, III, and super obese women | Excluded for out of context |
| 1733 | 2011 | Camargo, Rodrigo S.; Santana, Danielly S.; Cecatti, José G.; Pacagnella, Rodolfo C.; Tedesco, Ricardo P.; Melo, Elias F.; Sousa, Maria H. | Severe maternal morbidity and factors associated with the occurrence of abortion in Brazil | Irrelevant results |
| 1734 | 2017 | Hehir, Mark P.; Ananth, Cande V.; Wright, Jason D.; Siddiq, Zainab; D'Alton, Mary E.; Friedman, Alexander M. | Severe maternal morbidity and comorbid risk in hospitals performing <1000 deliveries per year | Excluded for out of context |
| 1735 | 2021 | Panelli, Danielle M.; Leonard, Stephanie A.; Joudi, Noor; Girsen, Anna I.; Judy, Amy E.; El-Sayed, Yasser Y.; Gilbert, William M.; Lyell, Deirdre J. | Severe maternal and neonatal morbidity after attempted operative vaginal delivery | Excluded for out of context |
| 1736 | 2019 | Perry, Allison K.; Rossi, Robert M.; DeFranco, Emily A. | Severe adverse maternal outcomes associated with chorioamnionitis | Excluded for out of context |
| 1737 | 2016 | Witteveen, Tom; Van Den Akker, Thomas; Zwart, Joost J.; Bloemenkamp, Kitty W.; Van Roosmalen, Jos | Severe acute maternal morbidity in multiple pregnancies: a nationwide cohort study | Excluded for out of context |
| 1738 | 2009 | Ronsmans, Carine | Severe acute maternal morbidity in low-income countries | Irrelevant results |
| 1739 | 2009 | van Roosmalen, Jos; Zwart, Joost | Severe acute maternal morbidity in high-income countries | Excluded for out of context |
| 1740 | 2012 | Lucas, D. N.; Robinson, P. N.; Nel, M. R. | Sepsis in obstetrics and the role of the anaesthetist | Irrelevant results |
| 1741 | 2021 | Ahmed, Badredeen; Konje, Justin C. | Screening for infections in pregnancy – An overview of where we are today | Irrelevant results |
| 1742 | 2019 | Luke, Barbara; Brown, Morton B.; Wantman, Ethan; Baker, Valerie L.; Doody, Kevin J.; Seifer, David B.; Spector, Logan G. | Risk of severe maternal morbidity by maternal fertility status: a US study in 8 states | Excluded for out of context |
| 1743 | 2022 | Boulet, Sheree L.; Stanhope, Kaitlyn K.; Worrell, Nikkia; Jamieson, Denise J. | Risk of recurrent severe maternal morbidity in an urban safety-net health system | Excluded for out of context |
| 1744 | 2018 | Daru, Jahnavi; Zamora, Javier; Fernández-Félix, Borja M; Vogel, Joshua; Oladapo, Olufemi T; Morisaki, Naho; Tunçalp, Özge; Torloni, Maria Regina; Mittal, Suneeta; Jayaratne, Kapila; Lumbiganon, Pisake; Togoobaatar, Ganchimeg; Thangaratinam, Shakila; Khan, Khalid S | Risk of maternal mortality in women with severe anaemia during pregnancy and post partum: a multilevel analysis | Irrelevant results |
| 1745 | 2021 | Stamilio, David M.; Beckham, A. Jenna; Boggess, Kim A.; Jelovsek, J. Eric; Venkatesh, Kartik K. | Risk factors for postpartum readmission for preeclampsia or hypertension before delivery discharge among low-risk women: a case-control study | Irrelevant results |
| 1746 | 2012 | Urquia, Marcelo L.; O'Campo, Patricia J.; Heaman, Maureen I. | Revisiting the immigrant paradox in reproductive health: The roles of duration of residence and ethnicity | Irrelevant results |
| 1747 | 2021 | Victora, Cesar G; Christian, Parul; Vidaletti, Luis Paulo; Gatica-Domínguez, Giovanna; Menon, Purnima; Black, Robert E | Revisiting maternal and child undernutrition in low-income and middle-income countries: variable progress towards an unfinished agenda | Irrelevant results |
| 1748 | 2015 | Broekhuijsen, Kim; Bernardes, Thomas; van Baaren, Gert-Jan; Tajik, Parvin; Novikova, Natalia; Thangaratinam, Shakila; Boers, Kim; Koopmans, Corine M.; and al | Relevance of individual participant data meta-analysis for studies in obstetrics: delivery versus expectant monitoring for hypertensive disorders of pregnancy | Irrelevant results |
| 1749 | 2017 | Main, Elliott K.; Cape, Valerie; Abreo, Anisha; Vasher, Julie; Woods, Amanda; Carpenter, Andrew; Gould, Jeffrey B. | Reduction of severe maternal morbidity from hemorrhage using a state perinatal quality collaborative | Irrelevant results |
| 1750 | 2020 | Main, Elliott K.; Chang, Shen-Chih; Dhurjati, Ravi; Cape, Valerie; Profit, Jochen; Gould, Jeffrey B. | Reduction in racial disparities in severe maternal morbidity from hemorrhage in a large-scale quality improvement collaborative | Excluded for out of context |
| 1751 | 2005 | Worthman, Carol M.; Kohrt, Brandon | Receding horizons of health: biocultural approaches to public health paradoxes | Irrelevant results |
| 1752 | 2007 | Humphreys, Margaret; Costanzo, Philip; Haynie, Kerry L.; Østbye, Truls; Boly, Idrissa; Belsky, Daniel; Sloan, Frank | Racial disparities in diabetes a century ago: Evidence from the pension files of US Civil War veterans | Irrelevant results |
| 1753 | 2021 | Wang, Eileen; Glazer, Kimberly B.; Sofaer, Shoshanna; Balbierz, Amy; Howell, Elizabeth A. | Racial and Ethnic Disparities in Severe Maternal Morbidity: A Qualitative Study of Women's Experiences of Peripartum Care | Excluded for out of context |
| 1754 | 2014 | Creanga, Andreea A.; Bateman, Brian T.; Kuklina, Elena V.; Callaghan, William M. | Racial and ethnic disparities in severe maternal morbidity: a multistate analysis, 2008-2010 | Irrelevant results |
| 1755 | 2022 | Hosier, Hillary; Xu, Xiao; Underwood, Katherine; Ackerman-Banks, Christina; Campbell, Katherine H.; Reddy, Uma M. | Racial and ethnic differences in severe maternal morbidity among singleton stillbirth deliveries | Excluded for out of context |
| 1756 | 2021 | Brown, Clare C.; Adams, Caroline E.; Moore, Jennifer E. | Race, Medicaid Coverage, and Equity in Maternal Morbidity | Excluded for out of context |
| 1757 | 2021 | Hamilton, Jameaka Latrice; Shumbusho, Diane; Cooper, Devin; Fletcher, Tara; Aden, James; Weir, Larissa; Keyser, Erin | Race matters: maternal morbidity in the Military Health System | Excluded for out of context |
| 1758 | 2016 | Koblinsky, Marjorie; Moyer, Cheryl A; Calvert, Clara; Campbell, James; Campbell, Oona M R; Feigl, Andrea B; and al | Quality maternity care for every woman, everywhere: a call to action | Irrelevant results |
| 1759 | 2021 | Mullan, Samantha J.; Vricella, Laura K.; Edwards, Alexandra M.; Powel, Jennifer E.; Ong, Samantha K.; Li, Xujia; Tomlinson, Tracy M. | Pulse pressure as a predictor of response to treatment for severe hypertension in pregnancy | Irrelevant results |
| 1760 | 2021 | Woolhandler, Steffie; Himmelstein, David U; Ahmed, Sameer; Bailey, Zinzi; Bassett, Mary T; Bird, Michael; and al | Public policy and health in the Trump era | Irrelevant results |
| 1761 | 2014 | Haegerich, Tamara M; Dahlberg, Linda L; Simon, Thomas R; Baldwin, Grant T; Sleet, David A; Greenspan, Arlene I; Degutis, Linda C | Prevention of injury and violence in the USA | Irrelevant results |
| 1762 | 2016 | von Dadelszen, Peter; Magee, Laura A. | Preventing deaths due to the hypertensive disorders of pregnancy | Irrelevant results |
| 1763 | 2014 | Lawton, Beverley; MacDonald, Evelyn Jane; Brown, Selina Ann; Wilson, Leona; Stanley, James; Tait, John David; Dinsdale, Richard Alan; Coles, Carolyn Lee; Geller, Stacie E. | Preventability of severe acute maternal morbidity | Excluded for out of context |
| 1764 | 2020 | Macedo, Tereza C. C.; Montagna, Erik; Trevisan, Camila M.; Zaia, Victor; de Oliveira, Renato; Barbosa, Caio P.; Laganà, Antonio Simone; Bianco, Bianca | Prevalence of preeclampsia and eclampsia in adolescent pregnancy: A systematic review and meta-analysis of 291,247 adolescents worldwide since 1969 | Irrelevant results |
| 1765 | 2021 | Zejnullahu, Vjosa A.; Ukella-Lleshi, Dardane; Zejnullahu, Valon A.; Miftari, Ermira; Govori, Valbona | Prevalence of postpartum depression at the clinic for obstetrics and gynecology in Kosovo teaching hospital: Demographic, obstetric and psychosocial risk factors | Irrelevant results |
| 1766 | 2021 | Li, Fang; Qin, Jiabi; Zhang, Senmao; Chen, Lizhang | Prevalence of hypertensive disorders in pregnancy in China: A systematic review and meta-analysis | Irrelevant results |
| 1767 | 2021 | Deng, Kui; Liang, Juan; Mu, Yi; Liu, Zheng; Wang, Yanping; Li, Mingrong; Li, Xiaohong; Dai, Li; Li, Qi; Chen, Peiran; Xie, Yanxia; Zhu, Jun; Liu, Hanmin | Preterm births in China between 2012 and 2018: an observational study of more than 9 million women | Irrelevant results |
| 1768 | 2012 | von Dadelszen, Peter; Firoz, Tabassum; Donnay, France; Gordon, Rebecca; Justus Hofmeyr, G.; Lalani, Shifana; Payne, Beth A.; Roberts, James M.; Teela, Katherine C.; Vidler, Marianne; Sawchuck, Diane; Magee, Laura A. | Preeclampsia in Low and Middle Income Countries—Health Services Lessons Learned From the PRE-EMPT (PRE-Eclampsia–Eclampsia Monitoring, Prevention & Treatment) Project | Irrelevant results |
| 1769 | 2021 | Deichen Hansen, Megan E. | Predictors of preterm birth and low birth weight: A person-centered approach | Irrelevant results |
| 1770 | 2022 | Dude, Annie M.; Schueler, Kellie; Schumm, L. Philip; Murugesan, Manoradhan; Stulberg, Debra B. | Preconception care and severe maternal morbidity in the United States | Excluded for out of context |
| 1771 | 2010 | Steegers, Eric AP; von Dadelszen, Peter; Duvekot, Johannes J; Pijnenborg, Robert | Pre-eclampsia | Irrelevant results |
| 1772 | 2017 | Hjelm, Lisa; Handa, Sudhanshu; de Hoop, Jacobus; Palermo, Tia | Poverty and perceived stress: Evidence from two unconditional cash transfer programs in Zambia | Irrelevant results |
| 1773 | 2010 | Harper, Margaret Ann | Postpartum pyrexia of unknown origin | Irrelevant results |
| 1774 | 2016 | Bingham, Debra; Scheich, Benjamin; Byfield, Renée; Wilson, Barbara; Bateman, Brian T. | Postpartum Hemorrhage Preparedness Elements Vary Among Hospitals in New Jersey and Georgia | Irrelevant results |
| 1775 | 2016 | Domingues, Rosa Maria Soares Madeira; Dias, Marcos Augusto Bastos; Schilithz, Arthur Orlando Corrêa; Leal, Maria do Carmo | Factors associated with maternal near miss in childbirth and the postpartum period: findings from the birth in Brazil National Survey, 2011–2012 | Excluded for out of context |
| 1776 | 2017 | Mekango, Dejene Ermias; Alemayehu, Mussie; Gebregergs, Gebremedhin Berhe; Medhanyie, Araya Abrha; Goba, Gelila | Determinants of maternal near miss among women in public hospital maternity wards in Northern Ethiopia: A facility based case-control study | Irrelevant results |
| 1777 | 2017 | Witteveen, Tom; Bezstarosti, Hans; de Koning, Ilona; Nelissen, Ellen; Bloemenkamp, Kitty W.; van Roosmalen, Jos; van den Akker, Thomas | Validating the WHO maternal near miss tool: comparing high- and low-resource settings | Irrelevant results |
| 1778 | 2009 | Say, Lale; Souza, João Paulo; Pattinson, Robert C. | Maternal near miss – towards a standard tool for monitoring quality of maternal health care | Irrelevant results |
| 1779 | 2019 | Worke, Mulugeta Dile; Enyew, Habtamu Demelash; Dagnew, Maru Mekie | Magnitude of maternal near misses and the role of delays in Ethiopia: a hospital based cross-sectional study | Included |
| 1780 | 2000 | Tinker, Anne | Women’s health: the unfinished agenda | Irrelevant results |
| 1781 | 2007 | Gill, Kirrin; Pande, Rohini; Malhotra, Anju | Women deliver for development | Irrelevant results |
| 1782 | 2015 | Langer, Ana; Meleis, Afaf; Knaul, Felicia M; Atun, Rifat; Aran, Meltem; Arreola-Ornelas, Héctor; and al | Women and Health: the key for sustainable development | Irrelevant results |
| 1783 | 2001 | Gülmezoglu, A Metin; Villar, José; Ngoc, Nguyen Thi Nhu; Piaggio, Gilda; Carroli, Guillermo; Adetoro, Lekan; and al | WHO multicentre randomised trial of misoprostol in the management of the third stage of labour | Irrelevant results |
| 1784 | 2019 | Bateman, Brian T. | What’s New in Obstetric Anesthesia: a focus on maternal morbidity and mortality | Irrelevant results |
| 1785 | 2015 | Litorp, Helena; Mgaya, Andrew; Kidanto, Hussein L.; Johnsdotter, Sara; Essén, Birgitta | ‘What about the mother?’ Women׳s and caregivers׳ perspectives on caesarean birth in a low-resource setting with rising caesarean section rates | Irrelevant results |
| 1786 | 2020 | Suarez, Sebastian; Conde-Agudelo, Agustin; Borovac-Pinheiro, Anderson; Suarez-Rebling, Daniela; Eckardt, Melody; Theron, Gerhard; Burke, Thomas F. | Uterine balloon tamponade for the treatment of postpartum hemorrhage: a systematic review and meta-analysis | Irrelevant results |
| 1787 | 2016 | Dossa, Nissou I.; Philibert, Aline; Dumont, Alexandre | Using routine health data and intermittent community surveys to assess the impact of maternal and neonatal health interventions in low-income countries: A systematic review | Irrelevant results |
| 1788 | 2018 | Ward-Peterson, Melissa; Fennie, Kristopher; Mauck, Daniel; Shakir, Maryam; Cosner, Chelsea; Bhoite, Prasad; Trepka, Mary Jo; Madhivanan, Purnima | Using multilevel models to evaluate the influence of contextual factors on HIV/AIDS, sexually transmitted infections, and risky sexual behavior in sub-Saharan Africa: a systematic review | Irrelevant results |
| 1789 | 2018 | Suplee, Patricia D.; Bloch, Joan Rosen; Hillier, Amy; Herbert, Tasha | Using Geographic Information Systems to Visualize Relationships Between Perinatal Outcomes and Neighborhood Characteristics When Planning Community Interventions | Irrelevant results |
| 1790 | 2011 | Mbassi, Symplice Mbola; Mbu, Robinson; Bouvier-Colle, Marie Hélène | Use of routinely collected data to assess maternal mortality in seven tertiary maternity centers in Cameroon | Irrelevant results |
| 1791 | 2016 | Shields, Laurence E.; Wiesner, Suzanne; Klein, Catherine; Pelletreau, Barbara; Hedriana, Herman L. | Use of Maternal Early Warning Trigger tool reduces maternal morbidity | Excluded for out of context |
| 1792 | 2014 | Vogel, Joshua P; Souza, João Paulo; Gülmezoglu, A Metin; Mori, Rintaro; Lumbiganon, Pisake; Qureshi, Zahida; and al | Use of antenatal corticosteroids and tocolytic drugs in preterm births in 29 countries: an analysis of the WHO Multicountry Survey on Maternal and Newborn Health | Irrelevant results |
| 1793 | 2019 | Fischer, C.; Bonnet, M. P.; Girault, A.; Le Ray, C. | Update: Focus in-hospital maternal cardiac arrest | Irrelevant results |
| 1794 | 2021 | Kapito, Esnath; Chirwa, Ellen; Maluwa, Alfred; Kachimanga, Chembe; Openshaw, Maria; Rankin, Sally; Rose, Sharon; Muller, Anna; Baltzell, Kimberly | Underreporting of maternal and neonatal complications: A comparison of information in maternity registers and client charts at a rural community hospital in Malawi | Irrelevant results |
| 1795 | 2012 | Stevens, Gretchen A; Finucane, Mariel M; Paciorek, Christopher J; Flaxman, Seth R; White, Richard A; Donner, Abigail J; Ezzati, Majid | Trends in mild, moderate, and severe stunting and underweight, and progress towards MDG 1 in 141 developing countries: a systematic analysis of population representative data | Irrelevant results |
| 1796 | 2020 | Manley, Charisma N.; Deepak, Venkataraman; Ravikumar, Nithin; Smith, Alicia K.; Knight, Anna K.; Badell, Martina L.; Sidell, Neil; Rajakumar, Augustine | Transcription factor AP2A affects sFLT1 expression and decidualization in decidual stromal cells: Implications to preeclampsia pathology | Irrelevant results |
| 1797 | 2023 | Vuncannon, Danielle M.; Platner, Marissa H.; Boulet, Sheree L. | Timely treatment of severe hypertension and risk of severe maternal morbidity at an urban hospital | Excluded for out of context |
| 1798 | 2022 | Clapp, Mark A.; James, Kaitlyn E.; McCoy, Thomas H.; Perlis, Roy H.; Kaimal, Anjali J. | The value of intrapartum factors in predicting maternal morbidity | Irrelevant results |
| 1799 | 2013 | Mackenbach, Johan P; Karanikolos, Marina; McKee, Martin | The unequal health of Europeans: successes and failures of policies | Irrelevant results |
| 1800 | 2018 | Abubakar, Ibrahim; Aldridge, Robert W; Devakumar, Delan; Orcutt, Miriam; Burns, Rachel; Barreto, Mauricio L; and al | The UCL–Lancet Commission on Migration and Health: the health of a world on the move | Irrelevant results |
| 1801 | 2008 | Eastabrook, Genevieve; Hu, Yuxiang; von Dadelszen, Peter | The Role of Decidual Natural Killer Cells in Normal Placentation and in the Pathogenesis of Preeclampsia | Irrelevant results |
| 1802 | 2021 | Mal-Sarkar, Tatini; Keyes, Katherine; Koen, Nastassja; Barnett, Whitney; Myer, Landon; Rutherford, Caroline; Zar, Heather J.; Stein, Dan J.; Lund, Crick | The relationship between childhood trauma, socioeconomic status, and maternal depression among pregnant women in a South African birth cohort study | Irrelevant results |
| 1803 | 2020 | Wilson, R. Douglas | The Real Maternal Risks in a Pregnancy: A Structured Review to Enhance Maternal Understanding and Education | Irrelevant results |
| 1804 | 2017 | Chaiworapongsa, Tinnakorn; Romero, Roberto; Erez, Offer; Tarca, Adi L.; Conde-Agudelo, Agustin; Chaemsaithong, Piya; Kim, Chong Jai; Kim, Yeon Mee; Kim, Jung-Sun; Yoon, Bo Hyun; Hassan, Sonia S.; Yeo, Lami; Korzeniewski, Steven J. | The prediction of fetal death with a simple maternal blood test at 24-28 weeks: a role for angiogenic index-1 (PlGF/sVEGFR-1 ratio) | Irrelevant results |
| 1805 | 2008 | Stratton, Leeanne; O'Neill, Marie S.; Kruk, Margaret E.; Bell, Michelle L. | The persistent problem of malaria: Addressing the fundamental causes of a global killer | Irrelevant results |
| 1806 | 2017 | Agyepong, Irene Akua; Sewankambo, Nelson; Binagwaho, Agnes; Coll-Seck, Awa Marie; Corrah, Tumani; Ezeh, Alex; and al | The path to longer and healthier lives for all Africans by 2030: the Lancet Commission on the future of health in sub-Saharan Africa | Irrelevant results |
| 1807 | 2019 | Gostin, Lawrence O; Monahan, John T; Kaldor, Jenny; DeBartolo, Mary; Friedman, Eric A; Gottschalk, Katie; and al | The legal determinants of health: harnessing the power of law for global health and sustainable development | Irrelevant results |
| 1808 | 2021 | Vogel, Birgit; Acevedo, Monica; Appelman, Yolande; Bairey Merz, C Noel; Chieffo, Alaide; Figtree, Gemma A; Guerrero, Mayra; Kunadian, Vijay; Lam, Carolyn S P; Maas, Angela H E M; Mihailidou, Anastasia S; Olszanecka, Agnieszka; Poole, Jeanne E; Saldarriaga, Clara; Saw, Jacqueline; Zühlke, Liesl; Mehran, Roxana | The Lancet women and cardiovascular disease Commission: reducing the global burden by 2030 | Irrelevant results |
| 1809 | 2020 | Bukhman, Gene; Mocumbi, Ana O; Atun, Rifat; Becker, Anne E; Bhutta, Zulfiqar; Binagwaho, Agnes; and al | The Lancet NCDI Poverty Commission: bridging a gap in universal health coverage for the poorest billion | Irrelevant results |
| 1810 | 2018 | Watts, Nick; Amann, Markus; Ayeb-Karlsson, Sonja; Belesova, Kristine; Bouley, Timothy; Boykoff, Maxwell; and al | The Lancet Countdown on health and climate change: from 25 years of inaction to a global transformation for public health | Irrelevant results |
| 1811 | 2018 | Patel, Vikram; Saxena, Shekhar; Lund, Crick; Thornicroft, Graham; Baingana, Florence; Bolton, Paul; and al | The Lancet Commission on global mental health and sustainable development | Irrelevant results |
| 1812 | 2020 | Chan, Juliana C N; Lim, Lee-Ling; Wareham, Nicholas J; Shaw, Jonathan E; Orchard, Trevor J; Zhang, Ping; and al | The Lancet Commission on diabetes: using data to transform diabetes care and patient lives | Irrelevant results |
| 1813 | 2021 | Bane, Shalmali; Carmichael, Suzan L; Snowden, Jonathan M; Liu, Can; Lyndon, Audrey; Wall-Wieler, Elizabeth | The impact of Severe Maternal Morbidity on probability of subsequent birth in a population-based study of women in California from 1997-2017 | Excluded for out of context |
| 1814 | 2012 | Ameh, Charles; Adegoke, Adetoro; Hofman, Jan; Ismail, Fouzia M.; Ahmed, Fatuma M.; van den Broek, Nynke | The impact of emergency obstetric care training in Somaliland, Somalia | Irrelevant results |
| 1815 | 2018 | Brown, Mark A.; Magee, Laura A.; Kenny, Louise C.; Karumanchi, S. Ananth; McCarthy, Fergus P; Saito, Shigeru; Hall, David R.; Warren, Charlotte E.; Adoyi, Gloria; Ishaku, Salisu | The hypertensive disorders of pregnancy: ISSHP classification, diagnosis & management recommendations for international practice | Irrelevant results |
| 1816 | 2018 | Clapp, Mark A.; James, Kaitlyn E.; Kaimal, Anjali J. | The effect of hospital acuity on severe maternal morbidity in high-risk patients | Irrelevant results |
| 1817 | 2020 | Rothstein, Jessica D.; Caulfield, Laura E.; Broaddus-Shea, Elena T.; Muschelli, John; Gilman, Robert H.; Winch, Peter J. | “The doctor said formula would help me”: Health sector influences on use of infant formula in peri-urban Lima, Peru | Irrelevant results |
| 1818 | 2004 | Geller, Stacie E.; Rosenberg, Deborah; Cox, Suzanne M.; Brown, Monique L.; Simonson, Louise; Driscoll, Catherine A.; Kilpatrick, Sarah J. | The continuum of maternal morbidity and mortality: Factors associated with severity | Excluded for out of context |
| 1819 | 2015 | Prince, Martin J; Wu, Fan; Guo, Yanfei; Gutierrez Robledo, Luis M; O'Donnell, Martin; Sullivan, Richard; Yusuf, Salim | The burden of disease in older people and implications for health policy and practice | Irrelevant results |
| 1820 | 2011 | Ganzevoort, Wessel; Sibai, Baha M. | Temporising versus interventionist management (preterm and at term) | Irrelevant results |
| 1821 | 2011 | Flenady, Vicki; Middleton, Philippa; Smith, Gordon C; Duke, Wes; Erwich, Jan Jaap; Khong, T Yee; Neilson, Jim; Ezzati, Majid; Koopmans, Laura; Ellwood, David; Fretts, Ruth; Frøen, J Frederik | Stillbirths: the way forward in high-income countries | Irrelevant results |
| 1822 | 2016 | Lawn, Joy E; Blencowe, Hannah; Waiswa, Peter; Amouzou, Agbessi; Mathers, Colin; Hogan, Dan; and al | Stillbirths: rates, risk factors, and acceleration towards 2030 | Irrelevant results |
| 1823 | 2012 | Olagbuji, Biodun N.; Ezeanochie, Michael C.; Igbaruma, Solomon; Okoigi, Samson O.; Ande, Adedapo B. | Stillbirth in cases of severe acute maternal morbidity | Irrelevant results |
| 1824 | 2017 | Ivory, Catherine H.; Freytsis, Maria; Lagrew, David C.; Magee, Dale; Vallejo, Manuel; Hasley, Steve | Standardizing Maternity Care Data to Improve Coordination of Care | Irrelevant results |
| 1825 | 2021 | Lappen, Justin R.; Pettker, Christian M.; Louis, Judette M. | Society for Maternal-Fetal Medicine Consult Series #54: Assessing the risk of maternal morbidity and mortality | Irrelevant results |
| 1826 | 2019 | McDonnell, Brendan P.; Keogan, Sheila; Clancy, Luke; Regan, Carmen | Smoking cessation support and obstetric outcomes in an Irish maternity hospital | Irrelevant results |
| 1827 | 2018 | Jain, Joses A.; Temming, Lorene A.; D’Alton, Mary E.; Gyamfi-Bannerman, Cynthia; Tuuli, Methodius; Louis, Judette M.; and al | SMFM Special Report: Putting the “M” back in MFM: Reducing racial and ethnic disparities in maternal morbidity and mortality: A call to action | Irrelevant results |
| 1828 | 2016 | Howell, Elizabeth A.; Egorova, Natalia N.; Balbierz, Amy; Zeitlin, Jennifer; Hebert, Paul L. | Site of delivery contribution to black-white severe maternal morbidity disparity | Excluded for out of context |
| 1829 | 2015 | Todd, Catherine S.; Mansoor, G. Farooq; Haider, Sadia; Hashimy, Pashtoon; Mustafavi, Nazifa; Nasir, Abdul; Miller, Suellen | A Case-Control Study of Correlates of Severe Acute Maternal Morbidity in Kabul, Afghanistan | Excluded for out of context |
| 1830 | 2015 | Águila, Sonia | Zero in on postpartum hemorrhage to reduce Cuba's maternal mortality | Irrelevant results |
| 1831 | 2016 | Fagundez, Gabriela; Perez-Freixo, Hugo; Eyene, Juan; Momo, Juan Carlos; Biyé, Lucia; Esono, Teodoro; Ondó Mba Ayecab, Marcial; Benito, Agustín; Aparicio, Pilar; Herrador, Zaida | Treatment Adherence of Tuberculosis Patients Attending Two Reference Units in Equatorial Guinea | Irrelevant results |
| 1832 | 2018 | Daw, Mohamed A.; Buktir Ali, Lutfi A.; Daw, Amina M.; Sifennasr, Nadia E. M.; Dau, Aghnyia A.; Agnan, Mohamed M.; El-Bouzedi, Abdallah; In association with the Libyan Study Group of Hepatitis & HIV | The geographic variation and spatiotemporal distribution of hepatitis C virus infection in Libya: 2007-2016 | Irrelevant results |
| 1833 | 2003 | Cochet, L.; Pattinson, R.C.; Macdonald, A.P. | Severe acute maternal morbidity and maternal death audit - A rapid diagnostic tool for evaluating maternal care | Irrelevant results |
| 1834 | 2008 | Wandabwa, J.; Doyle, P.; Todd, J.; Kiondo, P.; Wandabwa, M. A.; Aziga, F. | Risk factors for ruptured uterus in Mulago hospital Kampala, Uganda | Irrelevant results |
| 1835 | 2018 | Iwuh, I.A.; Fawcus, S.; Schoeman, L. | Maternal near-miss audit in the metro west maternity service, Cape Town, South Africa: A retrospective observational study | Irrelevant results |
| 1836 | 2019 | Okonofua, F. | Maternal near-miss morbidity: is this evidence of maternal health quality in sub-Saharan Africa? | Irrelevant results |
| 1837 | 2017 | Schutte, A. E.; Botha, S.; Fourie, C. M. T.; Gafane-Matemane, L. F.; Kruger, R.; Lammertyn, L.; Malan, L.; Mels, C. M. C.; Schutte, R.; Smith, W.; van Rooyen, J. M.; Ware, L. J.; Huisman, H. W. | Recent advances in understanding hypertension development in sub-Saharan Africa | Irrelevant results |
| 1838 | 2017 | Sarfo, Fred Stephen; Kyem, Gloria; Ovbiagele, Bruce; Akassi, John; Sarfo-Kantanka, Osei; Agyei, Martin; Badu, Elizabeth; Adusei Mensah, Nathaniel | One-Year Rates and Determinants of Poststroke Systolic Blood Pressure Control among Ghanaians | Irrelevant results |
| 1839 | 2021 | Minehart, Rebecca D.; Bryant, Allison S.; Jackson, Jaleesa; Daly, Jaime L. | Racial/Ethnic Inequities in Pregnancy-Related Morbidity and Mortality | Excluded for out of context |
| 1840 | 2017 | Maswime, S.; Buchmann, E. J. | Why women bleed and how they are saved: a cross-sectional study of caesarean section near-miss morbidity | Irrelevant results |
| 1841 | 2020 | Verschueren, Kim J. C.; Paidin, Rubinah R.; Broekhuis, Annabel; Ramkhelawan, Olivier S. S.; Kodan, Lachmi R.; Kanhai, Humphrey H. H.; Browne, Joyce L.; Bloemenkamp, Kitty W. M.; Rijken, Marcus J. | Why magnesium sulfate ‘coverage’ only is not enough to reduce eclampsia: Lessons learned in a middle-income country | Irrelevant results |
| 1842 | 2020 | Weiniger, C. F. | What's new in obstetric anesthesia in 2018? | Irrelevant results |
| 1843 | 2016 | Sia, Drissa; Onadja, Yentéma; Hajizadeh, Mohammad; Heymann, S. Jody; Brewer, Timothy F.; Nandi, Arijit | What explains gender inequalities in HIV/AIDS prevalence in sub-Saharan Africa? Evidence from the demographic and health surveys | Irrelevant results |
| 1844 | 2012 | Assefa, Nega; Berhane, Yemane; Worku, Alemayehu | Wealth status, mid upper arm circumference (MUAC) and antenatal care (ANC) are determinants for low birth weight in Kersa, Ethiopia | Irrelevant results |
| 1845 | 2008 | Awusabo-Asare, Kofi; Annim, Samuel K. | Wealth status and risky sexual behaviour in Ghana and Kenya | Irrelevant results |
| 1846 | 2000 | Villamor, E.; Mbise, R.; Spiegelman, D.; Ndossi, G.; Fawzi, W. W. | Vitamin A supplementation and other predictors of anemia among children from Dar Es Salaam, Tanzania | Irrelevant results |
| 1847 | 2017 | Mbengue, Mouhamed Abdou Salam; Mboup, Aminata; Ly, Indou Deme; Faye, Adama; Camara, Fatou Bintou Niang; Thiam, Moussa; Ndiaye, Birahim Pierre; Dieye, Tandakha Ndiaye; Mboup, Souleymane | Vaccination coverage and immunization timeliness among children aged 12-23 months in Senegal: a Kaplan-Meier and Cox regression analysis approach | Irrelevant results |
| 1848 | 2010 | Vesel, Linda; Bahl, Rajiv; Martines, Jose; Penny, Mary; Bhandari, Nita; Kirkwood, Betty R.; WHO Immunization-linked Vitamin A Supplementation Study Group | Use of new World Health Organization child growth standards to assess how infant malnutrition relates to breastfeeding and mortality | Irrelevant results |
| 1849 | 2009 | Olusanya, B. O.; Ebuehi, O. M.; Somefun, A. O. | Universal infant hearing screening programme in a community with predominant non-hospital births: a three-year experience | Irrelevant results |
| 1850 | 2018 | Njagi, Purity; Arsenijevic, Jelena; Groot, Wim | Understanding variations in catastrophic health expenditure, its underlying determinants and impoverishment in Sub-Saharan African countries: a scoping review | Irrelevant results |
| 1851 | 2014 | Tunçalp, Ö.; Hindin, M.J.; Adu-Bonsaffoh, K.; Adanu, R.M. | Understanding the Continuum of Maternal Morbidity in Accra, Ghana | Irrelevant results |
| 1852 | 2019 | Takele, Kasahun; Zewotir, Temesgen; Ndanguza, Denis | Understanding correlates of child stunting in Ethiopia using generalized linear mixed models | Irrelevant results |
| 1853 | 2018 | Yaya, Sanni; Bishwajit, Ghose; Okonofua, Friday; Uthman, Olalekan A. | Under five mortality patterns and associated maternal risk factors in sub-Saharan Africa: A multi-country analysis | Irrelevant results |
| 1854 | 2003 | Adams-Guppy, Julie; Guppy, Andrew | Truck driver fatigue risk assessment and management: a multinational survey | Irrelevant results |
| 1855 | 2019 | Mukora-Mutseyekwa, Fadzai; Zeeb, Hajo; Nengomasha, Lydia; Kofi Adjei, Nicholas | Trends in Prevalence and Related Risk Factors of Overweight and Obesity among Women of Reproductive Age in Zimbabwe, 2005-2015 | Irrelevant results |
| 1856 | 2013 | Mane, Maram; Fisker, Ane B.; Ravn, Henrik; Aaby, Peter; Rodrigues, Amabelia | Trends and determinants of mortality in women of reproductive age in rural Guinea-Bissau, West Africa--a cohort study | Irrelevant results |
| 1857 | 2000 | Lutalo, T.; Kidugavu, M.; Wawer, M. J.; Serwadda, D.; Zabin, L. S.; Gray, R. H. | Trends and determinants of contraceptive use in Rakai District, Uganda, 1995-98 | Irrelevant results |
| 1858 | 2011 | Souza, João Paulo; Gülmezoglu, Ahmet Metin; Carroli, Guillermo; Lumbiganon, Pisake; Qureshi, Zahida; WHOMCS Research Group | The world health organization multicountry survey on maternal and newborn health: study protocol | Irrelevant results |
| 1859 | 2013 | Knight, Marian; Lindquist, Anthea | The UK Obstetric Surveillance System: Impact on patient safety | Irrelevant results |
| 1860 | 2008 | Harling, Guy; Ehrlich, Rodney; Myer, Landon | The social epidemiology of tuberculosis in South Africa: a multilevel analysis | Irrelevant results |
| 1861 | 2020 | Charles, Charles M'poca; Amoah, Emefa Modey; Kourouma, Kadidiatou Raissa; Bahamondes, Luis Guilhermo; Cecatti, José Guilherme; Osman, Nafissa Bique; Govule, Philip; Diallo, Abdou Karim; Sacarlal, Jahit; Pacagnella, Rodolfo de Carvalho | The SARS-CoV-2 pandemic scenario in Africa: What should be done to address the needs of pregnant women? | Irrelevant results |
| 1862 | 2014 | Hassanein, Ibrahim M. A.; Fathalla, Mohamed M. F.; Abdel Rahim, Taher | The role of newborn gender in postpartum depressive symptoms among women in Upper Egypt | Irrelevant results |
| 1863 | 2012 | Pacagnella, Rodolfo Carvalho; Cecatti, Jose Guilherme; Osis, Maria Jose; Souza, João Paulo | The role of delays in severe maternal morbidity and mortality: expanding the conceptual framework | Irrelevant results |
| 1864 | 2021 | Stanhope, Kaitlyn K; Adeyemi, Deborah I; Li, Tanya; Johnson, Tatyana; Boulet, Sheree L | The relationship between the neighborhood built and social environment and hypertensive disorders of pregnancy: A scoping review | Irrelevant results |
| 1865 | 2020 | Dorsamy, Vinogrin; Bagwandeen, Chauntelle; Moodley, Jagadesa | The prevalence, risk factors and outcomes of anaemia in South African pregnant women: a protocol for a systematic review and meta-analysis | Irrelevant results |
[truncated: 51,290 more chars]
