# Supplementary material for: Socioeconomic, demographic and obstetric determinants of maternal near miss in Africa: A systematic review
Source: PLoS One. 2025 Feb 12;20(2):e0313897. doi: 10.1371/journal.pone.0313897 (PMC11819575; doi:10.1371/journal.pone.0313897)
Supplement: S6 Table — (DOCX) [file pone.0313897.s009.docx]

**Tables S7 :** **Name of data extractors and date of data extraction, as well as Confirmation that the study was eligible for inclusion**

| **Publication Year** | **Author** | **Title** | **Name of data extractors** | **Date Added** | **study was eligible for inclusion** | **Library Catalog** |
| --- | --- | --- | --- | --- | --- | --- |
| 2020 | Tura, Abera Kenay; Scherjon, Sicco; Stekelenburg, Jelle; van Roosmalen, Jos; van den Akker, Thomas; Zwart, Joost | Severe Hypertensive Disorders of Pregnancy in Eastern Ethiopia: Comparing the Original WHO and Adapted sub-Saharan African Maternal Near-Miss Criteria | Mory DIAKITE and Assarag, Bouchra | 21/03/2024 20:37 | Yes | PubMed |
| 2004 | Gandhi, M.N.; Welz, T.; Ronsmans, C. | Severe acute maternal morbidity in rural South Africa | Mory DIAKITE and Assarag, Bouchra | 21/03/2024 20:37 | Yes | Scopus |
| 2012 | Briand, Valérie; Dumont, Alexandre; Abrahamowicz, Michal; Sow, Amadou; Traore, Mamadou; Rozenberg, Patrick; Watier, Laurence; Fournier, Pierre | Maternal and perinatal outcomes by mode of delivery in senegal and mali: a cross-sectional epidemiological survey | Mory DIAKITE and Assarag, Bouchra | 21/03/2024 20:32 | Yes | PubMed |
| 2017 | Kalisa, R.; Rulisa, S.; van Roosmalen, J.; van den Akker, T. | Maternal and perinatal outcome after previous caesarean section in rural Rwanda | Mory DIAKITE and Assarag, Bouchra | 21/03/2024 20:32 | Yes | Scopus |
| 2016 | Nansubuga, Elizabeth; Ayiga, Natal; Moyer, Cheryl A. | Prevalence of maternal near miss and community-based risk factors in Central Uganda | Mory DIAKITE and Assarag, Bouchra | 21/03/2024 20:29 | Yes | ScienceDirect |
| 2018 | Kasahun, A.W.; Wako, W.G. | Predictors of maternal near miss among women admitted in Gurage zone hospitals, South Ethiopia, 2017: A case control study | Mory DIAKITE and Assarag, Bouchra | 21/03/2024 20:23 | Yes | Scopus |
| 2021 | Geze Tenaw, Shegaw; Girma Fage, Sagni; Assefa, Nega; Kenay Tura, Abera | Determinants of maternal near-miss in private hospitals in eastern Ethiopia: A nested case-control study | Mory DIAKITE and Assarag, Bouchra | 12/03/2022 01:16 | Yes | SAGE Journals |
| 2021 | Tolesa, Dereje | Prevalence and Associated Factors with Maternal Near-Miss among Pregnant Women at Hawassa University Comprehensive Specialized Hospital, Sidama Region, Ethiopia | Mory DIAKITE and Assarag, Bouchra | 12/03/2022 00:18 | Yes | clinmedjournals.org |
| 2013 | Adeoye, Ikeola A.; Onayade, Adedeji A.; Fatusi, Adesegun O. | Incidence, determinants and perinatal outcomes of near miss maternal morbidity in Ile-Ife Nigeria: a prospective case control study | Mory DIAKITE and Assarag, Bouchra | 10/12/2021 09:42 | Yes | PubMed |
| 2015 | Assarag, Bouchra; Dujardin, Bruno; Delamou, Alexandre; Meski, Fatima-Zahra; De Brouwere, Vincent | Determinants of maternal near-miss in Morocco: too late, too far, too sloppy? | Mory DIAKITE and Assarag, Bouchra | 08/12/2021 12:04 | Yes | PubMed |
| 2020 | Dessalegn, Fikadu Nugusu; Astawesegn, Feleke Hailemichael; Hankalo, Nana Chea | Factors Associated with Maternal Near Miss among Women Admitted in West Arsi Zone Public Hospitals, Ethiopia: Unmatched Case-Control Study | Mory DIAKITE and Assarag, Bouchra | 20/05/2022 15:23 | Yes | PubMed |
| 2020 | Kumela, Lemi; Tilahun, Temesgen; Kifle, Demeke | Determinants of maternal near miss in Western Ethiopia | Mory DIAKITE and Assarag, Bouchra | 20/05/2022 15:23 | Yes | PubMed |
| 2022 | Teshome, Hana Nigussie; Ayele, Esubalew Tesfahun; Hailemeskel, Solomon; Yimer, Osman; Mulu, Getaneh Baye; Tadese, Mesfin | Determinants of maternal near-miss among women admitted to public hospitals in North Shewa Zone, Ethiopia: A case-control study | Mory DIAKITE and Assarag, Bouchra | 17/12/2022 10:44 | Yes | PubMed Central |
| 2019 | Worke, Mulugeta Dile; Enyew, Habtamu Demelash; Dagnew, Maru Mekie | Magnitude of maternal near misses and the role of delays in Ethiopia: a hospital based cross-sectional study | Mory DIAKITE and Assarag, Bouchra | 10/01/2023 21:01 | Yes | BioMed Central |
| 2020 | Turi, Ebisa; Fekadu, Ginenus; Taye, Bedasa; Kejela, Gemechu; Desalegn, Markos; Mosisa, Getu; Etafa, Worku; Tsegaye, Reta; Simegnew, Dawit; Tilahun, Temesgen | The impact of antenatal care on maternal near-miss events in Ethiopia: A systematic review and meta-analysis | Mory DIAKITE and Assarag, Bouchra | 21/03/2024 20:46 | Yes | ScienceDirect |
| 2010 | Storeng, Katerini Tagmatarchi; Murray, Susan F.; Akoum, Mélanie S.; Ouattara, Fatoumata; Filippi, Véronique | Beyond body counts: a qualitative study of lives and loss in Burkina Faso after 'near-miss' obstetric complications | Mory DIAKITE and Assarag, Bouchra | 21/03/2024 19:43 | Yes | PubMed |
| 2012 | Lori, Jody R.; Starke, Amy E. | A critical analysis of maternal morbidity and mortality in Liberia, West Africa | Mory DIAKITE and Assarag, Bouchra | 21/03/2024 19:38 | Yes | ScienceDirect |
| 2021 | Mengist, Belayneh; Desta, Melaku; Tura, Abera Kenney; Habtewold, Tesfa Dejenie; Abajobir, Amanuel | Maternal near miss in Ethiopia: Protective role of antenatal care and disparity in socioeconomic inequities: A systematic review and meta-analysis | Mory DIAKITE and Assarag, Bouchra | 21/03/2024 20:13 | Yes | ScienceDirect |
| 2020 | Heitkamp, Anke; Aronson, Simcha Lot; van den Akker, Thomas; Vollmer, Linda; Gebhardt, Stefan; van Roosmalen, Jos; de Vries, Johanna I.; Theron, Gerhard | Major obstetric haemorrhage in Metro East, Cape Town, South Africa: a population-based cohort study using the maternal near-miss approach | Mory DIAKITE and Assarag, Bouchra | 21/03/2024 20:05 | Yes | PubMed |
| 2020 | Yemane, Yayehyirad; Tiruneh, Firew | Incidence-Proportion of Maternal Near-Misses and Associated Factors in Southwest Ethiopia: A Prospective Cross-Sectional Study | Mory DIAKITE and Assarag, Bouchra | 21/03/2024 20:03 | Yes | www.ncbi.nlm.nih.gov |
| 2022 | Heitkamp, Anke; Vollmer Murray, Linda; van den Akker, Thomas; Gebhardt, Gabriel S.; Sandberg, Evelien M.; van Roosmalen, Jos; Ter Wee, Marieke M.; de Vries, Johanna I.; Theron, Gerhard | Great saves or near misses? Severe maternal outcome in Metro East, South Africa: A region-wide population-based case-control study | Mory DIAKITE and Assarag, Bouchra | 21/03/2024 20:01 | Yes | PubMed |
| 2018 | Liyew, Ewnetu Firdawek; Yalew, Alemayehu Worku; Afework, Mesganaw Fantahun; Essén, Birgitta | Distant and proximate factors associated with maternal near-miss: a nested case-control study in selected public hospitals of Addis Ababa, Ethiopia | Mory DIAKITE and Assarag, Bouchra | 21/03/2024 19:50 | Yes | PubMed |
| 2021 | Habte, Aklilu; Wondimu, Merertu | Determinants of maternal near miss among women admitted to maternity wards of tertiary hospitals in Southern Ethiopia, 2020: A hospital-based case-control study | Mory DIAKITE and Assarag, Bouchra | 21/03/2024 19:49 | Yes | PLoS Journals |
| 2020 | Tariku, Mequanent | Magnitude of Severe Acute Maternal Morbidity and Associated Factors Related to Abortion: A Cross-Sectional Study in Hawassa University Comprehensive Specialized Hospital, Ethiopia, 2019 | Mory DIAKITE and Assarag, Bouchra | 08/01/2023 09:13 | Yes | PubMed Central |
| 2019 | Oppong, Sa; Bakari, A; Bell, Aj; Bockarie, Y; Adu, Ja; Turpin, Ca; Obed, Sa; Adanu, Rm; Moyer, Ca | Incidence, causes and correlates of maternal near-miss morbidity: a multi-centre cross-sectional study | Mory DIAKITE and Assarag, Bouchra | 08/01/2023 09:15 | Yes | Wiley Online Library |

** The data extracted from the studies are presented in Table 1
