# Supplementary material for: Socioeconomic, demographic and obstetric determinants of maternal near miss in Africa: A systematic review
Source: PLoS One. 2025 Feb 12;20(2):e0313897. doi: 10.1371/journal.pone.0313897 (PMC11819575; doi:10.1371/journal.pone.0313897)
Supplement: Attachment — Submitted filename: Responses to reviewers.docx [file pone.0313897.s010.docx]

Responses to reviewers

Dear Editor,

Please find enclosed the revised version of our manuscript. We would like to thank the reviewers for their constructive comments, which in our view helped us to improve the manuscript.

Please find below our responses to each of the comments.

2. Thank you for providing the following financial information:

"The funders had no role in study design, data collection and analysis, decision to publish, or manuscript preparation."

**Question a:**

Please specify the sources of funding (financial or material support) for your study. Provide a list of grants or organizations that supported your study, including funding received from your institution.

**Answer a:**

I hereby confirm that this study has received no financial support from any funder or institution.

**Question b:**

Indicate the role played by funders in the study. If funders had no role in your study, please state: "The funders had no role in study design, data collection and analysis, decision to publish, or manuscript preparation."

**Answer b:**

No funders played a role in study design, data collection and analysis, decision to publish, or manuscript preparation.

**Question c:**

If any authors received salary support from any of your funders, please indicate which authors and which funders.

**Answer c:**

None of the authors received salary support from any funder for this study.

**Question d:**

If you did not receive any funding for this study, please state: "The authors received no specific funding for this work."

**Answer d:**

The authors received no specific funding for this work.

**3-When you completed the data availability statement in the submission form, you indicated that you would make your data available upon acceptance. We strongly recommend all authors to decide on a data sharing plan prior to acceptance, as the process can be lengthy and delay publication timelines. Please note that while access restrictions are currently acceptable, all of your data must be freely accessible if your manuscript is accepted for publication. This policy applies to all data unless public deposition would violate the protocol approved by your research ethics committee. If you are unable to comply with our open data policy, please revise your statement to explain your reasoning, and we will seek the editor's advice on an exemption. Rest assured, once you have provided your revised statement, the assessment of your exemption will not delay the peer review process**

**Answer 3:**

As mentioned in my submission form, all data are accessible and have been provided during the process. However, if additional data are needed, please specify the nature of the required data.

**4- Please modify the abstract in the online submission form (via Edit Submission) or the abstract in the manuscript so that they are identical.**

**Answer 4:**

We have adjusted the abstract in the submission form to match exactly the content of the manuscript.

**For question 3, "Have the authors made all underlying data supporting the results in their manuscript fully available?" Critic #1: No**

We have made all available data accessible. However, if additional data are requested, we would like to know which ones so that we can provide them5.

1- **The research question is well formulated, but the external validity (****generalization) may be limited because the phrase "in Africa" is not well captured by the review, as** **most articles or studies in the review are limited to a few countries or specific regions in Africa. The author may need to modify the title or include other studies from different regions of Africa.**

Thank you for this comment. We mentioned Africa in the title, because in this systematic review the search of articles was done for all African countries and we included all articles available and published from any African country. We agree with the reviewer, the generalization of our findings should be interpreted with caution.

To clarify that, we added the following sentence in the limitation section:

“In this systematic review, most articles are limited to a few countries or specific regions in Africa, the generalization of our findings should be interpreted with caution.”

2- **As expected, the authors utilized the PRISMA statement approach, but no flowchart was included in the review. A** **PRISMA flowchart clearly and transparently describing the sequence of events should be included in the review.**

The PRISMA flowchart has been included in this revised version of the manuscript.

3- **The PECO approach was used in designing the research question; the author should include a statement regarding the rationale for choosing this approach over other approaches such as PICO or SPIDER, especially considering that the review involved mixed methods of design.**

Thank you for your comment. Our decision to utilize the PECO framework was guided by its alignment with the nature of our exposure-focused study. In this systematic review, we were primarily concerned with the "exposure" of various socioeconomic, demographic, and obstetric factors, rather than specific "interventions". The PECO framework is advantageous in such contexts as it facilitates an examination of how these exposures influence outcomes in our population.

4- **The synthesis of the review is not explicitly clear, and the conceptual framework based on the adapted framework by Rosa Maria Soares, Madeira Domingue et al. used for the synthesis is not included in the review.**

We added the conceptual framework as supplementary materiel in this revised version of the manuscript.

**NB:** We were unable to include this study because the results of severe maternal morbidity and maternal mortality were not differentiated, whereas we were specifically seeking factors associated with severe maternal morbidity (https://pubmed.ncbi.nlm.nih.gov/25974281/).
