# Supplementary material for: Socioeconomic, demographic and obstetric determinants of maternal near miss in Africa: A systematic review
Source: PLoS One. 2025 Feb 12;20(2):e0313897. doi: 10.1371/journal.pone.0313897 (PMC11819575; doi:10.1371/journal.pone.0313897)
Supplement: Attachment — Submitted filename: PLOS ONE REVISED MANUSCRIPT PONE-D-23-17858R1.doc [file pone.0313897.s011.doc]

RE- PLOS ONE PONE-D-23-17858R1

Dear Editor,

Thank you for sending the revised version of this manuscript to me.

I have gone through this version and observed that the Authors have made the necessary corrections and included the missing items as recommended in the earlier review.

I hereby recommend that the manuscript be accepted for publication in this present form if it meets other publication criteria.

Thank you.

AAA.
