# Supplementary material for: Socioeconomic, demographic and obstetric determinants of maternal near miss in Africa: A systematic review
Source: PLoS One. 2025 Feb 12;20(2):e0313897. doi: 10.1371/journal.pone.0313897 (PMC11819575; doi:10.1371/journal.pone.0313897)
Supplement: Attachment — Submitted filename: Response to reviewers.docx [file pone.0313897.s012.docx]

**Responses to Reviewers** :

The following responses are for the Reviewer #3, the only one who made comments to our revised manuscript.

6. Review Comments to the Authors

**Question a: “Methods:** only descriptive or a metanalysis was considered?”

**Answer to a:** Not only were descriptive studies and meta-analyses considered, but all studies that examined the factors associated with maternal near miss were also included.

**Question b: “Methods:** Why wasn´t the review registered?”

**Answer to b:** Initially, we conducted a scoping review, and at that time, it was not yet possible to register this type of review on the PROSPERO platform. After the first submission of the article, we were advised to transform it into a systematic review and apply the PRISMA guidelines for writing systematic reviews. Unfortunately, it was no longer possible to register it.

**Question c: “**Is reference 17 the best for the definition of MNM, consider the WHO definition-2009 (Pattinson R, Say L, Souza JP, Broek Nv, Rooney C; WHO Working Group on Maternal Mortality and Morbidity Classifications. WHO maternal death and near-miss classifications. Bull World Health Organ. 2009 Oct;87(10):734. doi: 10.2471/blt.09.071001. PMID: 19876533; PMCID: PMC2755324.) »

**Answer to c:** We agree that the WHO definition provided by Pattinson et al. 2009 is excellent but its source is “Say L, Souza JP, Pattinson RC. Maternal near miss – towards a standard tool for monitoring quality of maternal health care. *Best Pract Res Clin Obstet Gynaecol.* 2009;23:287–96. doi: 10.1016/j.bpobgyn.2009.01.007 ». However, we followed your suggestion and cited Pattinson et al. 2009.

**Question d:** “Flowchart: Please include abbreviations in footnote.”

**Answer to d:** Done

**Question e:** “Need to be more clear about exclusions: what were irrelevant results? Or out of context? »

**Answer to e:** Irrelevant results and out-of-context studies are described on page 11 in the subsection on study selection.

**Question f:** “Table 1 is interesting, however very descriptive and there is no standard presentation of outcomes… it would be interesting to better describe that and include it as a limitation (variety of outcomes that were not compared- no possible meta-analysis??)”

**Answer to f:** We categorized the variables into major groups within each article. This is how the variables were organized. It is true that the lack of a meta-analysis is a limitation of our study.

**Question g:** “Discussion should clearly state what variables are specific or different from other continents and what is similar in other settings, especially LMIC.”

**Answer to g:** Done
